# Targeted school‐based interventions for improving reading and mathematics for students with, or at risk of, academic difficulties in Grades 7–12: A systematic review

**DOI:** 10.1002/cl2.1081

**Published:** 2020-04-01

**Authors:** Jens Dietrichson, Trine Filges, Rasmus H. Klokker, Bjørn C. A. Viinholt, Martin Bøg, Ulla H. Jensen

**Affiliations:** ^1^ VIVE—The Danish Center for Social Science Research Copenhagen Denmark; ^2^ Lundbeck A/S Copenhagen Denmark; ^3^ Professionshøjskolen Absalon Roskilde Denmark

## 1 PLAIN LANGUAGE SUMMARY

### 1.1 Targeted school‐based interventions improve achievement in reading and maths for at‐risk students in Grades 7–12

School‐based interventions targeting students with, or at risk of, academic difficulties in Grades 7–12 have on average positive effects on standardised tests in reading and maths. The most effective interventions have the potential to considerably decrease the gap between at‐risk and not‐at‐risk students. Effects vary substantially between interventions, however, and the evidence for using certain instructional methods or targeting certain domains is weaker.

### 1.2 What is this review about?

Low levels of literacy and numeracy skills are associated with a range of negative outcomes later in life, such as reduced employment, earnings and health. This review examines the effects of a broad range of school‐based interventions targeting students with, or at risk of, academic difficulties on standardised tests in reading and maths. Included interventions changed instructional methods by, for example, using peer‐assisted learning, introducing financial incentives, giving instruction in small groups, providing more progress monitoring, using computer‐assisted instruction (CAI) and giving teachers access to subject‐specific coaching.

Some interventions targeted specific domains in reading and maths, such as reading comprehension, fluency and algebra, while others focused on building for example meta‐cognitive and social‐emotional skills.

**Table:** 

This Campbell systematic review examines the effects of targeted school‐based interventions on standardised tests in reading and maths. The review analyses evidence from 71 studies, 52 of which are randomised controlled trials.

### 1.3 What studies are included?

Included studies examine targeted school‐based interventions that tested effects on standardised tests in reading and maths for students in Grades 7–12 in regular schools. The students either have academic difficulties, or are deemed at risk of such difficulties on the basis of their background. The interventions are targeted as they aim to improve achievement for these groups of students, and not all students.

The review summarises findings from 71 studies. Of these, 59 are from the United States, four from Canada, three from the UK, two from Germany, two from the Netherlands and one from Australia.

Fifty‐two studies are randomised controlled trials (RCTs) and 19 are quasiexperimental studies (QESs).

### 1.4 What are the main findings of this review?

The interventions studied have on average positive and statistically significant short‐run effects on standardised tests in reading and maths. This effect size is of an educationally meaningful magnitude, for example, in relation to the gap between groups of at‐risk and not‐at‐risk students. This means that the most effective interventions have the potential of making a considerable dent in this gap.

Only seven included studies tested effects more than three months after the end of intervention, and there is, therefore, little evidence of longer‐run effects.

Effects are very similar across reading domains. Interventions have larger effects on standardised tests in maths than on reading tests. Small group instruction has significantly larger effect sizes than CAI and incentive components.

### 1.5 What do the findings of this review mean?

The review provides support for school‐based interventions for students with, or at risk of, academic difficulties in Grades 7–12. However, the results do not provide a strong basis for prioritising between earlier and later interventions. For that, estimates of the long‐run cost‐effectiveness of interventions would be needed.

The lack of long‐run evidence should not be confused with a lack of effectiveness. We simply do not know whether the short‐run effects are lasting. More research about long‐run effects would therefore be a welcome addition to the literature.

More research is also needed from non‐English speaking countries; a large share of the included studies is from the United States, Canada, or the UK. There are also more interventions that have been tested by reading tests than maths tests, and interventions targeting maths seem like a promising research agenda.

Many studies are not included in the meta‐analysis due to low methodological quality. The most important improvement to research designs would be to increase the number of units and students in intervention and control groups. Lastly, the instruction given to control groups is often not described in detail. Variation in control group instruction is therefore difficult to analyse and a likely source of the effect size variation.

### 1.6 How up‐to‐date is this review?

The review authors searched for studies up to July 2018.

## 2 EXECUTIVE SUMMARY/ABSTRACT

### 2.1 Background

Low levels of numeracy and literacy skills are associated with a range of negative outcomes later in life, such as reduced earnings and health. Obtaining information about effective interventions for educationally disadvantaged youth is therefore important.

### 2.2 Objectives

The main objective was to assess the effectiveness of interventions targeting students with or at risk of academic difficulties in Grades 7–12.

### 2.3 Search methods

We searched electronic databases from 1980 to July 2018. We searched multiple international electronic databases (in total 14), seven national repositories, and performed a search of the grey literature using governmental sites, academic clearinghouses, and repositories for reports and working papers, and trial registries (10 sources). We hand searched recent volumes of six journals and contacted international experts. Lastly, we used included studies and 23 previously published reviews for citation tracking.

### 2.4 Selection criteria

Studies had to meet the following criteria to be included:

- Population: The population eligible for the review included students attending regular schools in Grades 7–12, who were having academic difficulties, or were at risk of such difficulties.
- Intervention: We included interventions that sought to improve academic skills, were performed in schools during the regular school year, and were targeted (selected/indicated).
- Comparison: Included studies used a treatment‐control group design or a comparison group design. We included RCTs, quasirandomised controlled trials (QRCTs) and QESs.
- Outcomes: Included studies used standardised tests in reading or mathematics.
- Setting: Studies carried out in regular schools in an OECD country were included.

### 2.5 Data collection and analysis

Descriptive and numerical characteristics of included studies were coded by members of the review team. A review author independently checked coding. We used an extended version of the Cochrane Risk of Bias tool to assess risk of bias. We used random‐effects meta‐analysis and robust‐variance estimation procedures to synthesise effect sizes. We conducted separate meta‐analyses for tests performed within three months of the end of interventions (short‐run effects) and longer follow‐up periods. For short‐run effects, we performed subgroup and moderator analyses focused on instructional methods and content domains. Sensitivity of the results to effect size measurement, outliers, clustered assignment of treatment, missing values, risk of bias and publication bias was assessed.

### 2.6 Results

We found 24,411 potentially relevant records and screened 4,244 in full text. In total 247 studies met our inclusion criteria and we included 71 studies in meta‐analyses. The reasons for not including studies in the meta‐analyses were that they had too high risk of bias (118), compared two alternative interventions (38 studies), lacked necessary information (13 studies), or used overlapping samples (7 studies). Of the 71 studies, 99 interventions, and 214 effect sizes included in the meta‐analysis, 76% were RCTs, and the rest QESs. The total number of student observations in the analysed studies was around 105,700. The target group consisted of, on average, 47% girls, 73% minority students, and 62% low income students. The mean grade was 8.3. Most studies included in the meta‐analysis had a moderate to high risk of bias.

The average effect size for short‐run outcomes was positive and statistically significant (weighted average effect size [ES] = 0.22, 95% confidence interval [CI] = [0.148, 0.284]). The effect size corresponds to a 56% chance that a randomly selected score of a student who received the intervention is greater than the score of a randomly selected student who did not. All measures indicated substantial heterogeneity across effect sizes. Seven studies included follow‐up outcomes. The average effect size was small and not statistically significant (ES = 0.05, 95% CI = [−0.096, 0.192]), but there was substantial variation.

We focused the analysis of comparative effectiveness on the short‐run outcomes and two types of intervention components: instructional methods and content domains. Interventions that included small group instruction (ES = 0.38, 95% CI = [0.211, 0.547]), peer‐assisted instruction (ES = 0.19, 95% CI = [0.061, 0.319]), progress monitoring (ES = 0.19, 95% CI = [0.086, 0.290]), CAI (ES = 0.17, 95% CI = [0.043, 0.309]) and coaching of personnel (ES = 0.10, 95% CI = [0.038, 0.166]) had positive and significant average effect sizes. Interventions that provided incentives for students did not have a significant average effect size (ES = 0.05, 95% CI = [−0.103, 0.194]). The average effect size of interventions that included none of the above components, but for example provided extra instructional time, instruction in groups smaller than whole class but larger than 5 students, or just changed the content had a relatively large, but statistically insignificant effect size (ES = 0.20, 95% CI = [−0.002, 0.394]).

The differences between effect sizes from interventions targeting different content domains were mostly small. Interventions targeting fluency, vocabulary, multiple reading areas, meta‐cognitive, social‐emotional, or general academic skills, comprehension, spelling and writing, and decoding had average effect sizes ranging from 0.14 to 0.22, all of them statistically significant. Effect sizes based on mathematics tests had a relatively large effect size (ES = 0.34, CI = [0.169, 0.502]).

Including all instructional methods and moderators without missing observations in meta‐regressions revealed that effect sizes based on mathematics tests were significantly larger than effect sizes based on reading tests, and QES showed significantly larger effect sizes than RCTs. Small group instruction was associated with significantly larger effect sizes than CAI and incentive components. The unexplained heterogeneity remained substantial throughout the comparative effectiveness analysis.

### 2.7 Authors’ conclusions

We found evidence of positive and statistically significant average effects of educationally meaningful magnitudes (and no significant adverse effects). The most effective interventions in our sample have the potential of making a considerable dent in the achievement gap between at‐risk and not‐at‐risk students. The results thus provide support for implementing school‐based interventions for students with or at risk of academic difficulties in Grades 7–12.

We want to stress that our results do not provide a strong basis for prioritising between earlier and later interventions. For that, we would need estimates of the long‐run cost‐effectiveness of interventions and evidence is lacking in this regard. Furthermore, there was substantial heterogeneity throughout the analyses that we were unable to explain by observable intervention characteristics.

## 3 BACKGROUND

### 3.1 The issue

Across countries, a large proportion of students leave secondary school without the skills and qualifications needed to succeed in the labour market. In the member countries of the Organisation for Economic Co‐operation and Development (OECD), 16% of all youth between 25 and 34 years of age have not earned the equivalent of an upper secondary education or high school degree (OECD, 2016a). According to the results from the Programme for International Student Achievement (PISA), on average around 20–25% of the participants are not proficient in reading and mathematics as 15 year olds (OECD, 2016b, 2019). Whilst the proportion of students that are not proficient in reading and mathematics is lower in some countries, it remains around 10% even in the best performing countries (OECD, 2016b, 2019). Thus, the share of students with academic difficulties is substantial in all OECD countries.

Entering adulthood with a low level of educational attainment is not only associated with reduced employment and financial prospects (De Ridder et al. 2012; Johnson, Brett, & Deary, 2010; Scott & Bernhardt, 2000), it is also associated with numerous health problems and risk behaviours, such as drug use and crime, which have serious implications for the individual as well as for society (Berridge, Brodie, Pitts, Porteous, & Tarling, 2001; Brook, Stimmel, Zhang, & Brook, 2008; Feinstein, Sabates, Anderson, Sorhaindo, & Hammond, 2006; Horwood et al., 2010; Sabates, Feinstein, & Shingal, 2013). Improving the educational attainment and achievement for students with academic difficulties is therefore important.

The group of students who experiences academic difficulties is diverse. It includes for instance students with learning disabilities, students who are struggling because they lack family support, because they have emotional or behavioural problems, or because they are learning the first language of the country they are living in. Some groups of students may not currently have academic difficulties but are “at risk” in the sense that they are in danger of ending up with difficulties in the future, at least in the absence of intervention (McWhirter, McWhirter, McWhirter, & McWhirter, 2004). Although being at risk points to a future negative situation, “at risk” is sometimes used to designate a current situation (McWhirter et al., 2004; Tidwell & Corona Garret, 1994), as current academic difficulties are a risk factor for future difficulties and having difficulties in one area may be a risk factor in other areas (McWhirter, McWhirter, McWhirter, & McWhirter, 1994). Separating students with and at risk of academic difficulties is therefore sometimes difficult.

Test score achievement gaps are typically present well before secondary school (e.g., Heckman 2006; Lipsey et al., 2012; von Hippel, Workman, & Downey, 2018), and there are often large differences in risk factors for academic difficulties before children start primary school. For example, the gap between majority and minority ethnic children on cognitive skills tests is apparent when children are as young as 3–4 years old (e.g., Burchinal et al., 2011; Fryer & Levitt, 2013). Low‐income preschool children can have more behaviour problems (e.g., Huaqing & Kaiser, 2003) and there is a strong continuity between emotional and behavioural problems in preschool and psychopathology in later childhood (Link Egger & Angold, 2006). Emotional and behavioural problems are in turn linked to lower academic achievement in school (e.g., Durlak, Weissberg, Dymnicki, Taylor, & Schellinger, 2011; Taylor, Oberle, Durlak, & Weissberg, 2017). Struggling readers tend to be persistently behind their peers from the early grades (e.g., Elbro & Petersen, 2004; Francis, Shaywitz, Stuebing, Shaywitz, & Fletcher, 1996) and early math and language abilities strongly predict later academic achievement (e.g., Duncan et al., 2007; Golinkoff, Hoff, Rowe, Tamis‐Lemonda, & Hirsh‐Pasek, 2018).

The prenatal and early childhood environment appears to be an important factor that keeps students from realising their academic potential (e.g., Almond, Currie, & Duque, 2018).^1^ Currie (2009) furthermore documented that children from families with low socioeconomic status (SES) have worse health, including measures of foetal conditions, physical health at birth, incidence of chronic conditions and mental health problems. Immigrant and minority children are often overrepresented among low SES families and face similar risks (e.g., Bradley & Corwyn, 2002; Deater–Deckard, Dodge, Bates and Pettit, 1998; Morgan, Farkas, Hillemeier, & Maczuga, 2012).

Family environments also differ in aspects thought to affect educational achievement. Low SES families are less likely to provide a rich language and literacy environment (Bus, Van IJzendoorn, & Pellegrini, 1995; Golinkoff et al., 2018; Hart & Risley, 2003). The parenting practices and access to resources such as early childhood education, health care, nutrition, and enriching spare‐time activities also differ between high and low risk groups (e.g., Esping‐Andersson et al., 2012; Morgan et al., 2012). Low SES parents also seem to have lower academic expectations for their children (Bradley & Corwyn, 2002; Slates, Alexander, Entwisle, & Olson, 2012), and teachers have lower expectations for low SES and minority students (e.g., Good, Aronson, & Inzlicht, 2003; Timperley & Phillips, 2003). Furthermore, low SES children are more likely to experience a decline in motivation during the course of primary, secondary, and upper secondary school (Archambault, Eccles, & Vida, 2010).

The neighbourhoods students grow up in is another potential determinant of achievement (e.g., Campbell, Shaw, & Gilliom, 2000; Chetty, Friedman, Hendren, Jones, & Porter, 2018; Chetty, Hendren, & Katz, 2016). It seems likely that many students in high risk groups live in neighbourhoods that are less supportive of high educational achievement in terms of, for example, peer support and role models. To get by in a disadvantaged neighbourhood may also require a very different set of skills compared to what is needed to thrive in school, something which may increase the risk that pupils have trouble decoding the “correct” behaviour in educational environments (e.g., Heller et al., 2017). Regarding the relative importance of families and neighbourhoods, the review in Björklund & Salvanes (2011) indicates that family resources are the more important explanatory factor.

After this review of risk factors for academic difficulties, it is worth noting that the life circumstances placing children and youth at risk are only partially predictive. That is, risk factors increase the probability of a having academic difficulties, but are not deterministic. As academic difficulties therefore cannot be perfectly predicted and may show up relatively late in a child's life, early interventions may not be enough and effective interventions in all grades may be needed to reduce the achievement gaps substantially.

As the test score gaps between high and low risk groups remain relatively stable from the early grades, schools do not seem to be a major reason for the inequality in academic achievement (e.g., Heckman 2006; Lipsey et al., 2012; von Hippel et al. 2018). Further evidence is provided by the seasonality in achievement gaps. In the United States, the gap between high and low SES students tends to widen during summer breaks when schools are out of session (e.g., Alexander, Entwisle, & Olson, 2001; Gershenson, 2013; Kim & Quinn, 2013; although von Hippel et al., 2018, show that this pattern is not universal across risk groups, grades and cohorts). However, the stability of the test score gaps also implies that current school practice is not, in general, enough to decrease the achievement gaps. As schools are perhaps the societal arena where most children and youth can be reached, finding effective school‐based interventions for students with or at risk of academic difficulties is a question of major importance.

### 3.2 The intervention

This review focusses on interventions that are targeted at students with or at risk of academic difficulties and that aim to improve students’ academic achievement. In line with the diversity of reasons for ending up with a low level of skills and educational attainment, we included interventions targeting students who for a broad range of reasons were having academic difficulties, or were at risk of such difficulties. We prioritised already having difficulties over belonging to an at risk group in the sense that if there was information about for example test scores, grade point averages, or low attendance, we did not require information about at risk status. Furthermore, we did not include interventions targeting high‐performing students in groups that may otherwise be at risk.

Interventions aimed at improving academic achievement are numerous and very diverse in terms of intervention focus, target group, and mode of delivery. This review focused on targeted interventions performed in schools and provided to students with or at risk of academic difficulties in Grades 7–12 (ages range from 12–14 to 17–19, depending on country/state), where academic skill building and learning were primary intervention aims.

Many targeted interventions are delivered individually as a supplement to regular classes and school activities. However, targeted interventions can be delivered in various settings, including in class (e.g., paired reading interventions or the Xtreme Reading programme) or in group sessions (e.g., the READ 180 programme). This review restricts the settings to school‐based interventions, by which we mean interventions delivered in school, during the regular school year, and where schools are a key stakeholder. This restriction excludes for example after‐school programmes, summer camps and summer reading programmes, and interventions involving only parents and families (see, e.g., Zief, Lauver, & Maynard, 2006 for a review of after school‐programmes, Kim & Quinn, 2013, for a review of summer reading programmes, and Jeynes, 2012, for a review of programmes that involve families or parents).

We include a wide range of interventions that aim to improve the academic achievement of students by either changing the method of instruction—such as tutoring, peer‐assisted learning or CAI interventions—or by changing the content of the instruction—for instance, interventions emphasising mathematical problem solving skills, reading comprehension or meta‐cognitive and social‐emotional skills. Many interventions involve changes to both teaching method and content of instruction, and very often consist of several major programmatic components. Thus, interventions were included in this review based on their aim to improve academic achievement of students with or at risk of academic under achievement and not on the type of components (or mechanisms) used in the intervention.

For this reason, the review excludes interventions that may improve academic achievement as a side‐effect, but do not state academic achievement as an explicit aim. For example, interventions where improvement in behavioural or social‐emotional outcomes are the primary aim of the intervention, like Classroom Management or the SCARE programme, are not included. However, interventions with behavioural and social‐emotional components may very well have academic achievement as one of their primary aims, and use standardised tests of reading and mathematics as one of their primary outcomes. If this is the case, and achievement is a primary outcome, such interventions *are* included. Thus, the content of the programme is less important than the primary outcome (academic achievement) and the target population (students with or at risk of academic difficulties).

Universal interventions which aim to improve the quality of the common learning environment at school in order to raise academic performance of all students (including average and above average students) are excluded. Whole‐school reform strategy concepts such as Success for All, curriculum‐based programmes like Elements of Mathematics (EMP), as well as reduced class size interventions and general professional development interventions for principals and teachers that do not target at‐risk students were also excluded. However, we do include interventions with a professional development component, for example, in the form of coaching of teachers during the implementation, as long as the intervention specifically targeted students with or at risk of academic difficulties.

### 3.3 How the intervention might work

Given the spectrum of interventions that are included in this review, it is unsurprising that they represent a range of diverse strategies to achieve improvement in academic outcomes. This diversity reflects the varying reasons that might explain why students are struggling, or are at risk. In turn, the theoretical background for the interventions also vary. It is therefore not possible to specify one particular theory of change or one theoretical framework for this review. Instead, we briefly review three theoretical perspectives that characterise the majority of the included interventions. We also discuss and exemplify how targeted interventions may address some of the reasons for academic difficulties in light of the theoretical perspectives.

#### 3.3.1 Theoretical perspectives

The reasons why students may be struggling are multifaceted and the theoretical perspectives underlying interventions are therefore likely to be broad. Nevertheless, three superordinate components are characteristic for the majority of the included interventions. These components can be abridged to:

- Adaptation of behaviour (social learning theory).
- Individual cognitive learning (cognitive developmental theory).
- Alteration of the social learning environment (pedagogical theory).

We emphasise that the following presentation of theoretical perspectives is not all‐encompassing and although components are presented as demarcated, they contain some conceptual overlap.

*Social learning theory* has its origins in social and personality psychology and was initially developed by psychologist Julian Rotter and further developed especially by Bandura (1977; 1986). From the perspective of social learning theory, behaviour and skills are primarily learned by observing and imitating the actions of others, and behaviour is in turn regulated by the recognition of those actions by others (reinforcement) or discouraged by a lack of recognition or sanctions (punishment). According to social learning theory, creating the right social context for the student can therefore stimulate more productive behaviour through social modelling and reinforcement of certain behaviours that can lead to higher achievement.

*Cognitive developmental theory* is not one particular theory, but rather a myriad of theories about human development that focus on how cognitive functions such as language skills, comprehension, memory and problem‐solving skills enable students to think, act and learn in their social environment. Some theories emphasise a concept of intelligence where children gradually come to acquire, construct, and use cognitive functions as the child naturally matures with age (e.g., Perry, 1999; Piaget, 2001). Other theories hold a more socio‐cultural view of cognitive development and use a more culturally distinct and individualised concept of intelligence that, to a greater extent, includes social interaction and individual experience as the basis for cognitive development. Examples include the theories of Sternberg (2009) and Gardner (1999).

*Pedagogical theory* draws on different disciplines in psychology and social theory such as cognitivism, social‐interactional theory and socio‐cultural theory of learning and development. There is not one uniform pedagogical model, but examples of contemporary models in mainstream pedagogy are concepts such as Scaffolding (Bruner, 2006) and the Zone of Proximal Development (Vygotsky, 1978), which originate in developmental and educational psychology. These theoretical positions hold that learning and development emerge through practical activity and interaction. Acquisition of new knowledge is therefore considered to be dependent on social experience and previous learning, as well as the availability and type of instruction. Accordingly, school interventions require educators to interact and organise the learning environment for the student in certain ways to fit the individual student's needs and potential for development.

#### 3.3.2 Interventions in practice

School interventions affect academic achievement either by changing the methods by which instruction is given (the instructional method), or by changing the content of what is taught (the content domain). Many interventions combine several programmatic components as well as theoretical perspectives. Examples from included interventions of instructional methods are small group instruction, coaching of teachers, peer‐assisted instruction, CAI, incentives and increased progress monitoring. Included reading interventions target content domains such as comprehension, fluency, decoding and vocabulary (most targeted more than one reading domain). Mathematics interventions target the following domains: operations (e.g., addition, subtraction and multiplication), word problems, fractions, algebra and general math proficiency (or multiple components). Some interventions target both mathematics and reading, and some target other types of skills, such as social‐emotional skills or meta‐cognitive strategies (e.g., learning how to learn, or self‐regulation strategies).

Earlier research indicates that different types of interventions can improve the academic achievement of students with or at risk of difficulties, both across methods, delivery mode, age group and duration (e.g., Cheung & Slavin, 2012; Dietrichson et al., 2017; Gersten et al., 2009; Slavin & Madden, 2011). For example, both reading strategy instruction and peer‐mediated learning programmes such as paired reading have shown positive effects on the literacy skills of struggling secondary school readers. These are two types of programmes that clearly have different components and delivery modes (Edmonds et al. 2009). In another example, Good et al. (2003) showed that changing expectations of seventh grade students at risk for stereotype‐based underperformance (minority and low‐income students in general, and girls regarding mathematics) can improve standardised test scores.

Furthermore, interventions, such as tutoring and structured peer‐assisted interventions, often have in common that they comprise an eclectic theoretical model that combines components from all three perspectives on learning presented in the previous section. They are comprehensive interventions that rely on a complexity of mechanisms such as increased feedback and tailor‐made instruction (pedagogical theory), regulation of behaviour by, for example, rewards or close interaction with role models (social learning theory), and development of meta‐cognitive functions such as learning how to learn (cognitive developmental theory).

Another way of viewing these and other types of interventions is that they attempt to address the differential family and neighbourhood resources of students with high and low risk of academic difficulties. Low risk students are more likely to have access to “tutors” all year round, in the form of parents, siblings and other family members who help out with homework and schoolwork. Interventions to change mindsets, improve expectations, and mitigate stereotype threat also target areas where low risk students may have an advantage, as low risk students are less likely to be subject to stereotype threat, and their families and teachers may already be teaching their children growth mindsets and have favourable expectations of their academic achievement. Different types of extrinsic rewards may be a way to bolster motivation, which may be especially important for students whose families place less weight on educational achievement.

Furthermore, if, as indicated in the previous section, the differences between high and low risk students can be understood as a consequence of differential access to a combination of resources, remedial efforts may need to address several problems at once in order to be effective. Programmes that combine certain components may therefore be more effective than others. Another reason to examine combinations of components relates to an often‐suggested explanation for missing impacts: lack of motivation among participants (e.g., Edmonds et al. 2009). It is therefore possible that interventions will be more effective if they also include some form of reward for participating students and implementing teachers, along with other components providing, for instance, pedagogical support.

For struggling students in Grades 7–12 (ages range from 12–14 to 17–19, depending on country), who are likely to have a history of low achievement, finding the right combination of intervention components may be especially pertinent (e.g., Edmonds et al. 2009). Some researchers have recommended, based on the perceived low relative cost‐effectiveness of interventions directed to adolescents, that resources should disproportionally be used for early interventions (e.g., Esping‐Andersen, 2004, Heckman, 2006), or that secondary schools should primarily be providing technical and vocational training for disadvantaged teenagers (Cullen, Levitt, Robertson, & Sadoff, 2013). However, Cook et al. (2014) argued that the low cost‐effectiveness may be a premature conjecture, as previous interventions for youths have often not combined the fostering of academic skills with other important factors for academic success, such as social‐cognitive skills. As, for example, social information processing programmes (Wilson & Lipsey, 2006a; 2006b), and programmes based on cognitive behavioural therapy (e.g., Lipsey, Landenberger, & Wilson, 2007) have been found to effectively reduce problematic behaviour and promote social‐cognitive skills, combinations with more academically oriented interventions may yield complementary effects.

### 3.4 Why it is important to do the review

In this section we first discuss earlier related reviews, and then the contributions of this review in relation to the earlier literature.

#### 3.4.1 Prior reviews

In some regards, this review shares common ground with existing Campbell reviews and reviews in progress such as “Impacts of After‐School Programs on Student Outcomes: A Systematic Review” (Zief et al. 2006), “Dropout Prevention and Intervention Programs: Effects on School Completion and Dropout among School‐aged Children and Youth” (Wilson, Tanner‐Smith, Lipsey, Steinka‐Fry, & Morrison, 2011) and “Effects of College Access Programs on College Readiness and Enrollment” (Harvill et al., 2012).

Nevertheless, this review differs in substantial ways from existing Campbell reviews. First, with the exception of Zief et al. (2006), the listed reviews do not explicitly target an educationally disadvantaged or low performing student population. Zief et al. (2006) on the other hand excluded interventions performed outside North America, and three of the five studies included were of programmes primarily designed to reduce negative behaviours such as delinquency and drug use. That is, the programmes did not target academic achievement as their primary outcome. Although Wilson et al. (2011) did not explicitly include only interventions that targeted students with or at risk of academic difficulties, many of the studies in their review of dropout prevention programmes included similar at‐risk groups as our review. The major difference between their review and ours is that they focused on programmes of school completion and dropout prevention, and outcome measures such as dropout and graduation rates. There is some overlap between the types of interventions included but also clear differences. For instance, none of the interventions included in our review involved components such as paid employment for students, community service programmes, or vocational training.

In addition to these Campbell reviews, there are other related reviews with a similar broad scope and a target group overlapping with ours to some degree. Slavin, Lake, and Groff (2009) reviewed programmes in middle and high school mathematics, whereas Slavin et al. (2008) reviewed reading programmes for middle and high schools. Fryer (2016) surveys randomised field experiments in all areas of education. However, these reviews included a broader range of programmes and student groups, not programmes targeting at‐risk or low‐performing students. Furthermore, Wanzek et al. (2006; including students in Grades 6–12) reviewed reading interventions directed at students in grades K‐12 with learning disabilities, and Edmonds et al. (2009; Grades 6–12), Flynn et al. (2012; Grades 5–9), and Scammaca et al. (2015; Grades 4–12), Wanzek et al. (2013; Grades 4–12) and Baye et al. (2018; Grades 6–12) reviewed interventions for struggling readers. These reviews thus covered low achieving students, but not other at‐risk students or areas other than reading. Gersten et al. (2009) examined four types of components of mathematics instruction for students with learning disabilities but did not include interventions for students at risk (or more general reasons for low performance than learning disabilities). Dietrichson et al. (2017) on the other hand included studies in both reading and mathematics and based inclusion on the proportion of students with low SES, but did not consider whether students had academic difficulties or not.

In terms of findings related to this review's primary outcome measures, the reviews that have focused on the effects of academic interventions on reading test scores all showed positive overall effect sizes, although there was considerable variation between the interventions in all of these reviews (Baye et al., 2018; Edmonds et al., 2009; Flynn et al., 2012; Scammaca et al., 2015; Slavin et al., 2008; Slavin et al., 2009; Wanzek et al., 2006, 2013). The six reviews of reading interventions directed to struggling readers reported positive effects in general but few reliable differences between effect sizes over reading domains (Edmonds et al., 2009; Flynn et al., 2012; Scammaca et al., 2015; Wanzek et al., 2006, 2013). An exception is that reading comprehension interventions were associated with significantly higher effect sizes than fluency interventions in Scammaca et al. (2015), but this difference disappeared when only standardised measures were considered. Baye et al. (2018) classified interventions by their main component and found the largest effects for tutoring by paid adults. Cooperative learning, whole‐school approaches, and approaches focused on writing, content, strategy‐focused, personalisation and group/personalisation rotation had positive effects of relatively similar magnitudes (we compare our results, including the magnitudes of effects, to those found in the earlier literature in section Agreements and disagreements with other studies or reviews).

Gersten et al. (2009) examined four components of mathematics instruction for students with learning disabilities, and found most support for approaches to instruction (e.g., explicit instruction, use of heuristics) and/or curriculum design, and providing formative assessment data and feedback to teachers. Dietrichson et al. (2017) examined interventions that have used standardised tests in reading and mathematics and categorised 14 intervention components mainly delimited by the instructional methods used. Tutoring, feedback and progress monitoring, and cooperative learning had the largest and most robust average effect sizes.

The best evidence syntheses by Slavin et al. (2008, 2009) both point to instructional‐process programmes, especially programmes that incorporate cooperative learning, as having larger effects than curriculum‐based interventions and CAI programmes. Slavin et al. (2009) found no indication that effect sizes differed between socioeconomically disadvantaged students and nondisadvantaged students. However, only a relatively small subset of studies reported results differentiated by SES, and the review did not contain information about whether the programmes that showed the largest effect sizes also had the largest effect sizes for disadvantaged students. Fryer (2016) found that high dosage tutoring and “managed professional development” programmes (e.g., Success for All, Reading Recovery) had the largest effect sizes of experiments conducted in schools.

Slavin et al. (2009) and Edmonds et al. (2009) reported that some programmes, which have been shown to be effective for younger students, may have smaller or no effects for older students. Effect sizes were smaller for older students also in Scammaca et al. (2015), although not significantly different. Fryer (2016) on the other hand concludes that “high dosage tutoring of adolescents seems to be as effective—if not more effective—than early childhood investments” (p. 78). Neither the question of whether interventions are less effective for older students, nor which components of interventions that are most important was settled in the reviews covered in this section.

#### 3.4.2 The contribution of this review

Academic difficulties and lack of educational attainment are significant societal problems. Moreover, as shown by the Salamanca declaration from 1994 (UNESCO, 1994), there has been a great, and decades long, interest among policy makers in improving the inclusion of students with academic difficulties in mainstream schooling, and a desire to increase the number of empirically supported interventions for these student groups.

The main objective of this review is to provide policy makers and educational decision‐makers at all levels—from governments to teachers—with evidence of the effectiveness of interventions aimed to improve the academic achievement of students with or at risk of academic difficulties in Grades 7–12. To this end, we compared the effects of interventions that differ in both instructional methods as well as the nature of the content taught.

We chose a broad scope in terms of the target group and type of intervention. We included interventions where the effects were measured by standardised tests in reading and mathematics because many interventions are not directed specifically to either subject and outcomes are therefore often measured in both (Dietrichson et al., 2017). Reading and mathematics are furthermore fundamental skills, which are important in more or less all school subjects and highly correlated with future educational and labour market success (OECD, 2016c). Earlier reviews of interventions aimed at similar target groups (e.g., Dietrichson et al., 2017; Gersten et al. 2009) have provided tentative evidence that similar types of interventions were effective for both struggling and low SES students, but stronger evidence of this is needed. In order to provide as complete a picture of the evidence as possible, the type of intervention and the target group were both kept deliberately broad. Including both students with and at risk of academic difficulties in the target group should decrease the risk of biasing the results due to omission of studies where information about either academic difficulties or at‐risk status is available, but not both. Furthermore, making comparisons between intervention components within one review, rather than across reviews, should increase the likelihood of a fair comparison. It is easier within the scope of one review (rather than across reviews) to ensure that effect sizes are calculated in the same way, the definitions of intervention components are consistent, and that moderators are coded in the same way.

In isolation, this last argument suggests that all interventions aiming to improve educational achievement for our target population should be included. However, we also wanted to explore if certain interventions work better than others. The results in the reviews of Slavin et al. (2008, 2009) and Dietrichson et al. (2017), for example, point to substantial variation in effect sizes of test scores in reading and mathematics. Importantly, considerable variation was also found within types of interventions. For the exploration of variation in effect sizes, a broad scope review may be a disadvantage, as information about moderators that are important in order to explain variation for some types of interventions are not relevant for others. We have therefore limited the included interventions to those that are targeted, rather than universal, and performed in a regular school situation during the regular school year. This delimitation increases the probability that potentially important moderators are reported in a comparable way.

Earlier reviews with a comparable focus have either not included intervention components together with other moderators in a meta‐regression, or only included broad categories of interventions. This risks confounding the effects of intervention components with for example participant characteristics. Furthermore, some reviews have coded interventions regarding the instructional methods used, or regarding the type of content taught, but not both (e.g., Dietrichson et al., 2017; Gersten et al., 2009; Scammaca et al., 2015). These analyses risk confounding methods of instruction with content.

## 4 OBJECTIVES

The objective of this review was to assess the effectiveness of targeted interventions aimed at improving the academic achievement for students with or at risk of academic difficulties in Grades 7–12.

The analysis focused on the comparative effectiveness of different types of interventions. We attempted to identify those intervention components that have the largest and most reliable effects on academic outcomes, as measured by standardised test scores in reading and mathematics. In addition, we explored evidence of differential effects for students with different characteristics, for example, in relation to grade. We also examined moderators related to study design, measurement of effect sizes and the duration of interventions.

## 5 METHODS

### 5.1 Criteria for considering studies for this review

#### 5.1.1 Types of studies

According to our protocol, included studies should use a treatment‐control group design or a comparison group design (Dietrichson, Bøg, Filges, & Klint Jørgensen, 2016). Included study designs were RCTs, including cluster‐RCTs; QRCTs, where participants are allocated by means such as alternate allocation, person's birth date, the date of the week or month, case number or alphabetical order (we found no such designs though); and QES. To be included, QES had to credibly demonstrate that outcome differences between intervention and control groups is the effect of the intervention and not the result of systematic baseline differences between groups. That is, selection bias should not be driving the results. This assessment is included as a part of the risk of bias tool, which we elaborate on in the Risk of Bias section below. A fair amount of studies within educational research use single group pre‐post comparisons (e.g., Edmonds et al., 2009; Wanzek et al., 2006); such studies were however not included due to the higher risk of bias.

Control groups received treatment‐as‐usual (TAU) or a placebo treatment. We found no studies where the control group explicitly received nothing (i.e., a no‐treatment control), as everybody experienced regular schooling. That is, control groups got whatever instruction the intervention group would have gotten, had there not been an intervention. The TAU condition can for this reason differ substantially between studies (although many studies did not describe the control condition in much detail). Eligible types of control groups included also waiting list control groups, in which the control group also receives the intervention after the posttest.

Comparison designs compared alternative interventions against each other; that is, they made it clear that all students get something other than TAU *because* of the intervention. Effect sizes from such studies are not fully comparable to effect sizes from treatment‐control designs. We therefore planned to analyse comparison designs separately from treatment‐control designs, and use them where they may shed light on an issue, which could not be fully analysed using the sample of treatment‐control studies. However, the number of studies that were, in this sense, relevant was small and we refrained from analysing comparison designs further.

#### 5.1.2 Types of participants

The population samples eligible for the review included students attending regular schools in Grades 7–12, who were having academic difficulties, or were at risk of such difficulties.

Students attending regular private, public, and boarding schools were included, and students receiving special education services within these school settings were also included. Grades 7–12 corresponds roughly to secondary school, defined as the second step in a three‐tier educational system consisting of primary education, secondary education and tertiary or higher education. We included studies with a student population in grades lower than 7–12 as long as the majority of the students were in Grades 7–12. The age range included differed between countries, and sometimes between states within countries. Typically, ages ranged from 12–14 to 17–19 years (fewer studies reported ages than grades though, which was also the main reason to focus inclusion on grades rather than age).

The eligible student population included both students identified in the studies by their observed academic achievement (e.g., low academic test results, low grade point average or students with specific academic difficulties such as learning disabilities), and students that were identified primarily on the basis of their educational, psychological or social background (e.g., students from families with low SES, students placed in care, students from minority ethnic/cultural backgrounds and second language learners). We excluded interventions only targeting students with physical learning disabilities (e.g., blind students), students with dyslexia/dyscalculia, and interventions that were specifically directed towards students with a certain neuropsychiatric disorder (e.g., autism, ADHD), as some interventions targeting such students are different from interventions targeting the general struggling or at‐risk student population.

Because there was substantial overlap between students that were already struggling and groups considered at risk of difficulties in studies found in a previous review (Dietrichson et al. 2017), we chose to include both students with difficulties and students that were deemed at risk, or were considered educationally disadvantaged. A motivating example is studies that target a high poverty area, and then randomly select a number of students with test scores below a certain level in each school that receive the intervention. These struggling students are thus likely to be low SES, but information about SES is not necessarily reported for the intervention or control group. A second example could be studies that target low performing schools, and then perform an intervention for the sub‐group of low SES students. In this case, low SES students are likely to be struggling, but this information need not be reported. Thus, choosing to include only studies that examine either students with academic difficulties or low SES students would have risked excluding studies that in all likelihood target the same or very similar student populations. We believed that the risk of biasing our results by such a choice would be larger than the possible comparison problems arising from including both students with academic difficulties and low SES students. A similar case can be made for other at risk groups, for example students from immigrant and minority backgrounds, which often partly overlap with low SES students. If the two criteria were inconsistent, we gave priority to students having academic difficulties. For example, we excluded interventions that targeted high achieving students from low income backgrounds.

Some interventions included other students, who had neither academic difficulties nor were at risk of such difficulties. For example, in some peer‐assisted learning interventions high performing students were paired with struggling students. Studies of such interventions were included if the total sample (intervention and control group) included at least 50% students that were either having academic difficulties or were at risk of developing such difficulties, or if there were separate effect sizes reported for these groups.

#### 5.1.3 Types of interventions

We included interventions that sought to improve academic achievement or specific academic skills. This does not mean that the intervention had to consist of academic activities, but there had to be an explicit expectation in the study that the intervention, regardless of the nature of the intervention content, would result in improved academic achievement or a higher skill level in a specific academic task. We however choose to exclude interventions that only sought to improve performance on a single test instead of improving a skill that would improve test scores. For similar reasons, we excluded studies of interventions where students are provided with accommodations when taking tests; for instance, when some students are allowed to use calculators and others not.

An explicit academic aim of the intervention did not per se exclude interventions that also included nonacademic objectives and outcomes. However, we excluded interventions having academic learning as a possible, not explicitly stated, secondary goal. In cases where the goals were not explicitly stated, we used the presence of a standardised test in mathematics or reading as a sign that the authors expected the intervention to improve academic achievement. We excluded cases where such tests were included but the authors explicitly stated that they did not expect the intervention to improve reading or math. Furthermore, we only included school‐based interventions; that is, interventions performed in schools during the regular school year, and where schools were one of the stakeholders. This latter restriction excluded summer reading programmes, after‐school programmes, parent tutoring programmes and other programmes delivered in the home of students.

Besides having an explicit expectation that the intervention would improve the academic performance of students, eligible interventions were also targeted (selected or indicated). That is, interventions which, in contrast to universal interventions, were aimed at certain students and/or student groups identified as having or being at risk of academic difficulties according to the definition in the previous section. Universal interventions that aimed to improve the quality of the common learning environment at the school level in order to raise academic achievement of all students (including average and above average students), were excluded. Interventions such as the one described in Fryer (2014) where a bundle of best practices were implemented at the school level in low achieving schools, where most students are struggling or at risk, was also excluded. This criterion also excluded whole‐school reform strategy concepts such as Success for All, curriculum‐based programmes like EMP, as well as reduced class size interventions.

This criterion also meant that we excluded interventions where teachers or principals receive professional development training in order to improve general teaching or management skills. Interventions targeting students with or at risk of academic difficulties may on the other hand include a professional development component, for example when a reading programme includes providing teachers with reading coaches. Such interventions were therefore included.

#### 5.1.4 Types of outcome measures

We included outcomes that cover two areas of fundamental academic skills:

- Standardised tests in reading
- Standardised tests in mathematics

Studies were only included if they considered one or more of the primary outcomes. Standardised tests included norm‐referenced tests (e.g., Gates‐MacGinitie Reading Tests and Star Math), state‐wide tests (e.g., Iowa Test of Basic Skills) and national tests (e.g., National Assessment of Educational Progress [NAEP]). If it was not clear from the description of the outcome measures in the studies, we used electronic sources to determine whether a test was standardised or not. For example, if a commercial test has been normed, this was typically mentioned on the publisher's homepage. For older tests however it was not always possible to find information about the test from electronic sources. In these cases, we included the test if there was a reference to a publication describing the test that made it clear that the test had not been developed for the intervention or study.

We restricted our attention to standardised tests in part to increase the comparability between effect sizes. Earlier related reviews of academic interventions have pointed out that effect sizes tend to be significantly lower for standardised tests compared to researcher‐developed tests (e.g., Flynn et al., 2012; Gersten et al., 2009; Scammaca et al., 2015). Scammaca et al. (2015) furthermore reported that whereas mean effect sizes differed significantly between the periods 1980‐2004 and 2005‐2011 for other types of tests, mean effect sizes were not significantly different for standardised tests. As researcher developed tests are usually less comprehensive and more likely to measure aspects of content inherent to intervention but not control group instruction (Slavin & Madden, 2011), standardised tests should provide a more reliable measure of lasting differences between intervention and control groups.

We excluded tests that provided composite results for several academic subjects, but included tests of specific domains (e.g., vocabulary, fractions) as well as more general tests, which tested several domains of reading or mathematics. Tests of subdomains had significantly larger effect sizes compared to more general tests in Dietrichson et al. (2017). This result may indicate that it may be easier to improve scores on tests of subdomains than on tests of more general skills, or that tests of subdomains may be more likely to be inherent to intervention group instruction. At the same time, it seems reasonable that interventions that target subdomains of reading and mathematics are tested on whether they affect these subdomains. Therefore, we did not want to exclude either type of test, but coded the type of test and used it as a moderator in the analysis. However, to mitigate problems with test content being inherent to intervention and not control group instruction, we did not consider tests where researchers themselves picked a subset of questions from a norm‐referenced test as being standardised. The subset should either have been predefined (as in e.g., the passage comprehension subset of Woodcock‐Johnson Tests of Achievement) or the picked by someone other than the researchers (e.g., released items from the NAEP).

We included all postintervention tests and coded the timing of each test (see the Multiple Time Points section below).

#### 5.1.5 Duration of follow‐up

Our protocol contained no initial criterion for the duration of interventions, we included interventions of all durations. Duration of the intervention was included as a moderator in some of the analyses.

#### 5.1.6 Types of settings

Only studies carried out in OECD countries were included. This selection was conducted to ensure a certain degree of comparability between school settings and to align treatment as usual conditions in included studies. For similar reasons we only included studies published in or after 1980. Due to language restrictions in the review team, we included studies written in English, German, Danish, Norwegian and Swedish.

### 5.2 Search methods for identification of studies

This section describes the search strategy for finding potentially relevant studies. We used the EPPI‐reviewer software to track the search and screening process. All searches were restricted to publication after 1980. We chose this year to balance the competing demands of comparability between intervention settings and comprehensiveness of the review. No further limiters were used in the searches. A flowchart describing the search process and specific numbers of references screened on different levels can be found in the Included Studies section.

#### 5.2.1 Electronic searches

Relevant studies were identified through electronic searches of bibliographic databases, government and policy databanks. The following electronic resources/databases were searched:

- *Academic Search Premier* (EBSCO‐host)
- *ERIC* (EBSCO‐host)
- *PsycINFO* (EBSCO‐host)
- *SocIndex* (EBSCO‐host)
- *British Education Index (EBSCO‐host)*
- *Teacher Reference Center* (EBSCO‐host)
- *ECONLIT* (EBSCO‐host)
- *FRANCIS* (EBSCO‐host)
- *ProQuest dissertation & theses A&I* (ProQuest‐host)
- *CBCA Education* (ProQuest‐host)
- *Social Science Citation Index* (ISI Web of Science)
- *Science Citation Index* (ISI Web of Science)
- *Medline* (OVID‐host)
- *Embase* (OVID‐host)

All databases were originally searched from 1st of January 1980 to 5th–7th of March 2016. We updated the searches in July 2018 using identical search strings. However, some database searches were not updated in 2018 due to access limitations. Details on searches are listed in Appendix Search Strategy by Database.

The search terms were modified to fit each resource searched. In Appendix Search Strategy by Database, we report an example of the original search string for each host/search platform (*ERIC* for EBSCO, *Social Science Citation Index* for ISI Web of Science, *Medline* for OVID, and *ProQuest dissertation & theses A&I* for ProQuest). We used the same search string, with minor modifications, for each platform.

Note that the searches contained terms relating to primary school, since the search contributed to a review about this younger age group (kindergarten to Grade 6, see Dietrichson, Bøg, Eiberg, Filges, & Klint Jørgensen, 2016, for the protocol). There is overlap in the literature among the age groups, and in order to rationalise and accelerate the screening process, we decided upon performing one extensive search.

#### 5.2.2 Searches on national indices/repositories

- *Australian Education Index* (ProQuest‐host)
- *DIVA* (http://www.diva‐portal.org/smash/search.jsf?dswid=9447)
- *NORA/CRISTIN* (http://nora.openaccess.no/)
- Forskningsdatabasen.dk
- *Theses Canada* (http://www.bac‐lac.gc.ca/eng/services/theses/Pages/theses‐canada.aspx)
- *Cochrane Library* (http://www.cochranelibrary.com/)
- *Social Care Online* (http://www.scie‐socialcareonline.org.uk/)
- *Centre for Reviews and Dissemination Databases* (https://www.crd.york.ac.uk/CRDWeb/)

All indices and repositories were originally searched from 1st of January 1980 to 2nd–11th of March 2016. We updated the searches in July 2018 using identical search strings.

#### 5.2.3 Contact to international experts

We contacted international experts to identify unpublished and ongoing studies. We primarily contacted corresponding authors of the related reviews mentioned in Section 3.4.1,^2^ and authors with many and/or recent included studies. The following authors replied: Douglas Fuchs, Lynn Fuchs, Russell Gersten, Nancy Scammaca, Robert Slavin and Sharon Vaughn.

#### 5.2.4 Citation tracking

In order to identify both published studies and grey literature we utilised citation‐tracking/snowballing strategies. Our primary strategy was to citation‐track related systematic‐reviews and meta‐analyses: 1,446 references from 23 existing reviews were screened in order to find further relevant grey and published studies (see Section 3.4.1 and the list in the appendix Grey Literature and Searches on Other Resources). The review team also checked reference lists of included primary studies for new leads.

#### 5.2.5 Trial registries

Our protocol stated that we should search two trial registries: The Institute for Education Sciences’ (IES) Registry of Randomized Controlled Trials (http://ies.ed.gov/ncee/wwc/references/registries/index.aspx), and American Economic Association's RCT Registry (https://www.socialscienceregistry.org). We were however unable to search the IES registry as it was not available (last tried in July 23, 2018). We have asked IES about availability, but have to date not received a reply. We updated the search of American Economic Association's RCT Registry in July 23, 2018.

#### 5.2.6 Hand searches

The following international journals were hand searched for relevant studies:

- American Educational Research Journal
- Journal of Educational Research
- Journal of Educational Psychology
- Journal of Learning Disabilities
- Journal of Research on Educational Effectiveness
- Journal of Education for Students Placed at Risk

The search was performed on editions from 2015 to July 2018 (i.e., including an updated search) of the journals mentioned, in order to capture relevant studies recently published and therefore not found in the systematic search.

#### 5.2.7 Grey literature

Different strategies were utilised in order to identify relevant grey literature. A wide range of searches were performed on the below institutional and governmental sites, academic clearinghouses and repositories for relevant academic theses, reports and conference/working papers:

- What Works Clearinghouse—U.S. Department of Education (whatworks.ed.gov)
- Danish Clearinghouse for Education Research (edu.au.dk/clearinghouse).
- European Educational Research Association (http://www.eera‐ecer.de/).
- American Educational Research Association (http://www.aera.net/).
- German Educational Research Association (http://www.dgfe.de/en/aktuelles.html).
- NBER working paper series (http://nber.org/).
- Best Evidence Encyclopedia (http://www.bestevidence.org/).
- OpenGrey (http://www.opengrey.eu/).
- Google and Google Scholar (https://scholar.google.dk/).^3^

### 5.3 Data collection and analysis

#### 5.3.1 Selection of studies

Under the supervision of the review authors, at least two review team assistants independently screened titles and abstracts to exclude studies that were clearly irrelevant. Any disagreement of eligibility was resolved by the review authors. Studies considered eligible were retrieved in full text. The full texts were then screened independently by two review team assistants under the supervision of the review authors. Any disagreement of eligibility was resolved by the review authors. The study inclusion criteria were piloted by the review authors with all members of the review team.

#### 5.3.2 Data extraction and management

Two members of the review team independently coded and extracted data from included studies. A coding sheet was piloted on several studies and revised. Any disagreements were resolved by discussion, and it was possible to reach consensus in all cases. Data was extracted on the characteristics of participants, characteristics of the intervention and control/comparison conditions, research design, sample size, outcomes and results. Extracted data was stored electronically, and we used EPPI Reviewer 4, Microsoft Excel, R and Stata as the primary software tools.

#### 5.3.3 Assessment of risk of bias in included studies

We assessed the risk of bias of effect estimates using a model developed by Prof. Barnaby Reeves in association with the Cochrane Non‐Randomised Studies Methods Group. This model is an extension of the Cochrane Collaboration's risk of bias tool and covers risk of bias in nonrandomised studies that have a well‐defined control group. The extended model is organised and follows the same steps as the risk of bias model according to the 2008‐version of the Cochrane Hand book, chapter 8 (Higgins & Green, 2008). The extension to the model is explained in the three following points:

(1) The extended model specifically incorporates a formalised and structured approach for the assessment of selection bias in nonrandomised studies by adding an explicit item about confounding. This is based on a list of confounders considered to be important and defined in the protocol for the review. The assessment of confounding is made using a worksheet where, for each confounder, it is marked whether the confounder was considered by the researchers, the precision with which it was measured, the imbalance between groups, and the care with which adjustment was carried out. This assessment informed the final risk of bias score for confounding.
(2) Another feature of effect estimates in nonrandomised studies that make them at high risk of bias is that they need not have a protocol in advance of starting the recruitment process (this is however also true for a very large majority of RCTs in education). The item concerning selective reporting therefore also requires assessment of the extent to which analyses (and potentially, other choices) could have been manipulated to bias the findings reported, for example, choice of method of model fitting, potential confounders considered/included. In addition, the model includes two separate yes/no items asking reviewers whether they think the researchers had a prespecified protocol and analysis plan.
(3) Finally, the risk of bias assessment is refined, making it possible to discriminate between effect estimates with varying degrees of risk. This refinement is achieved with the addition of a 5‐point scale for certain items (see the next section for details).

The refined assessment is pertinent when thinking of data synthesis as it operationalizes the identification of studies (especially in relation to nonrandomised studies) with a very high risk of bias. The refinement increases transparency in assessment judgements and provides justification for not including a study with a very high risk of bias in the meta‐analysis.

##### Risk of bias judgement items

The risk of bias model used in this review is based on nine items (see Appendix Risk of bias tool for a fuller description). The nine items refer to: Sequence generation, allocation concealment, blinding, incomplete outcome data, selective outcome reporting, other potential threats to validity, a priori protocol, a priori analysis plan and confounders (for nonrandomised studies). As all but the latter follow standard procedures described in the Cochrane Handbook (Higgins & Green, 2011), we focus on the confounding item below.

##### Confounding

An important part of the risk of bias assessment of effect estimates in nonrandomised studies is how studies deal with confounding factors. Selection bias is understood as systematic baseline differences between groups and can therefore compromise comparability between groups. Baseline differences can be observable (e.g., age and gender) and unobservable to the researcher (e.g., motivation). Included studies use for example matching and statistical controls to mitigate selection bias or demonstrate evidence of preintervention equivalence on key risk variables and participant characteristics. In each study, we assessed whether the observable confounding factors of age and grade level, performance at baseline, gender and socioeconomic background had been considered, and how each study dealt with unobservables.

There is no single nonrandomised study design that always deals adequately with the selection problem. Different designs represent different approaches to dealing with selection problems under different assumptions and require different types of data. There can be particularly great variations in how different designs deal with selection on unobservables. For example, differences in preintervention test score levels do not have to be a major problem in a difference‐in‐differences design, where the main identifying assumption is that the trends of the outcome variable in the intervention and control group would not have differed, had the intervention not occurred. Similar differences in levels would, in general, be more problematic in a matching design as they indicate that the matching technique has not been able to balance the sample even on observable variables. For this reason, we did not specify thresholds in terms of preintervention differences (in say, effect sizes) for when a study has too high risk of bias on confounding. Each QES was assessed in terms of the risk that the effect of the intervention was confounded with observed and unobserved variables.

##### Importance of prespecified confounding factors

The motivation for focusing on age and grade level, performance at baseline, gender and socioeconomic background is given below.

Development of cognitive functions relating to school performance and learning are age dependent. Furthermore, systematic differences in performance level often refer to systematic differences in preconditions for further development and learning of both cognitive and social character (Piaget, 2001; Vygotsky, 1978). Therefore, to be sure that an effect estimate was a result from a comparison of groups with no systematic baseline differences it was important to control for the students’ grade level (or age).

Performance at baseline is generally a very strong predictor of posttest scores (e.g., Hedges & Hedberg, 2007), and controlling for this confounder was therefore highly important.

With respect to gender it is well‐known that gender differences exist in school performance (e.g., Holmlund & Sund, 2005). In terms of our primary outcome measures, girls tend to outperform boys with respect to reading and boys tend outperform girls with respect to mathematics (Stoet & Geary, 2013), although part of the literature finds that these gender differences vanish over time (Hyde & Linn, 1988; Hyde, Fennema, & Lamon, 1990). As there is no consensus around the disappearance of gender differences, we found it important to include this potential confounder.

Students from more advantaged socioeconomic backgrounds on average begin school better prepared to learn (e.g., Fryer & Levitt, 2013). As outlined in the background section, students with socioeconomically disadvantaged backgrounds have lower test scores on international tests (OECD, 2010, 2013). Therefore, the accuracy of the estimated effects of an intervention may depend on how well socioeconomic background is controlled for. Socioeconomic background factors were for example parents’ educational level, family income and ethnic/cultural background.

##### Bias assessment in practice

At least two review authors independently assessed the risk of bias for each included study. Disagreements were resolved by discussion, and it was possible to reach a consensus in all cases. We reported the risk of bias assessment in risk of bias tables for each included study (see Appendix Risk of bias tool).

In accordance with Cochrane and Campbell methods we did not aggregate the 5‐point scale across items. Effect sizes given a rating of 5 on any item should be interpreted as being more likely to mislead than inform and were not included in the meta‐analysis (the items with a three‐point scale did not warrant exclusion). Although we only gave 5 points for an item to denote a very high risk of bias, we excluded a large number of effect sizes. If an effect size received a rating of 5 on any item (from both reviewers), we did not continue the assessment because, as per our protocol, these effect sizes would not be included in any analysis. We discuss the risk of bias assessment, including the most common reasons for excluding an effect size, in the Risk of Bias in Included Studies section. For studies with a lower than 5‐point rating, we used the ratings of the major items in sensitivity analyses.

A note is warranted for how we assessed some items in practice. Allocation concealment was assessed as a type of second order bias in RCTs. If there was doubt or uncertainty about how the random sequence was generated, this automatically carried over to the allocation concealment rating, which was also rated “Unclear”. Similarly, if the sequence generation rating was “High”, as, for example, in a QES, then the allocation concealment rating was also “High”. RCTs rated “Low” on sequence generation could get a “High” rating on allocation concealment if the sequence was not concealed from those involved in the enrolment and assignment of participants. However, if the randomisation was not done sequentially, this should not present a problem, and allocation concealment in nonsequentially randomised RCTs were rated “Low”, given that the rating on sequence generation was also “Low”.

Blinding is in practice always a problem in the interventions we included. No included study was double‐blind for example, a standard that is very difficult to attain in an educational field trial. Furthermore, blinding was, potentially because it is difficult to attain, not extensively discussed in many studies. For these reasons, we did not exclude any effect size due to insufficient blinding and rather than rating all studies that did not explicitly discuss blinding as “Unclear”, we sought to assess how likely it was that a particular group of participants was blind to treatment status. We used the following groups of participants: students in intervention and control groups, teachers, parents and testers. We assessed the blinding‐item by the following standard: If all participant groups were likely to be aware of treatment status or there was no indication of any group being blinded, we gave the study a rating of 4. If at least one group was likely blind to treatment status, it got a 3, and then we lowered the rating when more groups were blinded.

There were moreover very few studies that reported having a protocol or a preanalysis plan. This lack of prespecified outcome measures made it difficult to assess selective outcome reporting bias. However, a few studies lacked information regarding all outcomes described in, for example, the methods section of the study. To separate these effect sizes from the ones that did not contain information about a protocol or an analysis plan, we rated the latter ones with 1 (i.e., there was no evidence of selective outcome reporting). This rating should therefore not necessarily be considered as representing a low risk of bias.

#### 5.3.4 Measures of the intervention effect

The analysis of effect sizes involved comparing an intervention to a control condition. We conducted separate analyses for short‐ and long(er)‐term outcomes, although this latter analysis was highly constrained due to the lack of long‐term outcomes in the included studies. The analysis plan laid out below applies to both types of outcomes.

##### Effect sizes using continuous data

For continuous data, standardised mean differences (SMDs) were calculated when means and standard deviations were available. All studies included in the data synthesis provided information so that student level effect sizes could be calculated. We used Hedges' *g* to estimate SMDs where scales have been used to measure the same outcomes in different ways. Hedges' *g* and its standard error were calculated as (Lipsey & Wilson, 2001, pp. 47–49):

(1) $$g=\left(1-\frac{3}{4N-9}\right)\times \left(\frac{{\bar{X}}_{1}-{\bar{X}}_{2}}{s_{p}}\right),$$

(2) $$SE_{g}=\sqrt{\frac{N}{n_{1}n_{2}}+\frac{g^{2}}{2N}},$$

where $N=n_{1}+n_{2}$ is the total sample size, $\overset{®}{X}$ is a postintervention mean in each group and $s_{p}$ is the pooled standard deviation defined as

(3) $$s_{p}=\sqrt{\frac{\left(n_{1}-1\right)s_{1}^{2}+\left(n_{2}-1\right)s_{2}^{2}}{\left(n_{1}-1\right)+\left(n_{2}-1\right)}},$$

here, $s_{1}$ and $s_{2}$ denotes the standard deviation of the intervention and control group. We used covariate adjusted means, and the unadjusted posttest standard deviation whenever available. We decided to use the postintervention standard deviation, as more studies included this information than the preintervention standard deviation. In the few cases where instead the postintervention standard deviation was missing, we used the preintervention standard deviation.

Some studies reported only raw means. In these cases, we coded two effect sizes: one based on the unadjusted postintervention mean difference (i.e., exactly as in Equation (1) above), and one where we subtracted the preintervention mean from the postintervention mean for both the intervention and control group. That is,

(4) $$g=\left(1-\frac{3}{4N-9}\right)\times \left(\frac{\left({\bar{X}}_{12}{-\bar{X}}_{11})-({\bar{X}}_{22}{-\bar{X}}_{21}\right)}{s_{p}}\right),$$

where ${\overset{®}{X}}_{12}$ is the postintervention mean in the intervention group, ${\overset{®}{X}}_{11}$ is the preintervention mean in the intervention group, and ${\overset{®}{X}}_{22}$ and ${\overset{®}{X}}_{21}$ are the corresponding means in the control group. We used the effect sizes based on Equation (1), which was available in more studies, as our baseline effect size and used the second type in a sensitivity test (reported in the Results of Sensitivity Analyses section).

Six studies reported an effect size where the mean difference was standardised using the control group's standard deviation (i.e., Glass's delta), and two studies used, for example, the school‐, district‐, or nation‐wide standard deviation, but did not include information about the respective standard deviation for intervention and control group. We included these effect sizes and tested the sensitivity to their inclusion in the Results of Sensitivity Analyses section.

Our protocol stated that we would use intention‐to‐treat (ITT) estimates of the mean difference whenever possible. However, very few studies reported explicit ITTs, and the overwhelming majority only reported results for the students that actually received the intervention, rather than all for which the intervention was intended (often because they lacked outcome data for students that left the study). We therefore believe that the estimates are closer to treatment‐on‐the‐treated (TOT) effects and used TOT estimates in the few cases where both ITTs and TOTs were available. We tested the sensitivity to this choice in the Results of Sensitivity Analyses section.

##### Effect sizes using discrete data

Only two studies exclusively reported discrete outcome measures. We transformed the outcomes into SMDs using the methods described in Sánchez‐Meca et al. (2003) and included it in the analyses together with studies reporting continuous outcomes.

#### 5.3.5 Unit of analysis issues

Errors in statistical analysis can occur when the unit of allocation differs from the unit of analysis. In cluster randomised trials, participants are randomised to intervention and control groups in clusters, as when participants are randomised by treatment locality or school. QES may also include clustered assignment of treatment. Effect sizes and standard errors from such studies may be biased if the unit‐of‐analysis is the individual and an appropriate cluster adjustment is not used (Higgins & Green, 2011).

If the information was available, we adjusted the effect sizes using the methods suggested by Hedges (2007) and information about the intracluster correlation coefficient (ICC), realised cluster sizes, and/or estimates of the within and between variances of clusters. If this information was not available, we instead used estimates from the literature of the ICC and assumed equal cluster sizes. We calculated our ICCs by taking the average over the ICCs reported for low achievement schools in Grades 7–12 in column 1 of tables 6 (mathematics) or 7 (reading) in Hedges and Hedberg (2007). To calculate an average cluster size, we divided the total sample size in a study by the number of clusters (typically the number of classrooms or schools).

Most studies lacked information about one of more of the ICC, cluster sizes, and within and between variances. As the procedure therefore entailed considerable uncertainty about how correct the adjustment was, we used the effect sizes adjusted with estimates from the previous literature only in the sensitivity analysis. Effect sizes that could be individually adjusted using information from the study (or directly provided adjusted effect sizes) were adjusted (used) also in the main analysis.

##### Multiple intervention groups and multiple interventions per individual

Studies with multiple intervention groups with different individuals, and studies using multiple tests for the same intervention groups, were included in the review. To avoid problems with dependence between effect sizes, we primarily used the robust variance estimation (RVE) methods developed by Hedges et al. (2010). We used the results in Tanner‐Smith and Tipton (2014) and Tipton (2015) to evaluate if there were enough studies for this method to consistently estimate the standard errors. See Section 5.3.9 below for more details about the data synthesis. We did not include any study where the participants got multiple interventions per individual.

##### Multiple studies using the same sample of data

We reviewed studies of interventions given to (partly) the same groups of students, but included one estimate of the effect from each sample of data in the meta‐analysis to avoid overlapping samples. We chose the estimate from the intervention that had lowest risk of bias or contained the most information. See Appendix Studies with Overlapping Samples or Lacking Information, Table A3, for a summary description of included studies that we did not include in the meta‐analyses for this reason.

##### Multiple time points

Few studies reported outcomes measured at other time points than close to the end of the intervention, and none longer than 12 months after the end of intervention. As per our protocol we divided the analysis into:

- Short‐run effects (<3 months after the end of intervention).
- Medium‐ to long‐run effects (3 months or more after the end of intervention).

The examination of heterogeneity and moderator analysis focused on the short‐run effects. The number of studies reporting longer‐run outcomes was not large enough to permit a similar analysis. Some studies did not contain exact information about measurement timing. Unless there was information in the study that indicated that the measurement was not done within 3 months after the end of an intervention, we interpreted these as being short‐run effects.

#### 5.3.6 Dealing with missing data

Missing data and attrition rates in the individual studies were assessed using the risk of bias tool. Studies had to permit a calculation of a numeric effect size for the outcomes to be eligible for inclusion in the meta‐analysis. Where studies had missing summary data, such as missing standard deviations, we derived effect sizes where possible from, for example, *F* ratios, *t* values, *χ* ^2^ values and correlation coefficients using the methods suggested by Lipsey and Wilson (2001). If these statistics were also missing, we asked the study investigators if they could provide us with the information. We were unable to retrieve information from 13 studies. These studies were included in the review but excluded from the meta‐analysis (see Appendix Studies with Overlapping Samples or Lacking Information, Table A3, for a summary description).

Many studies did not provide data about all moderators. We focused on moderators that were relevant for all types of studies and used multiple imputation methods (see, e.g., Rubin, 1996, and Pigott, 2009) in a sensitivity analysis to test if our results were sensitive to omitting moderators. We used the Stata command *mi impute* with sequential imputation using chained equations to generate values for missing observations. All variables without missing observations were used in the estimation to impute values for variables with missing observations. We used 20 imputed data sets.

#### 5.3.7 Assessment of heterogeneity

Heterogeneity was assessed with *χ* ^2^ (*Q*) test, and the *I* ^2^, and *τ* ^2^ statistics (Higgins, Thompson, Deeks, & Altman, 2003).

#### 5.3.8 Assessment of reporting biases

Reporting bias refers to both publication bias and selective reporting of outcome data and results. Bias from selective reporting of outcome data and results is one of the main items in the risk of bias tool.

To examine possible publication bias, we used funnel plots, Egger's test, and tested whether studies published in scientific journals had different effect sizes compared to other studies.

#### 5.3.9 Data synthesis

The overall data synthesis in this review was conducted when effect sizes were available. Effect sizes coded with a very high risk of bias (score of 5 on any item judged on a 5‐point scale) were not included in the data synthesis. The analysis was conducted in the following steps. We described summary and descriptive statistics of the intervention‐level characteristics, and the risk of bias assessment. We also included a correlation matrix with all moderators. We performed analyses divided by measurement timing first (end‐of‐intervention or follow‐up). To be able to simultaneously include all effect sizes from each study and avoid problems with dependence between effect sizes we used the RVE procedure in the Stata command *robumeta* for estimation and to calculate robust standard errors (Hedges et al., 2010). We used the random effects model weighting scheme option, as it seemed most likely that the effects of the included interventions were not the same across studies, but follow a distribution. A fixed effect model would therefore be less appropriate in our case (e.g., Borenstein, Hedges, Higgins, & Rothstein, 2009).

The RVE procedure requires an initial estimate, $\hat{\rho }$, of the correlation between tests within the same study. We used $\hat{\rho }$ = 0.8 (as, e.g., Hedges et al., 2010; Wilson et al., 2011; and Dietrichson et al., 2017). We report 95% confidence intervals throughout the analysis and used the small sample adjusted standard errors and degrees of freedom suggested by Tipton (2015). The study level average effect sizes were also displayed in a forest plot. We used the Stata command *metan* to create the forest plot.

#### 5.3.10 Subgroup analysis and investigation of heterogeneity

The analysis revealed substantial heterogeneity in effect sizes and one of the main objectives in the review was to assess the comparative effectiveness of intervention components. We therefore performed subgroup and moderator analysis to attempt to identify the characteristics of study methods, interventions, and participant characteristics that were associated with smaller and larger effects. We used meta‐regressions to reduce the risk of misleading results due to correlated independent variables, and again used the RVE procedure in *robumeta*. We reported 95% confidence intervals for regression coefficients.

Most included moderators were coded as indicator variables (most variables are natural indicators, e.g., whether the study design was an RCT or not). Continuous variables were mean‐centred to facilitate interpretation. Our protocol specified the following types of moderators:

- Subject
- Study design
- Effect size measurement
- Participant characteristics
- Treatment modality
- Dosage
- Implementation quality

Below we describe the moderators we used. However, the number of included studies and effect sizes were not large enough to include all coded moderators in one meta‐regression (see the Appendix and the Coding Scheme Section for a description of all coded variables). In line with the objective of the review and our protocol, we therefore focused the analysis of subgroups and heterogeneity on instructional methods and content domains. These are substantive features of interventions that for example teachers and principals can affect, in contrast to other moderators (e.g., participant characteristics may be more difficult to affect for a school). They were also more often (quasi‐)experimentally manipulated in studies than other moderators in our sample. They may therefore be less likely to be confounded with other, omitted moderators.

To further reduce the number of moderators, we first excluded moderators with very low variation (i.e., where nearly all observations have the same value) or where information was missing from studies. We also excluded moderators that were not relevant for all intervention types (for example, there is no number of sessions in an intervention that provide students with incentives to read a certain number of books). For some analyses we excluded highly correlated variables.

We have characterised the included interventions by their components using two general categories of treatment modalities: *instructional method* and *content domain*. As described in our protocol, the components were not fully prespecified, but developed and adapted during the coding process. We used previous reviews and author‐reported classifications in included studies as a starting point, and an iterative process to construct component categories. Below, we describe the coded components by treatment modality, and how these components were used to develop the moderators we included in the meta‐regressions. Note that interventions often contained more than one component and they were coded in all component categories they contained. The categories below are therefore not mutually exclusive.

##### Instructional method

The instructional method‐categories describe the method of delivering the intervention; that is, the contrast between the intervention group and the control group in terms of how instruction was given. Many interventions contained more than one instructional component. In these cases, we have coded the intervention in all categories.

###### Coaching of personnel

Interventions in this category included programmes that provided teachers or other school personnel with coaches. Note that this component did not include professional development interventions that seek to develop more general teaching or management skills, as such interventions were never targeted to at‐risk students in our sample. The coaching in this category was mainly connected to the implementation of a specific reading or mathematics programme.

###### Computer‐assisted instruction

This category indicated whether the intervention, or parts of the intervention, was given with the help of computers, tablets or similar devices.

###### Incentives

Incentive programmes intended to increase the academic performance of students were included in this category. The incentives were not always monetary, nonfinancial incentives were also included. Examples included interventions where the incentive component was the only component, for example, students were paid to perform on interim assessments or for grades in 5 week courses or to improve general achievement, but several interventions combined incentives with other components.

###### Peer‐assisted instruction

We separated between adult‐led instruction and peer‐assisted instruction. Peers were defined as students in Grades 7–12. Interventions such as cross‐age tutoring where 9th graders tutor 7th graders were thus coded as peer‐assisted instruction (for both tutors and tutees if results were reported for both groups). If on the other hand college students acted as tutors to high school students, the intervention was coded as adult‐led small group instruction (see below for description). We coded the exact group size, if available in the studies, but to keep the number of moderators down, we created one moderator for the peer‐assisted instruction category. Most studies used small groups like pairs.

###### Progress monitoring

This category included interventions that added a specific progress monitoring component, where teachers received detailed information about the students’ development. Note that for example small group interventions of all kinds are also likely to contain increased feedback and, in a sense, increased (informal) progress monitoring. These interventions were not automatically coded in this category. Interventions had to add an extra component of progress monitoring, such as using curriculum‐based measurements during the intervention, to be coded here.

###### Small group instruction

As mentioned, we differentiated between adult‐led instruction and peer‐assisted instruction, and the small group instruction category included adult‐led instruction. In some interventions, instruction was given in class, and not divided into smaller groups (this was, or was very likely to be, the same type of instruction given to the control group, so we did not create a moderator for this group size). We coded the exact group size whenever available but quite a few studies did not provide exact information about group size (but reported for example a range). We coded interventions without specific information about group size in the most likely category, given other information in the study or based the coding on information from other studies of the same intervention (e.g., there are several studies of READ 180). Because of the missing data, and to keep the number of moderators down, we decided on one moderator contrasting adult‐led instruction in groups of five students or less with other group sizes. A smaller group usually meant that the information was included, and more exact. Some interventions vary the group size during the intervention, for example, used both 1:1 tutoring and larger groupings. If an intervention contained at least one component that involved instruction in groups of five or smaller, it was coded in this category.

Lastly, we created a category called “other method” that included interventions that were not coded in any of the above categories. There were three types of interventions making up this category: Some interventions provided instruction in groups smaller than whole class but larger five students, and they were thus not coded in the small group instruction category. In a few cases, there was no difference in how the intervention and control group was instructed, because it was either only the content that differed or the intervention group was just provided extra instruction time. The latter case was difficult to assess systematically for all interventions, as many studies did not provide information about how much instruction time the intervention and the control group got in a certain area. Therefore, we did not create a separate category for extra instruction time.

##### Content domain

The content domain describes the area targeted by the intervention and the material taught. We divided these components into reading, mathematics and other areas. For reading, we used the following categories:

###### Comprehension

Reading comprehension interventions focused on the understanding and learning from text. Reading comprehension is described by the National Reading Panel (2000) as an active process where interaction between the text, the author, and the reader results in the understanding or meaning making of the text. The RAND Reading Group defines comprehension as “the process of simultaneously extracting and constructing meaning through interaction and involvement with written language” (RAND Reading Study Group, 2002, p. 720).

###### Decoding

Decoding interventions focused on the translation of print to speech. This category included word identification, word study, and word reading interventions. Included interventions in this category also taught phonological awareness, phonemic awareness, and phonics. Such skills are often thought to be precursors to efficient decoding.

###### Fluency

Fluency is defined as the ability to read orally with speed, accuracy, and proper expression (The National Reading Panel, 2000). Interventions in this category aimed for example to improve the ability to read in a “smooth and effortless” manner (Allinder, Dunse, Brunken, & Obermiller‐Krolikowski, 2001).

###### Spelling and writing

Some interventions included spelling and writing training, which, while not strictly a reading skill, were related enough (and was also tested with standardised reading tests). This subdomain was not the sole target of any intervention.

###### Vocabulary

This category included interventions focused on increasing the number of words a student knows.

In addition to these single domains, we also coded a multiple reading domain category. Beside interventions focused on more than two of the above subdomains, this category included interventions that were described as focusing on reading in general, but did not explicitly mention any subdomains.

For math interventions, we found interventions targeting the following categories:

###### Algebra/prealgebra

Algebra and prealgebra interventions focused on, for example, basics of equations, and graphs.

###### Fractions

Fraction interventions taught the concept of fractions and how to manipulate them.

###### Geometry

Geometry refers to the study of, for example, shapes, sizes and positions. It was never the sole subdomain in any intervention but was always combined with one or more domains.

###### Operations

This category included for example training in addition, subtraction, multiplication, and more generally, computational skills.

###### Probability

Probability, which included, for example, statistics, was not the single domain in any intervention but was always combined with at least one more domain.

As for reading, we coded a multiple mathematics domains category, which included interventions explicitly covering more than one domain as well as more general math interventions. We found fewer math interventions overall, and there were few interventions studying a specific subdomain (ten for algebra/prealgebra, four for fractions, three for geometry, four for operations, and two for probability). Several interventions furthermore targeted both reading and math. For these reasons, we used a single subgroup/moderator for effect sizes based on mathematics tests in the analysis.

Finally, we coded three categories to characterise interventions targeting other areas instead of, or together with (subdomains of) reading and mathematics.

###### Meta‐cognitive strategies

Meta‐cognitive strategies and self‐regulation interventions aimed to help students think about their own learning more explicitly, and develop strategies for such learning, including managing one's own motivation towards and engagement with learning. The intention was often to give pupils a repertoire of strategies to choose from during learning activities, including study skills or learning how to learn. In comparison to the next domain, social‐emotional skills, the skills trained in this category were more focused on the student, and less on the relations to other students or school staff.

###### Social‐emotional skills

Interventions in this category focused on improving educational achievement through, for example, improving social skills, and mitigating problematic behaviour. They thus had a more relational focus compared to meta‐cognitive and self‐regulation interventions.

###### General academic skills

This category included studies without a particular content domain or a more general academic focus than just reading and math. As the authors studying such interventions still included a standardised test in reading or math, we interpreted the intervention in these cases as being expected to improve these subjects.

The latter two categories were relatively rare in our sample, social‐emotional skills were included in eight interventions and general academic skills in twelve interventions. We therefore combined these three categories into one moderator, called other domains, in the analysis.

##### Other intervention characteristics

When coding other intervention characteristics, we used information about the intervention group, if available. If information was not available on the intervention group level, we used joint intervention and control group information, and then higher levels (grades, schools). However, information on levels higher than schools, such as school districts, was treated as missing. Some studies included only information about intervention characteristics given in a range. In these cases, we used the mid‐point of that range. Below, we first describe the moderators used in the analysis in more detail.

###### Study context

We coded the country where the information was performed. When information was missing, we made an assessment based on, for example, where the authors were based at the time of the study, and on the mentioning of country‐specific reforms like No Child Left Behind in the United States. We reported the number of participants, schools and districts involved in the study.

###### Study design characteristics

We coded an indicator variable equal to one for QES. The reference category for the latter is RCTs, we found no QRCTs. We coded whether implementation was monitored in some way, whether problems were mentioned, and if so, what type of problems that was mentioned. In the analyses, we used an indicator equal to one if implementation problems were explicitly mentioned. Some problems mentioned by more than one study were low attendance, that implementers had low quality of implementation or low motivation, and that some in the control group might have received (some of) the intervention. But there were too few repetitions of problems for it to be possible for us to use this information in the analysis.

###### Effect size measurement

Effect sizes have been calculated on the basis of different types of tests, which may cause heterogeneity. We therefore coded the content domains of the tests. We found only one subdomain in math (algebra), most tests covered several domains. There was on the other hand a large number of tests covering subdomains of reading (e.g., comprehension, fluency, phonic and phonemic decoding, phonological and phonemic awareness, language mechanics and expression, spelling and vocabulary). Most of these subdomains were only used in a few instances though. In the meta‐regressions, we therefore included one moderator indicating whether a test was general, in the sense that it covered two or more subdomains. We furthermore coded two moderators that relate to the calculation of effect sizes, which we used for sensitivity analyses. Glass's delta indicated whether the SMD was standardised with the control group's standard deviation. This was the case in some studies that did not include information about the pooled standard deviation, and rather than excluding them, we tested whether our results are sensitive to their inclusion. We also used an indicator equal to one if the SMD had been standardised with a standard deviation from a super‐population (e.g., grade, district or state) instead of the intervention and control group, or if the number of included schools, districts or regions was larger than the intervention median (3, 1 and 1 respectively). Both standardisation with a super‐population and including more schools, districts, and regions may imply that the variance in the sample could be larger and effect sizes mechanically smaller, as the study included a possibly more varied group than other studies (see, e.g., Lipsey et al., 2012). We chose to make one variable for these two related problems as there were few studies that standardised with a super‐population.

###### Participant and sample characteristics

We measured the gender distribution by the share of girls. We coded both age and grade (minimum, maximum and mean for both) but the information about age, as well as minimum and maximum for both variables, were missing for far more interventions. We therefore focused on the mean grade and used the information about age in the one study missing grade information to estimate a mean grade. Outcomes were normally measured in the same grade that the intervention was performed, but in some cases interventions spanned one or more grades. The grade variable we used refers to the grade in which the outcome measurement was performed. We coded the share of minority students (defined as not being part of the majority population group in a country) and the share of students from low income families, which was almost always measured as the share of students with free‐ or reduced price meals.

###### Dosage

We coded three variables related to the dosage of an intervention: Duration is the length of the intervention measured in weeks. We used 40 weeks for a school year, and consequently 20 weeks for a semester. The frequency of an intervention was measured by the total number of sessions, and the intensity by the total number of intervention hours per week. For these dosage‐variables we coded both intended and received dosage. However, many studies lacked information on either the intended or received dosage. We used received dosage as a starting point, and added intended in the cases were the received number was missing.

###### Implementers

We used information about who implemented the instruction to develop two variables: *School staff* and *Researcher*. The first is an indicator equal to one if staff at school (e.g., teachers, special education teachers, coaches, assistants) served as instructors, and zero otherwise. The second is equal to one if researchers or research‐affiliated staff (e.g., project managers) handled the instruction. Other types of instructors, such as college students, belong to the reference category for both variables. There were too few interventions where categories other than researchers or school staff were responsible for the implementing the instruction to allow a meaningful construction of further variables.

In the moderator analysis, we focused on moderators that were relevant for all types of interventions, varied enough between interventions, and did not have any missing information. The included variables in the main analysis were the indicator for QES, two continuous variables measuring the grade and duration of interventions, and the indicator for implementation problems. The two continuous variables were mean‐centred.

Our protocol mentioned and we coded information from several moderators, which we in the end could not use. One reason was lack of information. For example, no study provided information about parental occupation and only three about parental education. The share of students speaking the majority language as a second language was included in 18 studies. For other moderators, there was very little variation: almost all were performed only in school, and few had target groups that were not defined in terms of having academic difficulties. That is, few studies defined the target group purely in terms of for instance the students’ SES. It was furthermore difficult to develop a moderator measuring the severity of academic difficulties, mainly because different tests were used to measure and define difficulties. Eleven studies specifically targeted students with learning disabilities but other studies also included some learning disabled studies and many more did not include information about the share of learning disabled students. Due to the small number of studies and the unclear contrast, we refrained from using learning disability as a moderator. We coded whether implementers received training before the intervention, but if this was not the case, it almost always meant that it was a researcher or someone affiliated with the research team who performed the intervention. The information is therefore overlapping with the variables measuring who implemented the intervention.

All effect sizes in our analysis were derived from treatment‐control studies. We coded whether the control group was a waitlist design, but it was often not explicitly mentioned whether the control group got the intervention after the intervention group. The instruction given to the control group differed between interventions. Control group instruction was nearly always some form of TAU, but it was difficult to separate different TAUs from each other as the information was not detailed enough. We were therefore unable to create moderators based on the control group instruction.

#### 5.3.11 Sensitivity analysis

We performed the following sensitivity analyses:

##### Effect size measurement

We tested sensitivity to measurement of effect sizes in the following way: One study, Cook et al. (2014), reported ITT and TOT estimates. We believed that their TOT estimate was closer to the estimates in other studies, which typically only reported effects for the actually participating students. We therefore tested whether using the Cook et al.'s ITT estimate changed our results. As described in the Effect Sizes Using Continuous Data section, we used preintervention means to calculate a second type of effect size based on mean differences in pre‐post means in studies where we could only use the raw postintervention means to calculate effect sizes. If the results differed from our baseline, this would be an indication that there are systematic preintervention differences between treatment and control group in studies that do not include preintervention tests as covariates to adjust their estimates. In turn, such systematic differences could bias our results. We also tested sensitivity to the inclusion of studies that reported SMDs standardised with a super‐population or only provided information about SMDs standardised with the control group standard deviation (i.e., Glass's delta).

##### Outliers

We examined the distributions of effect sizes for the presence of outliers and the sensitivity of our main results by methods suggested by Lipsey and Wilson (2001): trimming the distribution by dropping the outliers and by Winsorizing the outliers to the nearest nonoutlier value.

##### Clustered assignment of treatment

We tested sensitivity to clustered assignment of treatment by the methods described in Unit of Analysis Issues section.

##### Missing values

We used multiple imputation to account for missing values in some moderators (share of girls, share of low SES and share of minority students), as described in the Dealing with Missing Data section.

##### Risk of bias

We used the items with numerical ratings from the risk‐of‐bias assessment to examine if methodological quality was associated with effect sizes. We separated RCTs and QES in one analysis, as the confounding item is only relevant for QES. The items with nonnumerical ratings are not relevant for all types of studies, and there was also low variation in the ratings. We re‐coded the items to indicator variables because the items are categorical variables and to avoid having too many moderators. For blinding, incomplete outcome reporting, other bias, and confounding (just used for QES), we contrasted effect sizes given a rating of 4 to those given lower ratings. For the selective outcome reporting item, we contrasted those rated 1 with those given higher ratings.

##### Publication bias

Lastly, we examined publication bias using funnel plots, by performing Egger's test (Egger, Smith, Schneider, & Minder, 1997), and by testing whether unpublished studies have different effect sizes compared to published studies.

### 5.4 Deviations from protocol

The search strategy in our protocol listed “Education Research Complete” among the databases. However, at the time of the search, we no longer had institutional access to this database and did not include it either in our original search or in the updated search. Due to lack of institutional access, we did not search British Education Index, FRANCIS, Dissertation and theses A&I, CBCA Education, and Australian Education Index in the updated search. As mentioned earlier, we could not search the IES’ Registry of Randomized Controlled Trials, as the webpage was shut down at the time of both the original and updated search.

According to our protocol, we should contact international experts to identify studies and give them the list of included studies. We thought it would be advantageous to involve experts earlier in the process, and asked them about relevant studies before our screening process was completed. Therefore, they did not receive a list of included studies.

Our protocol stipulated that we would use ITT estimates whenever available. However, most studies did not report ITT estimates. The estimates reported were in our view closer to TOT estimates, and we therefore decided to use the TOT estimate in the one study reporting both ITT and TOT estimates in our main analysis. As mentioned, we tested the sensitivity to this choice in the Results of Sensitivity Analyses section.

We included studies that compared alternative interventions. We planned to analyse these studies separately from treatment‐control designs and use them where they may shed light on an issue, which could not be fully analysed using the sample of treatment‐control studies. There were however very few studies in which the comparison of interventions involved testing differential effects of components that corresponded to our categories (see online appendix Table A2 for a short description including the contrasts studied). There was no example of differential effects of a pair of components being studied by more than one comparison design. Due to the small number of relevant studies, we refrained from analysing the comparison designs further.

## 6 RESULTS

### 6.1 Description of studies

#### 6.1.1 Results of the search

**Figure 1:**
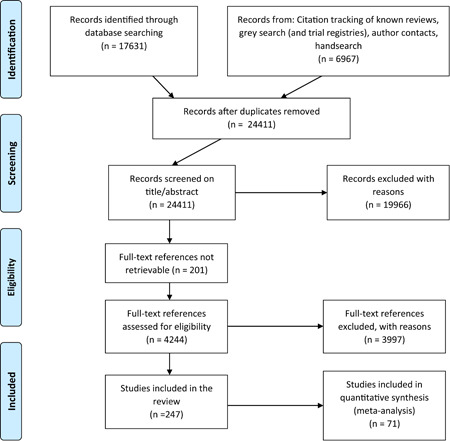
Flowchart of the search and screening process

Figure 1 displays the results of the search process. The total number of potentially relevant records was 24,411 after excluding 187 duplicates (database search: 17,444; grey literature: 3,014; citation tracking: 1,508; author contacts: 576; hand search: 1,024; trial registries and others: 845).

All records were screened based on title and abstract. Of the ones that were not excluded, 201 records were not retrievable in full text. Older reports and dissertations were overrepresented among these records. The remaining 4,244 retrievable records were screened in full text. A large number of studies were not relevant for this review due to the grade of the participating students. Studies that were relevant except for the grade of participating students will be included in a future review covering kindergarten to Grade 6 (see Dietrichson, Bøg, Eiberg, et al., 2016 for the protocol). Two hundred forty seven studies met the inclusion criteria for this review and data were extracted from these studies.

#### 6.1.2 Included studies

The number of included studies was 247. Of these studies, we included 71 in meta‐analyses. We were unable to retrieve sufficient information from 13 studies/study authors to calculate an effect size. Seven studies used samples that overlapped with other included studies and had either a higher risk of bias or contained less information. These studies were not included in the analysis. Some studies contained overlapping samples but included, for example, information about short‐ and long‐run outcomes. These were all included in some meta‐analysis, but never in the same. Thirty‐eight studies were not included in the meta‐analyses due to their study design. These studies used comparison designs that contrasted two alternative interventions. We did not include 118 studies in the meta‐analysis due to the risk of bias assessment. All eligible outcomes from these studies were excluded due to too high risk of bias.

We discuss the results of the risk of bias assessment further in the Risk of Bias in Included Studies section. See also the Risk of Bias Tables in the online appendix for details of the assessment for effect sizes that we included in the meta‐analysis, as well as those that we deemed had too high risk of bias. The comparison designs are described in the appendix, in the Comparison Designs section, Table A2. For more information about studies with overlapping samples or that lacked sufficient information for the calculation of an effect size, see the online appendix, section Studies with Overlapping Samples or Lacking Information, Table A3.

**Figure 2:**
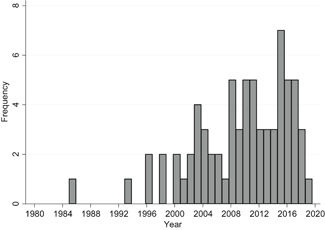
Number of studies included in the meta‐analysis by year

**Figure 3:**
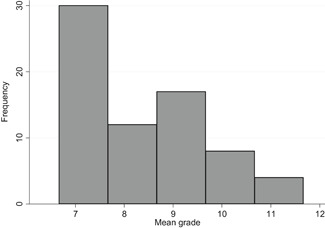
Number of studies included in the meta‐analysis by mean grade

The 71 studies included in the meta‐analyses are described in detail in the Studies Included in the Meta‐Analysis section, Table A1, in the appendix. Figure 2 displays the 71 studies by publication year. Studies with more than one intervention or where different cohorts received the same intervention were treated as one study. There is an increasing trend, only six included studies were published before the year 2000 and about half the studies have been published since 2010. Figure 3 shows the 71 studies by the mean grade of participating students. The bulk of studies included participants in the lower grades (e.g., the mean grade is around seven in 30 studies). There were few studies of interventions in Grades 11 and 12.

Table 1 contains descriptive statistics of the studies included in the meta‐analysis. Many studies contained more than one intervention, the effects of which may have been tested with more than one standardised test from which we calculated the effect sizes. We denoted the number of studies that provided information about a certain characteristic with *k*, the number of interventions with *i*,^4^ and the number of effect sizes with *n*. We used the average over interventions to calculate the mean, standard deviation and range, as most characteristics vary on this level. For example, in a study with two interventions where each intervention and control group take two tests, we averaged by intervention over the two tests. These averages are the basis for means, standard deviations, and ranges in Table 1 (which is why there is an *i* subscript in the table). There were in total 71 studies, 99 interventions and 214 effect sizes.

**Table 1:** Descriptive statistics: Study context, design, outcome assessment, participants and intervention characteristics for studies included in the meta‐analysis

Study characteristics	*k*	*i*	*n*	Mean_*i*_	*SD*_*i*_	Range_*i*_
Study context						
% performed in the United States	71	99	214	0.86	0.35	0–1
Participants	71	99	214	524.5	1300.1	16–9,187
Schools	63	85	193	7.7	14.3	1–121
Districts	57	81	186	2.0	2.41	1–10
Study design and implementation						
% QES	71	99	214	0.24	0.43	0–1
% Implementation problems	71	99	214	0.35	0.48	0–1
Outcome assessment						
% General test	71	99	214	0.45	0.47	0–1
% Follow‐up test	71	99	214	0.077	0.23	0–1
Participant characteristics						
% Girls	65	93	200	47.1	14.8	0–100
Grade	71	99	214	8.3	1.33	6.7–11.5
% Minority	58	83	182	73.4	24.7	6–100
Low income	45	65	124	62.1	20.8	5–100
General intervention characteristics						
% Mathematics tests	71	99	214	0.32	0.44	0–1
Duration in weeks	71	99	214	27.4	19.3	1–120
Number of sessions	56	77	182	75.3	88.0	5–480
Hours per week	53	70	176	2.95	3.62	0.6–30
Implemented by school staff	61	88	196	0.68	0.47	0–1
Implemented by researchers	61	88	196	0.20	0.41	0–1
Instructional methods						
Coaching of personnel	71	99	214	0.25	0.44	0–1
CAI	71	99	214	0.22	0.42	0–1
Incentives	71	99	214	0.12	0.33	0–1
Other method	71	99	214	0.14	0.34	0–1
Peer‐assisted	71	99	214	0.29	0.46	0–1
Progress monitoring	71	99	214	0.25	0.44	0–1
Small group	71	99	214	0.33	0.47	0–1
Multiple instructional methods	71	99	214	0.46	0.50	0–1
Content domain						
Comprehension	71	99	214	0.45	0.50	0–1
Decoding	71	99	214	0.30	0.46	0–1
Fluency	71	99	214	0.17	0.38	0–1
Spelling and writing	71	99	214	0.21	0.41	0–1
Vocabulary	71	99	214	0.34	0.48	0–1
Multiple reading areas	71	99	214	0.52	0.50	0–1
Algebra/prealgebra	71	99	214	0.10	0.30	0–1
Fractions	71	99	214	0.04	0.20	0–1
Geometry	71	99	214	0.03	0.17	0–1
Operations	71	99	214	0.04	0.20	0–1
Probability	71	99	214	0.02	0.14	0–1
Multiple math areas	71	99	214	0.16	0.37	0–1
Meta‐cognitive strategies	71	99	214	0.43	0.50	0–1
Social‐emotional skills	71	99	214	0.08	0.27	0–1
General academic skills	71	99	214	0.12	0.33	0–1
Single component interventions						
Coaching of personnel	71	99	214	0.05	0.22	0–1
CAI	71	99	214	0.01	0.10	0–1
Incentives	71	99	214	0.04	0.20	0–1
Peer‐assisted	71	99	214	0.08	0.27	0–1
Progress monitoring	71	99	214	0	0	0
Small group	71	99	214	0.21	0.41	0–1
Comprehension	71	99	214	0.04	0.20	0–1
Decoding	71	99	214	0.03	0.17	0–1
Fluency	71	99	214	0	0	0
Spelling and writing	71	99	214	0	0	0
Vocabulary	71	99	214	0.01	0.10	0–1
Algebra/prealgebra	71	99	214	0.05	0.22	0–1
Fractions	71	99	214	0.04	0.20	0–1
Geometry	71	99	214	0	0	0
Operations	71	99	214	0	0	0
Probability	71	99	214	0	0	0
Meta‐cognitive strategies	71	99	214	0.09	0.08	0–1
Social‐emotional skills	71	99	214	0.01	0.01	0–1
General academic skills	71	99	214	0.02	0.02	0–1

*Note:* The number of studies that provided information about a variable is denoted *k*, the number of interventions *i* and the number of effect sizes *n*. The mean, standard deviation of the mean, and the range is taken over interventions.

Abbreviations: CAI, computer‐assisted instruction; QES, quasiexperimental study.

Included interventions were to a large extent, 87%, performed in the United States. The remaining interventions were from Canada (4%), Germany (4%), the United Kingdom (UK; 3%), the Netherlands (2%) and Australia (1%).^5^ The mean number of participants, schools, and districts were 525, 8 and 2, respectively, but sample sizes varied quite widely and many studies were small. Most included study designs were RCTs, 24% of interventions were studied in a QES. Around 35% of interventions reported having some form of implementation problem, and slightly less than half the tests tested more than one reading or mathematics domain, that is, they were general in our terminology. There were very few follow‐up tests (in 8% of interventions). Participants were slightly more likely to be boys (47% were girls), and most were minority (73%) and low income students (62%). Note that information about minority and, in particular, low income students were relatively often missing (information missing for 16 and 34 interventions, respectively).

There were more interventions using reading tests, 32% used a mathematics test. Note that the separation of effect sizes was made based on the test, not the subject targeted, as there were several interventions (*i* = 11) that used tests in both reading and mathematics. This is also the main reason why we do not separate results into reading and mathematics interventions to start with (we will return to this issue in the analysis of heterogeneity).

The mean duration was about 27 weeks, and the mean frequency and intensity equalled 75 sessions and 3.0 hr per week. The ranges for all three variables measuring dosage were wide and note that we lack information for quite a few interventions regarding frequency and intensity (for 22 and 29 interventions, respectively). The same is true to a somewhat lesser extent for the variables characterising the implementer. Among the interventions with information, 68% was implemented by school staff, and 20% by researchers, and the rest by other categories, such as college students.

The proportion of instructional methods ranged from 12% of interventions using an incentive component to 33% that used small group instruction. In 14% of the interventions (*i* = 14) there was no component included that matched the categories laid out in the Instructional Methods section, and they were coded in the other method‐category. Eight of these interventions contained instruction in groups smaller than whole class, but larger than five students. Three provided extra instructional time and three changed only the content, not the instructional method.

Nearly half of the interventions (46%) combined more than one instructional method. Accordingly, as shown at the bottom of the table, the share of interventions using a single instructional method was, for most methods, small. The exceptions are peer‐assisted instruction, which was the only instructional method‐component in 8% of the interventions, and small group instruction, which was the only instructional method‐component in 21% of the interventions.

The proportion of interventions targeting reading domains ranged from 17% that targeted fluency to 45% that targeted comprehension. Most interventions (51%) targeted multiple reading areas. For most math domains there were few interventions and we were unable to analyse heterogeneity over math domains any further. Lastly, while there were many interventions targeting meta‐cognitive strategies (43%), fewer targeted social‐emotional skills (8%) or general academic skills (12%). In the rest of the analysis, we combined meta‐cognitive strategies, social‐emotional skills, and general academic skills into one domain, called other domain, due to the low number of interventions targeting the latter two domains. None of the domains was the only targeted domain in more than 10% of the interventions.

#### 6.1.3 Excluded studies

Due to the large amount of studies screened in full text, we were unable to describe all excluded studies. This section instead describes studies that met almost all our inclusion criteria and that one may have thought should be included. They therefore exemplify how we applied the inclusion criteria.

The included study designs contrasted intervention and control groups, or alternative interventions, to estimate effects. Gajria and Salvia (1992) used a randomised design where one intervention group consisting of students with learning disabilities were given training in summarisation strategies. The control group of students with learning disabilities did not receive this training. Results were also compared to a group of students without learning disabilities, which we deemed to be not comparable to the intervention group. Furthermore, only the intervention group was given a standardised postintervention test (there were researcher developed tests given to all groups). For the measure we wanted to include, the design was therefore a single group pre‐post design, and we excluded the study.

We included interventions that sought to improve academic achievement or specific academic skills. We however excluded interventions that only sought to improve performance on a single test instead of improving a skill that would improve test scores. Two studies about incentives provide contrasting examples: Levitt, List, Neckermann and Sadoff (2016) provided students with incentives to perform well on a single test, where students were unaware of the incentives right up until they arrived at the testing session. In Levitt, List, and Sadoff (2016) students were provided monthly financial incentives for meeting achievement standards based on multiple measures of performance including attendance, behaviour, grades and standardised test scores. We excluded the former study and included the latter, as the former did not target academic skill building, just motivation to perform on a particular test, whereas the latter did target skill building.

Interventions had to be targeting students with or at risk of academic difficulties to be included. Glazerman et al. (2013) examined a transfer‐incentive programme for high‐performing teachers, which aimed to increase the supply of effective teachers in struggling schools. Although these schools served disadvantaged students, we excluded the study because the programme did not specifically target students with or at risk of academic difficulties, but all students, including high‐performing students, in these schools. Voight and Velez (2018) study an intervention where more than 50% of the participants were minority students and students receiving free‐ or reduced price lunches. However, as the intervention group average was higher than the national average on a normed test, the intervention included mainly high‐performing students. Therefore, we excluded the study.

Interventions should be school‐based to be included, meaning that they were performed in school during regular school‐hours and semesters. Munoz et al. (2008) and Munoz et al. (2012) studied tutoring‐programmes for at risk students within the United States Supplemental Educational Services programme. As the tutoring was only offered outside of regular school hours (e.g., before or after school, on weekends, or during the summer) by other providers than schools themselves, we excluded these studies because the interventions were not school‐based.

Studies had to test the effects of interventions using standardised tests in reading and mathematics. Reyes and Jason (1991) used the Test of Academic Proficiency, which is a standardised test with a composite score in reading, mathematics, and writing. As we did not include writing tests, and it is unclear whether composite scores can be compared to tests in reading or mathematics, we excluded the study.

### 6.2 Risk of bias in included studies

Because they had too high risk of bias, many effect sizes and 118 included studies were not included in the meta‐analyses. That is, our assessment was that they were more likely to mislead than inform the analysis. There was no study in which we excluded one effect size and included others. The reasons for giving a too high risk of bias rating were primarily related to the study design, or affected all effect sizes more or less equally. The most common reasons for giving a too high risk of bias rating were: confounding of intervention effects with for example school, teacher, or class effects (48 studies), for example, when there was only one intervention and one control school (we drew the line at two intervention and two control units); inadequate control for confounding factors (36 studies), for example, a QES without any statistical controls or with very large preintervention imbalances on important confounders; noncomparable intervention and control groups (20 studies), for example, when at‐risk students were compared to not‐at‐risk students or voluntary participants were compared to students who declined participation. We excluded the 14 remaining studies for more idiosyncratic reasons.

Almost all studies with too high risk of bias were QES, only seven were RCTs. The RCTs were for example excluded because randomisation was compromised and there was inadequate control for confounding; because of large and differential attrition; or because only one unit was assigned to the intervention or control group. We listed the reasons for giving a rating of too high risk of bias by study in the Studies with a Too High Risk of Bias Rating section, Table A5, in the appendix. We reported the main reason per study, but note that there were cases with more than one reason for too high risk of bias ratings.

**Figure 4:**
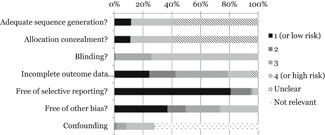
Summary of risk of bias items for studies included in the meta‐analysis

Figure 4 shows the distribution of the assessments for all effect sizes included in the meta‐analysis by the items in the risk of bias tool. See Appendix Risk of Bias in Studies Included in the Meta‐Analysis, Table A4, for a description of the rating per study and item.

Few RCTs reported how they generated the random sequence used to assign students to intervention and control groups, only 12% of effect sizes were given a low risk assessment (QES have high risk by default on this item and therefore also on allocation concealment). More generally, the procedure of randomisation was often not described in detail. In almost all cases where the random sequence generation was described, the allocation was most likely concealed, as the randomisation was not done sequentially.

All studies have problems with the blinding of treatment status, that is, no effect size received a rating of 1. Very few studies provided an explicit discussion about this problem. There was some variation between effect sizes though: For around 73% of effect sizes, no participant group was likely to be blind to treatment status. About 27% of effect sizes had at least one group blinded to treatment (usually the persons performing the tests), and in a few cases, several groups were likely blinded. The ratings for this item vary to some extent also within studies, as some studies used both tests performed by persons outside the study (e.g., statewide tests) and by involved, nonblinded, study personnel.

The distribution of assessments for incomplete outcome reporting was more mixed. Fewer effect sizes had a high risk of bias rating on this item (16%) and almost all studies provided information. We rated a large majority of studies (and effect sizes) to be free of selective reporting, but this does not mean that they followed prespecified protocols or analysis plans. The figure omits the items examining if the study followed an a priori protocol and an analysis plan, as just two studies mention a protocol and an analysis plan written before the analysis was made. Only one study provided information enough that the actual protocol and plan could be located (Rutt, Kettlewell, & Bernardinelli, 2015). About 27% of effect sizes were rated as having a high risk of bias for the other bias item.

The confounding item was only assessed for the 19 QES; 70% of effect sizes from these studies received a rating of 4, that is, high risk of bias. Only 5% of effect sizes from QES received a rating of 2, and none was rated in the lowest risk of bias category.

In sum, the included effect sizes and studies have in general a high risk of bias, but there is also variation. We return to the sensitivity of our results to different part of the risk of bias assessment in Section 6.3.3.

### 6.3 Synthesis of results

#### 6.3.1 Overall short‐run and follow‐up effects

This section presents the results from the robust variance estimation of short‐run effects and effects at follow‐up. Note that the number of clusters used in the robust‐variance estimation differs from the total number of included studies. Somers et al. (2010) was excluded in the analyses of short‐run effects. This study included a mix of the samples in Kemple et al. (2008) and Corrin et al. (2008), which examined the same intervention and were consequently treated as one cluster. Results from Somers et al. (2010) are included in the follow‐up analysis, as none of the other two studies provided results from follow‐up tests. Kim et al. (2011) and Olson et al. (2012) report estimates from the year 1 cohort and year 2 cohort of the same intervention. Vaughn et al. (2011) and Swanson et al. (2015) similarly report effects for two cohorts of one intervention. The effect sizes in these two pairs of studies are therefore unlikely to be independent. We included all effect sizes from these four studies but clustered on the intervention level rather than the study level.

**Figure 5:**
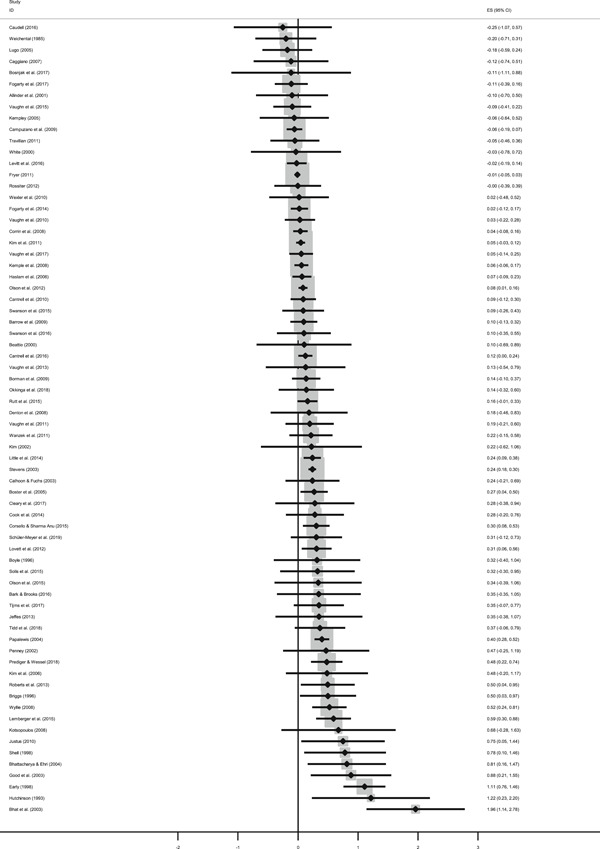
Study level weighted effect sizes with outcomes measured at the end of interventions

There were 194 short‐run effect sizes, 66 clusters, and 79,191 student observations.^6^ There was only one study that did not provide results from a short‐run test. The weighted average short‐run effect size was positive and significant (ES = 0.22, CI = [0.148, 0.284]). This effect size corresponds to a 56% chance that a randomly selected score of a student who received the intervention is greater than the score of a randomly selected student who did not (see, e.g., Ruscio, 2008, for a conversion formula). The *Q* statistic was 302.8., the *τ* ^2^ 0.038, and the *I* ^2^ was 78.5. All three heterogeneity measures therefore indicated substantial heterogeneity. The individual effect sizes ranged from −0.65 to 3.68, again indicating substantial heterogeneity. To provide a further illustration of the heterogeneity of short‐run effects, Figure 5 displays a forest plot of the average effect sizes by study. The study level distribution ranges from −0.25 to 1.96 and there is some evidence of outliers, particularly among the studies with very large positive average effect sizes.^7^ We test for the sensitivity to outliers in Section 6.3.3.

There were 16 effect sizes from seven clusters (equal to the number of studies in this case) measured more than 3 months after the end of intervention, including 21,630 student observations. The ES at follow‐up was small and not statistically significant (ES = 0.05, CI = [−0.096, 0.192]). The number of studies and the degrees of freedom are low enough that the RVE procedure may yield untrustworthy results regarding the standard errors (Tanner‐Smith & Tipton, 2014; Tipton, 2015). However, we got similar results when we, instead of using RVE, averaged effect sizes over studies and used the DerSimonian‐Laird (DL) procedure in the Stata command *metan* (ES = 0.03, CI = [−0.051, 0.117]).^8^ The *Q*‐statistic was 11.9 (RVE) and 8.9 (DL), the *τ* ^2^ 0.007 (RVE) and 0.004 (DL) and the *I* ^2^ 49.5 (RVE) and 32.2 (DL). The low number of studies makes these heterogeneity results relatively unreliable. The individual effect sizes ranged from −0.06 to 0.73, which indicated that there was substantial variation in effect sizes also for the follow‐up measures.

As there were few follow‐up studies and effects, the subgroup analysis and investigation of heterogeneity in the following sections focus only on the short‐run effects.

#### 6.3.2 Results of the subgroup analysis and investigation of heterogeneity

The previous analyses all indicated substantial heterogeneity. This section examines if we can explain some of this heterogeneity by dividing studies further into subgroups by instructional components and content domains, and by using meta‐regressions to examine the associations between intervention characteristics and effect sizes.

**Figure 6:**
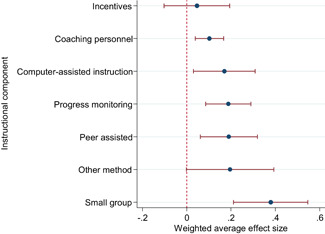
Subgroup analyses: Weighted average effect sizes (ESs) and 95%‐confidence intervals by instructional component

**Figure 7:**
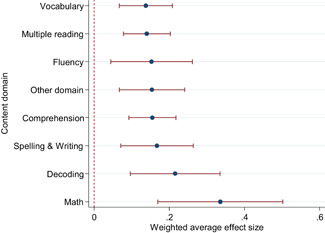
Subgroup analyses: Weighted average effect sizes (ESs) and 95%‐confidence intervals by content domain

Figures 6 and 7 show ESs and 95%‐confidence intervals from RVE estimations by instructional method (Figure 6) and content domains (Figure 7). We derived each effect size from a meta‐regression including just a constant with the outcome variable being the effect sizes from interventions including the component in question. Note that many interventions have more than one component, so the effect sizes should be interpreted as the ES for interventions that *included* a certain component, not the effect size of that component in isolation. Furthermore, many interventions included several components and contributed effect sizes to more than component category. Because there were few studies and interventions that studied subdomains of mathematics (see Table 1), we used a single math‐domain. The effect sizes in the math‐domain were from standardised mathematics tests.

Figure 6 indicates that all instructional components were associated with positive average effect sizes. Small group instruction is associated with the largest effect sizes (ES = 0.38, CI = [0.211, 0.547]) and incentives with the smallest (ES = 0.046, CI = [−0.103, 0.194]). The average effect size is also significantly different from zero for all instructional components, except for incentives and the other method category. There are three main messages from Figure 7: First, all content domains have positive and statistically significant average effect sizes. Second, the differences between the reading domains were small. The average effect sizes were around 0.14‐0.22 and none of them were significantly different from each other. Third, the average effect size for math (ES = 0.34, CI = [0.169, 0.502]) was considerably larger than most other domains.

Table 2 summarises the results for both types of components and includes the number of studies and effect sizes per component as well as results for the three heterogeneity measures. For almost all components, the heterogeneity measures indicated substantial heterogeneity. Incentives and coaching of personnel were partial exceptions, but the *Q* statistic was significant also for these components.

**Table 2:** Subgroup analyses: Effect sizes, confidence intervals, number of studies and effect sizes, and heterogeneity measures by component

Component	Average effect size	95% confidence interval	*k*	*n*	*N*	*Q*	*τ* ^2^	*I* ^2^
Incentives	0.046	−0.1030, 0.1943	8	36	21,236	10.9	0.0060	35.9
Coaching personnel	0.102	0.0383, 0.1664	16	39	22,827	20.7	0.0037	27.5
CAI	0.170	0.0306, 0.3088	14	49	9,502	53.3	0.044	75.6
Progress monitoring	0.188	0.0860, 0.2900	19	35	8,670	51.4	0.035	66.6
Peer‐assisted	0.190	0.0614, 0.3188	22	57	33,161	88.9	0.034	76.4
Other method	0.196	−0.0020, 0.3937	12	37	7,663	25.4	0.059	56.6
Small group	0.379	0.2112, 0.5469	22	57	8,162	98.3	0.086	81.7
Vocabulary	0.137	0.0669, 0.2085	23	102	41,381	81.6	0.027	73.0
Multiple reading	0.140	0.0781, 0.2026	35	135	70,267	147.5	0.023	76.9
Fluency	0.153	0.0442, 0.2612	10	62	33,541	38.6	0.039	76.7
Other domain	0.154	0.0667, 0.2411	28	83	43,380	76.5	0.016	64.7
Comprehension	0.155	0.0923, 0.2178	32	120	44,180	88.0	0.023	64.8
Spelling and writing	0.167	0.0707, 0.2638	13	37	37,491	68.3	0.025	82.4
Decoding	0.216	0.0962, 0.3351	19	101	38,101	89.0	0.046	79.8
Math	0.336	0.1693, 0.5019	25	36	14,961	158.5	0.092	84.9

*Note*: The number of studies contributing to the average effect size is denoted *k*, the number of effect sizes is denoted *n*, and the number of student observations is denoted *N. N* is calculated by taking the sample size for each effect size included in an estimation, which is equal to the number of students in the intervention and control group for which the effect size is calculated, and summing over effect sizes.

Abbreviation: CAI, computer‐assisted instruction.

This component‐by‐component analysis has a few important drawbacks. If there is correlation between components, that is, when interventions contain more than one component, we risk confounding the association of one component with the others. As shown in Table 1, several components were always used in combination with at least one other component and there are many combinations, so this risk is pertinent. One way to mitigate this problem would be to examine interventions that only included one instructional method or one content domain and run separate regressions using only effect sizes from these interventions.

However, for most components, we encountered degrees of freedom problems in the RVE procedure when we examined single component interventions. The exceptions for instructional methods were peer‐assisted instruction and small group instruction, which both showed large, positive, and significant associations with effect sizes (ES = 0.44, CI = [0.011, 0.869] for peer‐assisted instruction and ES = 0.56, CI = [0.307, 0.808] for small group instruction). Regarding content domains, it was only the combined category for meta‐cognitive, social‐emotional, and general skills (i.e., the other domain) and the combined categories for math and reading that did not have degrees of freedom problems. The average effect size was positive but not statistically significant for the other domain (ES = 0.20, CI = [−0.119, 0.519]), and positive and significant for math (ES = 0.35, CI = [0.047, 0.656]) and reading (ES = 0.20, CI = [0.109, 0.293]).

The component‐by‐component analyses do not take into account other moderators and we therefore risk confounding the instructional components and content domains with other study, participant and intervention characteristics. Next, we therefore performed meta‐regressions where we included indicators of intervention components and moderators based on study, participant and intervention characteristics in the same regression. However, as discussed in the Subgroup Analysis and Investigation of Heterogeneity section, there were not enough studies and effect sizes to include all potentially important moderators. To prune the set of moderators, we included only moderators without missing observations and then examined the correlations between them. Table 3 shows a matrix with the pairwise correlation between effect sizes (the two continuous variables, grade and duration, were mean‐centred). Correlations over 0.5 are marked in bold. Note in particular that moderators indicating content domains were highly correlated with each other. There were fewer highly correlated moderators among the instructional methods, but the correlations between CAI and incentives, and CAI and QES were higher than 0.5. There were also relatively high negative correlations between the indicator for mathematics test and some of the reading domains.

**Table 3:** Correlation matrix for moderators

Moderator	(1)	(2)	(3)	(4)	(5)	(6)	(7)	(8)	(9)	(10)	(11)	(12)	(13)	(14)	(15)	(16)	(17)	(18)	(19)	(20)
1. Coaching of personnel	1																			
2. CAI	−.12	1																		
3. Incentives	−.25	.**51**	1																	
4. Other method	−.24	−.26	−.21	1																
5. Peer‐assisted	.39	−.18	−.23	−.30	1															
6. Progress monitoring	.03	.26	−.02	−.21	−.06	1														
7. Small group	−.25	−.16	−.25	−.30	−.32	.16	1													
8. Comprehension	.30	.24	.08	.12	.17	−.00	−.46	1												
9. Decoding	.02	−.01	.16	.10	−.10	−.29	−.22	.**59**	1											
10. Fluency	.09	−.35	−.27	.23	.08	−.19	−.15	.45	.**68**	1										
11. Spelling and writing	.39	−.05	−.18	−.18	.08	.13	.04	.33	.23	.37	1									
12. Vocabulary	.18	.27	.16	.07	−.09	−.00	−.20	.**71**	.**60**	.46	.39	1								
13. Multiple reading	.17	.13	.19	−.00	.02	−.08	−.20	.**64**	.**62**	.48	.36	.**69**	1							
14. Other domain	.27	−.09	.22	−.11	−.00	−.24	−.12	−.16	−.13	−.31	.09	−.19	−.12	1						
15. Math	−.14	−.06	−.06	−.11	.01	.09	.16	‐.**55**	−.48	−.30	−.15	−.49	‐.**60**	.09	1					
16. QES	−.13	**.50**	.42	−.23	−.10	.02	.02	.13	.11	−.35	.09	.13	.32	.11	−.20	1				
17. General test	−.03	−.02	.05	.06	−.04	.01	.01	−.35	−.34	−.24	0.13	−.23	−.27	.29	.37	−.06	1			
18. Grade	.14	−.20	−.28	−.06	.16	.11	−.06	−.25	−.06	.01	0.06	−.33	−.12	.09	.17	−.12	.19	1		
19. Duration	.32	−.20	−.25	.03	−.06	.35	.10	.12	.06	.26	0.36	.19	.20	.02	−.07	−.11	.12	.10	1	
20. Implementation problems	.21	.35	.34	−.37	.17	−.14	−.29	.24	.37	.15	0.04	.27	.29	.08	−.19	.09	−.12	.14	−.23	1

*Note:* Correlations over 0.5 are marked in bold.

High correlations between moderators increases the risk of multicollinearity and makes it less likely that we can estimate meaningful separate associations. Considering the correlations in Table 3, this problem seems to be particularly high for content domains. The correlations between instructional methods in Table 3 are not as high as for content domains (but they are clearly not zero). We therefore focused the further analysis on the instructional components.

Table 4 displays the results from meta‐regressions where we included all end of intervention effect sizes from both math and reading tests. The component estimates are estimated from both within‐ and between‐study variation, as some studies included more than one intervention, which in turn included different components. However, there is much more between‐study variation.

**Table 4:** Results from meta‐regressions examining differences in effect sizes

Moderator	(1)	(2)	(3)	(4)	(5)
Math	0.157 [−0.022, 0.337]		0.175* [0.007, 0.343]	0.207* [0.0428, 0.371]	0.188* [0.039, 0.336]
QES		0.174 [−0.016, 0.364]	0.192* [0.015, 0.368]	0.218* [0.0136, 0.423]	0.242* [0.032, 0.452]
*Coaching of* personnel				−0.056 [−0.206, 0.0937]	−0.015 [−0.172, 0.142]
CAI				−0.240* [−0.465, −0.0156]	−0.277* [−0.532, −0.023]
Incentives				−0.156 [−0.396, 0.085]	−0.151 [−0.390, 0.087]
Peer‐assisted				−0.064 [−0.258, 0.131]	−0.107 [−0.317, 0.104]
Progress monitoring				0.020 [−0.130, 0.170]	0.082 [−0.090, 0.255]
Small group				0.084 [−0.111, 0.278]	0.109 [−0.079, 0.296]
Other domain				−0.105 [−0.285, 0.075]	−0.098 [−0.289, 0.094]
General test					0.004 [−0.137, 0.144]
Grade					0.013 [−0.049, 0.074]
Duration					−0.004 [−0.010, 0.003]
Implementation problems					0.006 [−0.110, 0.121]
Constant	0.173** [0.113, 0.234]	0.171** [0.103, 0.239]	0.119** [0.056, 0.182]	0.238** [0.054, 0.423]	0.225* [0.035, 0.414]
*Q*	300.2	265.2	264.8	201.8	183.9
*τ* ^2^	0.040	0.037	0.038	0.045	0.050
*I* ^2^	78.7	75.9	76.2	72.2	71.7

*Note:* Confidence intervals, based on robust standard errors, in brackets. The number of clusters is 66, the number of effect sizes is 194, and the number of student observations is 79,191 in all specifications. The number of student observations is calculated by taking the sample size for each effect size included in an estimation, which is equal to the number of students in the intervention and control group for which the effect size is calculated, and summing over effect sizes.

Abbreviations: CAI, computer‐assisted instruction; QES, quasiexperimental study.

* *p* < .05.

** *p* < .01.

In column 1, we included a single moderator indicating if the basis for the effect size was a mathematics test. The coefficient of the constant can therefore be interpreted as the average effect size based on reading tests. Column 2 includes a single indicator for QES, so the coefficient of the constant represents the average effect size in RCTs. The specification in column 3 includes both indicators for QES and math‐tests; the constant is therefore the average effect size from reading tests in RCTs. In column 4, we added the six instructional components and a moderator indicating that the intervention targeted other domains than (or possibly in addition to) reading or math content. The constant represents the average effect size from reading tests in RCTs of interventions that did not use any of the included components (i.e., interventions coded in the other method category that did not focus on meta‐cognitive, social‐emotional or general academic skills). Finally, column 5 includes indicators for general tests and implementation problems, the mean grade, and the mean duration along with all the previous moderators. The constant becomes harder to interpret in this regression. It represents the average effect size from reading tests in RCTs of interventions that did not use any of the included components, and had a value of zero on the three added variables (i.e., was exactly at the mean). However, no effect size is likely to be exactly at the mean value of all three variables.

Although there is a relatively large number of moderators compared to the number of clusters in columns 4 and 5, the small sample corrected degrees of freedom were not close to the level where robust‐variance estimation starts to perform poorly (Tanner‐Smith & Tipton, 2014; Tipton, 2015). None of our moderators has below 10 degrees of freedom in any specification.

The associations between moderators and effect sizes were consistent over specifications in Table 4 in the sense that no moderator changed sign when we added more variables. The *Q* statistic was reduced the more moderators we added and ranged from 300.2 (column 1) to 184.8 (column 5). These numbers can also be compared to the heterogeneity in the overall short‐term effects (*Q* = 302.8). However, there was still significant heterogeneity and adding moderators did not greatly reduce *I* ^*2*^.

There were few statistically significant moderators, despite some coefficients being relatively large in relation to the baseline effect. Mathematics tests were associated with significantly larger effect sizes than reading tests and QES were associated with significantly larger effect sizes than RCTs in the specifications in columns 3–5 where we included other moderators. The differences are around 0.2 for both moderators. The average effect size in RCTs was still reasonably large and significant in column 2 (ES = 0.17, CI = [0.105, 0.238]).

Two instructional components were associated with larger effect sizes than the baseline effect—progress monitoring and small group instruction—but none of them were statistically significantly different. CAI was associated with smaller effect sizes than the mean and the coefficient was relatively large and significant. Other components with relatively large negative but not statistically significant coefficients were incentives, peer‐assisted instruction, and other domain. The coefficients on general tests, grade, duration, and implementation problems were all small and not significant.

The lack of statistical significance in Table 4 does not necessarily mean that there are no differences between the components. In Table 5, we used the specification in column 5 of Table 4 and report the results from tests, which examined if the coefficients of the intervention components were significantly different from each other. There were two significant differences out of 21 tests: small group instruction was associated with significantly larger effect sizes than CAI and incentives.

**Table 5:** Tests of differences between intervention components

Test	Coefficient difference (1)	*F* statistic (2)	*df* (3)	*p* value (4)
Coaching of personnel = CAI	0.263	2.75	27.86	0.109
Coaching of personnel = incentives	0.136	1.92	15.94	0.185
Coaching of personnel = peer‐assisted	0.093	0.36	17.23	0.554
Coaching of personnel = progress monitoring	−0.090	0.61	17.42	0.445
Coaching of personnel = small group	−0.121	1.26	19.10	0.275
Coaching of personnel = other domain	0.088	0.36	26.49	0.553
CAI = incentives	−0.127	0.60	15.49	0.449
CAI = peer‐assisted	−0.170	2.10	16.79	0.165
CAI = progress monitoring	−0.353	4.84	9.56	0.054
CAI = small group	−0.384	7.71	20.80	0.011
CAI = other domain	−0.175	2.55	9.59	0.142
Incentives = peer‐assisted	−0.043	0.08	21.79	0.784
Incentives = progress monitoring	−0.226	2.26	17.11	0.151
Incentives = small group	−0.257	4.99	15.99	0.040
Incentives = other domain	−0.048	0.09	22.81	0.771
Peer‐assisted = progress monitoring	−0.183	2.04	16.92	0.172
Peer‐assisted = small group	−0.214	4.13	21.72	0.055
Peer‐assisted = other domain	−0.005	0.00	19.16	0.959
Progress monitoring = small group	−0.031	0.06	15.41	0.803
Progress monitoring = other domain	0.178	2.77	21.01	0.111
Small group = other domain	0.209	3.53	22.66	0.073

*Note:* Column 1 reports the difference between the coefficients of the two components mentioned in the Test‐column. To calculate the *F* statistic, the degrees of freedom, and the (two‐sided) *p* value in columns 2–4, we used the test described in Tipton and Pustejovsky (2015) and implemented it using an extension to the function *Wald_test* and the “HTZ” small‐sample correction procedure contained in the R package *Wald_test* (Pustojevsky, 2019). The coefficients and variance estimates are from the model reported in Table 4, column 5.

The specifications in Table 4 and the tests reported in Table 5 are an attempt to get closer to isolating the association between intervention components and effect sizes. However, this approach has its own drawbacks. Importantly, as there are many potentially omitted variables, we want to emphasise that the coefficients in Table 4 represent associations and not causal effects. For example, Table 4 does not contain any interactions among instructional methods and many interventions change more than one instructional method. We could not investigate combinations of instructional methods because no combination of methods was included in enough interventions. For all combinations, the RVE procedure ran into degrees of freedom problems when we ran meta‐regressions including just a constant with the outcome variable being the effect sizes from interventions including the combination of interest (i.e., without adding additional moderators).

Adding more moderators may help account for confounding, but by adding moderators that do not explain much variation we may lose statistical power. Including many moderators may moreover cause multicollinearity. There were for example two pairs with a higher than 0.5 correlation in Table 3: QES and CAI (0.50), and incentives and CAI (0.51). Furthermore, multicollinearity does not only arise from pairwise correlations and may therefore be a problem for the other estimates as well. That is, we may lack sufficient variation to get precise estimates of the moderators’ associations with effect sizes in the more comprehensive specifications in columns 4 and 5 of Table 4. On the other hand, we are testing multiple hypotheses and should adjust the *p*‐values accordingly. We did not prespecify a multiple hypothesis testing adjustment but none of the significant differences survive an adjustment for the 21 tests in Table 5 using the procedure suggested by Holm (1979). We would have to allow a false discovery rate of 24% for the test with the lowest *p* value to be significant using the adjustment procedure suggested by Benjamini and Hochberg (1995).

In sum, our analysis of heterogeneity revealed that almost all intervention components are associated with positive and significant effect sizes in at least one type of analysis (the exception is incentives). However, our evidence of differences in effect sizes between intervention components and our evidence of how components should be combined was relatively weak.

#### 6.3.3 Results of sensitivity analyses

The sections below report results from our sensitivity analyses. We tested whether effect sizes were associated with effect size measurement, adjusting for outliers, adjusting for clustered assignment of treatment, multiply imputing moderators with missing observations, risk of bias, and finally, publication bias. For all but the last analysis, we concentrated on differences between effect sizes based on mathematics and reading, and RCTs and QES. That is, we focused on the two moderators associated with the largest and the most consistently significant differences in effect sizes. The reason was that the main analysis either indicated small(er) differences or did not have enough power to detect differences between other moderators. We used the RVE procedure and estimated, when possible, separate specifications for QES and RCTs, and for effect sizes based on reading and mathematics tests.

**Figure 8:**
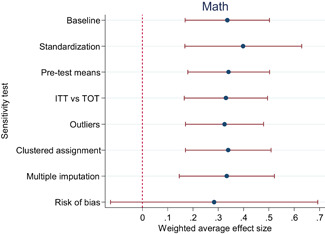
Sensitivity analyses: Effect sizes based on mathematics tests

**Figure 9:**
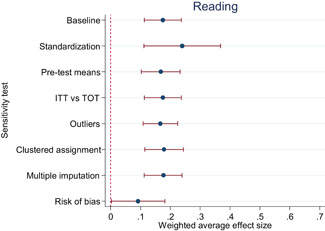
Sensitivity analyses: Effect sizes based on reading tests

**Figure 10:**
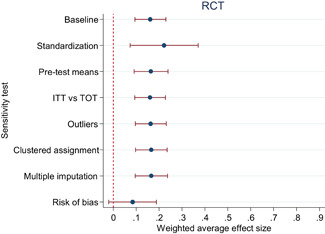
Sensitivity analyses: Effect sizes in randomised controlled trials

**Figure 11:**
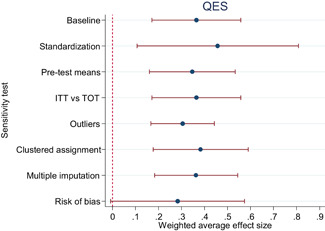
Sensitivity analyses: Effect sizes in quasiexperimental study

Figures 8, 9, 10, 11 show the results for effect size measurement, outliers, clustered assignment of treatment, moderators with missing observations, and risk of bias. The baseline average effect size and confidence interval for mathematics tests (Figure 8), reading tests (Figure 9), RCTs (Figure 10) and QES (Figure 11) is shown at the top of each graph, and the changes to the effect size and confidence interval is displayed by the type of sensitivity analysis.^9^ The results for multiply imputing missing values and risk of bias are also reported separately in Tables 6 and 7. Regarding publication bias, we focused on the full sample of studies.

**Table 6:** Multiply imputed moderators

Moderator	(1) MI, math	(2) MI, reading	(3) MI, RCT	(4) MI, QES
Proportion of girls	0.0045 [−0.0197, 0.0288]	0.00245 [−0.0039, 0.0088]	0.0046 [−0.0039, 0.0130]	−0.0112 [−0.0369, 0.0145]
Proportion of minority students	0.0002 [−0.0086, 0.0090]	−0.00064 [−0.0039, 0.0027]	−0.00003 [−0.0024, 0.0023]	−0.0013 [−0.0212, 0.0187]
Proportion of low income students	−0.0009 [−0.0120, 0.0101]	0.00053 [−0.0042, 0.0053]	0.00079 [−0.0035, 0.0051]	−0.0019 [−0.021, 0.017]
Constant	0.333 [0.146, 0.522]	0.177 [0.113, 0.241]	0.167 [0.0958, 0.237]	0.364 [0.183, 0.544]

*Note:* Confidence intervals, based on robust standard errors, in brackets. The number of clusters, effect sizes, and student observations in the respective specifications are 25, 36 and 14,961 in column 1; 51, 158 and 64,230 in column 2; 49, 135 and 50,499 in column 3 and 17, 59 and 28,692 in column 4.

Abbreviations: RCT, randomised controlled trial; QES, quasiexperimental study.

**Table 7:** Sensitivity to risk of bias items

Moderator	(1) Math	(2) Reading	(3) RCT	(4) QES
High risk	0.077 [−0.329, 0.483]			
Blinding		0.034 [−0.076, 0.144]	0.007 [−0.117, 0.131]	
Incomplete outcome data		0.119 [−0.103, 0.341]	0.257 [−0.014, 0.528]	
Selective reporting		−0.037 [−0.158, 0.083]	−0.006 [−0.150, 0.137]	
Other bias		0.142 [−0.060, 0.345]	0.223 [−0.007, 0.454]	
Confounding		0.288* [0.023, 0.553]		0.199 [−0.180, 0.579]
Constant	0.284 [−0.125, 0.693]	0.092* [0.002, 0.182]	0.085 [−0.019, 0.189]	0.283 [−0.007, 0.574]

*Note:* Confidence intervals, based on robust standard errors, in brackets. The number of clusters, effect sizes, and student observations in the respective specifications 25, 36, and 14,961 in column 1; 51, 158, and 64,230 in column 2; 49, 135, and 50,499 in column 3; and 17, 59, and 28,692 in column 4.

Abbreviations: RCT, randomised controlled trial; QES, quasiexperimental study.

* *p* < .05.

##### Effect size measurement

Including indicators for effect sizes standardised with a super‐population and effect sizes calculated with only the control group standard deviation (i.e., Glass's delta) did not change our results greatly. We could not include an indicator for Glass’ delta for effect sizes based on mathematics tests and for QES, as the variables were collinear. The specification for mathematics tests and QES therefore only included the indicator for standardising with a super‐population. The indicators for uncommon standardisation were always negatively associated with effect size but never significant in any specification, although the indicator for standardising with a super‐population is sometimes large. Adjusting for pretest means did not affect any of our results. Only one study, Cook et al. (2014), reported ITT and TOT estimates. Using the ITT estimates from this study instead of the TOT estimates did not visibly affect any of our results.

##### Outliers

We examined the distributions of effect sizes for the presence of outliers. There are no clear outliers in the lower end of the distribution, and only a few in the upper end. We excluded (not shown in figure) or Winsorized all effect sizes with *g >* 1 (seven effect sizes, which were given the value 0.98). Our results were, as can be seen in the figure, not sensitive to Winsorizing the outliers. Simply removing the outliers did not greatly affect the estimates for reading tests (ES = 0.16, CI = [0.102, 0.215]) and RCTs (ES = 0.15, CI = [0.088, 0.217]) but had a larger impact on the effect sizes for mathematics based tests (ES = 0.26, CI = [0.121, 0.408]) and for QES (ES = 0.25, CI = [0.138, 0.371]). The latter two average effect sizes are still statistically significant (*p* < .01) and well within the confidence interval for the respective baseline effect size.^10^

##### Clustered assignment of treatment

We tested sensitivity to clustered assignment of treatment by adjusting effect sizes with the methods described in the Unit of Analysis Issues section. The confidence intervals are wider and the average effect sizes larger in all four cases, but the differences to the baseline estimates are very small.^11^

##### Moderators with missing observations

We used multiple imputation to account for missing values in some moderators. We focused on moderators that were relevant for all types of interventions: the proportion of girls, the proportion of minority students, and the proportion of low‐income students. Table 6 shows the results for these moderators. The coefficients on all three moderators are small and not significant.^12^ Adding these moderators did not change the baseline results as can also be seen in Figures 8, 9, 10, 11.

##### Risk of bias

We tested sensitivity to risk of bias in the following way. For the specifications including effect sizes based on reading and mathematics tests, we included indicators for numerical items in the risk of bias assessment. For blinding, incomplete outcome reporting, other bias and confounding, we contrasted effect sizes given a rating of 4 to those given lower ratings. For the selective outcome reporting item, we contrasted those rated 1 with those given higher ratings. Confounding is only relevant for QES and this item was therefore excluded in the specification including only RCTs. The number of studies with effect sizes based on mathematics tests and the number of QES were too small for it to be possible to include all item‐indicators. We created a high‐risk indicator equal to one if the effect size had a rating of 4 on any numerical item, which we included for the effect sizes based on mathematics tests. For QES, we focused on confounding and contrasted effect sizes with a rating of 4 to those with lower ratings. The average effect sizes represented by the constant in the regression in columns 1–3 in Table 7 and shown at the bottom of Figures 8, 9, 10 is therefore the average over effect sizes that were rated lower than 4 on blinding, incomplete outcome data, other bias, and confounding, and had a rating of 1 on selective reporting. The corresponding estimate in column 4 of Table 7 and at the bottom of Figure 10 is the average over effect sizes in QES with a lower than 4 rating on confounding.

The average of effect sizes based on mathematics tests with relatively low risk of bias was smaller than the baseline in Figure 8 and the confidence interval was much wider. The results are similar for reading tests, RCTs, and QES (Figures 9, 10, 11): the average effect sizes with relatively low risk of bias are smaller and the confidence intervals wider. The wider confidence intervals are not surprising given that the number of effect sizes rated low on all items (or the confounding item for QES) was small in all cases.

Table 7 contains the results of the sensitivity analysis by item. Only one item‐indicator was statistically significant, the confounding indicator in column 2 (reading tests). The indicators for other bias and incomplete outcome data were however relatively large in columns 2 and 3. The results in column 4 indicated that QES with effect sizes rated 4 on confounding were associated with larger effect sizes but not significantly so (9 out of 17 QES with information were rated 4 on confounding). The association between effect sizes based on mathematics tests and the high‐risk indicator was positive and not statistically significant. Eight out of 25 studies did not have a high risk according to this definition, so there was not much variation.

##### Publication bias

**Figure 12:**
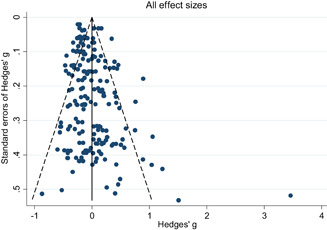
Funnel plot: Mean‐centred effect sizes on the *x* axis and standard errors on the *y* axis

**Figure 13:**
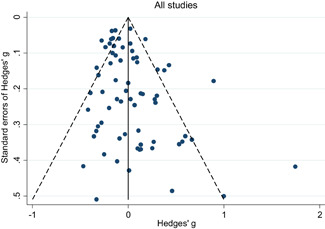
Funnel plot: Mean‐centred study level effect sizes on the *x* axis and study level standard errors on the *y* axis

Figure 12 displays a funnel plot of all short‐run effect sizes on the *x* axis and standard errors on the *y* axis. The effect sizes were mean‐centred, so that the zero‐line in the plot represents the mean effect size. There were, as mentioned, a few outliers with very large, positive effect sizes, but we found no outliers with large, negative effect sizes. Apart from these outliers, there were no clear signs of asymmetry. We got similar results when we used study level averages (see Figure 13).

We also performed Egger's test (Egger et al. 1997) and rejected the null hypothesis of no bias (*p <* .001*)*. However, when we excluded four outliers (study level average *g* > 1), we could no longer reject the null hypothesis (*p* = .099). Lastly, we included an indicator for studies published in scientific journals in the baseline meta‐regression. There was little evidence that published studies contained larger effect sizes than studies that were not published in journals, the coefficient on the journal indicator was small and not significant (*β* = .035, CI = [−0.123, 0.192]).^13^

##### Summary of sensitivity analysis

Summing up the results of the sensitivity analyses, we found few indications that our main results where sensitive to effect size measurement, adjusting for outliers, adjusting for clustered assignment of treatment, and multiply imputing moderators with missing observations. Lower risk of bias was associated with smaller and mostly not significant average effect sizes. However, there were also few low risk studies/effect sizes, which may explain the lack of statistical significance. There were some indications of publication bias, but the asymmetry seemed mostly driven by a few outliers with very large positive effect sizes.

## 7 DISCUSSION

### 7.1 Summary of main results

The main objective of this review was to assess the effectiveness of targeted interventions for students with or at risk of academic difficulties in Grades 7–12, as measured by standardised tests in reading and mathematics. We found in total 247 studies that met our inclusion criteria and included 71 of these studies in meta‐analyses. The reasons for not including studies in the meta‐analyses were that they had too high risk of bias (118), that they compared two alternative interventions instead of an intervention and a control group (38 studies), that we were unable to retrieve enough information to calculate an effect size (13 studies), or that the studies used samples that overlapped with other included studies, and had either a higher risk of bias or contained less information (7 studies). Of the 71 studies, 99 interventions and 214 effect sizes that we included in some meta‐analysis, 76% were RCTs, and the rest were QES.

The ES for short‐run outcomes (measured at the end of intervention) was positive and statistically significant (ES = 0.22, CI = [0.148, 0.284]). This result indicates that interventions for at‐risk students in Grades 7–12 were in general effective at improving standardised test scores but the result includes effects from many types of interventions. All our measures of heterogeneity indicated substantial variation among short‐run effect sizes and the individual effect sizes ranged from −0.65 to 3.68. Only seven studies included standardised tests measured more than three months after the end of intervention. The ES for these longer‐run effects was small and not significant (ES = 0.05, CI = [−0.096, 0.192]). The individual effect sizes ranged from −0.06 to 0.73, which indicated that there was substantial variation in effect sizes also for the follow‐up measures.

Due to the small number of studies providing longer‐run follow‐up measurements, we focused the analysis of heterogeneity and comparative effectiveness of types of interventions on the short‐run outcomes. We examined two main types of intervention components: instructional methods and content domains. As interventions often contained several components, the average effect sizes by component should be interpreted as the average effect of interventions that included a certain component, not the effect of the component in isolation.

Heterogeneity remained substantial within most component categories. Furthermore, interventions that included small group instruction (ES = 0.38, CI = [0.211, 0.547]), peer‐assisted instruction (ES = 0.19, CI = [0.061, 0.319]), progress monitoring (ES = 0.19, CI = [0.086, 0.290]), CAI (ES = 0.17, CI = [0.031, 0.309]) and coaching of personnel (ES = 0.10, CI = [0.038, 0.166]) had positive and statistically significant average effect sizes. Interventions that provided incentives for students did not have a significant average effect size (ES = 0.05, CI = [−0.103, 0.194]). The average effect size in interventions that included none of the above components, but for example provided extra instructional time, instruction in groups smaller than whole class but larger than five students, or changed just the content had a relatively large, but not a statistically significant effect size (ES = 0.20, CI = [−0.002, 0.394]). Relatively few interventions included only one instructional method and we could only calculate average effect sizes in single‐method interventions for peer‐assisted instruction (ES = 0.44, CI = [0.011, 0.869]) and small group instruction (ES = 0.56, CI = [0.307, 0.808]).

The differences between effect sizes from interventions targeting different content domains were mostly small. An exception was that interventions testing effects in mathematics had a relatively large effect size (ES = 0.34, CI = [0.169, 0.502]). Interventions targeting fluency; vocabulary; multiple reading areas; meta‐cognitive, social‐emotional and general academic skills (these three domains were combined into one domain in our analysis); comprehension, spelling and writing, and decoding had average effect sizes ranging from 0.14 to 0.22, all of them statistically significant. Many interventions targeted more than one reading domain and the correlations between the reading domains were relatively high.

To mitigate the risk of confounding the effect of components with each other, and to examine other moderators, we performed meta‐regressions. The number of studies and effect sizes were not large enough that we could include all potentially important moderators in one meta‐regression. Because the content domains were more highly correlated with each other, we focused the analysis on the instructional methods and on moderators without missing observations. The analysis revealed few significant moderators and the unexplained heterogeneity remained high regardless of specification. However, some estimates had a large magnitude in relation to the baseline effects. Depending on the specification, effect sizes were around 0.17‐0.24 higher in QES than in RCTs, and effect sizes based on tests in mathematics were around 0.16–0.21 higher than effect sizes based on reading tests. These differences were statistically significant in the more comprehensive specifications, where we also included other moderators.

Among the instructional methods, small group instruction was associated with significantly larger effect sizes than CAI and incentives. There were no other significant differences between instructional methods. Due to the risk of confounding, our lack of statistical power to detect relatively small differences between instructional methods, and that adjusting for multiple hypothesis testing rendered the differences insignificant, our evidence of differences between components is relatively weak.

In sum, we found evidence of positive and statistically significant overall average effects, but there was substantial heterogeneity throughout the analysis that we were unable to explain by observable intervention characteristics.

### 7.2 Overall completeness and applicability of evidence

We performed a comprehensive search of electronic data bases and national indexes/repositories and trial/review archives, combined with grey literature searching, hand searching of key journals, and extensive citation tracking. In addition, experts in the field were consulted. We found a large number of records, which was screened and coded independently by at least two team members. We were however unable to retrieve all potentially relevant records in full text (*k* = 201). For reasons we discuss in the next section, we believe that they are unlikely to have biased our results.

In line with the comprehensive search, we included many forms of publications: journal articles, conference papers, dissertations, and reports. However, there may still be unpublished studies that we did not find, as educational researchers do not have a tradition of publishing working papers. Publication bias may be another source of missing unpublished studies. We discuss this issue further in the next section. We were also unable to include 13 studies that met our inclusion criteria in the meta‐analysis because they lacked information necessary to calculate an effect size, and we were unable retrieve the missing information from the authors. These studies were more often not published in scientific journals (10 were either reports or dissertations) and were older than the average included study (eight were published before 2000). The studies lacking information were otherwise reasonably similar to the studies included in the meta‐analysis (e.g., 11 were from the United States, one from the UK and one from the Netherlands) and there were few reasons to expect them to have a different impact than our included studies.

Almost all studies included in the meta‐analysis were of interventions performed in the United States. The other countries represented were Canada, Germany, the UK, and the Netherlands, but there were few studies from either of these countries. Our evidence is therefore primarily applicable to the United States. There were 9 studies from other countries than the United States among the studies that met our inclusion criteria, which we for different reasons were unable to include in the meta‐analysis.

We included fewer studies in the later grades and there was in particular no study of students only in Grade 12. There were furthermore very few studies of long‐run effects. We searched for studies back to 1980, but included few studies conducted before 2000. Therefore, we do not believe that the period limit is the cause of the lack of long‐run evaluations.

### 7.3 Quality of the evidence

Many effect sizes were not included in the meta‐analyses because they had too high risk of bias. That is, our assessment was that they were more likely to mislead than inform the analysis. The most common reasons for this assessment were: confounding of intervention effects with for example school, teacher, or class effects because there was only one intervention or one control unit; inadequate (often no) control for confounding factors; and noncomparable intervention and control groups, for example, when at‐risk students were compared to not‐at‐risk students or voluntary participants were compared to students who declined participation. Almost all effect sizes with too high risk of bias were from QES. Reasons for excluding effect sizes from RCTs were for example that the randomisation was compromised and there was inadequate control for confounding; because of very large and differential attrition; or because only one unit was assigned to the intervention or control group.

The effect sizes included in the meta‐analysis had a lower risk of bias. We performed a sensitivity analysis where we adjusted for ratings on the risk of bias items. Effect sizes with a high risk of bias tended to show larger effect sizes, in particular on items such as incomplete outcome data, other bias, and confounding, but few items were significant. The averages of effect sizes with lower risk of bias were still positive and of a meaningful size, albeit with larger confidence intervals than in the baseline analysis. The wider confidence intervals are likely a reflection of the number of studies with low risk of bias, so we do not want to overstate this problem. But it is worth discussing in some more detail what caused the high risk of bias ratings, and how the risk of bias might be decreased.

Information about how the random sequence was generated was lacking in most RCTs, and the randomisation procedure was often sparsely described. As this information is easy to include, this is an area where the reporting of studies can be improved.

Blinding was a problem in all included studies. Complete blinding is difficult to achieve in educational research, but it is for example possible to use testers that are blind to treatment status. Indeed, a substantial minority of studies achieved this by for example by using nationwide or statewide tests that are administered by personnel outside the study, or by hiring external testers. However, we found no strong association between a high rating on the blinding item and effect sizes. One explanation could be that the differences in ratings were small between effect sizes; that is, most that were not rated 4 were rated 3. Another explanation may be that lack of blinding could both bias the results in favour of the intervention group and in favour of the control group (Glennerster & Takavarasha, 2013). For example, if knowledge about treatment status and that they are participating in an experiment make students try harder—that is, a Hawthorne effect—then the beneficial effects are overstated. However, control group students (or their parents) may seek out help elsewhere or try harder because they know they did not get the intervention or because they want to compete with the intervention group (i.e., a John Henry effect). In that case, the beneficial effects are understated. We were unable examine this issue with the material at hand, and, unfortunately, we are not aware of any other study that has examined this issue in educational interventions.

Most studies had a moderate to high risk in terms of incomplete outcome data, or did not provide information. High risk on this item was associated with larger effect sizes, although not significantly so. If attrition by comparatively low‐achieving students in the intervention group is more common, then the effects in our meta‐analysis would be overestimated. However, it seems plausible that successful interventions may also make low‐achieving treated students stay in school or show up at testing occasions at a higher rate than the control group. Such a pattern would instead imply that the effects were underestimated. Incomplete outcome data is difficult to avoid completely. Nevertheless, some studies could do more to mitigate these problems by examining and testing whether there is differential attrition between intervention and control groups, and adjust for such attrition if present. The data needed to perform such tests and adjustments are usually available to study authors.

Skewed data increase the risk of bias when analysing continuous outcomes, particularly in small sample studies. Consequently, such data may bias our meta‐analysis. We found little evidence of problems with skewed data in our risk of bias assessment, although one reason may be that relatively few studies provided information about more moments of the distribution of outcome variables than means and variances. Another problematic feature of the included studies is the near universal lack of prepublished protocols and analysis plans. This made it difficult for us to assess whether there was selective reporting or not, but, more importantly, prepublishing trial protocols and analysis plans could also mitigate researcher bias and promote transparency.

QES were associated with higher effect sizes than RCTs in the main analysis. Many effect sizes from QES were rated high risk of bias on confounding, and these effect sizes tended to be larger than those rated 3 or lower. One feature contributing to this rating is the lack of “natural experiments” that can mimic a randomised experiment in terms of balancing both observable and unobservable confounders between intervention and control groups. Often, studies did not explain why one group of students was assigned to the intervention group and another to the control group. It was therefore difficult to assess, for example, the risk of selection into the intervention. Included effect sizes were reasonably well balanced on observable confounders though.

Beside risk of bias, we tested the sensitivity of our results, separately for effect sizes based on tests in mathematics and reading, and for QES and RCTs, to how effect sizes were measured, to outliers, by adjusting for the clustered assignment of treatment, and by including moderators with missing observations. We also tested if there were indications of publication bias. With the partial exception of the latter, the results were robust.

We found some indications of publication bias. In particular, there were outliers with very large and positive effect sizes but no outliers with large and negative effect sizes. We performed a thorough search for studies not published in scientific journals and we found only a small and not significant difference between effect sizes from studies published in journals and from studies published elsewhere. A possible interpretation of these results is that the missing negative effect sizes are mainly a file‐drawer problem (Rosenthal, 1979). That is, small sample studies that show relatively large negative effects are not written up and are therefore never published in any form. This interpretation is consistent with the evidence presented in Franco, Malhotra, and Simonovits (2014), which indicates that the file‐drawer problem is the main culprit behind publication bias in the social sciences. Note though that there are other possible explanations for the results, which do not involve any publication bias. To give just one example, it may be easier to get large positive effects in studies with very small samples, as students and teachers can be given more attention and be better monitored by researchers. Furthermore, there were few, if any, examples of interventions in our sample that we believe risked having large, negative effects. The lack of outliers with large negative effects was therefore not surprising. When we excluded outliers, the indications of publication bias became weaker, while the significance of the main results was maintained.

### 7.4 Limitations and potential biases in the review process

We performed a comprehensive search and all records were screened and coded by at least two independent screeners in order to minimise the risk of bias in the review process. Three features of the process are however worth discussing in some more detail as they may be a cause for concern.

First, the review team has included many people during the screening and coding phases. All team members were thoroughly introduced to the review methods used, and extensive pilot screening and coding were undertaken in each case. All uncertain cases during both first and second level screening were assessed by at least one review author, in addition to two review team assistants. The number of people involved in the coding of studies was smaller, and the first author was one of at least two coders on all studies included in the analyses, which should increase the level of consistency.

Second, we were unable to retrieve 201 records in full text, which amounts to 5% of the total number of records included after screening of titles and abstracts, and 0.8% of the total number of records. Note that these were records that we could not exclude as obviously irrelevant in the first level screening, not records that necessarily were relevant. Based on the proportions of the included studies, a large share of these records likely pertains to a second review about students in kindergarten to Grade 6 (Dietrichson et al., 2016). Less than 2% of the studies screened in full text were included in any meta‐analysis in this review. Furthermore, older reports from the 1980's and dissertations were overrepresented among the potentially missing studies, which were types of studies that less often met our inclusion criteria. Due to these features, we believe that very few of the 201 studies would have been included in our analyses, and that the risk that our results are biased because of these missing records is low.

Third, almost all interventions were performed in English‐speaking countries, and 86% were from the United States. Although our search was not limited to records written in English and we did find studies in other languages, we had to restrict the included studies to languages that the review team understood (Danish, English, German, Norwegian and Swedish). As a result, we may have missed studies from countries where none of these languages are used. Another reason for the dominance of studies from English‐speaking countries could be the, at least historically, stronger focus on qualitative methods in educational research in some European countries (e.g., Pontoppidan et al., 2018).

### 7.5 Agreements and disagreements with other studies or reviews

All reviews including students with or at risk of academic difficulties in Grades 7–12 that we are aware of have found positive average effect sizes. Few of these reviews include a majority of studies in Grades 7–12 though. Below, we comment on the most closely related reviews that have examined similar intervention types and outcomes. Some of the definitions of intervention types used in these reviews were however not comparable to ours, and we only comment on those parts that we deemed were comparable. There are also differences across the reviews in how outcomes were measured and how effect sizes were calculated. We provide the most comparable average effect sizes from the reviews below, but the reader should be aware that they may still not be fully comparable to our effect sizes. Furthermore, most of the reviews mentioned below did not use meta‐regressions to examine the association of individual intervention components with effect sizes while adjusting for other components/moderators. Therefore, we believe the results from our subgroup analysis where we examine effect sizes by instructional method and content domain, reported in Table 2, are most comparable across reviews.

Interventions including the instructional methods small group instruction, progress monitoring and peer‐assisted instruction have the largest effect sizes in our review. Three similarly defined instructional methods had the largest effect sizes also in Dietrichson et al. (2017), who covered interventions targeting low SES students in elementary and middle school (although a large majority of studies were performed in elementary school). The average effect sizes for interventions including small group instruction and peer‐assisted learning in this review were very close to the average effect sizes for interventions including tutoring (ES = 0.36) and cooperative learning (ES = 0.22) in Dietrichson et al. (2017). Other categories that were similarly defined and had similar average effect sizes were coaching of personnel (ES = 0.16) and incentives (ES = 0.01). Interventions including progress monitoring (ES = 0.32) had a smaller average effect size, and CAI (ES = 0.11) a larger effect size, in this review than in Dietrichson et al. (2017). However, the difference was not large in the latter case, and there were few studies of the former in Dietrichson et al. (2017).

Baye et al. (2018) reviewed interventions for struggling readers in Grades 6–12 and defined struggling readers as students who qualified for special education services but attended mainstream English or reading classes. They found a mean weighted effect size of 0.09. Comparing effect sizes by intervention type, Baye et al. found effect sizes for tutoring by paid adults (ES = 0.24) and cooperative learning (ES = 0.10) that are reasonably similar to our effect sizes for the related categories small group instruction and peer‐assisted learning. Their effect sizes for reading/writing domains ranged between 0.06‐0.13, that is, just slightly below ours.

Gersten et al. (2009) examined math interventions for students with learning disabilities. Their categories teacher feedback combined (ES = 0.23) and peer‐assisted instruction (ES = 0.14) are close to how we defined the components progress monitoring and peer‐assisted instruction. Their effect sizes for these two categories are similar to ours.

Edmonds et al. (2009); Flynn, Zheng, et al. (2012); Scammaca et al. (2015); and Wanzek et al. (2006) reviewed reading interventions for struggling readers (including students in Grades 7–12). They found mainly positive effects but few significant differences over reading domains, in line with what we found. The overall average effects in Scammaca et al. (2015) measured by standardised tests were 0.25 in Grades 6–8 and 0.09 in Grades 9–12. Their effect sizes reported by reading domain included also younger students and were larger than ours for reading comprehension (ES = 0.47) and word study (ES = 0.68), and similar for fluency (ES = 0.17) and multiple components (ES = 0.14). Flynn, Zheng, et al. (2012) found larger effects on standardised tests for comprehension (ES = 0.73), decoding (ES = 0.43), and word identification (ES = 0.41) than we did but found negative effects on fluency (ES = −0.29; they included students in Grades 5‐6 as well). Edmonds et al. (2009) found larger effects on reading comprehension than we do (ES = 0.47; Wanzek et al., 2006, did not conduct a meta‐analysis). Both Flynn, Zheng, et al. (2012) and Edmonds et al. (2009) included relatively few studies in some of their meta‐analyses.^14^

Similar results regarding some instructional methods have also been found in reviews that did not specifically target at‐risk students, but also included studies of at‐risk students. Fryer (2016) included a very large set of different types of interventions. Fryer's incentive and high dosage tutoring categories have partly overlapping definitions with our categories of incentives and small group instruction. Fryer found a small average effect size for incentives (ES = 0.02 in both math and reading), as we did, and an average effect size for high dosage tutoring (ES = 0.30 in math, ES = 0.23 in reading) that is also reasonably close to ours.

Other reviews were more difficult to compare directly to our effect sizes, but reported results that are qualitatively in line with ours. Slavin et al. (2008, 2009) found that instructional‐process programmes had the highest effect sizes for general student populations of different ages. In their definitions, instructional‐process programmes included for example tutoring and cooperative learning, as well as for example classroom management intervention, motivation interventions and teaching of metacognitive strategies.

We found few long‐run effects in our review, but the average effects at follow‐up were smaller than effects measured at the end of interventions. There seems to be very little evidence about long‐run effects for school‐based interventions targeting older students with or at risk of academic difficulties. Suggate (2016) reviewed the long‐run effects of phonemic awareness, phonics, fluency, and reading comprehension interventions up to Grade 7 and included both at‐risk and not‐at‐risk students. Suggate reported positive effect sizes in general, but they were on average around 40% smaller at follow‐up compared to posttest. The mean time between posttest and follow‐up in Suggate's review was around 11 months.

## 8 AUTHORS' CONCLUSIONS

### 8.1 Implications for practice and policy

Our results indicate that interventions targeting students with or at risk of academic difficulties in Grades 7–12 have on average positive and statistically significant short‐run effects on standardised tests in reading and mathematics. We believe the average effect size is of an educationally meaningful magnitude, for example in relation to the gap between groups of at‐risk and not‐at‐risk students, based on for example SES and minority status (Hill, Bloom, Black, & Lipsey, 2008; Lipsey et al., 2012). This means that the most effective interventions in our sample have the potential of making a considerable dent in this gap, should they be targeted only towards at‐risk students. Considering that students typically make smaller gains in higher grades than lower, an equal effect size represents a larger gain in relation to typical progress for older students than younger students (Hollands et al., 2016). With magnitude measured in this way, the effect sizes found in our review were larger than comparable effect sizes from a previous review of interventions targeting predominantly younger at‐risk students (Dietrichson et al., 2017). Thus, the results of this review provide support for trying out school‐based interventions for older students, and evidence against the view that school‐based interventions for older students are in general ineffective.

We want to stress though that our results do not provide a strong basis for prioritising between earlier and later interventions. For that, we would need estimates of the long‐run cost‐effectiveness of interventions. Examining the costs of interventions was outside the scope of this review (and few studies included information about costs). The mean duration of interventions in this review was longer than the mean duration of interventions for younger students in Dietrichson et al. (2017), which measured duration in the same way. This may indicate that school‐based interventions for older students are costlier than similar interventions for younger students. Furthermore, evidence about long‐run effects was scarce in our review, and is lacking more generally in the literature of targeted school‐based interventions. Therefore, evidence about long‐run cost‐effectiveness is also scarce and we are not aware of any study comparable to the literature on targeted preschool interventions, where there is evidence of very long lasting beneficial effects (e.g., Conti, Heckman, & Pinto, 2016; Heckman, Moon, Pinto, Savelyev, & Yavitz, 2010; Reynolds & Temple, 2008; Reynolds, Magnuson, & Ou, 2010). This lack of evidence should not be confused with a lack of cost‐effectiveness. We simply do not know whether the short‐run effects found in many targeted school‐based interventions are lasting, or whether they are cost‐effective.

An objective of the review was to examine and compare the effect sizes of different types of interventions. We primarily wanted to compare interventions in terms of the instructional methods used and the targeted content domains. Effect sizes were very similar over reading domains. Interventions had larger effects on standardised tests in mathematics than on reading tests. While the differences between instructional methods were sometimes sizeable, few were significantly different. An exception was that using small group instruction was associated with significantly larger effect sizes than using CAI and incentives.

Most intervention components were therefore not significantly different from each other. Furthermore, interventions with small effects may still be cost‐effective and in the absence of strong evidence of differential effects, trying the least costly type of intervention may be a good idea. As mentioned, examining the costs of interventions was outside the scope of this review. However, there are clear differences across the instructional methods regarding how much personnel resources are used, which is often the largest proportion of intervention costs (e.g., Hollands et al., 2013). For example, providing incentives typically involves minimal use of personnel, while small group instruction tends to be personnel intensive. In this sense, progress monitoring may seem like a promising candidate: interventions including this component had reasonably large effect sizes, and it does not have to cost much to provide more information about student progress to teachers. However, progress monitoring was always combined with at least one other instructional method, and our evidence for the effectiveness of just implementing more progress monitoring is not strong. Furthermore, progress monitoring may work because it leads to other changes of the instruction, and these changes may be resource intensive. Lastly, the number of included studies and the variation in component combinations was not large enough to examine whether particular combinations of components had higher effect sizes than others.

Our results should be interpreted with at least two caveats in mind. First, we focused the review on outcomes measured by standardised tests in reading and mathematics. The included interventions may have had important effects on other outcomes and interventions that did not improve standardised test scores may well be effective when it comes to other important skills, such as social‐emotional skills,^15^ or outcomes such as grades, dropout and uptake of secondary/tertiary education. We chose to focus on standardised tests for several reasons: to streamline the review, to avoid tests that tested only content inherent to the instruction in the intervention group, and to increase comparability across both time and contexts. Second, the vast majority of included interventions were performed in the United States. Although many included intervention types seem possible to implement also in other contexts, the evidence base, both for and against, targeted interventions to at‐risk students in Grades 7–12 is weak in other countries.

### 8.2 Implications for research

The implications for research to some extent mirror those made for practice and policy. Our results provided no support for the view that targeted school‐based interventions are in general ineffective for older students. On the contrary, the results indicated that school‐based interventions targeting students with or at risk of academic difficulties in Grades 7–12 are on average effective, at least in the short‐run. Thus, the review provides support for continuing to implement and research interventions in these grades.

More research is needed from non‐English speaking countries, as more than 90% of interventions were performed in English speaking countries, in particular the United States. There were also more interventions tested by reading tests than mathematics tests. The latter had larger effect sizes, so targeting mathematics seems like a promising research agenda. The lack of studies examining longer‐run effects seem particularly acute and such studies would be an important addition to the literature.

We found few robust differences between intervention components and the heterogeneity of effect sizes was substantial in all our analyses. The exceptions were that small group instruction was associated with significantly larger effect sizes than CAI and incentives. For other instructional methods, the differences were sometimes sizable, but not statistically significant. Lack of statistical power may be an explanation for the lack of significant differences between for example progress monitoring and incentives, and coaching of personnel and CAI. The differences between reading domains were small. Even with a much larger sample, we would have trouble finding significant differences of these magnitudes, and most are small enough to not be educationally important. We were unable to examine differences between math domains, as there were too few studies.

There were aspects of interventions that were difficult to code systematically for many studies. Most pertinent is, we believe, the difficulty to describe and code the control group condition. Although almost all control groups received treatment as usual‐like instruction, this instruction may vary considerably over studies. It was difficult to classify the control condition, as it was described in much less detail than the intervention condition in many of the included studies. We believe this is an important source of the unexplained heterogeneity found throughout the analyses. We realise that it may often be much more difficult to get precise information about the control group condition, but this is essential information for the interpretation of effect sizes, both in an individual study and for reviews.

The risk of bias of effect sizes found in this review was in general high. We did not include a large number of effect sizes in the meta‐analyses, because of our assessment that they had too high risk of bias. Although some of this risk is difficult or costly to fully mitigate in educational research, we believe it is important to improve research designs and increase sample sizes in studies, especially in terms of the number of units assigned to intervention and control groups. By far the most common reason for giving a too high risk of bias rating was that studies assigned only one unit (e.g., a school, teacher or class) to the intervention group or the control group, in which case the intervention effect is likely to be confounded with “unit”‐effects. There are also several other steps that researchers can take to decrease the risk of bias. Examples include: more detailed reporting about how the randomisation, or more generally the assignment of treatment, was done; using external testers that are blind to treatment status; testing for differential attrition between intervention and control groups; and prepublishing protocols or analysis plans.

## INFORMATION ABOUT THIS REVIEW

## ROLES AND RESPONSIBILITIES

J. D., T. F., M. B., and Anne‐Marie Klint Jørgensen contributed to the writing and revising of this protocol. The search strategy was developed by B. C. A. V. and Anne‐Marie Klint Jørgensen. All authors contributed to the writing of the review. The following review team assistants provided valuable help with screening and coding: Christiane Præstgaard Christensen, Ole Gregersen, Astrid Broni Heinemeier, Freja Jørgensen, Ida Lykke Kristiansen, Erika Lundqvist, Julie Schou Nicolajsen, Vivian Poulsen, Ida Scheel Rasmussen, Tróndur Møller Sandoy, Julie Kaas Seerup, Ida Skytt, Mette Trane, Mai Tødsø Jensen and Amanda Weber.

## SOURCES OF SUPPORT

VIVE—The Danish Center for Social Science Research/SFI Campbell.

## DECLARATIONS OF INTEREST

Three of the authors were involved in a previous review on a related topic: the effects of interventions targeting low SES students (Dietrichson et al., 2017). The authors have no vested interest in the outcomes of this review, nor any incentive to represent findings in a biased manner.

## PLANS FOR UPDATING THE REVIEW

Jens Dietrichson will be responsible for updating this review, as new studies and additional funding becomes available.

## Supporting information

Supplementary information

## Footnotes

^1^
Hereditary factors do not seem like a major explanation for the achievement gap between at‐risk and not‐at risk groups, see e.g., Hackman and Farah (2009), Nisbett et al. (2012), and Tucker‐Drob et al. (2013) for discussions.

^2^
Authors of reviews mentioned in that section but published after our search were not contacted.

^3^
Searches on Google Scholar were performed using the free software Publish or Perish (https://www.harzing.com/resources/publish‐or‐perish). Publish or Perish can bundle‐export references in bibliographical formats. This was utilised to quickly identify which references that could be classified as grey literature. Searches on Google Scholar were limited to the first 150 hits.

^4^
Note that these are not necessarily unique study populations, as some studies with more than one intervention group used only one control group.

^5^
There was one study from France among the comparison designs (see Appendix Table A2), one study lacking the information needed to calculate an effect size was performed in the Netherlands (see Appendix Table A3), and there were four studies from Australia, one from Germany, one from Israel, one from New Zealand, one from Spain, and one from South Korea among the studies given a too high risk of bias rating (see Appendix Table A5).

^6^
We calculated the number of student observations by taking the sample size for each effect size, which is equal to the number of students in the intervention and control group for which the effect size is calculated, and summing over effect sizes. This is *not* the same as the number of participants, as participants take more than one test in most studies, and control groups are sometimes compared to more than one intervention group. In these cases, participants contribute to more than one effect size. The estimations are based on effect sizes and their standard errors, and therefore indirectly on the number of student observations, not on the number of participants.

^7^
A forest plot on the effect size level displayed a similar looking distribution, but was difficult to read due to the large number of effect sizes and we therefore omitted this figure.

^8^
The short‐run results were not sensitive either to using study level average effect sizes and estimating the between‐study variance with the DL instead of using RVE.

^9^
The number of clusters, effect sizes, and student observations in the respective figure are 25, 36 and 14,961 in Figure 8; 51, 158 and 64,230 in Figure 9; 49, 135 and 50,499 in Figure 10 and 17, 59 and 28,692 in Figure 11. These numbers are the same in all sensitivity analyses shown in the figures.

^10^
The number of clusters, effect sizes, and student observations with outliers removed are 23, 31 and 14,717 for math tests; 51, 156 and 64,150 for reading tests; 49, 130 and 50,360 for RCTs and 16, 57 and 28,507 for QES.

^11^
The average effect sizes become larger, despite that adjusting for clustering makes every adjusted effect size smaller. The reason is that the weighting of effect sizes changes as well: individually randomised effect sizes, which, in our sample, are slightly larger than cluster‐randomised effect sizes, receive more weight when we adjust for clustering.

^12^
Note that for effect sizes based on mathematics tests and QES, the small sample corrected degrees of freedom in the RVE procedure are sometimes below the level where the procedure starts to perform poorly (Tanner‐Smith & Tipton, 2014; Tipton, 2015). The estimates are however so small and far from significance that it seems highly unlikely that this would affect our interpretations of the results.

^13^
For Egger's test, we used the Stata command *metabias* and study level effect sizes and standard errors (the command does not perform cluster‐robust variance estimation). The number of studies was 69 and the number of student observations included in these studies was 33,772 (i.e., we included all studies except one that only provided follow‐up effect sizes and one with an overlapping sample). When we adjusted for outliers, the same numbers were 65 and 33,547, respectively. The number of clusters, effect sizes, and student observations in the estimation testing whether journal publications have larger effect sizes was estimations are 66, 194 and 79,191, respectively.

^14^
Flynn, Zheng, et al. (2012) included 11 studies of the analysis of decoding and word identification, six of comprehension, and three of fluency. Edmonds et al. (2009) included seven studies of comprehension in their analysis.

^15^
Note though that social‐emotional skills and other noncognitive skills influence test scores as well (e.g., Kautz, Heckman, Diris, ter Weel, & Borghans, 2014), so there is reason to expect effects of interventions targeting these skills also on standardised tests.
