# Supplementary material for: Targeted school‐based interventions for improving reading and mathematics for students with, or at risk of, academic difficulties in Grades 7–12: A systematic review
Source: Campbell Syst Rev. 2020 Apr 1;16(2):e1081. doi: 10.1002/cl2.1081 (PMC8356291; doi:10.1002/cl2.1081)
Supplement: Supplementary file 1 — Supplementary information [file CL2-16-e1081-s001.docx]

Cover page

| Title | Targeted school-based interventions for improving reading and mathematics for students with, or at risk of, academic difficulties in Grades 7 to 12: A systematic review |
| --- | --- |
| Authors | Jens Dietrichson, Trine Filges, Rasmus H. Klokker, Bjørn C. A. Viinholt, Martin Bøg, Ulla H. Jensen |
| Protocol link | https://onlinelibrary.wiley.com/doi/10.1002/CL2.165 |
| Corresponding author | jsd@vive.dk |
|  |  |

Colophon

| **Title** |  | Targeted school-based interventions for improving reading and mathematics for students with or at risk of academic difficulties in grade 7 to 12: A systematic review |
| --- | --- | --- |
| **Institution** |  | The Campbell Collaboration |
| **Authors** |  | Dietrichson, Jens  Filges, Trine  Klokker, Rasmus H.  Viinholt, Bjørn C. A.  Bøg, Martin  Jensen, Ulla H. |
| **DOI** |  | 10.4073/csr.200x.x [delete this text: check number in separate list] |
| **No. of pages** |  | 231 |
| **Last updated** |  | July 2018 |
| **Citation** |  | Dietrichson J, Filges T, Klokker RH, Viinholt, BCA, Bøg M, & Jensen UH. Targeted school-based interventions for improving reading and mathematics for students with or at risk of academic difficulties in grade 7 to 12: A systematic review. Campbell Systematic Reviews 20xx:x DOI: 10.4073/csr.200x.x |
| **ISSN** |  | 1891-1803 |
| **Copyright** |  | © Dietrichson et al. This is an open-access article distributed under the terms of the Creative Commons Attribution License, which permits unrestricted use, distribution, and reproduction in any medium, provided the original author and source are credited. |
| **Roles and responsibilities** |  | Dietrichson, Filges, Bøg and Anne-Marie Klint Jørgensen contributed to the writing and revising of this protocol. The search strategy was developed by Viinholt and Anne-Marie Klint Jørgensen. All authors contributed to the writing of the review. The following review team assistants provided valuable help with screening and coding: Christiane Præstgaard Christensen, Ole Gregersen, Astrid Broni Heinemeier, Freja Jørgensen, Ida Lykke Kristiansen, Erika Lundqvist, Julie Schou Nicolajsen, Vivian Poulsen, Ida Scheel Rasmussen, Tróndur Møller Sandoy, Julie Kaas Seerup, Ida Skytt, Mette Trane, Mai Tødsø Jensen, and Amanda Weber. Jens Dietrichson will be responsible for updating this review as additional evidence accumulates and as funding becomes available. |
| **Editors for this review** |  | Editor: Managing editor: |
| **Sources of support** |  | VIVE–The Danish Center for Social Science Research/SFI Campbell |
| **Declarations of interest** |  | Three of the authors were involved in a previous review on a related topic: the effects of interventions targeting students from families with low socioeconomic status (Dietrichson, Bøg, Filges, & Klint Jørgensen, 2017). The authors have no vested interest in the outcomes of this review, nor any incentive to represent findings in a biased manner. |
| **Corresponding author** |  | Jens Dietrichson VIVE-The Danish Center for Social Science Research Herluf Trollesgade 11 DK-1052 Copenhagen K, Denmark E-mail: jsd@vive.dk  Full list of author information is available at the end of the article |

Campbell Systematic Reviews

| **Editor-in-Chief** |  | Vivian Welch, University of Ottawa, Canada |
| --- | --- | --- |
| **Editors** |  |  |
| *Crime and Justice* |  | David B. Wilson, George Mason University, USA Charlotte Gill, George Mason University, USA  Angela Higginson, Queensland University of Technology, Australia |
| *Disability* |  | Carlton J. Fong, Texas State University, USA |
| *Education* |  | Sarah Miller, Queen’s University Belfast, UK |
| *International Development* |  | Birte Snilstveit, 3ie, UK Hugh Waddington, 3ie, UK |
| *Social Welfare* |  | Brandy Maynard, Saint Louis University, USA |
| *Knowledge Translation and Implementation* |  | Aron Shlonsky, University of Melbourne, Australia |
| *Methods* |  | Therese Pigott, Loyola University, USA Ryan Williams, AIR, USA |
| **Managing Editor** |  | Chui Hsia Yong, The Campbell Collaboration |
| **Co-Chairs** |  |  |
| *Crime and Justice* |  | David B. Wilson, George Mason University, USA Peter Neyroud, Cambridge University, UK |
| *Disability* |  | Oliver Wendt, Purdue University, USA Joann Starks, AIR, USA |
| *Education* |  | Sarah Miller, Queen's University Belfast, UK Gary W. Ritter, University of Arkansas, USA |
| *Social Welfare* |  | Brandy Maynard, Saint Louis University, USA |
| *Knowledge Translation and Implementation* |  | Robyn Mildon, CEI, Australia Cindy Cai, AIR, USA |
| *International Development* |  | Peter Tugwell, University of Ottawa, Canada Hugh Waddington, 3ie, UK |
| *Methods* |  | Ariel Aloe, University of Iowa, USA |
|  |  | The Campbell Collaboration was founded on the principle that systematic reviews on the effects of interventions will inform and help improve policy and services. Campbell offers editorial and methodological support to review authors throughout the process of producing a systematic review. A number of Campbell’s editors, librarians, methodologists and external peer reviewers contribute. |
|  |  | The Campbell Collaboration P.O. Box 4404 Nydalen 0403 Oslo, Norway [www.campbellcollaboration.org](http://www.campbellcollaboration.org) |

# Table of contents

Table of contents 5

Appendix 6

Coding Scheme 6

Characteristics of Included Studies 8

Search Strategy by Database 35

Risk of Bias Tables 60

# Appendix

## Coding Scheme

The following information was extracted from all studies included in the meta-analysis:

**1. Report Characteristics**

1.1. Authors

1.2. Title

1.3. Publication year

1.4 Language

1.5 Publishing status (published in scientific journal or not)

1.6. Outlet/Type of publication (e.g., journal name/report series/dissertation)

**2. Study Characteristics**

2.1. Study location (country and state/city)

2.2. Study design (RCT, QRCT, QES)

2.3. Describe treatment assignment

2.4. Number of separate sites included in the study (classrooms, schools, districts, states or regions, countries)

2.5 If multiple sites, describe if there were differences in assignment between sites?

**3. Participant Characteristics**

3.1. Describe the target group of the intervention, e.g. students with specific learning difficulties, low achievement, low SES etc.

3.2. Gender (share of girls)

3.3. Age distribution (min, max, mean)

3.4. Grade distribution (min, max, mean)

3.5. Ethnic, cultural, and language background (share of students with a different

background than the majority student population)

3.6. Socioeconomic status (share low income, share low parental education, share low status parental occupation)

**4. Intervention Characteristics**

4.1. Name of intervention

4.2. Describe the instructional methods used in the intervention and any differences between intervention and control groups regarding these methods. State explicitly if there are no differences.

4.3. Describe the content domain targeted by the intervention and any differences between treatment and control groups regarding the content they are instructed in. State explicitly if there are no differences.

4.4. Intervention site (school/other, in which case, specify)

4.5. Group size (e.g. 1:1, 1:2, …)

4.6. Intervention implementer (Describe the person(s) delivering the intervention: e.g., teachers, college students, researchers).

4.7. Is the implementer trained, and for how long? (the number of hours/days/weeks).

4.8. Duration of intervention in weeks (intended, received)

4.9. Frequency of intervention in sessions (intended, received)

4.10. Intended intensity of intervention in hours per week (intended, received)

4.11. Implementation quality (questions from Wilson, Lipsey, Tanner-Smith, Huang, & Steinka-Fry, 2010): Was the implementation of the programme monitored by the author/researcher or programme personnel to assess whether it was delivered as intended? (Yes/No/Cannot tell)

4.13. Based on evidence or author acknowledgment, was there any uncontrolled variation or degradation in implementation or delivery of treatment, e.g., high dropouts, erratic attendance, treatment not delivered as intended, wide differences between settings or individual providers, etc.? Assume that there is no problem if one is not specified (yes (describe below)/ possible (describe below)/ no, apparently implemented as intended)

4.14. Describe implementation problems, if any.

**5. Control/Comparison Characteristics**

5.1. What is the nature of the control/comparison condition? (Controls do not receive any intervention/treatment/service after the end of intervention; Controls are wait-list controls; Comparison intervention)

**6. Outcome Measurement (each step is repeated for all relevant outcomes)**

6.1. Measurement timing (number of months after end of intervention)

6.2. Name of standardised test

6.3. Subject of standardised test (mathematics, reading)

6.4. Content domain(s) of test (e.g., vocabulary, algebra, general etc)

6.5. Who performs the tests?

**7. Sample Size**

7.1. Sample size used in analysis for outcome measurement (separate for intervention and control groups)

**8. Outcomes**

8.1. Test score (Code for both pre- and post-intervention tests separate for intervention and control groups if possible. Describe test in terms: continuous/dichotomous; whether high score/1 is desirable; type of statistic, e.g., mean, t-test, beta-coefficient, F-test etc)

8.2. Standard deviation (Code for both pre- and post-intervention tests separate for

intervention and control groups if possible)

8.3. Estimation method (e.g. raw means, adjusted means, regression adjusted etc)

8.4. Uncommon standardisation (No/Mention method of standardisation if available standard deviation is other than the raw standard deviation for the intervention and control group)

## Characteristics of Included Studies

**Studies Included in the Meta-Analysis**

The below Table A1 provides a short description of the methods, participants, interventions, and outcome measures for studies included in the meta-analysis. Note that participants and information about their characteristics refer to the group of students for which we have calculated effect sizes, which is not necessarily all participants in a study. The number of participants was calculated as the average by intervention over included effect sizes. In a few studies, students in grades that were not included in this review or students that were not at-risk were also given an intervention. Similarly, many studies used other outcomes alongside standardised tests in reading and mathematics but we have only listed the tests used to calculate effect sizes below. The number of participants in intervention and control groups is the average number that has taken these tests.

Table A1. Characteristics of included studies.

Allinder et al. (2001)

| **Methods** | Randomised controlled trial. |
| --- | --- |
| **Participants** | At-risk students and students with learning disabilities (speech-language impairment primarily) in grade 7. Intervention group: 33 students. Control group: 16 students. |
| **Interventions** | Students get instruction in groups of 11-20 students in fluency strategies, e.g., reading with inflection, not adding words, pausing at periods and commas, and self-monitoring for accuracy. Strategies are matched to students' reading problems, and instruction also involves comprehension, phonics skills, and oral reading. |
| **Outcomes** | Woodcock Reading Mastery Test – Revised; subtests Word identification, Word attack, and Passage comprehension. |

*Bark & Brooks (2016)*

| **Methods** | Quasi-experimental study. |
| --- | --- |
| **Participants** | Students in grade 7 with weak literacy skills: spelling age below 10, reading age below 9.4 or spelling below a standardized score below 90. Intervention group: 16 students. Control group: 16 students. |
| **Interventions** | Use an individual diagnostic assessment of literacy attainment and cognitive skills to develop an individual education plan, which is reviewed every 6 week. Teachers designed the intervention around the specific needs of each student using one-to-one teaching situation. Some focused more on language skills, some on writing skills, some on reading skills, and some on spelling. There was also emphasis on improving meta-cognitive skills, independent learning, and academic self-esteem. |
| **Outcomes** | Test of Word Reading Achievement, subtest Word Reading Efficiency, and Wide Range Achievement Test, subtests Spelling and Single Word Reading. |

*Barrow et al. (2009)*

| **Methods** | Randomised controlled trial. |
| --- | --- |
| **Participants** | Minority and poor students in grade 8 and 10. Intervention and control groups are in three districts: District 1: 227 in the intervention group and 227 in the control group. District 2: 171 in the intervention group and 171 in the control group. District 3: 100 in the intervention group and 100 in the control group. |
| **Interventions** | Computer-assisted instruction with I Can Learn, which included a classroom management tool for educators and on-site support for administrators and teachers. Each student’s performance is recorded in a grade book and teachers can monitor students’ progress through a series of reports. The programme targeted pre-algebra and algebra. |
| **Outcomes** | Different statewide tests. |

*Beattie (2000)*

| **Methods** | Quasi-experimental study. |
| --- | --- |
| **Participants** | Participants are in grades 6 to 8 and have a significant deficit in language learning as defined by performance in the bottom quartile on a standardised spoken language or reading test. Four different intervention groups - FF/FF: 12 students, FF/SM: 15 students, SM/FF: 11 students, SM/SM: 14 students. One control group: 12 students. |
| **Interventions** | Computer-assisted instruction with either Fast ForWord (FF) or Successmaker (SM). Both include a motivational component: A token economy system was employed as a component of the intervention programmes, where participants earned points which were subsequently exchanged for reinforcers such as age-appropriate toys and games, gift certificates for CDs, video rentals, or bowling games. FF targeted auditory processing, auditory memory, phonological analysis, phonemic awareness and grammar. SM targeted foundations in basic reading skills, study skills, content-area reading strategies, and higher-order thinking. It also included a wide range of literature-based activities focus on building vocabulary and comprehension. |
| **Outcomes** | Gray Oral Reading Test-Third Edition, subtest Oral Reading Quotient; Woodcock-Johnson, subtest Letter-Word Identification, Word Attack, Passage Comprehension, Phonemic Awareness; Wide Range Achievement Test, subtest Spelling. |

*Bhat et al. (2003)*

| **Methods** | Quasi-experimental study. |
| --- | --- |
| **Participants** | Middle school students in grade 6 to 8 with learning disabilities, identified as having phonological awareness deficits. Intervention group: 20 students. Control group: 20 students. |
| **Interventions** | Instruction in phonological awareness skills was provided on a one-on-one basis for students to a treatment and a waitlist control group. Instruction was based on Great Leaps Reading programme plus added lessons about blending two words to make a new word, segmenting a word to make two words, phoneme reversal, and phoneme substitution. |
| **Outcomes** | Woodcock Reading Mastery Test - Revised (WRMT-R), subtest Word identification; Comprehensive Test of Phonological Processes, a total score calculated from subtests of elision, blending words, nonword repetition, phoneme reversal, blending nonwords, segmenting words, and segmenting nonwords. |

*Bhattacharya et al. (2004)*

| **Methods** | Randomised controlled trial. |
| --- | --- |
| **Participants** | Students in grade 6 to 10 with below-average word-reading skills. Two intervention groups: 20 students in each intervention. One control group: 20 students. |
| **Interventions** | Instruction was provided as one-on-one tutoring by a teacher. The intervention targeted analysis of graphosyllabic constituent and whole-word reading. |
| **Outcomes** | Woodcock Reading Mastery Test – Revised, subtest Word Attack. |

*Borman et al. (2009)*

| **Methods** | Randomised controlled trial. |
| --- | --- |
| **Participants** | Predominantly African American and low socioeconomic status students, all in grade 7, who performed below national averages on norm-referenced reading achievement tests. Intervention group: 139 students. Control group: 135 students. |
| **Interventions** | Computer-assisted instruction with Fast ForWord. The programmes’ mathematical algorithms change many of the features of oral language, including volume, pitch, and duration. The exercises are provided in a game-like computerized environment, with animations to help maintain the child’s interest. On-screen rewards for successful completion of training segments are supplemented with token economy rewards, which are awarded for achievement of point goals determined in conjunction with the participant. Data from the child’s daily exercises are uploaded and weekly reports are generated to summarize the child’s progress. Fast ForWord typically supplemented the regular classroom literacy instruction. |
| **Outcomes** | Form A of Comprehensive Test of Basic Skills, Fifth Edition, a test of reading and language. |

*Bosnjak et al. (2017)*

| **Methods** | Randomised controlled trial. |
| --- | --- |
| **Participants** | Students in grade 9 with inadequate levels of scholastic and behavioural performance not deemed commensurate with expected levels. Intervention group: 8 students. Control group: 8 students. |
| **Interventions** | Instruction in small groups (6-10 students) using principles from cognitive behavioural therapy to help students improve meta-cognitive skills through, e.g., the establishment of links between thoughts, feelings, and actions, understanding the impact of negative and positive thoughts on learning, utilising skills as thought-stopping, and positive self-talk to achieve change. |
| **Outcomes** | Wechsler Individual Achievement Test, math and reading. |

*Boster et al. (2005)*

| **Methods** | Randomised controlled trial. |
| --- | --- |
| **Participants** | Students in grade 7 performing one to two years below grade level. Intervention group: 139 students. Control group: 167 students. |
| **Interventions** | Computer-assisted instruction through The New Century Integrated Instructional System that provides immediate feedback at multiple levels: cues to lesson questions, confirmation of correct responses, progress updates following each lesson, and a continuous motivational point system with award certificates for gains achieved. New Century lessons incorporated substantial audio as feedback, instruction, and support to students. The intervention targeted general math skills. |
| **Outcomes** | California Standards Test, math. |

*Boyle (1996)*

| **Methods** | Randomised controlled trial. |
| --- | --- |
| **Participants** | Low performing students in grade 6 to 8 with mild disabilities or educable mental retardation. Intervention group: 15 students. Control group: 15 students. |
| **Interventions** | Each classroom was staffed with a full-time teacher and a paraprofessional, both of whom were present throughout the study. The cognitive mapping strategy incorporated the use of the mnemonic "TRAVEL", which taught students, step by step, how to construct cognitive maps for use during reading. The control group was administered the same pretests, posttests, and reading passages, but did not receive training on the cognitive mapping strategy. While students in the experimental group received strategy instruction sessions, students in the control group remained in their classes and worked on separate reading assignments. The intervention targeted reading comprehension. |
| **Outcomes** | Stanford Diagnostic Reading Test. |

*Briggs (1996)*

| **Methods** | Quasi-experimental study. |
| --- | --- |
| **Participants** | Students in grade 7 that scored 1.5 or more years below grade level on the Gates-MacGinitie comprehension scale. Intervention group: 37 students. Control group: 37 students. |
| **Interventions** | An audio-visual approach using read-along tapes and walkmans that the students can use individually. The walkmans are provided to students and they can therefore practice also when they are not in class. The intervention targeted vocabulary and comprehension. |
| **Outcomes** | Gates-McGinitie Reading Tests, subtests vocabulary and comprehension. |

*Caggiano (2007)*

| **Methods** | Quasi-experimental study. |
| --- | --- |
| **Participants** | Struggling adolescent readers. Grade 7: 20 students in intervention group, and 20 students in control group. Grade 8: 20 students in intervention group, and 20 students in control group. |
| **Interventions** | The intervention, the READ 180 programme, incorporated a software component, best literacy practices, and the Lexile levelling system. The instructional model for READ 180 is comprised of a 90-minute time block, divided into several rotations: it begins with a 20-minute whole group activity, such as a read aloud, a vocabulary lesson, or a team-building exercise. Then there is a small group activity where students rotate among three 20-minute stations. The first rotation is the workshop, where students receive direct instruction by a teacher in a small group setting. A second station is the computer. This rotation requires students to work independently, interacting with the programme’s software. Upon successful completion of the three instructional zones, a formative evaluation is administered which provides feedback to the instructor. The programme was used in addition to regular instruction in language arts. READ 180 targeted comprehension, vocabulary and spelling. |
| **Outcomes** | Scholastic Reading Inventory (reading comprehension); Virginia Standards of Learning Assessment in Reading. |

*Calhoon & Fuchs (2003)*

| **Methods** | Randomised controlled trial. |
| --- | --- |
| **Participants** | Students in grade 9 to 12 with a learning disability in mathematics. Some have behavioral and mental disorders, and are far behind grade level in math. Intervention group: 39 students. Control group: 41 students. |
| **Interventions** | Classwide peer-tutoring coupled with weekly CBM feedback. The intervention also included a reinforcement system that provided tangible rewards, such as money, tickets donated from local sports teams, and certificates from local restaurants. Intervention focuses on computation skills and concepts/applications. |
| **Outcomes** | Tennessee Comprehensive Achievement Test; Math Operations Test–Revised; Math Concepts and Applications Test. |

*Campuzano et al. (2009)*

| **Methods** | Randomised controlled trial. |
| --- | --- |
| **Participants** | Students in grade 9 from low-income families. Cognitive Tutor Algebra I: 440 in intervention group and 315 in control group. Larson Algebra I: 648 students in intervention group and 556 in control group. |
| **Interventions** | Two computer-assisted instruction programmes, both accompanied by a textbook. Cognitive Tutor Algebra I: The product presents problems in scenarios, asks students to use graphs to represent problems related to the scenarios, and asks the students to use a solver to answer questions related to the scenarios. It also evaluates students’ skill levels based on their answers. The product provides teachers with reports on student progress and performance. Focuses on proportional reasoning, solving linear equations and inequalities, solving systems of linear equations, analysing data, and using polynomial functions, powers, and exponents. Larson Algebra I: published by Houghton-Mifflin and designed to supplement the curriculum with extra instruction, practice, and assessments. The programme addresses both skill building and problem solving, and allows the teacher to track student progress. The intervention covers whole numbers, fractions, decimals, percents, rational numbers, probability and statistics, coordinate geometry, pre-algebra, and algebra I. |
| **Outcomes** | Educational Testing Services End-of-Course Algebra, subtest Algebra I. |

*Cantrell et al. (2010)*

| **Methods** | Randomised controlled trial. |
| --- | --- |
| **Participants** | Adolescent struggling readers in grade 9 who score the equivalent of two grade levels below grade level on the study pretest, the Group Reading and Diagnostic Evaluation. Intervention group: 194 students. Control group: 159 students. |
| **Interventions** | The learning strategies curriculum intervention is divided into three strands: acquisition, storage, and expression. Each strand includes the teaching of a number of strategies designed to help students derive information from texts, identify and remember important information, or develop writing or academic competence. Each strategy is taught through eight instructional stages: pretest and commitments, describe, model, verbal practice, controlled practice and feedback, posttest and commitments, and generalization. The intervention implemented in this study included all three strands. Students in this study were taught the strategies of word identification, visual imagery, self-questioning, paraphrasing, and sentence writing. |
| **Outcomes** | Group Reading Assessment and Diagnostic Evaluation. |

*Cantrell et al. (2016)*

| **Methods** | Randomised controlled trial. |
| --- | --- |
| **Participants** | Adolescents in grade 9 with learning disabilities receiving instruction in general education classrooms. Intervention group: 593 students. Control group: 535 students. |
| **Interventions** | The learning strategies curriculum intervention is divided into three strands: acquisition, storage, and expression. Each strand included the teaching of a number of strategies designed to help students derive information from texts, identify and remember important information, or develop writing or academic competence. Each strategy was taught through eight instructional stages: pretest and commitments; describe; model; verbal practice; controlled practice; feedback; posttest and commitments; and generalization. Teachers’ implementation was supported by coaches who visited teachers’ classrooms to observe, model, and help teachers solve problems. Further support was provided through telephone calls, email correspondence, and more formal distance support. |
| **Outcomes** | Group Reading Assessment and Diagnostic Evaluation. |

*Caudell (2016)*

| **Methods** | Randomised controlled trial. |
| --- | --- |
| **Participants** | Students in grade 11 who had failed at least one of the Georgia High School Graduation Tests. Intervention group: 22 (math) and 9 (reading) students. Control group: 10 (math) and 11 (reading) students. |
| **Interventions** | Two interventions, one more focused on math and one more on reading. Student Success Skills program with small group counselling sessions (6-8 students) were a part of both interventions. The program focuses on meta-cognitive, social, and self-management skills. |
| **Outcomes** | Georgia High School Graduation Tests, math and reading. |

*Cleary et al. (2017)*

| **Methods** | Randomised controlled trial. |
| --- | --- |
| **Participants** | Students in grade 7 and 8 with low math performance and deficiencies in motivation and regulation. Intervention group: 21 students. Control group: 16 students. |
| **Interventions** | SREP coaches provide individualized feedback to: (a) increase student awareness about why they are struggling in a particular class, (b) enhance their knowledge and skills in using strategies to remedy these problems, and (c) adapt and refine these strategies as they attempt to improve or make progress in school. Because SREP is typically administered in small group formats, peer-mediated learning experiences are naturally emphasized. The majority of the sessions focused on developing students’ self-regulated learning, strategic thinking and action rather than remediation of specific mathematics skills. |
| **Outcomes** | New Jersey Assessment of Skills and Knowledge. |

*Cook et al. (2014)*

| **Methods** | Randomised controlled trial. |
| --- | --- |
| **Participants** | Disadvantaged boys in grade 9 and 10 who are falling behind and are at great risk of dropping out. Intervention group: 57 students. Control group: 24 students. |
| **Interventions** | Studied tutoring and social-cognitive skills training with one intervention group receiving both, and one intervention group receiving just social-cognitive skills training. However, tutoring spilled over to the social-cognitive group, and the authors treat them as one in much of the analysis. BAM is in-school programming that exposes youth to pro-social adults, and provides them with social-cognitive skills training that follows the principles of cognitive behavioral therapy. The control group receives treatment as usual, which included approximately 21 hours of writing tutoring and 20 hours of math tutoring per year, much less than the intervention groups. |
| **Outcomes** | EXPLORE; PLAN |

*Corrin et al. (2008)*

| **Methods** | Randomised controlled trial. |
| --- | --- |
| **Participants** | Students in grade 9 that score between two and five years below grade level on reading comprehension tests. RAAL: 645 students in intervention group, 470 students in control group. Xtreme Reading: 619 students in intervention group, 437 students in control group. |
| **Interventions** | The interventions were two reading programmes: Reading Apprenticeship Academic Literacy (RAAL) and Xtreme Reading. RAAL: Instructors usually used one or two of the following routines during a class period: 1. Think aloud. 2. Talking to the text. 3. Metacognitive logs/journals. 4. Preambles (daily warm-ups). Xtreme reading: Each strategy is taught using a prescribed eight-stage instructional methodology: 1. Describe. 2. Model. 3. Verbal practice. 4. Guided practice. 5. Paired practice. 6. Independent practice. 7. Differentiated instruction. 8. Integration and generalization. Content domains included: (1) student motivation and engagement; (2) reading fluency, or the ability to read quickly, accurately, and with appropriate expression; (3) vocabulary, or word knowledge; (4) comprehension, or making meaning from text; (5) phonics and phonemic awareness (for students who could still benefit from instruction in these areas); and (6) writing. |
| **Outcomes** | Group Reading Assessment and Diagnostic Examination, subtests reading comprehension and vocabulary test scores. |
| **Notes** | Kemple et al. (2009) and Somers et al. (2010) study the same interventions and we have used information from all three studies during coding. |

*Corsello & Sharma (2015)*

| **Methods** | Randomised controlled trial. |
| --- | --- |
| **Participants** | Students in grade 9 that score below median on at least one out of two pre-intervention tests. Intervention group: 159 students. Control group: 159 students. |
| **Interventions** | In the BARR model, students take three core courses. The model integrates eight strategies to provide a structure that helps educators build safe, strong, trusting relationships with their students, and for students to engage in learning. The model works with a school's existing curriculum and existing staff. The eight strategies are: 1. Focus on the whole student. 2. Provide professional development for staff, including coaching. 3. Time Classroom Curriculum to foster learning. 4. Create cohorts of students. 5. Hold regular teacher team meetings. 6. Conduct Risk Review meetings. 7. Engage families in student learning. 8. Engage administration. |
| **Outcomes** | Measures of Academic Progress (MAP) |

*Denton et al. (2008)*

| **Methods** | Randomised controlled trial. |
| --- | --- |
| **Participants** | Students in grade 6 to 8 with severe reading difficulties. Intervention group: 20 students. Control group: 18 students. |
| **Interventions** | Through tutoring the intervention targets word-level reading skills, comprehension, vocabulary and fluency. The intervention was designed to be individualized, rather than highly prescriptive, to be responsive to student needs. Soon after the onset of intervention, in response to students’ severe word-level reading deficits, the decision was made to place heavy emphasis on phonics, word recognition, spelling, and fluency. |
| **Outcomes** | Woodcock–Johnson Tests of Achievement III, subtests Passage Comprehension, Word Attack, Letter-Word identification (these two are combined into one score, called Basic Reading Skills); Test of Word Reading Efficiency, subtest Sight Words; DIBELS, subtest Oral Reading Fluency |

*Early (1998)*

| **Methods** | Quasi-experimental study. |
| --- | --- |
| **Participants** | At-risk students in grade 10 defined by their result on the 8th grade Texas Assessment of Academic Skills. Intervention group: 67 students. Control group: 78 students. |
| **Interventions** | The intervention provided peer-tutoring with the aim to improve results in mathematics on a statewide test, which incorporated several areas of mathematics. |
| **Outcomes** | Texas Assessment of Academic Skills. |

*Fogarty et al. (2014)*

| **Methods** | Randomised controlled trial. |
| --- | --- |
| **Participants** | Struggling readers in middle school, grade 6 to 8. Intervention group: 349 students. Control group: 387 students. |
| **Interventions** | The intervention, Comprehension Circuit Training (CCT), used cooperative learning and provided coaches to teachers. The students work with partners, first taking a quiz individually and then working in groups of three to four to complete the same quiz. The intervention targeted reading comprehension and vocabulary. |
| **Outcomes** | Gates-McGintie, subtest Comprehension. |

*Fogarty et al. (2017)*

| **Methods** | Randomised controlled trial. |
| --- | --- |
| **Participants** | Struggling readers in grade 6 to 8, previously assigned to reading intervention classes based on their failure to reach proficiency. Intervention group: 105 students. Control group: 97 students. |
| **Interventions** | Comprehension Circuit Training with electronic tablets. Students work with partners and teachers receive coaching and professional development. The program aim to improve reading comprehension and also trains vocabulary. |
| **Outcomes** | Gates-MacGinite, reading comprehension; Group Reading Assessment and Diagnostic Evaluation (GRADE), reading comprehension; Gray Oral Reading Test, passage comprehension; State of Texas Assessements of Academic Readiness; Test of Word Reading Efficiency, subtest sight word efficiency; Test of Silent Reading Efficiency and Comprehension, reading fluency and sentence comprehension. |

*Fryer (2011)*

| **Methods** | Randomised controlled trial. |
| --- | --- |
| **Participants** | Students in grade 7 to 9 in low-performing urban school districts. Chicago: 3255 students in intervention group 4353 students in control group. New York City: 4548 students in intervention group, 4638 students in control group. |
| **Interventions** | The intervention used financial incentives to improve performance in reading and mathematics, for example rewarding the students when they finish reading a book, do well on a test or get a high grade. |
| **Outcomes** | New York State Assessment; PLAN |

*Good et al. (2003)*

| **Methods** | Randomised controlled trial. |
| --- | --- |
| **Participants** | Students in grade 7 at risk of “stereotype threat”, e.g., girls in mathematics, ethnic minority and low-income students. There were three types of interventions, which in turn were tested in reading for minorities and low-income students, and in mathematics for girls. The group sizes for minorities and low-income students were 29 in the attribution intervention; 33 in the incremental intervention; 31 in the combined intervention; and 32 in the control group. The group sizes for girls were 13 in the attribution intervention; 14 in the incremental intervention; 13 in the combined intervention; and 13 in the control group. |
| **Interventions** | The interventions used college students as mentors and implemented three interventions: 1) an incremental condition, where students learn about intelligence and the brain, and how intelligence is an malleable quality; 2) an attributional condition, where mentors were asked to describe their own difficulty in making the adjustment to junior high but that they eventually overcame these difficulties; 3) a combined condition, where mentors delivered both messages. The control group received a “placebo” treatment, participants in this condition learned about the perils of drug use. |
| **Outcomes** | Texas Assessment of Academic Skills. |

*Haslam et al. (2006)*

| **Methods** | Quasi-experimental study. |
| --- | --- |
| **Participants** | Participants, in grade 7 and 8, were either Low English Proficiency (LEP) students, special education students, or students performing below grade level on measures of reading proficiency. Intervention group: 307 students. Control group: 307 students. |
| **Interventions** | The intervention used the programme READ 180. The READ 180 programme incorporates a software component, best literacy practices, and the Lexile levelling system. The instructional model for READ 180 is comprised of a 90-minute time block, divided into several rotations. READ 180 targets comprehension, vocabulary and spelling. Control group received no intervention. |
| **Outcomes** | Texas Assessment of Knowledge and Skills. |
| **Notes** | We used information about the READ 180 programme from other included studies about the same programme to code this study. |

*Hutchinson (1993)*

| **Methods** | Randomised controlled trial. |
| --- | --- |
| **Participants** | Learning disabled students in grade 8 to 10 scheduled for small group instruction in mathematics. Intervention group: 12 students. Control group: 8 students. |
| **Interventions** | Intervention and control groups were selected from students scheduled for small group instruction. The intervention targeted cognitive strategies in algebra word problem solving. Treated students met individually with instructor. The following: scripts were used to guide the instruction for treated students: orientation script, general script for word problems, and scripts specific to representation and solution for each problem type. There was an assessment task after each session that makes it possible to monitor progress. |
| **Outcomes** | British Columbia Mathematics Achievement Test (grade 7-8); Q2 British Columbia Achievement Test (Grade 10). |

*Jeffes (2013)*

| **Methods** | Randomised controlled trial. |
| --- | --- |
| **Participants** | The poorest word readers in grade 7 to 10 in their school year with a reading age 18 months or more behind their chronological age. Intervention group: 15 students. Control group: 15 students. |
| **Interventions** | The intervention, Toe By Toe, used one-to-one instruction, providing individual instruction to students tailored to their specific needs. Multi-sensory activities are included to enable students to focus on words they are really struggling with. The primary skills targeted by Toe By Toe are word recognition, phonic decoding accuracy and fluency. |
| **Outcomes** | York Assessment of Reading Comprehension-Secondary Test, subtests Word Recognition Accuracy, Passage Reading Fluency, Passage comprehension; Wechsler Individual Achievement Test, subtest Phonic Decoding Accuracy; Test of Word Reading Efficiency, subtests Sight Word Reading Fluency, Phonic Decoding Fluency. |

*Justus (2010)*

| **Methods** | Randomised controlled trial. |
| --- | --- |
| **Participants** | Students in grade 11 who were not predicted to meet the ACT college readiness benchmark for mathematics. Intervention group: 17 students. Control group: 17 students. |
| **Interventions** | The intervention was a curriculum-based ACT Math Intervention course, with the attached standards and description. It targeted general math skills, including ACT Test-taking Skills, Basic Arithmetic, Computations, Pre-Algebra, Algebra, Basic Geometry; Basic Probability; Graphs. |
| **Outcomes** | ACT |

*Kemple et al. (2008)*

| **Methods** | Randomised controlled trial. |
| --- | --- |
| **Participants** | Students entering ninth grade with reading skills that were between two and four years below grade level. RAAL: 686 students in intervention group, 454 students in control group. Xtreme Reading: 722 students in intervention group, 551 students in control group. |
| **Interventions** | The interventions were two reading programmes: Reading Apprenticeship Academic Literacy (RAAL) and Xtreme Reading. RAAL: Instructors usually used one or two of the following routines during a class period: 1. Think aloud. 2. Talking to the text. 3. Metacognitive logs/journals. 4. Preambles (daily warm-ups). Xtreme reading: Each strategy is taught using a prescribed eight-stage instructional methodology: 1. Describe. 2. Model. 3. Verbal practice. 4. Guided practice. 5. Paired practice. 6. Independent practice. 7. Differentiated instruction. 8. Integration and generalization. Content domains included: 1. student motivation and engagement; 2. reading fluency, or the ability to read quickly, accurately, and with appropriate expression; 3. vocabulary, or word knowledge; 4. comprehension, or making meaning from text; 5. phonics and phonemic awareness (for students who could still benefit from instruction in these areas); and 6. writing. |
| **Outcomes** | Group Reading Assessment and Diagnostic Examination (GRADE), subtests reading comprehension and vocabulary test scores |
| **Notes** | Corrin et al. (2009) and Somers et al. (2010) study the same interventions and we have used information from all three studies during coding. |

*Kempley (2005)*

| **Methods** | Randomised controlled trial. |
| --- | --- |
| **Participants** | Underperforming 7th grade math students. Intervention group: 23 students. Control group: 23 students. |
| **Interventions** | A two period-double math programme. The intervention group substituted their elective for a second period of math instruction. The control received their regular elective period. The intervention targeted several areas of mathematics: pre-teaching and re-teaching of content standards, and homework assistance by one of the 7th grade math instructors. |
| **Outcomes** | MDTP Pre-Algebra Readiness Test. |

*Kim (2002)*

| **Methods** | Randomised controlled trial. |
| --- | --- |
| **Participants** | High school students in grade 9 to 12 with learning disabilities. Intervention group: 14 students. Control group: 9 students. |
| **Interventions** | The intervention, Computer-Assisted Collaborative Strategic Reading (CACSR), focused on reading comprehension. CACSR provided individualized instruction, learner control, immediate feedback, the students’ performance or progress, and interactive and non-linear learning environment. Since CACSR has a function to record students’ learning paths and performances, a teacher can monitor and evaluate students’ performances. |
| **Outcomes** | Woodcock Reading Mastery Test–Revised, subtest Passage Comprehension |

*Kim et al. (2006)*

| **Methods** | Randomised controlled trial. |
| --- | --- |
| **Participants** | Middle school students in grade 6 to 8 with learning disabilities. Intervention group: 16 students. Control group: 18 students. |
| **Interventions** | Tested a similar CACSR intervention as Kim (2002), but with some added features, including a type of peer-assisted learning where students working in pairs. Computer assisted instruction, where students learned three steps for getting the gist: Identify who or what the paragraph is about, identify what the most important information is about the who or what, and write the gist in 10 words or less in a complete sentence. CACSR also has a built-in function that records students’ performance data. Then, at the beginning of each session, the instructor would spend 5 to 10 minutes discussing how to improve their responses and reminding them about the procedures for using the CACSR programme. The intervention targets reading comprehension. |
| **Outcomes** | Woodcock Reading Mastery Test–Revised, subtest Passage Comprehension. |

*Kim et al. (2011)*

| **Methods** | Quasi-experimental study. |
| --- | --- |
| **Participants** | Latino ELL’s in grade 6 to 11 scoring at or above intermediate on the CELDT. Intervention group: 1421 students. Control group: 1305 students. |
| **Interventions** | There are three core components of the Pathway Project: (1) training in the use of the cognitive strategies tool kit and curriculum materials with the help of student partners; (2) intervention activities focused on the revision of the pretest on-demand writing assessment into a multiple draft essay, and (3) coaching from a more experienced, veteran teacher previously trained in the Pathway Project on how to integrate a cognitive strategies approach into the existing English language arts curriculum. Components were implemented with a mix of direct instruction, individual assignments and coaching of students. The intervention targets reading, writing, cognitive and meta-cognitive strategies. |
| **Outcomes** | California Standards Test. |
| **Notes** | We used information about the programme from Olson et al. (2012), who study a second cohort that gets the programme, in the coding of this study. |

*Kotsopoulos (2008)*

| **Methods** | Quasi-experimental study. |
| --- | --- |
| **Participants** | Students in grade 9 with a learning disability, diagnosed through a psychoeducational assessment. Intervention group: 20 students. Control group: 6 students. |
| **Interventions** | The intervention uses cross-age peer-tutoring. Participants leave their GLE class to receive the programme. The PASS programme uses explicit instruction and a scaffolding approach to teach cognitive and metacognitive learning strategies in areas such as writing, reading comprehension, grammar, and math, as well as more general areas, such as organization and test-taking. |
| **Outcomes** | Wide Range Achievement Test, subtest Arithmetic. |

*Lemberger et al. (2015)*

| **Methods** | Randomised controlled trial. |
| --- | --- |
| **Participants** | Given to all in a school but targeting minority and poor students in grade 7. Intervention group: 111 students. Control group: 82 students. |
| **Interventions** | The Student Success Skills (SSS) is a school counselor-delivered intervention designed to support students by exposing them to select learning and personal–social skills that are considered important to school success. The programme includes a structured classroom guidance component and a small group (6-8) counseling component for students needing additional support. The small group component was however *not* used in this study. Classroom lessons are centered around skills organized into five categories: (a) cognitive factors (b) attitudinal skills (c) self-regulatory and metacognitive skills (d) behavioral strategies and (e) social skills. The intervention targets reading and mathematics. |
| **Outcomes** | Discovery Education Assessments. |

*Levitt et al. (2016)*

| **Methods** | Randomised controlled trial. |
| --- | --- |
| **Participants** | Low-income and minority students in grade 9. Intervention group: 750 students. Control group: 175 students. |
| **Interventions** | The intervention used monthly financial incentives. In order to qualify for the monthly reward, a student had to meet a monthly achievement standard for attendance, behavior, grades and test scores. Each month was independent so that students who did not qualify for a reward in one month could qualify for a reward the following month and vice versa. In a parent intervention, parents received the incentives; in the student intervention, students received the incentives. |
| **Outcomes** | Standardised school reading assessment (not named). |

*Little et al. (2014)*

| **Methods** | Randomised controlled trial. |
| --- | --- |
| **Participants** | Students from low income background with low passing rate on state tests. Grade 7: 448 students in intervention group, 310 students in control group. Grade 8: 416 students in intervention group, 336 students in control group. |
| **Interventions** | The intervention has two phases: 1) teachers exposed students in a large group to a variety of books, genres, and authors through short read-alouds and brief discussions. 2) teachers had students read independently in their self-selected, challenging books. While students read, teachers conducted individualized 5- to 7-min conferences with students, meeting with each student approximately once every 1 to 2 weeks. |
| **Outcomes** | Gates–MacGinitie Reading Tests, subtest Comprehension. |

*Lovett et al. (2012)*

| **Methods** | Quasi-experimental study. |
| --- | --- |
| **Participants** | Students in grade 9 who scored 1 standard deviation or more below age norm expectations on a averaged standard score obtained from three of four reading achievement tests. Intervention group: 268 students. Control group: 83 students. |
| **Interventions** | The intervention, the PHAST PACES programme, integrated word identification and text comprehension strategy instruction. The programme offered a decoding track (the PHAST Track) and two integrated comprehension tracks, the Text Knowledge and Comprehension Strategy Tracks. It also included metacognitive components, and small instructional groups that allowed for the inclusion of cooperative learning experiences. |
| **Outcomes** | Woodcock Reading Mastery Tests-Revised, subtests Word Identification, Word Attack, Passage Comprehension. |

*Lugo (2004)*

| **Methods** | Randomised controlled trial. |
| --- | --- |
| **Participants** | Students in grade 9 to 12 scoring 65% or lower on the pretest High School Subject Tests. Intervention group: 48 students. Control group: 42 students. |
| **Interventions** | The intervention used a multimedia mathematics windows-based software programme, Destination Mathematics, which targeted algebra. |
| **Outcomes** | High School Subject Tests: Algebra Form B. |

*Okkinga et al. (2018)*

| **Methods** | Randomised controlled trial. |
| --- | --- |
| **Participants** | Adolescent low achievers in pre-vocational education, grade 7. Intervention group: 168 students. Control group: 170 students. |
| **Interventions** | Reciprocal teaching program where students practice and discuss reading strategies with other students, guided and coached by the teacher. Teachers are also coached. There is also direct instruction from teachers in whole class. The program aims to improve reading comprehension. |
| **Outcomes** | SALT-reading, test of reading comprehension. |

*Olson et al. (2012)*

| **Methods** | Quasi-experimental study. |
| --- | --- |
| **Participants** | Schools in grade 6 to 11 who failed to meet state and federal accountability goals. Students were English language learners. Intervention group: 1492 students. Control group: 1463 students. |
| **Interventions** | There are three core components of the Pathway Project: (1) training in the use of the cognitive strategies tool kit and curriculum materials with help of student partners (2) intervention activities focused on the revision of the pretest on-demand writing assessment into a multiple draft essay, and (3) coaching from a more experienced, veteran teacher previously trained in the Pathway Project on how to integrate a cognitive strategies approach into the existing English language arts curriculum. Components were implemented with a mix of direct instruction, individual assignments and coaching of students. The intervention targets reading, writing, cognitive and meta-cognitive strategies. |
| **Outcomes** | California Standards Test. |

*Olson et al. (2015)*

| **Methods** | Quasi-experimental study. |
| --- | --- |
| **Participants** | Students in grade 10 who do not have English as their first language and minority students. Year 1: 313 students in intervention group, 262 students in control group. Year 2: 122 students in intervention group, 114 students in control group. |
| **Interventions** | There are three core components of the Pathway Project: (1) training in the use of the cognitive strategies tool kit and curriculum materials with the help of student partners, (2) intervention activities focused on the revision of the pretest on-demand writing assessment into a multiple draft essay, and (3) coaching from a more experienced, veteran teacher previously trained in the Pathway Project on how to integrate a cognitive strategies approach into the existing English language arts curriculum. Components were implemented with a mix of direct instruction, individual assignments and coaching of students. The intervention targets reading, writing, cognitive and meta-cognitive strategies. |
| **Outcomes** | California High School Exit Exam. |

*Papalewis (2004)*

| **Methods** | Quasi-experimental study. |
| --- | --- |
| **Participants** | Struggling readers in grade 8. Intervention group: 537 students. Control group: 536 students. |
| **Interventions** | The intervention uses the programme READ 180. The programme incorporates a software component, best literacy practices, and the Lexile levelling system. The instructional model for READ 180 is comprised of a 90-minute time block, divided into several rotations. READ 180 targets comprehension, vocabulary and spelling. The intervention furthermore used tutoring and small-group instruction, and teachers get detailed progress reports. |
| **Outcomes** | Stanford Achievement Test-9 |
| **Notes** | Information from other studies using the READ 180 programme (Haslam et al., 2006; Caggiano, 2007) was used to code this study. |

*Penney (2002)*

| **Methods** | Quasi-experimental study. |
| --- | --- |
| **Participants** | Poor readers in grade 11 to 12. Intervention group: 21 students. Control group: 12 students. |
| **Interventions** | The tutor was instructed to print one or two words with a consonant followed by the pattern (e.g., bad and sad) and to ask the student to read these words. If the student read a word correctly, the tutor simply said good or correct; if the student could not read the word, the tutor simply pronounced it. The tutor would then ask, “What do the letters a-d say?” or “How do you spell ad?” Again, a correct answer was rewarded; if the student gave no answer or an incorrect one, the tutor simply gave the answer. The student would be asked to pronounce and spell parts of the word such as sad or addle or ly. If the student was successful with two-syllable words, multisyllabic words were introduced. The intervention targeted processing the meaning of words, decoding, and phonemic awareness. |
| **Outcomes** | Woodcock Reading Mastery Test, subtests Word Identification, Word Attack and Passage Comprehension. |

*Prediger & Wessel (2018)*

| **Methods** | Randomised controlled trial. |
| --- | --- |
| **Participants** | Low-performing grade 7 students in mathematics. Intervention group: Diskursive förderung: 83 students, Lexikalische förderung: 103 students. Control group: 157 students. |
| **Interventions** | Supplementary instruction in small groups (3-5 students). Two versions of the intervention, both are focused on fractions but one adds integrated vocabulary training. |
| **Outcomes** | Unnamed standardized test in math. |

*Roberts et al. (2013)*

| **Methods** | Randomised controlled trial. |
| --- | --- |
| **Participants** | Struggling readers in grade 8. Intervention group: 99 students. Control group: 24 students. |
| **Interventions** | The intervention included three tiers (over a period of three years), with different components and different samples. The teachers participated in biweekly staff development meetings and received regular (once every one to two weeks) on-site feedback and coaching based on ongoing fidelity checks. Students received daily instruction and practice with individual letter sounds, letter combinations, and affixes, writing and spelling. The intervention targeted the following reading domains: word study, fluency, vocabulary, sentence and paragraph meaning, and overall comprehension. |
| **Outcomes** | Woodcock-Johnson III test of Achievement, subtests Letter-word identification, Word attack, Passage Comprehension. |

*Rossiter (2012)*

| **Methods** | Quasi-experimental study. |
| --- | --- |
| **Participants** | Ninth-graders who were extremely deficient in their mathematical skills (tests taken in tenth grade). Intervention group: 32 students. Control group: 120 students. |
| **Interventions** | The three components of the Math Recovery programme were: (1) direct instruction teaching mathematical skills that students were currently or will be using in a Algebra/Geometry I class; (2) peer-tutoring instruction related to the mathematics homework of the student; and (3) Auto-Skills computer programme. |
| **Outcomes** | Missouri Assessment Programme |

*Rutt et al. (2015)*

| **Methods** | Randomised controlled trial. |
| --- | --- |
| **Participants** | Struggling readers who are starting 7th grade. Intervention group: 286 students. Control group: 271 students. |
| **Interventions** | Structured one-to-one literacy intervention where students were pulled-out of ordinary classes. Targeted individual needs and selected an appropriate book through a bank of assessments for learning (these tests were not used to monitor progress). Intervention targets segmentation and blending of phonemes, and memorising letter names of high frequency words. |
| **Outcomes** | New Group Reading Test |

*Schüler-Meyer et al. (2019)*

| **Methods** | Randomised controlled trial. |
| --- | --- |
| **Participants** | German-Turkish bilingual students with low math achievement. Intervention group: Monolingual intervention: 44 students, Bilingual intervention: 41 students. Control group: 43 students. |
| **Interventions** | Two interventions, one in German and one in both German and Turkish. In both, instruction is performed in small groups (3-5 students) and aims to improve knowledge of fractions. |
| **Outcomes** | Unnamed standardized test in math. |

*Shell (1998)*

| **Methods** | Randomised controlled trial. |
| --- | --- |
| **Participants** | Students in grade 9 and 10 with high absence, low performance and a special education background with emotional disturbances or behavioural problems. MPSI: 32 students in intervention group. TRAD/C: 16 students in intervention group. Control group: 15 students. |
| **Interventions** | There are two interventions, Metacognitive Problem Solving Intervention (MPSI) and Traditional dropout prevention (TRAD/C). MPSI: Phase I is two weeks of teaching to recognize and apply each of the five stages of problem-solving to specific problematic-situations. Phase II stimulate subjects’ critical thinking skills through applied practice activities related to their own personal school problems. Phase III introduced subjects to personal goal-setting and building self-control skills. TRAD/C: The intervention was facilitated by the investigator using existing components of the school’s dropout prevention programme, which consisted of career/pre-vocational planning and academic tutorial support. Students learn metacognitive skills in MPSI, general academic and planning skills in TRAD/C. |
| **Outcomes** | Stanford Achievement Test. |

*Solís et al. (2015)*

| **Methods** | Randomised controlled trial. |
| --- | --- |
| **Participants** | Ninth graders with very low reading comprehension. Intervention group: 23 students. Control group: 18 students. |
| **Interventions** | The intervention used both adult-led small group instruction and peer-assisted learning. After the explicit instruction, students worked with partners to answer turn-and-talk questions, and discuss position statements. At the end of each unit, a CBM tool was administered to students in the intervention group to monitor progress. The intervention targets reading comprehension and vocabulary. |
| **Outcomes** | Test of Sentence Reading Efficiency (TOSRE); Woodcock-Johnson, subtest Passage Comprehension (WJ-III-PC) |

*Somers et al. (2010)*

| **Methods** | Randomised controlled trial. |
| --- | --- |
| **Participants** | Students entering ninth grade with reading skills that were between two and four years below grade level. RAAL: 676 students in intervention group, 495 students in control group. Xtreme Reading: 735 students in intervention group, 559 students in control group. |
| **Interventions** | The interventions were two reading programmes: Reading Apprenticeship Academic Literacy (RAAL) and Xtreme Reading. RAAL: Instructors usually used one or two of the following routines during class period: 1. Think aloud 2. Talking to the text 3. Metacognitive logs/journals 4. Preambles (daily warm-ups). Xtreme reading: Each strategy was taught using a prescribed eight-stage instructional methodology: 1. Describe 2. Model 3. Verbal practice 4. Guided practice 5. Paired practice 6. Independent practice 7. Differentiated instruction 8. Integration and generalization. The intervention targeted: 1. student motivation and engagement; 2. reading fluency, or the ability to read quickly, accurately, and with appropriate expression; 3. vocabulary, or word knowledge; 4. comprehension, or making meaning from text; 5. phonics and phonemic awareness (for students who could still benefit from instruction in these areas); and 6. writing. |
| **Outcomes** | Different statewide tests |
| **Notes** | Kemple et al. (2009) and Corrin et al. (2010) study the same interventions and we have used information from all three studies during coding. |

*Stevens (2003)*

| **Methods** | Quasi-experimental study. |
| --- | --- |
| **Participants** | Middle school students in grade 6 and 8 from low income families. Intervention group: 1798 students. Control group: 2188 students. |
| **Interventions** | Student Team Reading and Writing has 3 principals: literature-related activities, direct instruction in reading comprehension strategies, and selection-related writing. All activities followed a regular cycle that involved teacher presentation, cooperative learning with team practice, independent practice, peer pre-assessment and individual accountability. The intervention targets: vocabulary, fluency, comprehension, decoding, and writing. |
| **Outcomes** | California Achievement Test, subtests Vocabulary, Comprehension, Language mechanics, Language expression. |

*Swanson et al. (2015)*

| **Methods** | Randomised controlled trial. |
| --- | --- |
| **Participants** | Struggling readers in grade 8. Intervention group: 72 students. Control group: 58 students. |
| **Interventions** | The Promoting Acceleration of Comprehension and Content Through Text (PACT) intervention consisted of five components of instruction that focused on improving comprehension through text reading, connecting new text-based learning to prior learning, and applying new knowledge to unique problem-solving activities completed in cooperative groups of different size. Ongoing support of teachers was provided through in-person coaching. The intervention targets comprehension and vocabulary. |
| **Outcomes** | Gates-MacGinite, subtest Reading Comprehension. |

*Swanson et al. (2016)*

| **Methods** | Randomised controlled trial. |
| --- | --- |
| **Participants** | Students with reading difficulties in grade 8. Intervention group: 45 students. Control group: 33 students. |
| **Interventions** | The intervention, PACT, consisted of the following five components: 1. Comprehension canopy: designed to build background knowledge and motivation. 2. Essential words: a set of 4–5 high-utility, high-frequency concepts are taught and reviewed over the span of each 10-day unit, 3. Warm-up: lessons begin with a 5-min review of an essential word using an activity that requires students to apply the meaning of the word. 4. Critical reading: students read and discuss information from primary and secondary sources, 5. Team-based learning. Furthermore, a doctoral candidate–level research assistant with extensive teaching experience and experience implementing the PACT intervention provided in-person coaching once per week. |
| **Outcomes** | Gates-MacGinitie, subtest Reading Comprehension. |

*Tidd et al. (2018)*

| **Methods** | Quasi-experimental study. |
| --- | --- |
| **Participants** | Students in grade 9 with the lowest level of prior math performance. Intervention group: 277 students. Control group: 242 students. |
| **Interventions** | Students are given an intensified algebra course, which includes extra time, use of comprehensive, web-based tools and print material in the context of class-room instruction, laptops, desktops, and tablets and online assessments, which are used by both teachers and students. Teachers are coached. In addition to algebra, the course aims to improve meta-cognitive skills and reading comprehension. |
| **Outcomes** | State of Florida’s algebra 1 end-of-course assessment |

*Tijms et al. (2017)*

| **Methods** | Randomised controlled trial. |
| --- | --- |
| **Participants** | Young adolescents in first year of secondary education (grade 7) from urban, low SES communities. Intervention group: 40 students. Control group: 50 students. |
| **Interventions** | Instruction aimed to improve the young adolescents’ reading attitudes, reading comprehension and social–emotional competences, delivered in small groups (5-7 students). |
| **Outcomes** | Vlaamse Test Begrijpend Lezen, test of reading comprehension. |

*Travillian (2011)*

| **Methods** | Quasi-experimental study. |
| --- | --- |
| **Participants** | Students at risk of dropping out of school in grade 6 to 8. Intervention group: 46 students. Control group: 46 students. |
| **Interventions** | The intervention used a graduation coach to work with students at-risk of dropping out. The coach used a reward system for those students who came to school, behaved in class, and completed their homework. Other components included helping the students develop short and long-range goals, informal student progress monitoring, recognizing accomplishments, mentoring, tutoring, monitoring attendance, and monitoring behaviour. |
| **Outcomes** | Georgia Criterion-Referenced Competency Test |

*Vaughn et al. (2010)*

| **Methods** | Randomised controlled trial. |
| --- | --- |
| **Participants** | Struggling readers in grade 7 to 8. Small group intervention: 50 students. Large group intervention: 191 students. Control group: 207 students. |
| **Interventions** | The intervention included instruction in groups of 6-15 students and one with groups of 10-15 students. The intervention targeted multisyllable word study, academic vocabulary acquisition, development, reading fluency, and comprehension. |
| **Outcomes** | Texas Assessment of Knowledge and Skills; Group Reading Assessment and Diagnostic Evaluation; Woodcock-Johnson III; Test of Sentence Reading Efficiency, subtests Letter-Word Identification, Word Attack, Passage Comprehension, Spelling; Test of Word Reading Efficiency, subtest Reading Efficiency. |

*Vaughn et al. (2011)*

| **Methods** | Randomised controlled trial. |
| --- | --- |
| **Participants** | Low performing students in grade 7 to 8. Intervention group: 49 students. Control group: 46 students. |
| **Interventions** | The intervention, Collaborative Strategic Reading (CSR), uses collaborative learning among students (i.e., peer-assisted learning) and targets reading comprehension strategies and meta-cognitive strategies. The research team provided in-class support and coaching for each participating teacher. |
| **Outcomes** | Gates–MacGinitie, subtest Comprehension; Test of Silent Reading Efficiency and Comprehension. |

*Vaughn et al. (2013)*

| **Methods** | Randomised controlled trial. |
| --- | --- |
| **Participants** | Students with reading difficulties in grade 7 to 8. Intervention group: 17 students. Control group: 18 students. |
| **Interventions** | Guided by their teacher, students work collaboratively with their peers to read a text and use comprehension strategies. CSR systematically builds students’ background knowledge, motivation, and engagement, as well as assists teachers in identifying appropriate levels of text difficulty for their students. The research team provided in-class support and coaching for each participating teacher. |
| **Outcomes** | Gates–MacGinitie, subtest Comprehension; Test of Silent Reading Efficiency and Comprehension. |

*Vaughn et al. (2015)*

| **Methods** | Randomised controlled trial. |
| --- | --- |
| **Participants** | Struggling readers in grade 10. Dropout intervention: 87 students. Reading intervention: 69 students. Dropout + reading intervention: 70 students. Control group: 79 students. |
| **Interventions** | One intervention group get a reading programme plus the dropout prevention programme (Check & Connect), and one intervention group gets only the reading programme. The reading programme included instruction in groups not larger than 10 students, students’ progress was monitored, and a research staff member provided regular coaching. The domains covered were word study, vocabulary in content text, comprehension in content texts, and engagement. The staff responsible for implementing the Check & Connect model with individual students and families functioned similar to a mentor. The Check component is based on behavioural and academic indicators of engagement (e.g., attendance, suspensions, course failures). The Connect component refers to the personal connections that mentors make with students, families, and school staff in the implementation of this intervention. |
| **Outcomes** | Gates-MacGinitie Reading Tests, subtest Comprehension |
| **Notes** | We used information from Anderson, Christenson, Sinclair, & Lehr (2004) to code the Check & Connect component. |

*Vaughn et al. (2017)*

| **Methods** | Randomised controlled trial. |
| --- | --- |
| **Participants** | English language learners in grade 8. Intervention group: 236 students. Control group: 173 students. |
| **Interventions** | Students work in pairs or small groups during most parts of the intervention. Videos, visuals and graphic organizers were used. There are comprehension checks through continuous targeted feedback in which teachers affirmed or corrected students' understanding of the content. Teachers are coached. Intervention aim to improve reading comprehension and vocabulary. |
| **Outcomes** | Gates-MacGinite, reading comprehension subtest. |

*Wanzek et al. (2011)*

| **Methods** | Randomised controlled trial. |
| --- | --- |
| **Participants** | Students in grade 6 to 8 with learning disabilities. Intervention group: 65 students. Control group: 55 students. |
| **Interventions** | The students assigned to the intervention were provided a supplemental, remedial reading intervention class for one period a day, 5 days a week for a school year. The students were placed in small class sizes of 10 to 15 students for the supplemental intervention with other students with reading difficulties that were participating in the larger study. Fluency was promoted by using oral reading fluency data and pairing higher and lower readers for partner reading. The intervention combined vocabulary and comprehension techniques with opportunities for guided discussion to address student needs in understanding the words and text. It also included explicit instruction in English phonology, recognizing high frequency words accurately and quickly, and a strategy for applying phonics elements to reading multisyllable words. |
| **Outcomes** | Woodcock-Johnson III Tests of Achievement, subtests Letter Word Identification, Word Attack, and Passage Comprehension; Test of Word Reading Efficiency, subtests Sight Word Efficiency and Phonemic Decoding Efficiency. |

*Weichenthal (1985)*

| **Methods** | Randomised controlled trial. |
| --- | --- |
| **Participants** | Learning-disabled students in grade 4 to 8 enrolled in a special education resource-specialist programme. All subject have a large discrepancy between achievement on a standardised test and their IQ. Intervention group: 30 students. Control group: 30 students. |
| **Interventions** | The intervention provided metacognitive training of broad, general, metacognitive, self-regulatory techniques that was based on transfer principles with the aim to improve reading. These techniques included active involvement, self-talk, self-monitoring, self-questioning, and self-correction. The intervention also contained a motivational component, where students got points for doing tasks which can be exchanged for either stickers or posters. |
| **Outcomes** | Gates-McGinite Reading Tests, subtest Comprehension |
| **Notes** | Another intervention in this study (Directions Training) had a majority of students in grades below 7, and was therefore not included in this review. |

*Wexler et al. 2010*

| **Methods** | Randomised controlled trial. |
| --- | --- |
| **Participants** | Students in grade 9 to 12 with significant reading disabilities. Repeated Reading: 33 students in intervention. Wide Reading: 34 students in intervention. Control group: 29 students. |
| **Interventions** | Studied two interventions, Repeated Reading and Wide Reading. Repeated Reading involved students working in pairs, one higher and one lower level reader, so that each partner read the same text three times, exposing the pair to one text six times. Modeling for the lower level reader was incorporated by having the higher-level reader read the passage first. Students were explicitly taught how to provide immediate and corrective feedback during the week prior to implementing the intervention. Error correction consisted of each student reviewing missed words with their partner after their second read. Wide reading had a similar set up in terms of peer-assisted learning but students in the wide reading group did not read the same text more than one time. Each partner read three different texts one time each while his/her partner followed along, exposing the pair to six different texts. In this condition, error correction from partners and a summarization component was also included. Both interventions targeted fluency, comprehension, and word reading. |
| **Outcomes** | Test of Silent Contextual Reading Fluency; Test of Silent Reading Efficiency; Woodcock-Johnson Tests of Achievement III, subtests Passage Comprehension, Letter-Word Identification |

*White (2000)*

| **Methods** | Randomised controlled trial. |
| --- | --- |
| **Participants** | At-risk students in grade 8 defined as any student whose score in mathematics on the nationally norm-referenced Stanford 9 Achievement Test was at least two years below grade level. Intervention group: 13 students. Control group: 15 students. |
| **Interventions** | For the 8^th^ graders the intervention consisted of tutoring 6^th^ grade students. The tutors also received small group training in several skills. In addition, the participants (tutors) received support from the researchers (teachers) to assist them in presenting the curriculum and reflecting on their participation. Participant reflection occurred through the use of journals. The overall aim was to increase mathematics achievement, academic efficacy, and cognitive development. The intervention also targeted principles of tutoring, communication skills, helping behaviours, record keeping procedures, behaviour management and journal keeping skills. |
| **Outcomes** | Texas Assessment of Academic Skills, subtest Problem-solving; Arlin Test of Formal Reasoning. |
| **Notes** | The intervention for 6^th^ graders was not included in this review. |

*Wyllie (2008)*

| **Methods** | Quasi-experimental study. |
| --- | --- |
| **Participants** | Low performing students in grade 11. Intervention group: 96 students. Control group: 97 students. |
| **Interventions** | Students participated in a variety of instructional activities ranging from individual work to small group and whole-class instruction. Activities included several forms of skill practice following direct instruction, group discussion, one-on-one instruction, and interaction with a computer software programme. Students received additional instruction in reading and math in classes with a size limit of twelve students. The intervention targeted general domains of both reading and mathematics. |
| **Outcomes** | PSSA reading and mathematics scores |

**Comparison Designs**

Table A2 describes the 33 comparison designs in terms of publication year, country, subject, and the contrast between the two or more alternative interventions studied.

*Table A2. Comparison designs*

| **Authors** | **Country** | **Test subject** | **Contrast** |
| --- | --- | --- | --- |
| Alloway (2012) | US | Math, reading | Interactive working memory training vs targeted educational support. |
| Bemboom & McMaster (2013) | US | Reading | Compares a peer-mediated versus teacher-directed reading intervention. |
| Bowman (1999) | US | Reading | CAI vs no CAI in a phonics intervention. |
| Butler et al. (2003) | US | Math | Compares two sequences of instruction in a fraction intervention. |
| Calhoun (2011) | US | Math | Compares CAI with extra instructional time. |
| Cook et al. (2015) | US | Math | Compares a group that received small group tutoring or small group tutoring plus a social-cognitive skills intervention with the comparison group that either received treatment as usual or the social-cognitive skills intervention. |
| Crowe (2007) | US | Reading | Explicit rule-based reading instruction was compared to a more traditional basal instruction intervention. |
| Dee (2015) | US | Math, reading | Stereotype threat intervention, however all students received something extra from the intervention. |
| Denton et al. (2017) | US | Reading | One group receives training in reading comprehension by experienced teachers and one group receives time-on-task control conditions using a software application. |
| Doss (2015) | US | Reading | A comparison group got a smaller dosage of a CAI intervention, than two intervention groups that received a higher dosage. |
| Freeman (2010) | US | Math | Compare groups in different years who receive more or less of a CAI intervention with embedded instructional support. |
| Fryer et al. (2016) | US | Math, reading | Compare interventions with payment given either to single students to improve attendance, behaviour, and interim assessments or to multiple agents for the improvement of math plus the Accelerated Math program to an intervention where students get only the program. |
| Fuchs (1988) | US | Reading | Teachers of both the intervention and comparison group received (different forms of) feedback from a CBM tool. |
| Gourgey (1987) | US | Math, reading | All students received different forms of CAI. |
| Hanselman et al. (2014) | US | Math, reading | Stereotype threat intervention, however all students received something extra from the intervention. |
| Harding et al. (2012) | US | Math, reading | Different forms of supplemental educational services, e.g., small group or one-to-one tutoring. |
| Jacobs (2012) | US | Reading | The effect sizes that were included compared two interventions, Read 180 and Thinking Reader. |
| Klingner & Vaughn (1996) | US | Reading | Compare reciprocal teaching with cross-age tutoring to reciprocal teaching with cooperative grouping. |
| Lang et al. (2009) | US | Reading | Four different interventions to which students of two broad reading levels were randomly assigned. Compares READ 180, REACH, and RISE to a fourth condition, of which it is stated that “the control condition was considered a fourth approach to reading intervention rather than the absence of treatment.” (p. 152). |
| Lysynchuk et al. (1990) | Canada | Reading | Small group instruction with strategy training with feedback from assessments vs fewer session of small group instruction and without strategy training, but with assessment feedback. |
| Marttila (2017) | US | Math | Compare two separate mathematics programs, Saxon Math and Ready Common Core. |
| Montague et al. (2014) | US | Math | The intervention consisted of CBM-based measurement feedback and cognitive strategy instruction. The comparison group also get extra CBM-based feedback although fewer measurement points than the intervention group. |
| Muoneke (2001) | US | Math | Both the intervention group and the comparison group received training from a teacher that had received professional development due to the intervention. |
| Parker (2014) | US | Reading | Examined the effectiveness of two direct instruction programmes, SRA Reading Mastery Signature and SRA Reading Success. |
| Potocki (2015) | France | Reading | Students in the intervention groups worked with a software programme designed to encourage either their written word decoding skills (grapho-syllabic software training) or their text comprehension skills (comprehension software training). |
| Powell (2007) | US | Math, reading | Compare 3 interventions: Support services delivery model (SS): A teaching model distinguished by the use of a paraprofessional assisting the student with disabilities in the general education classroom with the general education teacher as the teacher of record in teaching the content taught. Team or collaborative service delivery model (TC): A partnership where two or more individuals or organizations actively work together on a project or problem. Departmental or pull-out delivery model (DP): A special education classroom environment where students receive instruction from a special education teacher for the content taught. |
| Robby (2008) | US | Reading | A READ 180 course was compared to comparison students receiving a Standard ELA course plus an Intensive English 1 course delivered by a teacher. |
| Sasser (2016) | US | Reading | Compare full and partial inclusion of special needs students. |
| Shimizu (1995) | US | Reading | Study of two research based reading comprehension models. |
| Shippen et al. (2005) | US | Reading | Investigated the differential effects of two direct instruction (DI) reading programmes, one with overt decoding strategies and one with more covert decoding strategies. |
| Spencer (2010) | US | Reading | The experimental group participated in the Great Leaps Reading programme for 10 minutes a day, working one-on-one with trained paraprofessionals. The comparison group participated in a similar one-on-one intervention with different paraprofessionals, trained to utilize the Skills for School Success study skills programme. |
| Stultz (2008) | US | Math | Both intervention and comparison group got instruction from a researcher. |
| Thomas (2005) | US | Reading | The intervention group received the READ 180 programme and the comparison group an alternative reading initiative. |
| Tillman-Walker (2017) | US | Math | Compared differences in student achievement in math in three classroom conditions: 1) Students with specific learning disabilities (SLD) in co-taught classrooms, 2) non-SLD in co-taught, 3), SLDs in resource classes with no co-taught, and 4) non-SLD in general education and non-co-taught classes. |
| Wang et al. (2016) | US | Reading | Compared four reading intervention programmes: Language! Live, Corrective Reading, Soar to Success, and Wilson reading intervention. |
| Warner & Alley (1981) | US | Reading | Compared two strategies for recalling prose passages: using visual imagery as an aid for memory and paraphrase recall practice. |
| Woods (2007) | US | Reading | The intervention group received READ 180 and comparison group received small-group tutoring. |
| Wynne (2012) | US | Math, reading | Stereotype threat intervention, however all students received something extra from the intervention. |

**Studies with Overlapping Samples or Lacking Information**

Table A3 displays the studies that we did not include in the meta-analysis because we could not retrieve enough information to calculate an effect size, or because they used samples that overlapped with other included studies. To save space, the last group of included studies that we did not use in the meta-analysis, those with too high risk of bias, are displayed in Table A5 further below along with information about the risk of bias rating.

*Table A3. Studies with overlapping samples or lacking information*

| **Authors** | **Country** | **Test subject** | **Reason for not including the study in the meta-analysis** |
| --- | --- | --- | --- |
| Barnard (2004) | US | Math, reading | Lack information to calculate an effect size. |
| Dunleavy & Heinecke (2007) | US | Math, science | Lack information to calculate an effect size. |
| Dynarski et al. (1998) | US | Math, reading | Lack information to calculate an effect size. |
| Hoek et al. (1997) | Netherlands | Math | Lack information to calculate an effect size. |
| Hummel & Hahn (1982) | US | Math | Lack information to calculate an effect size. |
| Hunter (1994) | US | Math, reading | Lack information to calculate an effect size. |
| Lange et al. (2009) | UK | Reading | Lack information to calculate an effect size. |
| Monye (2017) | US | Math | Lack information to calculate an effect size. |
| Opuni et al. (1991) | US | Math | Lack information to calculate an effect size. |
| Rodick & Henggeler (1980) | US | Reading | Lack information to calculate an effect size. |
| Silva (2009) | US | Reading | Lack information to calculate an effect size. |
| Stavros (1989) | US | Math | Lack information to calculate an effect size. |
| Stevens et al. (1989) | US | Math | Lack information to calculate an effect size. |
| Dynarski et al. (2007) | US | Math | Overlapping samples with Campuzano et al. (2009). |
| Klingner (1994) | US | Reading | Overlapping samples with Klingner & Vaughn (1996). |
| Solis et al. (2014) | US | Reading | Overlapping samples with Vaughn et al. (2010). |
| Vaughn et al. (2011) | US | Reading | Overlapping samples with Vaughn et al. (2010). |
| Vaughn et al. (2012) | US | Reading | Overlapping samples with Roberts et al. (2013). |
| Wanzek et al. (2016) | US | Reading | Overlapping samples with Vaughn et al. (2017). |
| Wexler (2007) | US | Reading | Overlapping samples with Wexler et al. (2010). |

## Search Strategy by Database

Following figure shows the search results for each bibliographic database searched in the original 2016 search and the 2018 update.

| **Database** | **Results in March 2016** | **Results in July 2018** |
| --- | --- | --- |
| Academic Search Premier (EBSCO-host) | 1,548 | 759 |
| ERIC (EBSCO-host) | 5,862 | 422 |
| PsycINFO (EBSCO-host) | 5,505 | 1,065 |
| SocIndex (EBSCO-host) | 328 | 91 |
| British Education Index (EBSCO-host) | 96 | No access in 2018 |
| Teacher Reference Center (EBSCO-host) | 643 | 188 |
| ECONLIT (EBSCO-host) | 58 | 16 |
| FRANCIS (EBSCO-host) | 165 | No access in 2018 |
| Dissertation & theses A&I (ProQuest-host) | 786 | No access in 2018 |
| CBCA Education (ProQuest-host) | 97 | No access in 2018 |
| Australian Education Index (ProQuest-host) | 85 | No access in 2018 |
| Social Science Citation Index (SSCI)(WOS) | 2,376 | 907 |
| Science Citation Index (SCI) (WOS) | Searched with SSCI | Searched with SSCI |
| PubMed (OVID-host) | 323 | 0 |
| Embase (OVID-host) | Searched with PubMed | Searched with PubMed |

**ERIC**

Searched March 2016. Limiters - Published Date: 19800101-20161231. Search modes - Boolean/Phrase. Interface - EBSCOhost Research Databases. The same search string with minor modifications was used to search the other databases from EBSCO-host and in 2018.

| **Search** | **Query** | **Results** |
| --- | --- | --- |
| S63 | S41 AND S62 - Limiters - Published Date: 19800101-20161231 | 5,862 |
| S62 | S42 OR S43 OR S44 OR S45 OR S46 OR S47 OR S48 OR S49 OR S50 OR S51 OR S52 OR S53 OR S54 OR S55 OR S56 OR S57 OR S58 OR S59 OR S60 OR S61 | 351,270 |
| S61 | TI (regression N1 discontinuity OR difference-in-difference* OR event N1 stud* OR interrupted time serie* OR instrumental variable* OR waitlist control*) OR AB (regression N1 discontinuity OR difference-in-difference* OR event N1 stud* OR interrupted time serie* OR instrumental variable* OR waitlist control*) | 1,696 |
| S60 | TI ((control N5 case) or (control N5 subject*) or (control N5 group*) or (control N5 patient*) or (control N5 intervention) ) or AB ( (control N5 case) or (control N5 subject*) or (control N5 group*) or (control N5 patient*) or (control N5 intervention)) | 19,818 |
| S59 | TI ((treatment N5 case) or (treatment N5 subject*) or (treatment N5 group*) or (treatment N5 patient*) or (treatment N5 intervention) ) or AB ( (treatment N5 case) or (treatment N5 subject*) or (treatment N5 group*) or (treatment N5 patient*) or (treatment N5 intervention)) | 9,401 |
| S58 | TI ((experiment* N5 case) or (experiment* N5 subject*) or (experiment* N5 group*) or (experiment* N5 patient*) or (experiment* N5 intervention)) or AB ( (experiment* N5 case) or (experiment* N5 subject*) or (experiment* N5 group*) or (experiment* N5 patient*) or (experiment* N5 intervention)) | 12,735 |
| S57 | TI ((intervention N5 case) or (intervention N5 subject*) or (intervention N5 group*) or (intervention N5 patient*) ) or AB ( (intervention N5 case) or (intervention N5 subject*) or (intervention N5 group*) or (intervention N5 patient*) ) | 5,107 |
| S56 | ((assign* N5 case) or (assign* N5 subject*) or (assign* N5 group*) or (assign* N5 patient*) or (assign* N5 intervention) ) or AB ( (assign* N5 case) or (assign* N5 subject*) or (assign* N5 group*) or (assign* N5 patient*) or (assign* N5 intervention) ) | 6,551 |
| S55 | TI ( quasi-experiment* or quasiexperiment* or Propensity score* or (compar* N1 group*) or (match* N1 control*) or (match* N1 group*) or (match* N1 compar*) or experiment* trial* or experiment* design* or experiment* method* or experiment* stud* or experiment* evaluation* or experiment* test* or experiment* assessment* or assessment only or (comparison n1 samp*) or propensity match* or (Between N1 group*)) or AB ( quasi-experiment* or quasiexperiment* or Propensity score* or (compar* N1 group*) or (match* N1 control*) or (match* N1 group*) or (match* N1compar*) or experiment* trial* or experiment* design* or experiment* method* or experiment* stud* or experiment* evaluation* or experiment* test* or experiment*assessment* or assessment only or (comparison n1samp*) or propensity match* or (Between N1 group*)) | 41,459 |
| S54 | TI ((random* N2 trial*) or RCT) OR AB ((random* N2 trial*) or RCT) | 2,573 |
| S53 | TI Non-random* or nonradom* or (non N1 random*) or AB Non-random* or Nonrandom* or (non N1 random*) | 595 |
| S52 | TI ((Propensity score* or (match* N1 control*) or (match* N1 compar* ) or assessment only or comparison samp* or propensity match*)) or AB ((Propensity score* or (match* N1 control*) or (match* N1 compar* ) or assessment only or comparison samp* or propensity match*)) | 4,215 |
| S51 | TI assign* N3 (subject* or patient* ) or AB assign* N3 (subject* or patient* ) | 1,017 |
| S50 | TI (quasi-experiment* or quasiexperiment* or experiment*) or AB (quasi-experiment* or quasiexperiment* or experiment*) | 70,651 |
| S49 | TI Intervention* N1 Stud* or AB Intervention* N1 Stud* | 3,147 |
| S48 | TI ((prospective n2 study) or AB (prospective n2 study)) or (TI retrospective or AB retrospective) | 4,258 |
| S47 | ( TI longitudinal or AB longitudinal) or ( TI observational or AB observational) | 26,677 |
| S46 | (TI (epidemiologic N2 study) or AB (epidemiologic N2 study)) or (Ti (follow up or followup) N2 study ) or AB ((follow up or followup) N2 study)) | 4,352 |
| S45 | TI cross sectional or AB cross sectional | 4,158 |
| S44 | TI ((case control) or AB (case control)) or TI cohort or AB cohort | 10,073 |
| S43 | DE "Cohort analysis" or DE "Case Studies" | 49,068 |
| S42 | AB randomized or AB placebo or AB randomly or trial or AB groups | 221,926 |
| S41 | S26 AND S33 AND S40 | 16,059 |
| S40 | S34 OR S35 OR S36 OR S37 OR S38 OR S39 | 349,663 |
| S39 | transfer* N2 effect | 487 |
| S38 | Writ* or DE "Writing Ability" or DE "Writing Achievement" | 131,831 |
| S37 | Numeracy or Mathematic* or Math | 94,817 |
| S36 | DE "Mathematics" or DE "Numeracy" | 9,141 |
| S35 | Reading or Literacy | 147,390 |
| S34 | DE "Reading" or DE "Literacy" | 15,509 |
| S33 | S27 OR S28 OR S29 OR S30 OR S31 OR S32 | 108,201 |
| S32 | Intellect* N2 develop* | 4,333 |
| S31 | DE "Intellectual Development" | 1,720 |
| S30 | School N1 (performan* or achiev*) | 8,802 |
| S29 | Academic* N2 (performance* or achiev* or abilit* or outcome*) | 75,551 |
| S28 | Learn* N2 ( disab* or Problem*) | 27,516 |
| S27 | DE "Academic Achievement" or DE "Academic Ability" or DE "Learning Problems" or (DE "Learning Disabilities") | 82,071 |
| S26 | S22 OR S23 OR S24 OR S25 | 214,904 |
| S25 | (Student* or pupil*) N3 (Learn* N2 ( disab* or Problem*)) | 7,218 |
| S24 | (Child* N2 (placed n1 care)) or ((DE "Foster Care") AND child*) | 1,833 |
| S23 | ((Primary N1 School ) N3 (Student* or pupil*)) or ((Elementary N1 School) N3 (Student* or pupil*)) or (DE "Elementary School Students") or ((Secondary N1 school) or ( high N2 school) or (middle N1 School) N3 (student* or pupil*)) | 188,810 |
| S22 | (Underachiev* or Under n1 achiev* or lowachiev* or low n1 achiev* or Low N1 perform* or lowperform* or (at-risk or at N1 risk)) N1 (student* or pupil*) or ((high-risk or high N1 risk) N1 (student* or pupil*)) or ((Special N1 Need*) N1 (Student* or pupil*)) or ((Low N1 income) N1 (student* or pupil*)) | 29,906 |
| S21 | S1 OR S2 OR S3 OR S4 OR S5 OR S6 OR S7 OR S8 OR S9 OR S10 OR S11 OR S12 OR S13 OR S14 OR S15 OR S16 OR S17 OR S18 OR S19 OR S20 | 1,362,816 |
| S20 | TI (regression N1 discontinuity OR difference-in-difference* OR event N1 stud* OR interrupted time serie* OR instrumental variable* OR waitlist control*) OR AB (regression N1 discontinuity OR difference-in-difference* OR event N1 stud* OR interrupted time serie* OR instrumental variable* OR waitlist control*) | 6,167 |
| S19 | TI ((control N5 case) or (control N5 subject*) or (control N5 group*) or (control N5 patient*) or (control N5 intervention) ) or AB ( (control N5 case) or (control N5 subject*) or (control N5 group*) or (control N5 patient*) or (control N5 intervention)) | 141,211 |
| S18 | TI ((treatment N5 case) or (treatment N5 subject*) or (treatment N5 group*) or (treatment N5 patient*) or (treatment N5 intervention) ) or AB ( (treatment N5 case) or (treatment N5 subject*) or (treatment N5 group*) or (treatment N5 patient*) or (treatment N5 intervention)) | 124,784 |
| S17 | TI ((experiment* N5 case) or (experiment* N5 subject*) or (experiment* N5 group*) or (experiment* N5 patient*) or (experiment* N5 intervention)) or AB ( (experiment* N5 case) or (experiment* N5 subject*) or (experiment* N5 group*) or (experiment* N5 patient*) or (experiment* N5 intervention)) | 44,633 |
| S16 | TI ((intervention N5 case) or (intervention N5 subject*) or (intervention N5 group*) or (intervention N5 patient*) ) or AB ( (intervention N5 case) or (intervention N5 subject*) or (intervention N5 group*) or (intervention N5 patient*) ) | 39,367 |
| S15 | ((assign* N5 case) or (assign* N5 subject*) or (assign* N5 group*) or (assign* N5 patient*) or (assign* N5 intervention) ) or AB ( (assign* N5 case) or (assign* N5 subject*) or (assign* N5 group*) or (assign* N5 patient*) or (assign* N5 intervention) ) | 22,883 |
| S14 | TI ( quasi-experiment* or quasiexperiment* or Propensity score* or (compar* N1 group*) or (match* N1 control*) or (match* N1 group*) or (match* N1 compar*) or experiment* trial* or experiment* design* or experiment* method* or experiment* stud* or experiment* evaluation* or experiment* test* or experiment* assessment* or assessment only or (comparison n1 samp*) or propensity match* or (Between N1 group*)) or AB ( quasi-experiment* or quasiexperiment* or Propensity score* or (compar* N1 group*) or (match* N1 control*) or (match* N1 group*) or (match* N1compar*) or experiment* trial* or experiment* design* or experiment* method* or experiment* stud* or experiment* evaluation* or experiment* test* or experiment*assessment* or assessment only or (comparison n1samp*) or propensity match* or (Between N1 group*)) | 204,454 |
| S13 | TI ((random* N2 trial*) or RCT) OR AB ((random* N2 trial*) or RCT) | 35,566 |
| S12 | TI Non-random* or nonradom* or (non N1 random*) or AB Non-random* or Nonrandom* or (non N1 random*) | 3,729 |
| S11 | TI ((Propensity score* or (match* N1 control*) or (match* N1 compar* ) or assessment only or comparison samp* or propensity match*)) or AB ((Propensity score* or (match* N1 control*) or (match* N1 compar* ) or assessment only or comparison samp* or propensity match*)) | 35,355 |
| S10 | TI assign* N3 (subject* or patient* ) or AB assign* N3 (subject* or patient* ) | 4,853 |
| S9 | TI (quasi-experiment* or quasiexperiment* or experiment*) or AB (quasi-experiment* or quasiexperiment* or experiment*) | 350,982 |
| S8 | TI Intervention* N1 Stud* or AB Intervention* N1 Stud* | 10,762 |
| S7 | TI ((prospective n2 study) or AB (prospective n2 study)) or (TI retrospective or AB retrospective) | 48,356 |
| S6 | ( TI longitudinal or AB longitudinal) or ( TI observational or AB observational) | 99,218 |
| S5 | (TI (epidemiologic N2 study) or AB (epidemiologic N2 study)) or (Ti (follow up or followup) N2 study ) or AB ((follow up or followup) N2 study)) | 17,617 |
| S4 | TI cross sectional or AB cross sectional | 50,361 |
| S3 | TI ((case control) or AB (case control)) or TI cohort or AB cohort | 63,802 |
| S2 | DE "Cohort analysis" | 1,178 |
| S1 | AB randomized or AB placebo or AB randomly or trial or AB groups | 843,084 |

**Updated search in 2018**

**Social Science Citation Index & Science Citation Index**

Searched March 2016. The search was updated 25/06-2018. The updated search was limited from 01/03/2016-25/06-2018. The updated search resulted in 907 new references.

| # 55 | 2,376 | #54 AND #25  *Indexes=SCI-EXPANDED, SSCI Timespan=1980-2016* |
| --- | --- | --- |
| # 54 | [8,643,433](http://apps.webofknowledge.com/summary.do?product=WOS&doc=1&qid=103&SID=R2hHC4XffySe81g3tkG&search_mode=CombineSearches&update_back2search_link_param=yes) | #53 OR #52 OR #51 OR #50 OR #49 OR #48 OR #47 OR #46 OR #45 OR #44 OR #43 OR #42 OR #41 OR #40 OR #39 OR #38 OR #37 OR #36 OR #35 OR #34 OR #33 OR #32 OR #31 OR #30 OR #29 OR #28 OR #27 OR #26  *Indexes=SCI-EXPANDED, SSCI Timespan=All years* |
| # 53 | [21,024](http://apps.webofknowledge.com/summary.do?product=WOS&doc=1&qid=102&SID=R2hHC4XffySe81g3tkG&search_mode=AdvancedSearch&update_back2search_link_param=yes) | (TS=(regression NEAR/1 (discontinuity)) OR TS=(difference-in-difference*) OR TS=(event NEAR/1 stud*) OR TS=(interrupted time serie*) OR TS=(instrumental variable*) OR TS=(waitlist control*)) *AND* **DOCUMENT TYPES:** (Article)  *Indexes=SCI-EXPANDED, SSCI Timespan=All years* |
| # 52 | [21,024](http://apps.webofknowledge.com/summary.do?product=WOS&doc=1&qid=101&SID=R2hHC4XffySe81g3tkG&search_mode=AdvancedSearch&update_back2search_link_param=yes) | ((TI=(regression NEAR/1 (discontinuity)) OR TI=(difference-in-difference*) OR TI=(event NEAR/1 stud*) OR TI=(interrupted time serie*) OR TI=(instrumental variable*) OR TI=(waitlist control*)) OR (TS=(regression NEAR/1 (discontinuity)) OR TS=(difference-in-difference*) OR TS=(event NEAR/1 stud*) OR TS=(interrupted time serie*) OR TS=(instrumental variable*) OR TS=(waitlist control*))) *AND* **DOCUMENT TYPES:** (Article)  *Indexes=SCI-EXPANDED, SSCI Timespan=All years* |
| # 51 | [687,264](http://apps.webofknowledge.com/summary.do?product=WOS&doc=1&qid=94&SID=R2hHC4XffySe81g3tkG&search_mode=AdvancedSearch&update_back2search_link_param=yes) | (TI=(control NEAR/5 (case)) OR TI=(control NEAR/5 (subject*)) OR TI=(control NEAR/5 (group*)) OR TI=(control NEAR/5 (patient*)) OR TI=(control NEAR/5 (intervention)) OR TS=(control NEAR/5 (case)) OR TS=(control NEAR/5 (subject*)) OR TS=(control NEAR/5 (group*)) OR TS=(control NEAR/5 (patient*)) OR TS=(control NEAR/5 (intervention))) *AND* **DOCUMENT TYPES:** (Article)  *Indexes=SCI-EXPANDED, SSCI Timespan=All years* |
| # 50 | [498,652](http://apps.webofknowledge.com/summary.do?product=WOS&doc=1&qid=93&SID=R2hHC4XffySe81g3tkG&search_mode=AdvancedSearch&update_back2search_link_param=yes) | (TI=(treatment NEAR/5 (case)) OR TI=(treatment NEAR/5 (subject*)) OR TI=(treatment NEAR/5 (group*)) OR TI=(treatment NEAR/5 (patient*)) OR TI=(treatment NEAR/5 (intervention*)) OR TS=(treatment NEAR/5 (case)) OR TS=(treatment NEAR/5 (subject*)) OR TS=(treatment NEAR/5 (group*)) OR TS=(treatment NEAR/5 (patient*)) OR TS=(treatment NEAR/5 (intervention*))) *AND* **DOCUMENT TYPES:** (Article)  *Indexes=SCI-EXPANDED, SSCI Timespan=All years* |
| # 49 | [116,438](http://apps.webofknowledge.com/summary.do?product=WOS&doc=1&qid=92&SID=R2hHC4XffySe81g3tkG&search_mode=AdvancedSearch&update_back2search_link_param=yes) | (TI=(experiment* NEAR/5 (case*)) OR TI=(experiment* NEAR/5 (subject*)) OR TI=(experiment* NEAR/5 (group*)) OR TI=(experiment* NEAR/5 (patient*)) OR TI=(experiment* NEAR/5 (intervention*)) OR TS=(experiment* NEAR/5 (case*)) OR TS=(experiment* NEAR/5 (subject*)) OR TS=(experiment* NEAR/5 (group*)) OR TS=(experiment* NEAR/5 (patient*)) OR TS=(experiment* NEAR/5 (intervention*))) *AND* **DOCUMENT TYPES:** (Article)  *Indexes=SCI-EXPANDED, SSCI Timespan=All years* |
| # 48 | [101,864](http://apps.webofknowledge.com/summary.do?product=WOS&doc=1&qid=90&SID=R2hHC4XffySe81g3tkG&search_mode=AdvancedSearch&update_back2search_link_param=yes) | (TI=(intervention NEAR/5 (case*)) OR TI=(intervention NEAR/5 (subject*)) OR TI=(intervention NEAR/5 (group*)) OR TI=(intervention NEAR/5 (patient*)) OR TS=(intervention NEAR/5 (case*)) OR TS=(intervention NEAR/5 (subject*)) OR TS=(intervention NEAR/5 (group*)) OR TS=(intervention NEAR/5 (patient*))) *AND* **DOCUMENT TYPES:** (Article)  *Indexes=SCI-EXPANDED, SSCI Timespan=All years* |
| # 47 | [72,600](http://apps.webofknowledge.com/summary.do?product=WOS&doc=1&qid=91&SID=R2hHC4XffySe81g3tkG&search_mode=AdvancedSearch&update_back2search_link_param=yes) | ((TS=(assign* NEAR/5 (case*)) OR TS=(assign* NEAR/5 (subject*)) OR TS=(assign* NEAR/5 (group*)) OR TS=(assign* NEAR/5 (patient*)) OR TS=(assign* NEAR/5 (intervention*)) OR TS=(assign* NEAR/5 (case*)) OR TS=(assign* NEAR/5 (subject*)) OR TS=(assign* NEAR/5 (group*)) OR TS=(assign* NEAR/5 (patient*)) OR TS=(assign* NEAR/5 (intervention*))) OR (TI=(assign* NEAR/5 (case*)) OR TI=(assign* NEAR/5 (subject*)) OR TI=(assign* NEAR/5 (group*)) OR TI=(assign* NEAR/5 (patient*)) OR TI=(assign* NEAR/5 (intervention*)) OR TI=(assign* NEAR/5 (case*)) OR TI=(assign* NEAR/5 (subject*)) OR TI=(assign* NEAR/5 (group*)) OR TI=(assign* NEAR/5 (patient*)) OR TI=(assign* NEAR/5 (intervention*)))) *AND* **DOCUMENT TYPES:** (Article)  *Indexes=SCI-EXPANDED, SSCI Timespan=All years* |
| # 46 | [2,577,788](http://apps.webofknowledge.com/summary.do?product=WOS&doc=1&qid=85&SID=R2hHC4XffySe81g3tkG&search_mode=AdvancedSearch&update_back2search_link_param=yes) | (TI=((quasi-experiment* OR quasiexperiment* OR Propensity score* OR (compar* NEAR/1 (group*))) OR (match* NEAR/1 (control*)) OR (match* NEAR/1 (group*)) OR (match* NEAR/1 (compar*)) OR (experiment* trial* OR experiment* design* OR experiment* method* OR experiment* stud* OR experiment* evaluation* OR experiment* test* OR experiment* assessment* OR assessment only OR (comparison NEAR/1 (samp*))) OR propensity match* OR (Between NEAR/1 (group*))) OR TS=(((quasi-experiment* OR quasiexperiment* OR Propensity score* OR (compar* NEAR/1 (group*))) OR (match* NEAR/1 (control*)) OR (match* NEAR/1 (group*)) OR (match* NEAR/1 (compar*)) OR (experiment* trial* OR experiment* design* OR experiment* method* OR experiment* stud* OR experiment* evaluation* OR experiment* test* OR experiment* assessment* OR assessment only OR (comparison NEAR/1 (samp*))) OR propensity match* OR (Between NEAR/1 (group*))))) *AND***DOCUMENT TYPES:** (Article)  *Indexes=SCI-EXPANDED, SSCI Timespan=All years* |
| # 45 | [274,613](http://apps.webofknowledge.com/summary.do?product=WOS&doc=1&qid=84&SID=R2hHC4XffySe81g3tkG&search_mode=AdvancedSearch&update_back2search_link_param=yes) | (TI=((random* NEAR/2 (trial* OR RCT))) OR TS=((random* NEAR/2 (trial* OR RCT)))) *AND* **DOCUMENT TYPES:** (Article)  *Indexes=SCI-EXPANDED, SSCI Timespan=All years* |
| # 44 | [15,043](http://apps.webofknowledge.com/summary.do?product=WOS&doc=1&qid=82&SID=R2hHC4XffySe81g3tkG&search_mode=AdvancedSearch&update_back2search_link_param=yes) | (TI=((Non-random* OR nonradom* OR (non NEAR/1 random*))) OR TS=((Non-random* OR nonradom* OR (non NEAR/1 random*)))) *AND***DOCUMENT TYPES:** (Article)  *Indexes=SCI-EXPANDED, SSCI Timespan=All years* |
| # 43 | [9,462](http://apps.webofknowledge.com/summary.do?product=WOS&doc=1&qid=80&SID=R2hHC4XffySe81g3tkG&search_mode=AdvancedSearch&update_back2search_link_param=yes) | (TI=(((propensity score* OR match* NEAR/1 (control*)) OR (match* NEAR/1 (compar*))) OR (assessment only OR comparison samp* OR propensity match*))) *AND* **DOCUMENT TYPES:** (Article)  *Indexes=SCI-EXPANDED, SSCI Timespan=All years* |
| # 42 | [26,437](http://apps.webofknowledge.com/summary.do?product=WOS&doc=1&qid=71&SID=R2hHC4XffySe81g3tkG&search_mode=AdvancedSearch&update_back2search_link_param=yes) | (TI=(assign* NEAR/3 (subject* OR patient*)) OR TS=(assign* NEAR/3 (subject* OR patient*))) *AND* **DOCUMENT TYPES:** (Article)  *Indexes=SCI-EXPANDED, SSCI Timespan=All years* |
| # 41 | [3,119,888](http://apps.webofknowledge.com/summary.do?product=WOS&doc=1&qid=70&SID=R2hHC4XffySe81g3tkG&search_mode=AdvancedSearch&update_back2search_link_param=yes) | ((TI=(quasi-experiment* OR quasiexperiment* OR experiment*) OR TS=(quasi-experiment* OR quasiexperiment* OR experiment*))) *AND***DOCUMENT TYPES:** (Article)  *Indexes=SCI-EXPANDED, SSCI Timespan=All years* |
| # 40 | [23,465](http://apps.webofknowledge.com/summary.do?product=WOS&doc=1&qid=69&SID=R2hHC4XffySe81g3tkG&search_mode=AdvancedSearch&update_back2search_link_param=yes) | (TI=(Intervention* NEAR/1 stud*) OR TS=(Intervention* NEAR/1 stud*)) *AND* **DOCUMENT TYPES:** (Article)  *Indexes=SCI-EXPANDED, SSCI Timespan=All years* |
| # 39 | [257,894](http://apps.webofknowledge.com/summary.do?product=WOS&doc=1&qid=68&SID=R2hHC4XffySe81g3tkG&search_mode=AdvancedSearch&update_back2search_link_param=yes) | (TI=(retrospective) OR TS=(retrospective)) *AND* **DOCUMENT TYPES:** (Article)  *Indexes=SCI-EXPANDED, SSCI Timespan=All years* |
| # 38 | [197,210](http://apps.webofknowledge.com/summary.do?product=WOS&doc=1&qid=67&SID=R2hHC4XffySe81g3tkG&search_mode=AdvancedSearch&update_back2search_link_param=yes) | (TI=(prospective NEAR/2 stud*) OR TS=(prospective NEAR/2 stud*)) *AND* **DOCUMENT TYPES:** (Article)  *Indexes=SCI-EXPANDED, SSCI Timespan=All years* |
| # 37 | [112,838](http://apps.webofknowledge.com/summary.do?product=WOS&doc=1&qid=66&SID=R2hHC4XffySe81g3tkG&search_mode=AdvancedSearch&update_back2search_link_param=yes) | (TI=(observational) OR TS=(observational)) *AND* **DOCUMENT TYPES:** (Article)  *Indexes=SCI-EXPANDED, SSCI Timespan=All years* |
| # 36 | [241,374](http://apps.webofknowledge.com/summary.do?product=WOS&doc=1&qid=55&SID=R2hHC4XffySe81g3tkG&search_mode=AdvancedSearch&update_back2search_link_param=yes) | (TS=(longitudinal) OR TI=(longitudinal)) *AND* **DOCUMENT TYPES:** (Article)  *Indexes=SCI-EXPANDED, SSCI Timespan=All years* |
| # 35 | [902](http://apps.webofknowledge.com/summary.do?product=WOS&doc=1&qid=65&SID=R2hHC4XffySe81g3tkG&search_mode=AdvancedSearch&update_back2search_link_param=yes) | (TS=(followup NEAR/2 stud*) OR TI=(followup NEAR/2 stud*)) *AND* **DOCUMENT TYPES:** (Article)  *Indexes=SCI-EXPANDED, SSCI Timespan=All years* |
| # 34 | [51,058](http://apps.webofknowledge.com/summary.do?product=WOS&doc=1&qid=64&SID=R2hHC4XffySe81g3tkG&search_mode=AdvancedSearch&update_back2search_link_param=yes) | (TI=(epidemiologic* NEAR/2 stud*) OR TS=(epidemiologic* NEAR/2 stud*)) *AND* **DOCUMENT TYPES:** (Article OR Book OR Book Chapter)  *Indexes=SCI-EXPANDED, SSCI Timespan=All years* |
| # 33 | [203,960](http://apps.webofknowledge.com/summary.do?product=WOS&doc=1&qid=63&SID=R2hHC4XffySe81g3tkG&search_mode=AdvancedSearch&update_back2search_link_param=yes) | (TI=("cross sectional*") OR TS=(cross sectional*)) *AND* **DOCUMENT TYPES:** (Article OR Book OR Book Chapter)  *Indexes=SCI-EXPANDED, SSCI Timespan=All years* |
| # 32 | [369,309](http://apps.webofknowledge.com/summary.do?product=WOS&doc=1&qid=62&SID=R2hHC4XffySe81g3tkG&search_mode=AdvancedSearch&update_back2search_link_param=yes) | (TI=("case control*") OR TS=(case control*)) *AND* **DOCUMENT TYPES:** (Article OR Book OR Book Chapter)  *Indexes=SCI-EXPANDED, SSCI Timespan=All years* |
| # 31 | [1,195,611](http://apps.webofknowledge.com/summary.do?product=WOS&doc=1&qid=61&SID=R2hHC4XffySe81g3tkG&search_mode=AdvancedSearch&update_back2search_link_param=yes) | (TS=(case stud*) OR TI=(case stud*)) *AND* **DOCUMENT TYPES:** (Article OR Book OR Book Chapter)  *Indexes=SCI-EXPANDED, SSCI Timespan=All years* |
| # 30 | [335,595](http://apps.webofknowledge.com/summary.do?product=WOS&doc=1&qid=60&SID=R2hHC4XffySe81g3tkG&search_mode=AdvancedSearch&update_back2search_link_param=yes) | (TS=(cohort*) OR TI=(cohort*)) *AND* **DOCUMENT TYPES:** (Article OR Book OR Book Chapter)  *Indexes=SCI-EXPANDED, SSCI Timespan=All years* |
| # 29 | [3,017,666](http://apps.webofknowledge.com/summary.do?product=WOS&doc=1&qid=59&SID=R2hHC4XffySe81g3tkG&search_mode=AdvancedSearch&update_back2search_link_param=yes) | (TS=(group*) OR TI=(group*)) *AND* **DOCUMENT TYPES:** (Article OR Book OR Book Chapter)  *Indexes=SCI-EXPANDED, SSCI Timespan=All years* |
| # 28 | [832,708](http://apps.webofknowledge.com/summary.do?product=WOS&doc=1&qid=58&SID=R2hHC4XffySe81g3tkG&search_mode=AdvancedSearch&update_back2search_link_param=yes) | (TS=(trial*) OR TI=(trial*)) *AND* **DOCUMENT TYPES:** (Article OR Book OR Book Chapter)  *Indexes=SCI-EXPANDED, SSCI Timespan=All years* |
| # 27 | [149,180](http://apps.webofknowledge.com/summary.do?product=WOS&doc=1&qid=57&SID=R2hHC4XffySe81g3tkG&search_mode=AdvancedSearch&update_back2search_link_param=yes) | (TS=(placebo) OR TI=(placebo)) *AND* **DOCUMENT TYPES:** (Article OR Book OR Book Chapter)  *Indexes=SCI-EXPANDED, SSCI Timespan=All years* |
| # 26 | [1,043,710](http://apps.webofknowledge.com/summary.do?product=WOS&doc=1&qid=56&SID=R2hHC4XffySe81g3tkG&search_mode=AdvancedSearch&update_back2search_link_param=yes) | (TS=(random*) OR TI=(random*)) *AND* **DOCUMENT TYPES:** (Article OR Book OR Book Chapter)  *Indexes=SCI-EXPANDED, SSCI Timespan=All years* |
| # 25 | [4,687](http://apps.webofknowledge.com/summary.do?product=WOS&doc=1&qid=38&SID=R2hHC4XffySe81g3tkG&search_mode=AdvancedSearch&update_back2search_link_param=yes) | (#24 AND #23 AND #22) *AND* **DOCUMENT TYPES:** (Article OR Book OR Book Chapter)  *Indexes=SCI-EXPANDED, SSCI Timespan=All years* |
| # 24 | [732,052](http://apps.webofknowledge.com/summary.do?product=WOS&doc=1&qid=37&SID=R2hHC4XffySe81g3tkG&search_mode=AdvancedSearch&update_back2search_link_param=yes) | (#21 OR #20 OR #19 OR #18 OR #17) *AND* **DOCUMENT TYPES:** (Article OR Book OR Book Chapter)  *Indexes=SCI-EXPANDED, SSCI Timespan=All years* |
| # 23 | [407,393](http://apps.webofknowledge.com/summary.do?product=WOS&doc=1&qid=36&SID=R2hHC4XffySe81g3tkG&search_mode=AdvancedSearch&update_back2search_link_param=yes) | (#16 OR #15 OR #14 OR #13 OR #12 OR #11 OR #10 OR #9) *AND* **DOCUMENT TYPES:** (Article OR Book OR Book Chapter)  *Indexes=SCI-EXPANDED, SSCI Timespan=All years* |
| # 22 | [110,160](http://apps.webofknowledge.com/summary.do?product=WOS&doc=1&qid=35&SID=R2hHC4XffySe81g3tkG&search_mode=AdvancedSearch&update_back2search_link_param=yes) | (#8 OR #7 OR #6 OR #5 OR #4 OR #3 OR #2 OR #1) *AND* **DOCUMENT TYPES:** (Article OR Book OR Book Chapter)  *Indexes=SCI-EXPANDED, SSCI Timespan=All years* |
| # 21 | [3,737](http://apps.webofknowledge.com/summary.do?product=WOS&doc=1&qid=34&SID=R2hHC4XffySe81g3tkG&search_mode=AdvancedSearch&update_back2search_link_param=yes) | ((TI=((transfer* NEAR/2 (effect))))) *AND* **DOCUMENT TYPES:** (Article OR Book OR Book Chapter)  *Indexes=SCI-EXPANDED, SSCI Timespan=All years* |
| # 20 | [1,455](http://apps.webofknowledge.com/summary.do?product=WOS&doc=1&qid=33&SID=R2hHC4XffySe81g3tkG&search_mode=AdvancedSearch&update_back2search_link_param=yes) | ((TS=(“math* educat*"))) *AND* **DOCUMENT TYPES:** (Article OR Book OR Book Chapter)  *Indexes=SCI-EXPANDED, SSCI Timespan=All years* |
| # 19 | [290,027](http://apps.webofknowledge.com/summary.do?product=WOS&doc=1&qid=32&SID=R2hHC4XffySe81g3tkG&search_mode=AdvancedSearch&update_back2search_link_param=yes) | ((TS=(“math*”))) *AND* **DOCUMENT TYPES:** (Article OR Book OR Book Chapter)  *Indexes=SCI-EXPANDED, SSCI Timespan=All years* |
| # 18 | [23,877](http://apps.webofknowledge.com/summary.do?product=WOS&doc=1&qid=31&SID=R2hHC4XffySe81g3tkG&search_mode=AdvancedSearch&update_back2search_link_param=yes) | ((TS=("literac*"))) *AND* **DOCUMENT TYPES:** (Article OR Book OR Book Chapter)  *Indexes=SCI-EXPANDED, SSCI Timespan=All years* |
| # 17 | [429,490](http://apps.webofknowledge.com/summary.do?product=WOS&doc=1&qid=30&SID=R2hHC4XffySe81g3tkG&search_mode=AdvancedSearch&update_back2search_link_param=yes) | ((TS=("read*"))) *AND* **DOCUMENT TYPES:** (Article OR Book OR Book Chapter)  *Indexes=SCI-EXPANDED, SSCI Timespan=All years* |
| # 16 | [758](http://apps.webofknowledge.com/summary.do?product=WOS&doc=1&qid=28&SID=R2hHC4XffySe81g3tkG&search_mode=AdvancedSearch&update_back2search_link_param=yes) | ((TI=((Intellect* NEAR/2 (develop*))))) *AND* **DOCUMENT TYPES:** (Article OR Book OR Book Chapter)  *Indexes=SCI-EXPANDED, SSCI Timespan=All years* |
| # 15 | [1,761](http://apps.webofknowledge.com/summary.do?product=WOS&doc=1&qid=27&SID=R2hHC4XffySe81g3tkG&search_mode=AdvancedSearch&update_back2search_link_param=yes) | ((TI=((school* NEAR/1 (performan* or achiev*))))) *AND* **DOCUMENT TYPES:** (Article OR Book OR Book Chapter)  *Indexes=SCI-EXPANDED, SSCI Timespan=All years* |
| # 14 | [4,918](http://apps.webofknowledge.com/summary.do?product=WOS&doc=1&qid=26&SID=R2hHC4XffySe81g3tkG&search_mode=AdvancedSearch&update_back2search_link_param=yes) | ((TI=((academic* NEAR/2 (performance* or achiev* or abilit* or outcome*))))) *AND* **DOCUMENT TYPES:** (Article OR Book OR Book Chapter)  *Indexes=SCI-EXPANDED, SSCI Timespan=All years* |
| # 13 | [7,413](http://apps.webofknowledge.com/summary.do?product=WOS&doc=1&qid=24&SID=R2hHC4XffySe81g3tkG&search_mode=AdvancedSearch&update_back2search_link_param=yes) | ((TI=((learn* NEAR/2 (disab* or problem*))))) *AND* **DOCUMENT TYPES:** (Article OR Book OR Book Chapter)  *Indexes=SCI-EXPANDED, SSCI Timespan=All years* |
| # 12 | [10,480](http://apps.webofknowledge.com/summary.do?product=WOS&doc=1&qid=22&SID=R2hHC4XffySe81g3tkG&search_mode=AdvancedSearch&update_back2search_link_param=yes) | ((TS=("learn* disabilit*"))) *AND* **DOCUMENT TYPES:** (Article OR Book OR Book Chapter)  *Indexes=SCI-EXPANDED, SSCI Timespan=All years* |
| # 11 | [396,189](http://apps.webofknowledge.com/summary.do?product=WOS&doc=1&qid=21&SID=R2hHC4XffySe81g3tkG&search_mode=AdvancedSearch&update_back2search_link_param=yes) | ((TS=("learn*"))) *AND* **DOCUMENT TYPES:** (Article OR Book OR Book Chapter)  *Indexes=SCI-EXPANDED, SSCI Timespan=All years* |
| # 10 | [26](http://apps.webofknowledge.com/summary.do?product=WOS&doc=1&qid=20&SID=R2hHC4XffySe81g3tkG&search_mode=AdvancedSearch&update_back2search_link_param=yes) | ((TS=("academic* achieve* gap*"))) *AND* **DOCUMENT TYPES:** (Article OR Book OR Book Chapter)  *Indexes=SCI-EXPANDED, SSCI Timespan=All years* |
| # 9 | [9,853](http://apps.webofknowledge.com/summary.do?product=WOS&doc=1&qid=19&SID=R2hHC4XffySe81g3tkG&search_mode=AdvancedSearch&update_back2search_link_param=yes) | ((TS=("academic* achieve*"))) *AND* **DOCUMENT TYPES:** (Article OR Book OR Book Chapter)  *Indexes=SCI-EXPANDED, SSCI Timespan=All years* |
| # 8 | [96,311](http://apps.webofknowledge.com/summary.do?product=WOS&doc=1&qid=18&SID=R2hHC4XffySe81g3tkG&search_mode=AdvancedSearch&update_back2search_link_param=yes) | ((TI=((Student* OR pupil* NEAR/3 (Learn*) NEAR/2 (disab* OR problem*))))) *AND* **DOCUMENT TYPES:** (Article OR Book OR Book Chapter)  *Indexes=SCI-EXPANDED, SSCI Timespan=All years* |
| # 7 | [18,278](http://apps.webofknowledge.com/summary.do?product=WOS&doc=1&qid=16&SID=R2hHC4XffySe81g3tkG&search_mode=AdvancedSearch&update_back2search_link_param=yes) | ((TI=(((Primary NEAR/1 (School)) NEAR/3 (Student* OR pupil*)) OR ((Elementary NEAR/1 (School)) NEAR/3 (Student* OR pupil*)) OR ((Secondary NEAR/1 (school*)) OR (high NEAR/2 (school*)) OR (middle NEAR/2 (School*)) NEAR/3 (student* OR pupil*))))) *AND* **DOCUMENT TYPES:** (Article OR Book OR Book Chapter)  *Indexes=SCI-EXPANDED, SSCI Timespan=All years* |
| # 6 | [3,323](http://apps.webofknowledge.com/summary.do?product=WOS&doc=1&qid=15&SID=R2hHC4XffySe81g3tkG&search_mode=AdvancedSearch&update_back2search_link_param=yes) | ((TI=((Underachiev* OR Under NEAR/1 (achiev* OR lowachiev*)) OR (low NEAR/2 (achiev*)) OR (Low NEAR/1 (perform* OR lowperform*)) OR ((at-risk OR “at” NEAR/1 (risk)) NEAR/1 (student* OR pupil*)) OR ((high-risk OR high NEAR/1 (risk)) NEAR/1 (student* OR pupil*)) OR ((Special NEAR/1 (Need*)) NEAR/1 (Student* OR pupil*)) OR ((Low NEAR/1 (income)) NEAR/1 (student* OR pupil*))))) *AND* **DOCUMENT TYPES:** (Article OR Book OR Book Chapter)  *Indexes=SCI-EXPANDED, SSCI Timespan=All years* |
| # 5 | [96,311](http://apps.webofknowledge.com/summary.do?product=WOS&doc=1&qid=14&SID=R2hHC4XffySe81g3tkG&search_mode=AdvancedSearch&update_back2search_link_param=yes) | ((TI=((Student* OR pupil* NEAR/3 (Learn*) NEAR/2 (disab* or problem*))))) *AND* **DOCUMENT TYPES:** (Article OR Book OR Book Chapter)  *Indexes=SCI-EXPANDED, SSCI Timespan=All years* |
| # 4 | [491](http://apps.webofknowledge.com/summary.do?product=WOS&doc=1&qid=12&SID=R2hHC4XffySe81g3tkG&search_mode=AdvancedSearch&update_back2search_link_param=yes) | ((TI=(("Foster Care*") AND child*))) *AND* **DOCUMENT TYPES:** (Article OR Book OR Book Chapter)  *Indexes=SCI-EXPANDED, SSCI Timespan=All years* |
| # 3 | [3](http://apps.webofknowledge.com/summary.do?product=WOS&doc=1&qid=10&SID=R2hHC4XffySe81g3tkG&search_mode=AdvancedSearch&update_back2search_link_param=yes) | (TI=((Child* NEAR/2 (placed) NEAR/1 (care)))) *AND* **DOCUMENT TYPES:** (Article OR Book OR Book Chapter)  *Indexes=SCI-EXPANDED, SSCI Timespan=All years* |
| # 2 | [18,278](http://apps.webofknowledge.com/summary.do?product=WOS&doc=1&qid=5&SID=R2hHC4XffySe81g3tkG&search_mode=AdvancedSearch&update_back2search_link_param=yes) | ((TI=(((Primary NEAR/1 (School)) NEAR/3 (Student* or pupil*)) OR ((Elementary NEAR/1 (School)) NEAR/3 (Student* or pupil*)) OR ((Secondary NEAR/1 (school*)) OR (high NEAR/2 (school*)) OR (middle NEAR/2 (School*)) NEAR/3 (student* OR pupil*))))) *AND* **DOCUMENT TYPES:**(Article OR Book OR Book Chapter)  *Indexes=SCI-EXPANDED, SSCI Timespan=All years* |
| # 1 | [3,323](http://apps.webofknowledge.com/summary.do?product=WOS&doc=1&qid=4&SID=R2hHC4XffySe81g3tkG&search_mode=AdvancedSearch&update_back2search_link_param=yes) | (TI=((Underachiev* OR Under NEAR/1 (achiev* or lowachiev*)) OR (low NEAR/2 (achiev*)) OR (Low NEAR/1 (perform* OR lowperform*)) OR ((at-risk OR "at" NEAR/1 (risk)) NEAR/1 (student* or pupil*)) OR ((high-risk or high NEAR/1 (risk)) NEAR/1 (student* or pupil*)) OR ((Special NEAR/1 (Need*)) NEAR/1 (Student* or pupil*)) OR ((Low NEAR/1 (income)) NEAR/1 (student* or pupil*)))) *AND* **DOCUMENT TYPES:** (Article OR Book OR Book Chapter)  *Indexes=SCI-EXPANDED, SSCI Timespan=All years* |

**PubMed &Medline**

Searched March 2016. The search in 2018 yielded no new results.

| **Search** | **Query** | **Results** |
| --- | --- | --- |
| 57 | 34 and 56 | 323 |
| 56 | 35 or 36 or 37 or 38 or 39 or 40 or 41 or 42 or 43 or 44 or 45 or 46 or 47 or 48 or 49 or 50 or 51 or 52 or 53 or 54 or 55 | 5063720 |
| 55 | ((((regression adj1 discontinuity) or difference-in-difference* or event) adj1 stud*) or interrupted time serie* or instrumental variable* or waitlist control*).ti. or ((((regression adj1 discontinuity) or difference-in-difference* or event) adj1 stud*) or interrupted time serie* or instrumental variable* or waitlist control*).ab. | 2881 |
| 54 | ((control adj5 case) or (control adj5 subject*) or (control adj5 group*) or (control adj5 patient*) or (control adj5 intervention)).ti. or ((control adj5 case) or (control adj5 subject*) or (control adj5 group*) or (control adj5 patient*) or (control adj5 intervention)).ab. | 550849 |
| 53 | ((treatment adj5 case) or (treatment adj5 subject*) or (treatment adj5 group*) or (treatment adj5 patient*) or (treatment adj5 intervention)).ti. or ((treatment adj5 case) or (treatment adj5 subject*) or (treatment adj5 group*) or (treatment adj5 patient*) or (treatment adj5 intervention)).ab. | 523471 |
| 52 | ((experiment* adj5 case) or (experiment* adj5 subject*) or (experiment* adj5 group*) or (experiment* adj5 patient*) or (experiment* adj5 intervention)).ti. or ((experiment* adj5 case) or (experiment* adj5 subject*) or (experiment* adj5 group*) or (experiment* adj5 patient*) or (experiment* adj5 intervention)).ab. | 79366 |
| 51 | ((intervention adj5 case) or (intervention adj5 subject*) or (intervention adj5 group*) or (intervention adj5 patient*)).ti. or ((intervention adj5 case) or (intervention adj5 subject*) or (intervention adj5 group*) or (intervention adj5 patient*)).ab. | 65864 |
| 50 | ((assign* adj5 case) or (assign* adj5 subject*) or (assign* adj5 group*) or (assign* adj5 patient*) or (assign* adj5 intervention)).ti. or ((assign* adj5 case) or (assign* adj5 subject*) or (assign* adj5 group*) or (assign* adj5 patient*) or (assign* adj5 intervention)).ab. | 62637 |
| 49 | (quasi-experiment* or quasiexperiment* or Propensity score* or (compar* adj1 group*) or (match* adj1 control*) or (match* adj1 group*) or (match* adj1 compar*) or experiment* trial* or experiment* design* or experiment* method* or experiment* stud* or experiment* evaluation* or experiment* test* or experiment* assessment* or assessment only or (comparison adj1 samp*) or propensity match* or (Between adj1 group*)).ti. or (quasi-experiment* or quasiexperiment* or Propensity score* or (compar* adj1 group*) or (match* adj1 control*) or (match* adj1 group*) or (match* adj1 compar*) or experiment* trial* or experiment* design* or experiment* method* or experiment* stud* or experiment* evaluation* or experiment* test* or experiment* assessment* or assessment only or (comparison adj1 samp*) or propensity match* or (Between adj1 group*)).ab. | 338123 |
| 48 | ((random* adj2 trial*) or RCT).ti. or ((random* adj2 trial*) or RCT).ab. | 176530 |
| 47 | (Non-random* or nonradom* or (non adj1 random*)).ti. or (Non-random* or nonradom* or (non adj1 random*)).ab. | 11689 |
| 46 | (Propensity score* or (match* adj1 control*) or (match* adj1 compar*) or assessment only or comparison samp* or propensity match*).ti. or (Propensity score* or (match* adj1 control*) or (match* adj1 compar*) or assessment only or comparison samp* or propensity match*).ab. | 74039 |
| 45 | (assign adj3 (subject* or patient*)).ti. or (assign adj3 (subject* or patient*)).ab. | 447 |
| 44 | (quasi-experiment* or quasiexperiment* or experiment*).ti. or (quasi-experiment* or quasiexperiment* or experiment*).ab. | 1357128 |
| 43 | (intervention* adj1 stud*).ti. or (intervention* adj1 stud*).ab. | 16512 |
| 42 | retrospective.ti. or retrospective.ab. | 288643 |
| 41 | (prospective adj2 stud*).ti. or (prospective adj2 stud*).ab. | 193506 |
| 40 | longitudinal.ti. or longitudinal.ab. or observational.ti. or observational.ab. | 218571 |
| 39 | (((epidemiologic adj2 study).ti. or epidemiologic.mp.) adj2 study.ab.) or ((followup or follow up) adj2 stud*).ti. or ((followup or follow up) adj2 stud*).ab. [mp=title, abstract, original title, name of substance word, subject heading word, keyword heading word, protocol supplementary concept word, rare disease supplementary concept word, unique identifier] | 45099 |
| 38 | cross sectional.ti. or cross sectional.ab. | 175279 |
| 37 | (case control or cohort).ti. or case control.ab. or cohort.ab. | 334639 |
| 36 | (cohort analysis or case studies).hw,kf,ui. | 13018 |
| 35 | (randomized or placebo or (randomly or trial*) or group*).ab. | 2929343 |
| 34 | 16 and 27 and 33 | 787 |
| 33 | 28 or 29 or 30 or 31 or 32 | 107384 |
| 32 | (transfer adj2 effect*).af. | 2559 |
| 31 | ((numerac* or math*) adj1 learn*).af. | 1233 |
| 30 | Literacy.hw,kf,ui. | 4390 |
| 29 | Reading.hw,kf,ui. | 43205 |
| 28 | Language.hw,kf,ui. | 60377 |
| 27 | 17 or 18 or 19 or 20 or 21 or 22 or 23 or 24 or 25 or 26 | 328504 |
| 26 | (intellect adj2 develop*).af. | 13 |
| 25 | (school* adj1 (performan* or achiev*)).af. | 4038 |
| 24 | (academic adj2 (performanc* or achiev* or abilit* or outcome*)).af. | 6288 |
| 23 | (learn* adj2 (disab* or problem*)).af. | 18651 |
| 22 | Intelligence.hw,kf,ui. | 52794 |
| 21 | Learning.hw,kf,ui. | 141813 |
| 20 | Learning Disorders.hw,kf,ui. | 13014 |
| 19 | Students.hw,kf,ui. | 91367 |
| 18 | Child Development.hw,kf,ui. | 43742 |
| 17 | achievement.hw,kf,ui. | 13876 |
| 16 | 9 or 10 or 11 or 12 or 13 or 14 or 15 | 100681 |
| 15 | ((student* or pupil*) adj3 (learn adj2 (disab* or problem*))).af. | 9 |
| 14 | foster home care.hw. and child*.af. | 2738 |
| 13 | (((secondary adj 1 school*) or (high adj2 school) or (middle adj1 school)) adj3 (student* or pupil*)).af. | 7980 |
| 12 | school*.hw. | 86001 |
| 11 | (elementary adj1 school* adj3 (student* or pupil*)).af. | 835 |
| 10 | (primary adj1 school* adj3 (student* or pupil*)).af. | 697 |
| 9 | 1 or 2 or 3 or 4 or 5 or 6 or 7 or 8 | 5336 |
| 8 | (low adj1 income adj1 (student* or pupil*)).af. | 59 |
| 7 | (special adj1 need* adj1 (student* or pupil*)).af. | 25 |
| 6 | ((high-risk or high) adj1 risk adj1 (student* or pupil*)).af. | 105 |
| 5 | ((at-risk or at) adj1 risk adj1 (student* or pupil*)).af. | 424 |
| 4 | (low adj1 perform*).af. | 1507 |
| 3 | (low adj1 achiev*).af. | 948 |
| 2 | (under adj1 achiev*).af. | 1500 |
| 1 | (underachiev* or lowachieve* or lowperform).af. | 845 |

**Dissertations and Thesis A&I**

Searched March 2016. Search was not updated in 2018 due to limitation of access.

The same search string was used in the ProQuest platform to search CBCA Education, which resulted in 97 hits, and Australian Education Index, which resulted in 85 hits.

| **Search** | **Search** | **Results** |
| --- | --- | --- |
| Set#: S42 | S18 AND S41 limited to 1980-2016 | 786 |
| Set#: S41 | S19 OR S20 OR S21 OR S22 OR S23 OR S24 OR S25 OR S26 OR S27 OR S28 OR S29 OR S30 OR S31 OR S32 OR S33 OR S34 OR S35 OR S36 OR S37 OR S38 OR S39 OR S40 | 2,082,703 |
| Set#: S40 | (TI(regression NEAR/1 (discontinuity)) OR TI(difference-in-difference*) OR TI(event NEAR/1 stud*) OR TI( interrupted time serie*) OR TI(instrumental variable*) OR TI(waitlist control*)) OR (AB(regression NEAR/1 (discontinuity)) OR AB(difference-in-difference*) OR AB(event NEAR/1 stud*) OR AB(interrupted time serie*) OR AB(instrumental variable*) OR AB(waitlist control*)) | 7,942 |
| Set#: S39 | TI(control NEAR/5 (case)) OR TI(control NEAR/5 (subject*)) OR TI(control NEAR/5 (group*)) OR TI(control NEAR/5 (patient*)) OR TI(control NEAR/5 (intervention)) OR AB(control NEAR/5 (case)) OR AB(control NEAR/5 (subject*)) OR AB(control NEAR/5 (group*)) OR AB(control NEAR/5 (patient*)) OR AB(control NEAR/5 (intervention)) | 64,842 |
| Set#: S38 | TI(treatment NEAR/5 (case)) OR TI(treatment NEAR/5 (subject*)) OR TI(treatment NEAR/5 (group*)) OR TI(treatment NEAR/5 (patient*)) OR TI(treatment NEAR/5 (intervention)) OR AB(treatment NEAR/5 (case)) OR AB(treatment NEAR/5 (subject*)) OR AB(treatment NEAR/5 (group*)) OR AB(treatment NEAR/5 (patient*)) OR AB(treatment NEAR/5 (intervention)) | 41,096 |
| Set#: S37 | TI(experiment* NEAR/5 (case*)) OR TI(experiment* NEAR/5 (subject*)) OR TI(experiment* NEAR/5 (group*)) OR TI(experiment* NEAR/5 (patient*)) OR (experiment* NEAR/5 (intervention)) OR AB(experiment* NEAR/5 (case*)) OR AB(experiment* NEAR/5 (subject*)) OR AB(experiment* NEAR/5 (group*)) OR AB(experiment* NEAR/5 (patient*)) OR AB(experiment* NEAR/5 (intervention*)) | 56901 |
| Set#: S36 | TI(intervention NEAR/5 (case*)) OR TI(intervention NEAR/5 (subject*)) OR TI(intervention NEAR/5 (group*)) OR TI(intervention NEAR/5 (patient*)) OR AB(intervention NEAR/5 (case*)) OR AB(intervention NEAR/5 (subject*)) OR AB(intervention NEAR/5 (group*)) OR AB(intervention NEAR/5 (patient*)) | 12,588 |
| Set#: S35 | AB(assign* NEAR/5 (case*)) OR AB(assign* NEAR/5 (subject*)) OR AB(assign* NEAR/5 (group*)) OR AB(assign* NEAR/5 (patient*)) OR AB(assign* NEAR/5 (intervention*)) OR AB(assign* NEAR/5 (case*)) OR AB(assign* NEAR/5 (subject*)) OR AB(assign* NEAR/5 (group*)) OR AB(assign* NEAR/5 (patient*)) OR AB(assign* NEAR/5 (intervention*)) | 18,102 |
| Set#: S34 | TI(quasi-experiment* OR quasiexperiment* OR Propensity score* OR (compar* NEAR/1 (group*))) OR (match* NEAR/1 (control*)) OR (match* NEAR/1 (group*)) OR (match* NEAR/1 (compar*)) OR (experiment* trial* OR experiment* design* OR experiment* method* OR experiment* stud* OR experiment* evaluation* OR experiment* test* OR experiment* assessment* OR assessment only OR (comparison NEAR/1 (samp*))) OR propensity match* OR (Between NEAR/1 (group*)) OR AB(quasi-experiment* OR quasiexperiment* OR Propensity score* OR (compar* NEAR/1 (group*))) OR (match* NEAR/1 (control*)) OR (match* NEAR/1 (group*)) OR (match* NEAR/1 (compar*)) OR (experiment* trial* OR experiment* design* OR experiment* method* OR experiment* stud* OR experiment* evaluation* OR experiment* test* OR experiment*assessment* OR assessment only OR (comparison NEAR/1 (samp*))) OR (propensity match* OR (Between NEAR/1 (group*))) | 1,767,065 |
| Set#: S33 | TI(random* NEAR/2 (trial* OR RCT)) OR AB(random* NEAR/2 (trial* OR RCT)) | 4,915 |
| Set#: S32 | TI(Non-random* OR nonradom* OR (non NEAR/1 random*)) OR AB(Non-random* OR Nonrandom* OR (non NEAR/1 random*)) | 4,150 |
| Set#: S31 | TI(Propensity score* OR match* NEAR/1 (control*)) OR (match* NEAR/1 ( compar*)) OR (assessment only OR comparison samp* OR propensity match*) OR AB(Propensity score* OR (match* NEAR/1 control*)) OR (match* NEAR/1 compar*) OR (assessment only OR comparison samp* OR propensity match*) | 1,437,399 |
| Set#: S30 | AB(assign* NEAR/3 (subject* OR patient*)) | 5,488 |
| Set#: S29 | TI(assign* NEAR/3 (subject* OR patient*)) | 22 |
| Set#: S28 | TI(quasi-experiment* OR quasiexperiment* OR experiment*) OR AB(quasi-experiment* OR quasiexperiment* OR experiment*) | 473,696 |
| Set#: S27 | TI(Intervention* NEAR/1 Stud*) OR AB(Intervention* NEAR/1 Stud*) | 4,500 |
| Set#: S26 | (TI(prospective NEAR/2 study) OR AB(prospective NEAR/2 study) OR TI(retrospective) OR AB(retrospective)) | 15,836 |
| Set#: S25 | TI(longitudinal) OR AB(longitudinal) OR TI(observational) OR AB(observational) | 48,855 |
| Set#: S24 | TI(followup NEAR/2 study) OR AB(followup NEAR/2 study) | 97 |
| Set#: S23 | TI(epidemiologic NEAR/2 study) OR AB(epidemiologic NEAR/2 study) | 1,434 |
| Set#: S22 | TI(cross sectional) or AB(cross sectional) | 22,979 |
| Set#: S21 | TI(case control) OR AB(case control) OR TI(cohort) OR AB(cohort) | 64,539 |
| Set#: S20 | SU(“Cohort”) OR SU(“case studies”) | 2,078 |
| Set#: S19 | (AB(randomized) OR AB(placebo) OR AB(randomly) OR AB(trial) OR AB(groups)) | 518,004 |
| Set#: S18 | S8 AND S13 AND | 842 |
| Set#: S17 | S14 OR S15 OR S16 | 116,044 |
| Set#: S16 | ti((transfer* NEAR/2 (effect))) | 452 |
| Set#: S15 | SU(“Mathematics”) OR SU(“Mathematics education”) | 94,310 |
| Set#: S14 | SU("Reading") OR SU("literacy") | 21,826 |
| Set#: S13 | S9 OR S10 OR S11 OR S12 | 16,182 |
| Set#: S12 | ti((Intellect* NEAR/2 (develop*))) | 378 |
| Set#: S11 | ti((School NEAR/1 (performan* or achiev*))) | 1,702 |
| Set#: S10 | ti((Academic* NEAR/2 (performance* or achiev* or abilit* or outcome*))) | 8,298 |
| Set#: S9 | ti((Learn* NEAR/2 (disab* or Problem*))) | 6,112 |
| Set#: S8 | S6 OR S7 | 164,850 |
| Set#: S7 | (SU("Academic Achievement") OR SU("academic achievement gaps") OR SU("Learning") OR SU("Learning Disabilities")) | 34,380 |
| Set#: S6 | ti((Underachiev* OR Under NEAR/1 (achiev* OR lowachiev*)) OR (low NEAR/2 (achiev*)) OR (Low NEAR/1 (perform* OR lowperform*)) OR ((at-risk OR at NEAR/1 (risk)) NEAR/1 (student* OR pupil*)) OR ((high-risk OR high NEAR/1 (risk)) NEAR/1 (student* OR pupil*)) OR ((Special NEAR/1 (Need*)) NEAR/1 (Student* OR pupil*)) OR ((Low NEAR/1 (income)) NEAR/1 (student* OR pupil*))) OR ti(((Primary NEAR/1 (School)) NEAR/3 (Student* OR pupil*)) OR ((Elementary NEAR/1 (School)) NEAR/3 (Student* OR pupil*)) OR ((Secondary NEAR/1 (school*)) OR (high NEAR/2 (school*)) OR (middle NEAR/2 (School*)) NEAR/3 (student* OR pupil*))) OR ti((Child* NEAR/2 (placed) NEAR/1 (care))) OR ((SU("Foster Care")) AND child*) OR ti((Student* OR pupil* NEAR/3 (Learn*) NEAR/2 (disab* OR problem*))) | 139,796 |
| Set#: S5 | ti((Student* OR pupil* NEAR/3 (Learn*) NEAR/2 (disab* or problem*))) | 107,191 |
| Set#: S4 | ((SU("Foster Care")) AND child*) | 678 |
| Set#: S3 | ti((Child* NEAR/2 (placed) NEAR/1 (care)) ) | 2 |
| Set#: S2 | ti(((Primary NEAR/1 (School)) NEAR/3 (Student* or pupil*)) OR ((Elementary NEAR/1 (School)) NEAR/3 (Student* or pupil*)) OR ((Secondary NEAR/1 (school*)) OR (high NEAR/2 (school*)) OR (middle NEAR/2 (School*)) NEAR/3 (student* OR pupil*))) | 45,355 |
| Set#: S1 | ti((Underachiev* OR Under NEAR/1 (achiev* or lowachiev*)) OR (low NEAR/2 (achiev*)) OR (Low NEAR/1 (perform* OR lowperform*)) OR ((at-risk OR at NEAR/1 (risk)) NEAR/1 (student* or pupil*)) OR ((high-risk or high NEAR/1 (risk)) NEAR/1 (student* or pupil*)) OR ((Special NEAR/1 (Need*)) NEAR/1 (Student* or pupil*)) OR ((Low NEAR/1 (income)) NEAR/1 (student* or pupil*))) | 3,766 |

**Grey Literature and Searches on Other Resources**

**DIVA**

(school* OR primary school* OR elementary school* OR secondary school* OR high school* OR middle school* OR student* OR pupil* OR child* OR lowachiev* OR underachiev*) AND (performance* OR academic* OR achieve* OR abilit* OR learn* OR outcome* OR intell* OR read* OR literac* OR math* OR develop* OR numerac*) AND (random* OR placebo OR RCT* OR trial* OR group* OR quasi-experiment* OR cohort* OR case* intervent* OR experiment* OR study* OR evaluat* OR treatment* OR longitudinal*)

Hits: 102

**CRISTIN**

180 hits after de-duplication.

school* achieve* = 11 hits.

school* perform* = 8 hits

school* academic* = 3 hits

school* learn* = 20 hits

school* develop* = 33 hits

school* abilit* = 2 hits

student* achieve* = 8 hits.

student* perform*= 8 hits.

student* academic*= 9 hits.

student* learn* = 88 hits.

student* develop* = 26 hits

student* abilit* = 2 hits.

student* outcome* = 7 hits.

**Forskningsdatabasen**

Student* outcome* learn* school*

Hits: 109

**Thesis Canada**

Search was performed 29/1 2016. Search is limited to separate phrases. Studies were screened before downloading. Only relevant hits were downloaded.

Term(s) Searched: Select One "school*" AND Select One "achieve*" AND Language "english". Hits=11

Term(s) Searched: Select One "school*" AND Select One "student*" AND Language "english". Hits=92

Term(s) Searched: Select One "School*" AND Select One "learn*" AND Language "english". Hits=19

Term(s) Searched: Select One "student*" AND Select One "achieve*" AND Language "english". Hits=7

Term(s) Searched: Select One "student*" AND Select One "learn*" AND Language "english". Hits=13

Term(s) Searched: Select One "student*" AND Select One "outcome*" AND Language "english". Hits=3

Hits: 145

**Cochrane Library**

Search performed in”record title”.

(school* OR primary school* OR elementary school* OR secondary school* OR high school* OR middle school* OR student* OR pupil* OR child* OR lowachiev* OR underachiev*) AND (performance* OR academic* OR achieve* OR abilit* OR learn* OR outcome* OR intell* OR read* OR literac* OR math* OR develop* OR numerac*)

Hits: 37

**Social Care Online**

Search performed in “title”.

(school* OR primary school* OR elementary school* OR secondary school* OR high school* OR middle school* OR student* OR pupil* OR child* OR lowachiev* OR underachiev*) AND (performance* OR academic* OR achieve* OR abilit* OR learn* OR outcome* OR intell* OR read* OR literac* OR math* OR develop* OR numerac*)

Hits: 204

**Centre for reviews and dissemination (CRD)**

Search performed in “title”.

(school* OR primary school* OR elementary school* OR secondary school* OR high school* OR middle school* OR student* OR pupil* OR child* OR lowachiev* OR underachiev*) AND (performance* OR academic* OR achieve* OR abilit* OR learn* OR outcome* OR intell* OR read* OR literac* OR math* OR develop* OR numerac*)

Hits: 62

**Reviews used for citation tracking**

The reference lists of the following reviews were screened for relevant records. Relevant records were obtained and screened in full text.

Alfieri, L., Brooks, P. J., Aldrich, N. J., & Tenenbaum, H. R. (2011). Does discovery-based instruction enhance learning?. *Journal of Educational Psychology*, 103(1), 1-18.

Cheung, A. C., & Slavin, R. E. (2012). How features of educational technology applications affect student reading outcomes: A meta-analysis. *Educational Research Review*, 7(3), 198-215.

de Boer, H., Donker, A. S., & van der Werf, M. P. (2014). Effects of the attributes of educational interventions on students’ academic performance: A meta-analysis. *Review of Educational Research*, 84(4), 509-545.

Dexter, D. D., & Hughes, C. A. (2011). Graphic organizers and students with learning disabilities: A meta-analysis. *Learning Disability Quarterly*, 34(1), 51-72.

Dietrichson, J., Bøg, M., Filges, T., & Klint Jørgensen, A-M. (2017). Academic interventions for elementary and middle school students with low socioeconomic status: A systematic review and meta-analysis. *Review of Educational Research*, 87(2), 243-282.

Edmonds, M. S., Vaughn, S., Wexler, J., Reutebuch, C., Cable, A., Klingler Tackett, K., & Wick Schnakenberg, J. (2009). A synthesis of reading interventions and effects on reading comprehension outcomes for older struggling readers. *Review of Educational Research*, 79(1), 262-300.

Elbaum, B., Vaughn, S., Tejero Hughes, M., & Watson Moody, S. (2000). How effective are one-to-one tutoring programs in reading for elementary students at risk for reading failure? A meta-analysis of the intervention research. *Journal of Educational Psychology*, 92(4), 605-619.

Flynn, L. J., Zheng, X., & Swanson, H. L., (2012). Instructing struggling older readers: A selective meta-analysis of intervention research. *Learning Disabilities Research & Practice*, 27(1), 21-32.

Forsman, H., & Vinnerljung, B. (2012). Interventions aiming to improve school achievements of children in out-of-home care: A scoping review. *Children and Youth Services Review*, 34(6), 1084-1091.

Gersten, R., Chard, D. J., Jayanti, M., Baker, S. K., Morphy, P., & Flojo, P. (2009). Mathematics instruction for students with learning disabilities: A meta-analysis of instructional components. *Review of Educational Research*, 79(3), 1202-1242.

Goodwin, A. P., & Ahn, S. (2010). A meta-analysis of morphological interventions: Effects on literacy achievement of children with literacy difficulties. *Annals of dyslexia*, 60(2), 183-208.

Kyndt, E., Raes, E., Lismont, B., Timmers, F., Cascallar, E., & Dochy, F. (2013). A meta-analysis of the effects of face-to-face cooperative learning. Do recent studies falsify or verify earlier findings?. *Educational Research Review*, 10, 133-149.

Reljić, G., Ferring, D., & Martin, R. (2015). A Meta-Analysis on the effectiveness of bilingual programs in Europe. *Review of Educational Research*, 85(1), 92-128.

Ritter, G., Albin, G., Barnett, J., Blankenship, V., & Denny, G. (2006). The effectiveness of volunteer tutoring programs: A systematic review. *Campbell Systematic Reviews*, 7. DOI: 10.4073/csr.2006.7. Retrieved from <http://campbellcollaboration.org/lib/project/16/>.

Robinson, D. R., Schofield, J. W., & Steers-Wentzell, K. L. (2005). Peer and cross-age tutoring in math: outcomes and their design implications. *Educational Psychology Review*, 17(4), 327-362.

Scammaca, N. K., Roberts, G., Vaughn, S., & Stuebing, K. K. (2015). A meta-analysis of interventions for struggling readers in grades 4-12: 1980-2011. *Journal of Learning Disabilities*, 48(4), 369-390.

Slavin, R. E., Cheung, A., Groff, C., & Lake, C. (2008). Effective reading programs for middle and high schools: A best-evidence synthesis. *Reading Research Quarterly*, 43(3), 290-322.

Slavin, R. E., & Lake, C. (2008). Effective programs in elementary mathematics: A best-evidence synthesis. *Review of Educational Research*, 78(3), 427-515.

Slavin, R. E., Lake, C., & Groff, C. (2009). Effective programs in middle and high school mathematics: A best-evidence synthesis. *Review of Educational Research*, 79(2), 839-911.

Slavin, R. E., Lake, C., Chambers, B., Cheung, A., & Davis, S. (2009). Effective reading programs for the elementary grades: A best-evidence synthesis. *Review of Educational Research*, 79(4), 1391-1466.

Slavin, R. E., Lake, C., Davis, S., & Madden, N. A. (2011). Effective programs for struggling readers: A best-evidence synthesis. *Educational Research Review*, 6(1), 1-26.

Wilson, S., Tanner-Smith, E. E., Lipsey, M. W., Steinka-Fry, K., & Morrison, J. (2011). Dropout prevention and intervention programs: Effects on school completion and dropout among school-aged children and youth. *Campbell Systematic Reviews*, 7 Retrieved from <http://www.campbellcollaboration.org/lib/project/158/>.

Wanzek, J., Vaughn, S., Wexler, J., Swanson, E. A., Edmonds, M., & Kim, A-H., 2006. A synthesis of spelling and reading interventions and their effects on the spelling outcomes of students with LD. *Journal of Learning Disabilities*, 39(2), 528-543.

Wanzek, J., Vaughn, S., Wexler, J., Swanson, E. A., Edmonds, M., & Kim, A-H., 2006. A synthesis of spelling and reading interventions and their effects on the spelling outcomes of students with LD. *Journal of Learning Disabilities*, 39(2), 528-543.

**Risk of Bias Tool**

**Risk of bias table**

| **Item** | **Judgement^a^** | **Description** (quote from paper, or describe key information) |
| --- | --- | --- |
| 1. Sequence generation |  |  |
| 2. Allocation concealment |  |  |
| 3. Confounding^b,c^ |  |  |
| 4. Blinding?^b^ |  |  |
| 5. Incomplete outcome data addressed?^b^ |  |  |
| 6. Free of selective reporting?^b^ |  |  |
| 7. Free of other bias? |  |  |
| *8. A priori* protocol?^d^ |  |  |
| *9. A priori* analysis plan?^e^ |  |  |

^a^ Some items on low/high risk/unclear scale (double-line border), some on 5 point scale/unclear (single line border), some on yes/no/unclear scale (dashed border). For all items, record “unclear” if inadequate reporting prevents a judgement being made.

^b^ For each outcome in the study.

^c^ This item is only used for QESs. It is based on a list of confounders considered as important at the outset and defined in the protocol for the review (*assessment against worksheet*).

^d^ Did the researchers write a protocol defining the study population, intervention and comparator, primary and other outcomes, data collection methods, etc. in advance of starting the study?

^e^ Did the researchers have an analysis plan defining the primary and other outcomes, statistical methods, subgroup analyses, etc. in advance of starting the study?

**Risk of bias tool**

*Studies for which the risk of bias tool is Intended*

The risk of bias model is developed by Prof. Barnaby Reeves in association with the Cochrane Non-Randomised Studies Methods Group.^^[[1]](#footnote-1)^^ This model, an extension of the Cochrane Collaboration’s risk of bias tool, covers both risk of bias in randomised controlled trials (RCTs and QRCTs), but also risk of bias in non-randomised studies (QESs).

The point of departure for the risk of bias model is the Cochrane Handbook for Systematic Reviews of interventions (Higgins & Green, 2008). The existing Cochrane risk of bias tool needs elaboration when assessing non-randomised studies because, for non-randomised studies, particular attention should be paid to selection bias / risk of confounding. Additional items on confounding are used only for non-randomised studies (QESs) and are not used for randomised controlled trials (RCTs and QRCTs).

*Assessment of Risk of Bias*

Issues when using modified RoB tool to assess included non-randomised studies:

- Use existing principle: score judgement and provide information (preferably direct quote) to support judgement.
- Additional items on confounding used only for non-randomised studies (QESs).
- 5-point scale for some items (distinguish “unclear” from intermediate risk of bias).
- Keep in mind the general philosophy – assessment is not about whether researchers could have done better but about risk of bias; the assessment tool must be used in a standard way irrespective of the difficulty / circumstances of investigating the research question of interest or the study design used.
- Anchors: “1/No/low risk” of bias should correspond to a high quality RCT. “5/high risk” of bias should correspond to a risk of bias that means the findings should not be considered (too risky, too much bias, more likely to mislead than inform).

1. Sequence generation

- Low/high/unclear RoB item.
- Always high RoB (not random) for a non-randomised study.
- Might argue that this item is redundant for QES since it is always high – but it is important to include it in an RoB table (‘level playing field’ argument).

2. Allocation concealment

- Low/high/unclear RoB item.
- Potentially low RoB for a non-randomised study, e.g., quasi-randomised (too high RoB to sequence generation) but concealed (reviewer judges that the people making decisions about including participants didn’t know how allocation was being done, e.g., odd/even date of birth/hospital number).

3. RoB from confounding (additional item for QES; assess for each outcome)

- Assumes a pre-specified list of potential confounders defined in the protocol
- Low(1) / 2 / 3 / 4 / high(5) / unclear RoB item
- Judgement needs to factor in:
  - proportion of confounders (from pre-specified list) that were considered
  - whether most important confounders (from pre-specified list) were considered
  - resolution/precision with which confounders were measured
  - extent of imbalance between groups at baseline
  - care with which adjustment was done (typically a judgement about the statistical modeling carried out by authors)
- Low RoB requires that all important confounders are balanced at baseline (not primarily/not only a statistical judgement OR measured ‘well’ and ‘carefully’ controlled for in the analysis.

Assess against pre-specified worksheet. Reviewers will make an RoB judgement about each factor first and then ‘eyeball’ these for the judgement RoB table.

4. RoB from lack of blinding (assess for each outcome)

- Low(1) / 2 / 3 / 4 / high(5) / unclear RoB item
- Judgement needs to factor in:
  - nature of outcome (subjective / objective; source of information)
  - who was / was not blinded and the risk that those who were not blinded could introduce performance or detection bias
  - see Ch.8

5. RoB from incomplete outcome data (assess for each outcome)

- Low(1) / 2 / 3 / 4 / high(5) / unclear RoB item
- Judgement needs to factor in:
- reasons for missing data
- whether amount of missing data balanced across groups, with similar reasons
- whether censoring is less than or equal to 25% and has been taken into account
- see Ch.8

6. RoB from selective reporting (assess for each outcome)

- Low(1) / 2 / 3 / 4 / high(5) /unclear RoB item
- Judgement needs to factor in:
- existing RoB guidance on selective outcome reporting (see Ch.8)
- also, extent to which analyses (and potentially other choices) could have been manipulated to bias the findings reported, e.g., choice of method of model fitting, potential confounders considered / included
- look for evidence that there was a protocol in advance of doing any. analysis / obtaining the data (difficult unless explicitly reported); QES very different from RCTs. RCTs must have a protocol in advance of starting to recruit (for REC/IRB/other regulatory approval); QES need not (especially older studies).
- hence, separate yes/no items asking reviewers whether they think the researchers had a pre-specified protocol and analysis plan.

7. RoB from other bias

- Low(1) / 2 / 3 / 4 / high(5) /unclear RoB item
- Judgement needs to factor in:
- existing RoB guidance on other potential threats to validity (see Ch.8)
- also, assess whether suitable cluster analysis is used (e.g., cluster summary statistics, robust standard errors, the use of the design effect to adjust standard errors, multi-level models and mixture models), if assignment of units to treatment is clustered.

*Confounding Worksheet*

| **Assessment of how researchers dealt with confounding** |  |
| --- | --- |
| Method for *identifying* relevant confounders described by researchers: yes  no  If yes, describe the method used: |  |
| Relevant confounders described: yes  no  List confounders described on next page |  |
| Method used for controlling for confounding  At design stage (e.g., matching, regression discontinuity, instrument variable):  ………………………………………………..  ………………………………………………..  ………………………………………………..  At analysis stage (e.g., stratification, regression, difference-indifference):  ………………………………………………..  ………………………………………………..  ………………………………………………..  Describe confounders controlled for below |  |

*Confounders described by researchers*

Tick (yes[0]/no[1] judgement) if confounder considered by the researchers [Considered].

Score (1[good precision] to 5[poor precision]) precision with which confounder measured.

Score (1[balanced] to 5[major imbalance]) imbalance between groups.

Score (1[very careful] to 5[not at all careful]) care with which adjustment for confounder was carried out.

| **Confounder** | Considered | Precision | Imbalance | Adjustment |
| --- | --- | --- | --- | --- |
| Gender |  |  |  |  |
| Age |  |  |  |  |
| Grade level |  |  |  |  |
| Socioeconomic background |  |  |  |  |
| Performance at baseline |  |  |  |  |
| Unobservables^^[[2]](#footnote-2)^^ |  | Irrelevant |  |  |
| Other: |  |  |  |  |

*User Guide for Unobservables*

Selection bias is understood as systematic baseline differences between groups and can therefore compromise comparability between groups. Baseline differences can be observable (e.g., age and gender) and unobservable (to the researcher; e.g., ‘appearance’). There is no single non-randomised study design that always solves the selection problem. Different designs solve the selection problem under different assumptions and require different types of data. There can be particularly great variations in how different designs deal with selection on unobservables. The “right” method depends on the model generating participation, i.e. assumptions about the nature of the process by which participants are selected into an intervention.

As there is no universally correct way to construct counterfactuals, we will assess the extent to which the identifying assumptions (the assumption that makes it possible to identify the counterfactual) are explained and discussed (preferably by the authors in an effort to justify their choice of method). We will look for evidence of authors using the following examples (this is NOT an exhaustive list):

*Natural Experiments*

Discuss whether they face a truly random allocation of participants and that there is no change of behavior in anticipation of, e.g., policy rules.

*Instrument Variable (IV)*

Explain and discuss the assumption that the instrument variable does not affect outcomes other than through their effect on participation.

*Matching (including propensity scores)*

Explain and discuss the assumption that there is no selection on unobservables, only selection on observables.

*(Multivariate, Multiple) Regression*

Explain and discuss the assumption that there is no selection on unobservables, only selection on observables. Further discuss the extent to which they compare comparable people.

*Regression Discontinuity (RD)*

Explain and discuss the assumption that there is a (strict) RD treatment rule. It must not be changeable by the agent in an effort to obtain or avoid treatment. Continuity in the expected impact at the discontinuity point is required.

*Difference-in-Difference (Treatment-control-before-after)*

Explain and discuss the assumption that outcomes of participants and nonparticipants evolve over time in the same way.

## Risk of Bias Tables

**Risk of Bias in Studies Included in the Meta-Analysis**

Table A4 below displays the risk of bias assessment by item for effect sizes in studies included in the meta-analysis.

*Table A4. Risk of bias in studies included in the meta-analysis.*

*Allinder et al. (2001)*

| **Item** | **Assessment** | **Support for assessment** |
| --- | --- | --- |
| Adequate sequence generation? | Unclear | No information on sequence generation. |
| Allocation concealment? | Unclear | Unclear how the random sequence was generated. |
| Blinding? | 3 | Educators are not blind to treatment status, while students may be. |
| Incomplete outcome data addressed? | 1 | One student leaves the study. |
| Selective reporting? | 1 | Nothing suggests selective reporting. |
| Other bias? | 1 | Small sample, but no large differences over demographic variables measured pre-intervention. |
| Followed a priori protocol? | Unclear | Not reported. |
| Followed a priori analysis plan? | Unclear | Not reported. |
| Confounding? | Not relevant | RCT. |

*Bark & Brooks (2018)*

| **Item** | **Assessment** | **Support for assessment** |
| --- | --- | --- |
| Adequate sequence generation? | High | Non-random sequence generation. |
| Allocation concealment? | High | High risk sequence generation. |
| Blinding? | 4 | Parental consent given and pupils were informed about the study. No information about other participant groups, so indication that any participant group was blind to treatment status. |
| Incomplete outcome data addressed? | 1 | No attrition. |
| Selective reporting? | 1 | Nothing suggests selective reporting. |
| Other bias? | 1 | No other bias detected. |
| Followed a priori protocol? | Unclear | Not reported. |
| Followed a priori analysis plan? | Unclear | Not reported. |
| Confounding? | 3 | Pair-match students within schools based on word reading attainment, spelling, age and gender. Schools assigned one student in each pair, to treatment and comparison, based on the "ease of timetabling". Compare means in the two groups. Groups are reasonably well-balanced on gender and pre-tests (some difference on the spelling test, but < 0.25 SD). |

*Barrow et al. (2009)*

| **Item** | **Assessment** | **Support for assessment** |
| --- | --- | --- |
| Adequate sequence generation? | Unclear | No information on sequence generation. |
| Allocation concealment? | Unclear | Unclear how the random sequence was generated. |
| Blinding? | 3 | Tester reasonably unaware of treatment status. |
| Incomplete outcome data addressed? | 3 | Uneven attrition between intervention and control group. Authors control for the student characteristics where attrition causes unbalanced treatment and control groups. |
| Selective reporting? | 1 | Nothing suggests selective reporting. |
| Other bias? | 4 | There are spillovers, some control students receive the intervention. In a few cases, there were not enough classes from which to randomly pick one to go into the lab. For these cases, the study combined classes from two periods. |
| Followed a priori protocol? | Unclear | Not reported. |
| Followed a priori analysis plan? | Unclear | Not reported. |
| Confounding? | Not relevant | RCT. |

*Beattie (2000)*

| **Item** | **Assessment** | **Support for assessment** |
| --- | --- | --- |
| Adequate sequence generation? | High | QES: Individual students were assigned by computer-generated randomization to one of four intervention groups or to a control group. However, randomization occurred in a two-step process due to external constraints. Some students participate in another study and are only randomized between different interventions, not between intervention and control groups. We therefore assessed this study as a QES. |
| Allocation concealment? | High | Sequential randomization in two-steps where students also participating in an fMRI study were randomized in to two out of four groups. |
| Blinding? | 4 | Participants are likely to be aware of treatment status. Unclear who performs the tests. |
| Incomplete outcome data addressed? | 3 | "Eighty-one of the eligible students began the study, however, 17 students were excluded from full participation due to attrition" (p. 65). One student is deliberately excluded: "not included in the analyses after completing treatment, due to an initial scoring error discovered during the analyses phase of the study." (p. 65) No analysis of differential attrition. |
| Selective reporting? | 1 | Nothing suggests selective reporting. |
| Other bias? | 4 | A few reasonably large pre-test differences, potentially due the problematic randomization. |
| Followed a priori protocol? | Unclear | Not reported. |
| Followed a priori analysis plan? | Unclear | Not reported |
| Confounding? | 4 | Method for identifying relevant confounders not described, and only pre-intervention test results are described. Use an ANCOVA to control for pre-test differences, and another to control for verbal intelligence. However, do not report adjusted means or any statistic that we can use to calculate effect sizes from these specifications. Also analyses difference scores in an ANOVA, and reports an F-test for difference scores. |

*Bhat et al. (2003)*

| **Item** | **Assessment** | **Support for assessment** |
| --- | --- | --- |
| Adequate sequence generation? | High | QES. “A within-group repeated-measures design was used. At the outset all students were given the pretest. Then the sample was split into two equivalent groups, A and B, based on LACT scores. Instruction began for Group A alone. After instruction for Group A was completed, both groups were given Test 2, and instruction was initiated for Group B” (p. 77). |
| Allocation concealment? | High | Allocation does not seem to be concealed for anyone. |
| Blinding? | 4 | Participants are unlikely to be blind to treatment status. |
| Incomplete outcome data addressed? | 1 | No indication of incomplete outcome data. |
| Selective reporting? | 1 | Nothing suggests selective reporting. |
| Other bias? | 1 | No other bias detected. |
| Followed a priori protocol? | Unclear | Not reported. |
| Followed a priori analysis plan? | Unclear | Not reported |
| Confounding? | 4 | Method for identifying relevant confounders is not described. Gender, age, race, SES plus pre-tests are included. The sample was split into two equivalent groups, A and B, based on LACT scores. However, groups are clearly not equivalent on several of the confounders included, and there are pre-test differences of substantial size on one outcome measure (CTOPP). The SES-numbers reported do not add up to correct group size. |

*Bhattacharya & Ehri (2004)*

| **Item** | **Assessment** | **Support for assessment** |
| --- | --- | --- |
| Adequate sequence generation? | Unclear | Students with similar scores from the same classroom formed triplets whose members were assigned randomly to one of the three experimental groups. But no information about how the random sequence was generated. |
| Allocation concealment? | Unclear | Unclear how the random sequence was generated. |
| Blinding? | 4 | Students are randomized in triplets within classroom to 2 interventions and 1 control group, so unlikely to be blind to treatment status. All other participants are also unlikely to be blinded. |
| Incomplete outcome data addressed? | 1 | No reported attrition. |
| Selective reporting? | 1 | Nothing suggests selective reporting. |
| Other bias? | 2 | Characteristics and pre-intervention tests are balanced, or at least not significantly different from each other for any group. |
| Followed a priori protocol? | Unclear | Not reported |
| Followed a priori analysis plan? | Unclear | Not reported |
| Confounding? | Not relevant | RCT. |

*Borman et al. (2009)*

| **Item** | **Assessment** | **Support for assessment** |
| --- | --- | --- |
| Adequate sequence generation? | Unclear | Within-school random assignment of students, but no information about how the random sequence was generated. |
| Allocation concealment? | Unclear | Unclear how the random sequence was generated. |
| Blinding? | 4 | Participants are unlikely to be blind to treatment status. |
| Incomplete outcome data addressed? | 3 | Attrition for the seventh grade sample was 38% for intervention group and 30% for the control group. Outliers are removed from the analysis: 12 from intervention and 9 from control group (but regression results are shown in the appendix include outliers). |
| Selective reporting? | 3 | Do not show raw post-test means, only results of ITT and LATE regressions. |
| Other bias? | 1 | No other bias detected. |
| Followed a priori protocol? | Unclear | Not reported. |
| Followed a priori analysis plan? | Unclear | Not reported. |
| Confounding? | Not relevant | RCT. |

*Bosnjak et al. (2017)*

| **Item** | **Assessment** | **Support for assessment** |
| --- | --- | --- |
| Adequate sequence generation? | Unclear | No information about sequence generation. |
| Allocation concealment? | Unclear | Unclear how the random sequence was generated. |
| Blinding? | 4 | No indication of any participant group being blind to treatment status. |
| Incomplete outcome data addressed? | 1 | Seems likely that there is no attrition (after treatment begins). |
| Selective reporting? | 1 | Nothing suggests selective reporting. |
| Other bias? | 3 | Only 16 participants, 8 in treatment and 8 in control. Two participants (25%) in control group had been previously assessed as having high intellectual potential. This was discovered after intervention had begun, and the two students were kept in the control group. Gender balanced but large imbalance on mathematics (almost 1 SD) and spelling (appr. 0.4 SD) and small imbalance on reading. |
| Followed a priori protocol? | Unclear | Not reported. |
| Followed a priori analysis plan? | Unclear | Not reported. |
| Confounding? | Not relevant | RCT. |

*Boster et al. (2005)*

| **Item** | **Assessment** | **Support for assessment** |
| --- | --- | --- |
| Adequate sequence generation? | Unclear | Students were assigned randomly to intervention and control groups but there is no more information about how the random sequence was generated. |
| Allocation concealment? | Unclear | Unclear how the random sequence was generated. |
| Blinding? | Unclear | There is no information on how control and intervention groups are distributed across schools. |
| Incomplete outcome data addressed? | 4 | 81 intervention participants were lost due to attrition, 18% of total sample. For the CST, 33% are said to be missing. No formal analysis of differential attrition. |
| Selective reporting? | 1 | Nothing suggests selective reporting. |
| Other bias? | 2 | Pre-tests are reasonably well balanced but there is no other variable reported. |
| Followed a priori protocol? | Unclear | Not reported. |
| Followed a priori analysis plan? | Unclear | Not reported. |
| Confounding? | Not relevant | RCT. |

*Boyle (1996)*

| **Item** | **Assessment** | **Support for assessment** |
| --- | --- | --- |
| Adequate sequence generation? | Unclear | Students were pair-matched on the categorical control variables and randomly assigned to intervention and control groups, but no further information on how the random sequence was generated. |
| Allocation concealment? | Unclear | Unclear how the random sequence was generated. |
| Blinding? | 4 | Participants are unlikely to be blind to treatment status. |
| Incomplete outcome data addressed? | 1 | There is no attrition reported after randomization. |
| Selective reporting? | 1 | Nothing suggests selective reporting. |
| Other bias? | 2 | Students are matched in pairs before randomization, and students where a satisfactory match cannot be found are not included in the study. This limits sample size and generalizability, but should not bias the effect estimates as randomization is performed after pair-matching. No information on how treated and control students are divided among the two schools and if matching is done within schools. |
| Followed a priori protocol? | Unclear | Not reported. |
| Followed a priori analysis plan? | Unclear | Not reported. |
| Confounding? | Not relevant | RCT. |

*Briggs (1997)*

| **Item** | **Assessment** | **Support for assessment** |
| --- | --- | --- |
| Adequate sequence generation? | High | QES: matched pairs where the students come from four classes (with 4 teachers), 2 intervention and 2 control, and were randomly assigned to these classes (but not to treatment). All teachers volunteered to participate in their respective role. But unclear how and why they were assigned to their respective roles. |
| Allocation concealment? | High | Non-random assignment. |
| Blinding? | 4 | Teachers, parents, treated and control students clearly aware of treatment status. No indication in the study that the tester is unaware of treatment status. |
| Incomplete outcome data addressed? | 3 | For the Textbook exam outcome they have data for 30 pairs (7 missing). A total of 58 students on the two intervention teams were eligible but only 37 included either because the researcher was unable to obtain informed consent from a parent (or guardian) or was unable to locate a good match among the control teams. The number lost to matching is not reported. |
| Selective reporting? | 1 | Nothing suggests selective reporting. |
| Other bias? | 1 | No other bias detected. |
| Followed a priori protocol? | Yes | See mention on p. 87. Unclear though exactly what the protocol specifies, it, or a reference, is not included in the study. |
| Followed a priori analysis plan? | Yes | See mention on p. 88. Unclear though exactly what the plan specifies, it, or a reference, is not included in the study. |
| Confounding? | 4 | Only consider the pre-intervention outcome measure, nothing else is mentioned. Match in pairs according to reading score patterns in the vocabulary and comprehension subtests of the Gates-MacGinitie Reading Test. Exclude students in the Learning Disabled (LD) and Emotionally Disturbed (ED) programmes. English as a Second Language (ESL) students who were taking supplemental language classes were also excluded. Unclear why some teachers agree to participate as intervention teachers and some as controls, this part of the assignment procedure is not well-described. Differences in pre-test GMRT scores are small according to authors, although no formal test or info on means is presented. Use an ANCOVA in the analysis. |

*Caggiano (2007)*

| **Item** | **Assessment** | **Support for assessment** |
| --- | --- | --- |
| Adequate sequence generation? | High | QES: participants selected into treatment based on pre-test scores and recommendations by teachers/guidance counsellors/reading specialists |
| Allocation concealment? | High | Non-random assignment. |
| Blinding? | 4 | Participants are unlikely to be blind to treatment status. |
| Incomplete outcome data addressed? | 1 | No attrition reported. |
| Selective reporting? | 2 | No tests for equivalence of intervention and control groups are reported. (p. 95) |
| Other bias? | 1 | Imbalances on pre-test are insignificant and relatively small (Table 4, p. 96). |
| Followed a priori protocol? | Unclear | There is a mention of a protocol on p. 170, but it regards the use of human subjects. It is unclear what was specified in the protocol regarding e.g., the analysis. |
| Followed a priori analysis plan? | Unclear | There is a mention of a protocol on p. 170, but it regards the use of human subjects. It is unclear what was specified in the protocol regarding e.g., the analysis. |
| Confounding? | 4 | Method for identifying relevant confounders unclear. Grade level, gender and ethnicity and pre-test score described. Students were matched, however there is no mention on the method used for matching. Furthermore, there are no tables or tests beyond pre-test scores to support that the matching was successful, possibly because the other variables are exactly matched. Students are selected into treatment by pre-test scores and teacher/guidance counsellor/reading specialist recommendations. Unclear why control group students are not recommended, and what recommendations are based on. |

*Calhoon & Fuchs (2003)*

| **Item** | **Assessment** | **Support for assessment** |
| --- | --- | --- |
| Adequate sequence generation? | Unclear | Random assignment, but no information about how the random sequence was generated. |
| Allocation concealment? | Unclear | Unclear how the random sequence was generated. |
| Blinding? | ¾ | 10 classrooms taught by 3 teachers are randomized to intervention and control, so teachers have both intervention and control classes. No indications that any other participants are blind to treatment status either. The state test is however not administered by the study, and testers are likely blind to treatment status. Rating is 3 for the effect size based on the state test, and 4 for effect sizes based on the Math Operations Test and the Math Concepts and Applications Test. |
| Incomplete outcome data addressed? | 3/2 | The state test is optional so only 56 students in the study took both the October and the March administrations. Thus, missing outcome data for 39%. In addition, there might be a selection of students taking the test. The levels of attrition and the risk of selection are lower regarding Math Operations Test and Math Concepts and Applications Test. Rating is 3 for effect sizes based on the state test, and 2 for effect sizes based on the other two. |
| Selective reporting? | 1 | Nothing suggests selective reporting. |
| Other bias? | 3 | “It is important to note that the study began with 120 students, but 28 of these students had dropped out of school by March; therefore, only 92 students began and completed the project. Using chi-square analyses on categorical data, no reliable differences were found between treatment groups for sex, special education label, and grade level. However, a significant difference was found between treatment groups for race. ANOVAs run on continuous data (grade; number of years in special education; and grade-level performance in math, as judged by teachers) showed no significant differences between treatment groups.” (p. 237). Pre-treatment differences on the statewide test are however relatively large, and the number of units randomized over are very small. The pre-test data is only shown for the 56 students who took the statewide test both times |
| Followed a priori protocol? | Unclear | Not reported. |
| Followed a priori analysis plan? | Unclear | Not reported. |
| Confounding? | Not relevant | RCT. |

*Campuzano et al. (2009)*

| **Item** | **Assessment** | **Support for assessment** |
| --- | --- | --- |
| Adequate sequence generation? | Unclear | Randomly assigned 428 volunteering teachers to either use or not use the products in their classrooms. No information about the random sequence generation. |
| Allocation concealment? | Unclear | Unclear how the random sequence was generated. |
| Blinding? | 2 | Randomization is done over teachers, who are aware of treatment status. Students and tester are likely to be unaware. |
| Incomplete outcome data addressed? | 3/2 | There are some substantial differences between the interventions (two CAI programmes), but not in a systematic way, i.e. they are both more and less attrition in intervention groups compared to control groups. More attrition though in the Cognitive Tutor Algebra 1 treatment (27.6%, rated 3), than in the Larson Algebra I (16.6%, rated 2). |
| Selective reporting? | 1 | Nothing suggests selective reporting. |
| Other bias? | 2 | From p. 17: "However, if teacher decisions to exit grade levels, their school, or teaching per se, are related to the use or non-use of products in the classrooms, the integrity of the experimental design is reduced. Tables presented below [...] indicate that teachers in the treatment and control groups who continued in the second year generally had similar characteristics, but whether unobserved characteristics are similar cannot be known.” |
| Followed a priori protocol? | Unclear | Not reported. |
| Followed a priori analysis plan? | Unclear | Not reported. |
| Confounding? | Not relevant | RCT. |

*Cantrell et al. (2010)*

| **Item** | **Assessment** | **Support for assessment** |
| --- | --- | --- |
| Adequate sequence generation? | High | “A within-school iterative random sampling process was used to place eligible students in treatment and control. To maximize power and to ensure the intervention and control groups were as similar as possible for all the demographic variables collected, we stratified the sample on four demographic variables: special education status, free or reduced-price lunch status, ethnicity, and gender. Average normal curve equivalent (NCE) scores for the two groups were then compared; if average NCEs were more than two NCEs different, students were randomly selected from affected strata until the average NCE scores of treatment and control were highly comparable. This process was repeated until treatment and control were equivalent in terms of achievement scores and in terms of each of the four strata variables” (p. 261-262). |
| Allocation concealment? | High | Sequence generation is rated high. |
| Blinding? | 4 | Participants are unlikely to be blind to treatment status. |
| Incomplete outcome data addressed? | 3 | Attrition treated 24% and control 31%. |
| Selective reporting? | 2 | Pre-test means and standard deviations are not reported. |
| Other bias? | 4 | Unclear what parts of the assignment that are randomised. The 'randomisation' procedure has assured the groups are equivalent and yet the adjusted post means shows a difference (although small) whereas the unadjusted post means do not. |
| Followed a priori protocol? | Unclear | Not reported. |
| Followed a priori analysis plan? | Unclear | Not reported. |
| Confounding? | Not relevant | RCT. |

*Cantrell et al. (2016)*

| **Item** | **Assessment** | **Support for assessment** |
| --- | --- | --- |
| Adequate sequence generation? | Low | “The students were systematically assigned to the intervention or control group by sorting the students by demographic group and GRADE score within each subgroup. A random number generator was used to assign the first student into either the intervention or control group. Each subsequent student was alternately assigned to intervention or control.” (p. 13) |
| Allocation concealment? | Unclear | Unclear if sequence is properly concealed, not stated explicitly. |
| Blinding? | Unclear | GRADE was administered in classrooms by teachers. There is a chance that the teachers know how the students are allocated, but it is not clear whether or not it is the same teachers that provide the intervention. Intervention and control students in the same schools, but not in the same classes. Unclear how much they know about treatment status. No statement in article about parental consent. |
| Incomplete outcome data addressed? | 2 | 104 of 744 in the intervention group leave the study, and 100 out of 734 leave the control group. No formal analysis of differential attrition, e.g., if it is the same type of students that leave the intervention and control groups. |
| Selective reporting? | 1 | Nothing suggests selective reporting. |
| Other bias? | 1 | No other bias detected. |
| Followed a priori protocol? | Unclear | Not reported. |
| Followed a priori analysis plan? | Unclear | Not reported. |
| Confounding? | Not relevant | RCT. |

*Caudell (2016)*

| **Item** | **Assessment** | **Support for assessment** |
| --- | --- | --- |
| Adequate sequence generation? | Low | Randomise between eligible students using randomizer.org. |
| Allocation concealment? | Low | Non-sequential randomisation. |
| Blinding? | 3 | Students and parents are aware of treatment status. Use a state test which is independent of the study, so assessor is likely blinded. No information about other participant groups. |
| Incomplete outcome data addressed? | 1 | 34 students in treatment and 34 students in the control group. 3 students, one from the treatment grou and two from the control group, leave the study. The treatment group student is removed from treatment due to low attendance. |
| Selective reporting? | 1 | Nothing suggests selective reporting. |
| Other bias? | 3 | A few students (9%) are tested at different times than the majority. No information on how they are distributed across treatment and control groups. Large imbalance on ethnicity. Also differences over content areas. Researcher is the guidance counselor in the participating school. Pre-tests available but not used as controls. However, well-balanced on all pre-tests. |
| Followed a priori protocol? | Unclear | Not reported. |
| Followed a priori analysis plan? | Unclear | Not reported. |
| Confounding? | Not relevant | RCT. |

*Cleary et al. (2017)*

| **Item** | **Assessment** | **Support for assessment** |
| --- | --- | --- |
| Adequate sequence generation? | Unclear | Utilized a stratified randomization procedure to ensure that the two groups had an equal number of students from each teacher. No information on the random sequence generation. |
| Allocation concealment? | Unclear | Unclear how the random sequence was generated. |
| Blinding? | 4 | All students are in the same school and have one of two teachers, so are likely not blind to treatment. No information about consent, but seems unlikely that parents do not know. Unclear who corrects the tests (might be teachers). |
| Incomplete outcome data addressed? | 3 | "Two students from the comparison group were removed from the dataset because of their extremely sporadic school attendance and corresponding missing data across several of the measures at posttest and follow-up" (p. 26). "In terms of the quarterly exams, there were a few students with missing data. Given the nature of our within group analyses, we elected to remove them prior to conducting this analysis. Based on school records, these students did not take some of the exams either because they moved to a different school in 8th grade or because they enrolled in a different type or level of mathematics class in 8th grade." (p. 27) |
| Selective reporting? | 1 | Nothing suggests selective reporting. |
| Other bias? | 2 | "No initial group differences were observed across any of the measures except for the microanalytic attributions measure." (p. 27), Reasonably well balanced on student characteristics, and on all pre-tests except one. |
| Followed a priori protocol? | Unclear | Not reported. |
| Followed a priori analysis plan? | Unclear | There is a section on Data Analysis Plan, but unclear if this was made before the intervention. |
| Confounding? | Not relevant | RCT. |

*Cook et al. (2014)*

| **Item** | **Assessment** | **Support for assessment** |
| --- | --- | --- |
| Adequate sequence generation? | Unclear | “Our research team carried out the random assignment for the current project ourselves. [...] our random assignment algorithm intentionally over-assigned eligible youth to the group that received both our academic and non-academic intervention, with a lower assignment probability for the non-academic-only group” (p. 15-16). |
| Allocation concealment? | Unclear | Unclear how the random sequence was generated. |
| Blinding? | 3 | Students are in the same school. For those assigned to programming, consents for programme participation were sought from youth and their parents. So control group are not actively informed. Tests are administered by the school district so tester should be blind to treatment status. |
| Incomplete outcome data addressed? | 3 | “Table 2 shows that youth assigned to any treatment (third column) turn out to be about eight percentage points more likely than controls to have valid scores for the spring 2013 (post-random assignment) EXPLORE and PLAN achievement tests. The results that we present below suggest the differential rate of missing-ness for end-of-year test scores does not seem to be due to treatment effects that reduce school dropout, but could be due to treatment effects that reduce student school absences." (p. 18 and 44) |
| Selective reporting? | 1 | Nothing suggests selective reporting. |
| Other bias? | 1 | No significant imbalances between the joint intervention group and the control group. There is cross-over between interventions but none between the control group and the intervention groups. Following the authors, we combined interventions into one effect size, so this should not be problematic. |
| Followed a priori protocol? | Unclear | Not reported. |
| Followed a priori analysis plan? | Unclear | They mention an analysis plan, but unclear if this was developed in advance (p. 17). |
| Confounding? | Not relevant | RCT. |

*Corrin et al. (2008)*

| **Item** | **Assessment** | **Support for assessment** |
| --- | --- | --- |
| Adequate sequence generation? | Low | Within each district, high schools were randomly assigned to one of the two interventions. Within each high school, eligible students were randomly assigned to treatment/control. Computerized random assignment of students was conducted solely by MDRC staff. |
| Allocation concealment? | Low | Non-sequential allocation. |
| Blinding? | 4 | Participants are unlikely to be blind to treatment status. |
| Incomplete outcome data addressed? | 3 | Among the students randomly assigned to the ERO group, 91 percent enrolled in the ERO classes, and 87 percent were still attending the classes at the end of the school year. Overall, Follow-up test scores are available for 2,171 (81 percent). For the Reading Apprenticeship intervention the numbers for treated/control are 82.4/79.4 and for the Xtreme Reading intervention 83.0/78.7. Their analysis of differences between responders/non responders show that response rates are lower for students with characteristics associated with doing poorly in school. |
| Selective reporting? | 1 | Nothing suggests selective reporting. |
| Other bias? | 1 | No other bias detected. |
| Followed a priori protocol? | Unclear | Not reported. |
| Followed a priori analysis plan? | Unclear | Not reported. |
| Confounding? | Not relevant | RCT. |

*Corsello & Sharma (2015)*

| **Item** | **Assessment** | **Support for assessment** |
| --- | --- | --- |
| Adequate sequence generation? | Unclear | Students were randomised by Abt Associates, the i3 oversight evaluators, and sorted to ensure balance of gender and ethnicity. However, no information about the random sequence generation. |
| Allocation concealment? | Unclear | Unclear how the random sequence was generated. |
| Blinding? | 3 | Tester is likely blind to treatment status, tests not administered by the study. No other participant is likely to be blind to treatment status. |
| Incomplete outcome data addressed? | 2 | Missing data in reading/math: Treated 6.1%/9.4% and control 15.5%/16.7%. |
| Selective reporting? | 1 | Nothing suggests selective reporting. |
| Other bias? | 2 | Principals assigned teachers to BARR or non-BARR conditions. They were told not to select the best or most willing teachers to be part of the BARR intervention but to mix teachers as evenly as possible according to years of experience, gender, level of education, and ethnicity. |
| Followed a priori protocol? | Unclear | Not reported. |
| Followed a priori analysis plan? | Unclear | Not reported. |
| Confounding? | Not relevant | RCT. |

*Denton et al. (2008)*

| **Item** | **Assessment** | **Support for assessment** |
| --- | --- | --- |
| Adequate sequence generation? | Unclear | Students were randomly assigned within classrooms, but no information about the random sequence generation. |
| Allocation concealment? | Unclear | Unclear how the random sequence was generated. |
| Blinding? | 4 | Only one school so teachers are likely to know treatment status. No information about whether students are taken from the same classes. Graduate students administer post-test, not stated that they are unaware of treatment status. |
| Incomplete outcome data addressed? | 1 | Two students out of 40 leave the study, both likely from the control group although it is not explicitly stated. The reasons given seem unrelated to treatment/lack of treatment: one student was sent to an alternative school for misbehavior and one student moved out of the school. |
| Selective reporting? | 2 | Unclear what the following means: “It is not possible to report group standard score means for the Spanish test, because several students performed so poorly that it was impossible to assign them standard scores” (p.4). This seems to concern a test that is only reported student-by-student. |
| Other bias? | 3 | Some imbalances on pre-intervention test scores and students characteristics, though not systematically in favor of any group. |
| Followed a priori protocol? | Unclear | Not reported. |
| Followed a priori analysis plan? | Unclear | Not reported. |
| Confounding? | Not relevant | RCT. |

*Early (1998)*

| **Item** | **Assessment** | **Support for assessment** |
| --- | --- | --- |
| Adequate sequence generation? | Unclear | Among the pool of students eligible for tutoring (based on low 8 grade scores) the intervention group was randomly selected and paired with students eligible for being tutors (high scores) who were also randomly selected. No information about the random sequence generation. |
| Allocation concealment? | Unclear | Unclear how the random sequence was generated. |
| Blinding? | 3 | Permission from all participants collected so no student is unaware of treatment status. At least some teachers supervise, unclear how many, so they are aware of treatment status. Use the 10th grade state test, so tester is likely not aware of treatment status. |
| Incomplete outcome data addressed? | 2 | No mention of e.g., tutors and tutees who turn down the offer or quit during the programme, which there ought to have been as participation was entirely voluntary. Data presented for 289 out of 340 students, i.e., about 15% attrition. |
| Selective reporting? | 1 | Nothing suggests selective reporting. |
| Other bias? | 1 | No other bias detected. |
| Followed a priori protocol? | Unclear | Not reported. |
| Followed a priori analysis plan? | Unclear | Not reported. |
| Confounding? | 3 | Only age is not explicitly considered, but all are from grade 10. All from grade 10. Some imbalance on gender for tutors. Also show pre-intervention measures of self-efficacy referring to students’ self-evaluation of competences with academic and social behaviour, which show no substantial imbalance. Use a stepwise multiple regression in the analysis (with demographics, pre-test and intervention indicator as variables), where only the significant variables remain in the final model. |

*Fogarty et al. (2014)*

| **Item** | **Assessment** | **Support for assessment** |
| --- | --- | --- |
| Adequate sequence generation? | Unclear | Students from participating classes were randomly assigned within teachers to intervention classes or typical practice conditions classes. No information about random sequence generation. |
| Allocation concealment? | Unclear | Unclear how the random sequence was generated. |
| Blinding? | 4 | A within teacher design, where classes are randomized to treatment or control, so teachers are not blind to treatment status. No indication that students or testers were blind either. |
| Incomplete outcome data addressed? | 2 | "Of the 859 students who began the study, 736 (85.6 %) participated in both pretest and posttest assessments. To determine whether there was differential attrition between students who exited the study and those who remained to complete the posttest assessment, we compared the two groups (i.e., the 123 attritors vs. the 736 nonattritors) on all demographic variables, including gender, ethnicity, special education status, and group (intervention and comparison conditions) on all pretest measures. No significant differences were found between attritors and nonattritors on any variable." (p. 434). These results are not shown, only described in text. |
| Selective reporting? | 3 | Do not show results from attrition analysis, only reports no significant differences. |
| Other bias? | 1 | No other bias detected. |
| Followed a priori protocol? | Unclear | Not reported |
| Followed a priori analysis plan? | Unclear | Not reported |
| Confounding? | Not relevant | RCT. |

*Fogarty et al. (2017)*

| **Item** | **Assessment** | **Support for assessment** |
| --- | --- | --- |
| Adequate sequence generation? | Unclear | No information about sequence generation. |
| Allocation concealment? | Unclear | Unclear sequence generation. |
| Blinding? | 3/4 | Nothing to indicate blinding of treatment status except that there is a state test, which is administered by schools. Rating 3 for this test and 4 for the others. |
| Incomplete outcome data addressed? | 1 | “Of the 237 students who entered the study, 223 (94,1%) participated in both pretst and posttest assessments; 14 (5,95) did not participate in any posttest assessments because they moved out of the district during the study" (p. 322)”. Pearson Chi-square indicates, that attrition is not associated with gender, ethnicity, grade, economic status or Special education/ELL status. |
| Selective reporting? | 1 | Nothing suggests selective reporting. |
| Other bias? | 3 | Study worked with school-identified participants, who were already placed in remedial classrooms. Large variation in class size, ranges from 4 to 24 students. Eight pre-tests, all except one reasonably balanced. Marked differences in control intervention (typical reading) practices. |
| Followed a priori protocol? | Unclear | Not reported. |
| Followed a priori analysis plan? | Unclear | Not reported. |
| Confounding? | Not relevant | RCT. |

*Fryer (2011)*

| **Item** | **Assessment** | **Support for assessment** |
| --- | --- | --- |
| Adequate sequence generation? | Low | Schools are randomised. Use a re-randomization procedure that minimizes the predictive ability of a regression of pre-determined covariates on an intervention indicator. |
| Allocation concealment? | Low | Non-sequential allocation. |
| Blinding? | 3 | Randomised at school level, but parental consent needed, so no family was blind to treatment status. It seems likely that control students and testers were blind to treatment. |
| Incomplete outcome data addressed? | 1 | Across all cities there is little evidence that there was differential mobility across intervention and control schools in the year of treatment. |
| Selective reporting? | 1 | Nothing suggests selective reporting. |
| Other bias? | 3 | All interventions are balanced. An extensive set of control variables are included. In New York City, seven schools [out of 143] were switched from control to intervention (three in fourth grade and four in seventh grade) (p. 19 in working paper). |
| Followed a priori protocol? | Unclear | Not reported. |
| Followed a priori analysis plan? | Unclear | Not reported. |
| Confounding? | Not relevant | RCT. |

*Good et al. (2003)*

| **Item** | **Assessment** | **Support for assessment** |
| --- | --- | --- |
| Adequate sequence generation? | Unclear | No information about the random sequence generation. |
| Allocation concealment? | Unclear | Unclear how the random sequence was generated. |
| Blinding? | 3 | Mentors are not blind to treatments. Students are unlikely to know about the differences among the treatments and the (placebo) control condition. |
| Incomplete outcome data addressed? | 4 | There is very little attrition. Authors however remove outliers which affects some of the results. |
| Selective reporting? | 4 | Not all comparisons are shown. The authors refer to planned comparisons, but there is no mention of where this plan or protocol can be accessed. |
| Other bias? | 4 | No statistics on sample balance is shown. |
| Followed a priori protocol? | Unclear | Not reported. |
| Followed a priori analysis plan? | Unclear | Not reported. |
| Confounding? | Not relevant | RCT. |

*Haslam et al. (2006)*

| **Item** | **Assessment** | **Support for assessment** |
| --- | --- | --- |
| Adequate sequence generation? | High | QES: so sequence is not random. |
| Allocation concealment? | High | Non-random assignment. |
| Blinding? | 4 | Participants are unlikely to be blind to treatment status. |
| Incomplete outcome data addressed? | 3 | For the intervention group 25% had missing data on primarily the test (pre and/or post). 409 treated, 307 analysed. |
| Selective reporting? | 1 | Nothing suggests selective reporting. |
| Other bias? | 1 | No other bias detected. |
| Followed a priori protocol? | Unclear | Not reported. |
| Followed a priori analysis plan? | Unclear | Not reported. |
| Confounding? | 3 | No method for identifying relevant confounders is described by researchers. Use an exact matching procedure for grade, gender, race, economically disadvantaged, and special education. It was not always possible to find an exact match for Low English Proficiency or the pre-test score, the latter shows a difference of some substance. |

*Hutchinson (1993)*

| **Item** | **Assessment** | **Support for assessment** |
| --- | --- | --- |
| Adequate sequence generation? | Unclear | Twenty students within two schools were randomised. No information about the random sequence generation. |
| Allocation concealment? | Unclear | Unclear how the random sequence was generated. |
| Blinding? | 4 | Participants are unlikely to be blind to treatment status. |
| Incomplete outcome data addressed? | 1 | No mention or indication of incomplete outcome data. |
| Selective reporting? | 1 | Nothing suggests selective reporting. |
| Other bias? | 4 | The strategy instructor was the researcher for all intervention students (p. 40), so researcher is part of the intervention. Very small group, but small imbalances on both pre-tests (p. 47). The tests used are subsets of larger standardised tests and it is unclear how these questions have been selected. We interpret it as all word problems have been used, and not a subset suited for the intervention, but this is not stated explicitly. |
| Followed a priori protocol? | Unclear | Not reported. |
| Followed a priori analysis plan? | Unclear | Not reported. |
| Confounding? | Not relevant | RCT. |

*Jeffes (2013)*

| **Item** | **Assessment** | **Support for assessment** |
| --- | --- | --- |
| Adequate sequence generation? | Unclear | The 30 students with the lowest word reading ability were allocated to either a WCG or Test group using a matched randomisation method (match in pairs on age, gender and word-reading ability), but no more information on how the random sequence was generated. |
| Allocation concealment? | Unclear | Unclear how the random sequence was generated. |
| Blinding? | 4 | All students are in the same two schools, and control group is waitlisted. Thus, students and teachers are likely to be aware of treatment status. Parents’ consent is asked for. “All individual reading test results from the base-line and test stages were marked and scored by an experimenter” (p. 64), but no mention of whether the experimenter is blind to treatment status. |
| Incomplete outcome data addressed? | 2 | There is no attrition mentioned. Sample size is not included in the tables showing results. Two students were removed from analysis due to having missed almost half the sessions (one each from intervention and control groups). Unclear if removed students belong to the same pair or not. |
| Selective reporting? | 2 | “As measures of fluency and accuracy had already been taken, accuracy and reading rate scores generated by the passage reading comprehension test were not analysed.” (p. 56). There is however a passage comprehension test being analysed (e.g., p. 73). Unclear therefore if the sentence refers to some other test that was performed but not reported. |
| Other bias? | 4 | Intervention and control groups are well-balanced on pre-tests, and pre-determined characteristics. “There was a concern surrounding the reliability of these results as so many students tested fell below the minimum standard score of 70 at baseline and test on the Word Recognition Accuracy test used. Any scores below a standard score of 70 on this test are simply recorded as <70. This lack of sensitivity in the test made it hard to measure progress using standard scores as any students scoring <70 at baseline and test had their potential progress masked by their unchanging standard score. For this reason the data was re-analysed using the student's raw scores from the test to investigate group differences further” (p. 68). This concern is not mention for the other tests. It is unclear how means are calculated if some scores are just recorded as <70. Yet this is done for the concerned test, and perhaps also for the others. The degrees of freedom on the F-tests do not match the number of students. |
| Followed a priori protocol? | Unclear | Not reported. |
| Followed a priori analysis plan? | Unclear | Not reported. |
| Confounding? | Not relevant | RCT. |

*Justus (2010)*

| **Item** | **Assessment** | **Support for assessment** |
| --- | --- | --- |
| Adequate sequence generation? | Low | Students are matched in pairs by gender, ethnicity, and PLAN score. A coin toss determined assignment to intervention and control group. |
| Allocation concealment? | Low | Non-sequential allocation. |
| Blinding? | 3 | Tester is likely blind to treatment status, as the post-test is performed by a separate, private firm. Teachers are notified, parental permission is sought, and control group students are waitlisted, so no other group is likely to be blind to treatment status. |
| Incomplete outcome data addressed? | Unclear | “The intervention course, ACT Mathematics Intervention, was implemented first semester of 2009-2010 at only one of the two schools. The other school had to disband the class due to overcrowding and lack of staffing availability.” (p. 64) Unclear if this is after randomization and if they started the course at all. Of the assigned 20 treatment students in the implementing school, 2 drop out. Their matched pairs are also dropped from the analysis. |
| Selective reporting? | 1 | Nothing suggests selective reporting. |
| Other bias? | 1 | Small sample, but matched exactly on gender and ethnicity, and almost exactly on pre-test scores. |
| Followed a priori protocol? | Unclear | Not reported. |
| Followed a priori analysis plan? | Unclear | Not reported. |
| Confounding? | Not relevant | RCT. |

*Kemple et al. (2008)*

| **Item** | **Assessment** | **Support for assessment** |
| --- | --- | --- |
| Adequate sequence generation? | Low | Within each district, high schools were randomly assigned to one of the two interventions (there were an even number of schools in each district, 7 with 4 schools and 3 with 2 schools). Within each high school, eligible students were randomly assigned to intervention/control. Computerized random assignment of students was conducted by MDRC staff |
| Allocation concealment? | Low | Non-sequential allocation. |
| Blinding? | 4 | Participants are unlikely to be blind to treatment status. |
| Incomplete outcome data addressed? | 3 | Follow-up test scores and surveys are available for 2,413 (83 percent) of the students. For the Reading Apprenticeship intervention the numbers for treated/control are 84.6/79.3 and for the Xtreme Reading intervention 83.6/82.7. Their analysis of differences between responders/non responders show that response rates are lower for students with characteristics associated with doing poorly in school but overall, there are no systematic differences between the treated and control group respondents. |
| Selective reporting? | 1 | Nothing suggests selective reporting. |
| Other bias? | 1 | No other bias detected. |
| Followed a priori protocol? | Unclear | Probably not. They mention protocols for the primary data collection instrument for the site visits was a set of protocols for classroom observations and interviews with the ERO teachers. But no explicit mention of study protocol. |
| Followed a priori analysis plan? | Unclear | Not reported. |
| Confounding? | Not relevant | RCT. |

*Kempley (2005)*

| **Item** | **Assessment** | **Support for assessment** |
| --- | --- | --- |
| Adequate sequence generation? | Unclear | The placement of the students in each group was based on random assignment, no mention of how the random sequence is generated. |
| Allocation concealment? | Unclear | Unclear how the random sequence was generated. |
| Blinding? | 4 | Participants are unlikely to be blind to treatment status. |
| Incomplete outcome data addressed? | 3 | 27 students in each group at baseline, only 23 in each group at analysis. Researchers do not elaborate further on what effect this might have. Seven students are said to exit during the first quarter, unclear whether they are part of the analysis or not. |
| Selective reporting? | 2 | Unclear what the adjusted test means are adjusted for/with. |
| Other bias? | 3 | ”Seven students entered during the 1st quarter. Fifteen students entered double math during the second quarter” (p. 41). Not clear if these students were randomly assigned. There is an imbalance on the pre-test survey of math attitudes, pre-intervention grades, and the pre-test. All in favour of the control group. |
| Followed a priori protocol? | Unclear | Not reported. |
| Followed a priori analysis plan? | Unclear | Not reported. |
| Confounding? | Not relevant | RCT. |

*Kim (2002)*

| **Item** | **Assessment** | **Support for assessment** |
| --- | --- | --- |
| Adequate sequence generation? | Unclear | The four class sections of the participating teacher were randomly assigned to either the CACSR group or the comparison group (two sections in each group), but no mention of how the random sequence is generated. |
| Allocation concealment? | Unclear | Unclear how the random sequence was generated. |
| Blinding? | 4 | The same teacher teaches all students (intervention and control). Students are asked to participate so no one is blind to treatment status. Researcher and research assistant perform the tests, unclear whether they know of treatment status (as it is unclear how randomisation is done). |
| Incomplete outcome data addressed? | 3 | 28 students randomized, 5 students (2 in the CACSR; 3 in the comparison group) discontinued their participation in the study for various reasons. |
| Selective reporting? | 1 | Nothing suggests selective reporting. |
| Other bias? | 4 | Some of the differences on pre-tests and characteristics are relatively large. No pre-test difference is significant, but the sample size is very small. Also risk that the teachers favors one of the groups, there is only one teacher. |
| Followed a priori protocol? | Unclear | Not reported. |
| Followed a priori analysis plan? | Unclear | Not reported. |
| Confounding? | Not relevant | RCT. |

*Kim et al. (2006)*

| **Item** | **Assessment** | **Support for assessment** |
| --- | --- | --- |
| Adequate sequence generation? | Unclear | Not reported, other than that the two classroom sections of each teacher were randomised. |
| Allocation concealment? | Unclear | Unclear how the random sequence was generated. |
| Blinding? | 4 | Participants are unlikely to be blind to treatment status. |
| Incomplete outcome data addressed? | 1 | Not reported explicitly but probably very low level of missing data (34 participate and F-value has degrees of freedom of 31). |
| Selective reporting? | 1 | Nothing suggests selective reporting. |
| Other bias? | 4 | The two classroom sections of each teacher were randomly assigned to either the intervention group or the comparison group. A total of 16 students participated in the intervention group, and 18 participated in the comparison group. Although CACSR implementation in the intervention group was classwide, only the data from students with disabilities were analyzed in this study. Some relatively large pre-intervention imbalances. |
| Followed a priori protocol? | Unclear | Not reported. |
| Followed a priori analysis plan? | Unclear | Not reported. |
| Confounding? | Not relevant | RCT. |

*Kim et al. (2011)*

| **Item** | **Assessment** | **Support for assessment** |
| --- | --- | --- |
| Adequate sequence generation? | High | Randomised on teacher level. SAUSD (the school district) software was used to randomly assign eligible students into English classes where lessons are designed for native English speakers. ”In the second step, we selected one classroom to include in the study. Most English teachers, however, are responsible for teaching multiple sections including classes for mainstreamed ELLs and classes for students who score below grade level and have very limited English proficiency (e.g., CELDT scores of 1 or 2). Therefore, when a teacher had multiple sections of regular English language arts, we selected the one classroom that had the highest percentage of students at or above intermediate on the CELDT, because the Pathway materials were designed specifically for these students” (p. 239). We treated this study as a QES, due to the non-random selection of classes. |
| Allocation concealment? | High | Non-random assignment. |
| Blinding? | 3 | Randomisation clustered on teachers, unclear what students know about treatment status. Unclear whether control teachers are unaware. Tester is likely unaware of treatment status. |
| Incomplete outcome data addressed? | 3 | “To test for differential attrition, we fit a multilevel model in which the key outcome was the 2007 CST pretest score and the predictor variables were treatment status, attrition status, and the interaction between treatment status and the attrition status. […] These results revealed no difference in the baseline CST 2007 scores of treatment and control students who remained in the study and those lost to attrition. In sum, there appears to be no differential attrition and the characteristics of the final sample and baseline sample appear statistically equivalent” (p. 240). However, both teachers that leave the study are from the treatment group, which indicate some differences. Twelfth grade does not seem to be included in this attrition analysis. |
| Selective reporting? | 2 | Not entirely clear how many baseline tests between intervention and control groups that are performed versus reported. |
| Other bias? | 1 | No other bias detected. |
| Followed a priori protocol? | Unclear | Not reported. |
| Followed a priori analysis plan? | Unclear | Not reported. |
| Confounding? | 2 | Randomised study but one part of the assignment is non-random. There are only small baseline differences between intervention and control teachers, and small pretest score differences between students. Use a hierarchical model to control for classroom pre-test score. |

*Kotsopoulos (2009)*

| **Item** | **Assessment** | **Support for assessment** |
| --- | --- | --- |
| Adequate sequence generation? | High | Students were “placed” in groups, no mention of randomisation in text except the abstract that mentions “a randomized experimental design”. On p. 26, a non-random placement of 3 control group students is described, in a way that indicates that the rest might have been randomly assigned. We treated the study as a QES. |
| Allocation concealment? | High | Non-random assignment. |
| Blinding? | 4 | All tests were administered and scored by the author with some tests double scored by other graduate students. All students, parents and teachers aware of treatment status. |
| Incomplete outcome data addressed? | 2 | Attrition were 4 of 28 (14%) in the intervention group and 2 of 9 (22%) in the control group. Missing data: three teachers did not fill out questionnaires for some of the participants. |
| Selective reporting? | 2 | Nelson-Denny test (standardised) is mentioned on p. 31, but unclear where the results are presented. There is a reading comprehension measure mentioned on p. 65 but not clear if this is the same, and the control group’s results are not presented. |
| Other bias? | 4 | Both treated and controls are from several schools and classes. Not completely clear what time 1 and time 2 are for the nine students who started treatment later. A total of 24 students were assigned to the PASS programme. Out of the 24 students, 9 were placed on a waiting list for the PASS programme in the first term. These 9 students were to commence the PASS programme in the winter term as their GLE (learning strategies) course was scheduled for that time (the PASS programme and GLE course run simultaneously, where students are taken out of their GLE class for one period a week to participate in the PASS programme). |
| Followed a priori protocol? | Unclear | Not reported. |
| Followed a priori analysis plan? | Unclear | Not reported. |
| Confounding? | 4 | Grade, age, gender and pre-test are considered. All are grade 9 students. Table 1 page 27 shows gender division by group. No imbalance between PASS/GLE and the no-treatment groups, but the GLE group has a 10 percent point higher share of boys than the other groups. State on page 47 that no significant differences were found with regard to age in the study across groups, but age is not shown by group. On page 52 test results by group at time 1 and time 2 are shown; the no treatment control group scores are higher in 4 out of 6 tests (there are 4 written expression subtests and one total written expression and one arithmetic subtest). The GLE control group scores higher in one written expression subtest and lower in the remaining 5 tests. The combined control group scores higher in 4 tests. Only controls for pre-test in the analysis, and only uses the combined control group. |

*Lemberger et al. (2015)*

| **Item** | **Assessment** | **Support for assessment** |
| --- | --- | --- |
| Adequate sequence generation? | Unclear | Classrooms were randomly assigned to receive either the intervention or the control condition. Control classrooms were waitlisted. No further information about the random sequence generation. |
| Allocation concealment? | Unclear | Unclear how the random sequence was generated. |
| Blinding? | 3 | Waitlist controls, so teachers and students should be aware of treatment status. The tests seem to be performed by a third party who is likely blind to treatment status. |
| Incomplete outcome data addressed? | 4 | 346 students participated at start. 205 students (59%) returned the appropriate institutional review board documentation, but 12 students were not included in the analysis (seven because of incomplete data and five who identified as receiving special education services), resulting in 193 study participants (55.8% of the initial number). Separate information on the six intervention and five control class rooms is not reported. |
| Selective reporting? | 1 | Nothing suggests selective reporting. |
| Other bias? | 3 | Some imbalances on pre-intervention measures. Very large imbalance for both math and reading, 0.8-0.9 SDs. Use a multilevel model with controls for pre-test, gender, and ethnicity in the analysis. |
| Followed a priori protocol? | Unclear | Not reported. |
| Followed a priori analysis plan? | Unclear | Not reported. |
| Confounding? | Not relevant | RCT. |

*Levitt el al. (2016)*

| **Item** | **Assessment** | **Support for assessment** |
| --- | --- | --- |
| Adequate sequence generation? | Unclear | Individual level randomization blocked on school (Bloom or Trail), gender, race/ethnicity and baseline (eighth grade) test score. Exception to individual randomization is that siblings are randomized to same intervention. No info about sequence generation though. |
| Allocation concealment? | Unclear | Unclear how the random sequence was generated. |
| Blinding? | 3 | Students randomized within schools to intervention and control, and parents are sometimes also treated and likely informed. Not clear if teachers know of treatment status, but they likely do. Tester is likely to be blind to treatment status. |
| Incomplete outcome data addressed? | 2 | Some attrition but no evidence of any substantial differential attrition. |
| Selective reporting? | 1 | Nothing suggests selective reporting. |
| Other bias? | 3 | Most pre-intervention characteristics are well-balanced: “The one exception is highly significant differences across groups for the number of honors class assignments (honors status was not available at the time of randomization). The percentage of honors students in the treatment groups is about twice the percentage in the control group […]. As shown below, the results are robust to including honors classes along with our other covariates.” (p. 18). |
| Followed a priori protocol? | Unclear | Not reported. |
| Followed a priori analysis plan? | Unclear | Not reported. |
| Confounding? | Not relevant | RCT. |

*Little et al. (2014)*

| **Item** | **Assessment** | **Support for assessment** |
| --- | --- | --- |
| Adequate sequence generation? | Unclear | Cluster randomization at teacher level. No information about the random sequence generation. |
| Allocation concealment? | Unclear | Unclear how the random sequence was generated. |
| Blinding? | 4 | Participants are unlikely to be blind to treatment status. |
| Incomplete outcome data addressed? | 1 | No explicit mention of student or teacher attrition. But 47 teachers participated and 2,150 students and Table 2 show data for 2,011 students and Table 3 for 2,028 students so missing data level is around 6%. |
| Selective reporting? | 2 | Do not show data at the level of randomisation. |
| Other bias? | 3 | In total, 2,150 students and 47 teachers participated in the study. 4 schools, of which 12 intervention and 6 control teachers came from the same school (West) as it was organised in ”houses” and the 3 “houses” were randomised. 27-12=15 treament teachers left for the 3 remaining schools and 20-6=14 control teachers. So effectively the number randomised is 32 and not on the same level at all schools. There are some large imbalances (on the student level) on the pre-intervention fluency test in the South and North schools and some imbalance on GMRT in the North school. |
| Followed a priori protocol? | Unclear | Not reported. |
| Followed a priori analysis plan? | Unclear | Not reported. |
| Confounding? | Not relevant | RCT. |

*Lovett et al. (2012)*

| **Item** | **Assessment** | **Support for assessment** |
| --- | --- | --- |
| Adequate sequence generation? | High | QES: “The study was quasi-experimental in that randomization of participants to the treatment or control condition was contaminated” (p. 154). Originally a waitlist RCT. |
| Allocation concealment? | High | Non-random assignment. |
| Blinding? | 4 | Participants are unlikely to be blind to treatment status. |
| Incomplete outcome data addressed? | 1 | Attrition during the study is very low. |
| Selective reporting? | 1 | Nothing suggests selective reporting. |
| Other bias? | 3 | Unclear how many that was originally planned to be in the intervention group. It is much larger than the control group (see e.g., p. 154). |
| Followed a priori protocol? | Unclear | Not reported. |
| Followed a priori analysis plan? | Unclear | Not reported. |
| Confounding? | 3 | Originally a randomised assignment, but parts of the control group got the intervention. No method for identifying relevant confounders is described. Control for age, gender and pre-test. Sample is quite well balanced on all pre-test measures. The few differences are mostly in favor of the control group, which speaks against selection on ability. But more students with English as a first language than English Language Learners are assigned to the intervention. Use a hierarchical linear model with age, gender, and two pre-test measures as covariates. |

*Lugo (2005)*

| **Item** | **Assessment** | **Support for assessment** |
| --- | --- | --- |
| Adequate sequence generation? | High | Individual randomization within classes. “The student in each class selected a colored ticket out of a hat—to either a regular classroom instruction group or a multimedia-based instruction” (p. 38). |
| Allocation concealment? | High | Sequential treatment, possibly not well-concealed. |
| Blinding? | 4 | Participants are unlikely to be blind to treatment status. |
| Incomplete outcome data addressed? | 3 | “Fifteen students dropped out of the study during the intervention period. These participants were subsequently excluded from the sample, leaving only 90 students remaining.” (p. 82). “Five students were absent over a four-week period and subsequently were eliminated from the study.” (p. 83). No analysis of differential attrition. |
| Selective reporting? | 1 | Nothing suggests selective reporting. |
| Other bias? | 3 | There is no balance test presented. |
| Followed a priori protocol? | Unclear | Not reported. |
| Followed a priori analysis plan? | Unclear | Not reported. |
| Confounding? | Not relevant | RCT. |

*Okkinga et al. (2018)*

| **Item** | **Assessment** | **Support for assessment** |
| --- | --- | --- |
| Adequate sequence generation? | Unclear | No information about sequence generation. |
| Allocation concealment? | Unclear | Unclear sequence generation. |
| Blinding? | 4 | No indication that any participant group is blind to treatment status. |
| Incomplete outcome data addressed? | 2 | One teacher becomes terminally ill and the class is not included in the analysis. 7 students leave the study and 6 new students enter. Incomplete data due to missing values reduce the number of students from 369 to 338 in the analysis. No test of differential attrition, but the levels are low. |
| Selective reporting? | 1 | Nothing suggests selective reporting. |
| Other bias? | 2 | Significant imbalance on the share of girls. Pre-test differences on reading comprehension, vocabulary, and IQ are small. Due to the inclusion of mediators, we cannot use the model-based estimates, where confounders are otherwise included, to calculate effect sizes. |
| Followed a priori protocol? | Unclear | Not reported. |
| Followed a priori analysis plan? | Unclear | Not reported. |
| Confounding? | Not relevant | RCT. |

*Olson et al. (2012)*

| **Item** | **Assessment** | **Support for assessment** |
| --- | --- | --- |
| Adequate sequence generation? | High | Cluster randomization at teacher level with grade x school blocking. Students were randomly assigned to classrooms. Because of resource constraints one English language arts classroom per teacher was selected to include in the study and a random sample of approximately 50% of the students within each class were used for analysis. As the selection of the teachers’ classrooms does not seem random, we treated this study as a QES. |
| Allocation concealment? | High | Non-random assignment. |
| Blinding? | 4 | Participants are unlikely to be blind to treatment status. |
| Incomplete outcome data addressed? | 3 | Approximately 30% of sample was lost due to teacher lay-offs. However researchers found no substantial differences between intervention and control teachers due to attrition. In addition 6 12th-grade classrooms (6%) that were included in the randomization at the beginning of the study were excluded in the analysis of CST and ALA as students did not take the CST test |
| Selective reporting? | 2 | Do not report post-test means. |
| Other bias? | 1 | No other bias detected. |
| Followed a priori protocol? | Unclear | Not reported. |
| Followed a priori analysis plan? | Unclear | Not reported. |
| Confounding? | 2 | Randomised study but one part of the assignment is non-random. Method for identifying relevant confounders not described. Baseline differences on class room level is shown for pre-test, gender, percent Latino, English language learner whose primary home language is Spanish, and eligible for free- or reduced price lunch. Almost no imbalances. Age and grade are not considered. Only pre-test is included in an HLM regression. |

*Olson et al. (2015)*

| **Item** | **Assessment** | **Support for assessment** |
| --- | --- | --- |
| Adequate sequence generation? | High | Partly randomised study, but as classrooms and students seem to be non-randomly selected (“Therefore, when a teacher had multiple sections of regular English language arts, we selected the one classroom that had the highest percentage of students at or above the intermediate level on the CELDT because the Pathway materials were designed specifically for these students.”), we treated it as a QES. |
| Allocation concealment? | High | Non-random assignment. |
| Blinding? | 3 | Teachers within the same schools are assigned to intervention and control after consent, so not blind to treatment status. Parents and students might have been. Test is a statewide exam, so tester is likely to be blind to treatment status. |
| Incomplete outcome data addressed? | 3 | “There was teacher attrition between the years (Pathway teachers declined from 49 to 41 and controls from 46 to 40), and this altered the demographics of their students (where the Pathway students were receiving the programme for the first time and the controls had never experienced the programme).” (p. 12-13). There is some incomplete outcome data also in Year 1 (some concerns the writing test which we do not use), they use only the CAHSEE, which is taken by 10th graders. Not all take this test either, but comparing shares/numbers in table 1 & 72(p. 48-49) with table 6 & 7 (p. 53-54) attrition seems small. |
| Selective reporting? | 1 | Nothing suggests selective reporting. |
| Other bias? | 2 | Sample is well balanced on teacher characteristics, and on 1st year student characteristics, including pre-tests. Second year is less well balanced due to attrition. |
| Followed a priori protocol? | Unclear | Not reported. |
| Followed a priori analysis plan? | Unclear | Not reported. |
| Confounding? | 4 | Randomised study but one part of the assignment is non-random. Method for identifying relevant confounders not described. Gender, race, grade, language proficiency status, and free/reduced lunch eligibility are considered. There is some pre- intervention imbalances. For the CAHSEE (which is the test we can use) they run logistic regressions with an intervention indicator plus controls as independent variables. Note that there does not seem to be a pre-test variable included (although language proficiency is included) when the CAHSEE is used as outcome variable. Specification of outcome variable is a bit unclear, but seem to contrast those who passed and failed. |

*Papalewis (2004)*

| **Item** | **Assessment** | **Support for assessment** |
| --- | --- | --- |
| Adequate sequence generation? | High | Non-random assignment. |
| Allocation concealment? | High | Non-random assignment. |
| Blinding? | 4 | Participants are unlikely to be blind to treatment status. |
| Incomplete outcome data addressed? | 2 | Attrition and missing data total for T group is 14%. C is matched so no missing data by construction |
| Selective reporting? | 1 | Nothing suggests selective reporting. |
| Other bias? | 1 | No other bias detected. |
| Followed a priori protocol? | Unclear | Not reported |
| Followed a priori analysis plan? | Unclear | Not reported. |
| Confounding? | 4 | Use a type of matching procedure based on finding students with similar pre-test scores and approximately the same percentages of gender, ethnicity and language group as the treated students (p.31). Very small differences between intervention and control group on the pre-intervention test scores. Nothing else concerning the control group is reported. |

*Penney (2002)*

| **Item** | **Assessment** | **Support for assessment** |
| --- | --- | --- |
| Adequate sequence generation? | High | Teachers (non-randomly) assigned 21 students to experiment and 12 students to control. |
| Allocation concealment? | High | Non-random assignment. |
| Blinding? | 4 | Participants are unlikely to be blind to treatment status. |
| Incomplete outcome data addressed? | 2 | 46 students initially agreed to participate, returning permission slips and completing the first assessment. Nine students (5 female and 4 male) dropped out of school after the first assessment. Two male students received the initial assessment and began the tutoring programme, but refused to complete it. Two control participants did not receive the second assessment after the control period and before the tutoring programme. |
| Selective reporting? | 1 | Nothing suggests selective reporting. |
| Other bias? | 1 | No other bias detected. |
| Followed a priori protocol? | Unclear | Not reported. |
| Followed a priori analysis plan? | Unclear | Not reported. |
| Confounding? | 4 | Teachers assign students to condition (21 treated and 12 control). All students were Caucasian and age is balanced too. Imbalance on gender (57% boys in the intervention group and 75% in the control group), and some pre-intervention test imbalance. Only pre-tests are controlled for in the analysis by an ANCOVA. |

*Prediger & Wessel (2018)*

| **Item** | **Assessment** | **Support for assessment** |
| --- | --- | --- |
| Adequate sequence generation? | Unclear | No information about sequence generation. |
| Allocation concealment? | Unclear | Unclear sequence generation. |
| Blinding? | 4 | No indication that any participant group is blind to treatment status. |
| Incomplete outcome data addressed? | Unclear | Treatment group Diskursive Sprachförderung = 83 at pre-test, Treatment group Lexikalischer Sprachförderung = 103 at pre-test, control group 157 at pre-test. It is unclear why the groups differ in size. Seem to be no attrition at all, but it is unclear whether they are just using the students that are still in the study at follow-up. |
| Selective reporting? | 1 | Nothing suggests selective reporting. |
| Other bias? | 3 | No information about how many of the eligible students that was randomized, and the randomization is not described, only mentioned in a figure. Reasonably small differences on pre-test as well as student characteristics. |
| Followed a priori protocol? | Unclear | Not reported. |
| Followed a priori analysis plan? | Unclear | Not reported. |
| Confounding? | Not relevant | RCT. |

*Roberts et al. (2013)*

| **Item** | **Assessment** | **Support for assessment** |
| --- | --- | --- |
| Adequate sequence generation? | Unclear | Randomized within schools using a 2:1 assignment ratio. No information about the random sequence generation. |
| Allocation concealment? | Unclear | Unclear how the random sequence was generated. |
| Blinding? | 4 | No indication that any participant group is blind to treatment status. |
| Incomplete outcome data addressed? | 4 | Overall attrition was considerable in present study. Total sample size at start is 768, at the end it is 166. A redrawing of district boundaries and that they trim both intervention and control groups using random sampling due to budget constraints are the main reasons. There are significant differences over single variables, and they do not check the joint significance in their attrition analysis. |
| Selective reporting? | 1 | Nothing suggests selective reporting. |
| Other bias? | 1 | No other bias detected. |
| Followed a priori protocol? | Unclear | Not reported. |
| Followed a priori analysis plan? | Unclear | Not reported. |
| Confounding? | Not relevant | RCT. |

*Rossiter (2012)*

| **Item** | **Assessment** | **Support for assessment** |
| --- | --- | --- |
| Adequate sequence generation? | High | QES: there was no random assignment of the students. |
| Allocation concealment? | High | Non-random assignment. |
| Blinding? | 4 | No indication that any participant group is blind to treatment status. |
| Incomplete outcome data addressed? | 3 | Only students who have data from all three years are used. Starts with a total of 214 students, 167 students in the Non-Math Recovery group and 47 students in the Math Recovery group. Further reduced with 62 students due to missing test data. Missing data for intervention group is 32% and control group 28%. |
| Selective reporting? | 1 | Nothing suggests selective reporting. |
| Other bias? | 1 | No other bias detected. |
| Followed a priori protocol? | Unclear | Not reported. |
| Followed a priori analysis plan? | Unclear | Not reported. |
| Confounding? | 2 | Method for identifying relevant confounders not described. No imbalance on gender, race and special education. All from same grade but age is not considered. Some imbalance on pre-test, the difference is significant but not large. Use a hierarchical linear regression in the analysis with gender, race and pre-test, plus interactions. |

*Rutt et al. (2015)*

| **Item** | **Assessment** | **Support for assessment** |
| --- | --- | --- |
| Adequate sequence generation? | Low | Use an Excel-based algorithm to assign treatment to three groups: intervention, control and a reserve group. |
| Allocation concealment? | Low | Non-sequential allocation. |
| Blinding? | 3 | An external tester corrects the tests, who is unaware of treatment status. However, the teaching assistants who deliver the intervention administer the tests. No other participant is likely to be blind to treatment status. |
| Incomplete outcome data addressed? | 2 | Overall attrition rate is about 10% in the intervention group, and about 9% percent in the control group. Most of these where students that never transferred to the relevant secondary school. There is no significant differential attrition between the intervention and control group. |
| Selective reporting? | 1 | Researchers follow the protocol. |
| Other bias? | 1 | No significant or substantial baseline imbalances over pupil characteristics. Some pupils are selected from a reserve list, but this is a very small number (3 in the ITT analysis). |
| Followed a priori protocol? | Yes | Mentioned on p. 9, where a link is also provided. |
| Followed a priori analysis plan? | Yes | Protocol contains an analysis plan. |
| Confounding? | Not relevant | RCT. |

*Schüler-Meyer et al. (2019)*

| **Item** | **Assessment** | **Support for assessment** |
| --- | --- | --- |
| Adequate sequence generation? | Unclear | No information about sequence generation. |
| Allocation concealment? | Unclear | Unclear sequence generation. |
| Blinding? | 4 | Blinding is not discussed. No participant group is likely to be blind to treatment status. |
| Incomplete outcome data addressed? | 1 | Start with 128 eligible students, who are randomized to treatment and control. Apparently no attrition. |
| Selective reporting? | 1 | Nothing suggests selective reporting. |
| Other bias? | 3 | Some imbalance on the pre-test in math and on socioeconomic status. Use an ANOVA to control for pre-test, SES, intelligence and language proficiency but do not report the results in a way that we can use to calculate effect sizes. Unclear stratification and assignment procedure, stratification not taken into account in analysis. |
| Followed a priori protocol? | Unclear | Not reported. |
| Followed a priori analysis plan? | Unclear | Not reported. |
| Confounding? | Not relevant | RCT. |

*Shell (1998)*

| **Item** | **Assessment** | **Support for assessment** |
| --- | --- | --- |
| Adequate sequence generation? | Unclear | Two-step randomization procedure but no information about the random sequence generation. |
| Allocation concealment? | Unclear | Unclear how the random sequence was generated. |
| Blinding? | 4 | Consent is sought from parents, students and teachers beforehand. The investigator administers the tests. So no participant is likely to be blind to treatment status. |
| Incomplete outcome data addressed? | 2 | Original sample after consent given is 69 students, final sample 64. |
| Selective reporting? | 1 | Nothing suggests selective reporting. |
| Other bias? | 4 | Large imbalances on all pre-tests between the control and the two intervention groups. The study uses a MANCOVA procedure to partially control for baseline imbalances, but there is not enough information in the study so that we can use these results to calculate effect sizes. Prior to hand-scoring the achievement pre-test, test protocols of ninth and tenth grade subjects from special education with serious emotional disturbance were separated from the total testing group and randomly assigned to one of the intervention or control groups. A similar procedure is used for the general education group. |
| Followed a priori protocol? | Unclear | Not reported. |
| Followed a priori analysis plan? | Unclear | Not reported. |
| Confounding? | Not relevant | RCT. |

*Solis et al. (2015)*

| **Item** | **Assessment** | **Support for assessment** |
| --- | --- | --- |
| Adequate sequence generation? | Unclear | Students randomised within schools, but no information about how the random sequence was generated. |
| Allocation concealment? | Unclear | Unclear how the random sequence was generated. |
| Blinding? | 4 | Participants are unlikely to be blind to treatment status. |
| Incomplete outcome data addressed? | 3 | Attrition rate for intervention group is 36% and for control 5%. |
| Selective reporting? | 2 | The number of control classes is not reported and it is not reported how the control students is divided on schools. |
| Other bias? | 4 | Students were placed in five classes with 5-9 students in each. One intervention teacher taught two groups of five students, each group at a different school. The other intervention teacher taught one group of nine students at the one school and one group of four at another school. There are three schools and five classes and 25 treated students, which does not add up. The number of control classes is not reported |
| Followed a priori protocol? | Unclear | Not reported. |
| Followed a priori analysis plan? | Unclear | Not reported. |
| Confounding? | Not relevant | RCT. |

*Somers et al. (2010)*

| **Item** | **Assessment** | **Support for assessment** |
| --- | --- | --- |
| Adequate sequence generation? | Low | Within each district, high schools were randomly assigned to one of the two interventions (there were an even number of schools in each district, 7 with 4 schools and 3 with 2 schools). Within each high school, eligible students were randomly assigned to intervention/control. Computerized random assignment of students was conducted by MDRC staff. |
| Allocation concealment? | Low | Non-sequential allocation. |
| Blinding? | 3 | Statewide tests, so tester is most likely blind. No other participant is likely to be blind to treatment status. |
| Incomplete outcome data addressed? | 3 | Only a subset of the study districts administered state tests; moreover, in most districts students only take the test when they have completed the relevant coursework. Less than 50 percent of students in the full study sample have state test data in any given year. This problem is larger for the follow-up tests, where there is more attrition, but importantly differential attrition does not seem to be a large problem for any sample. |
| Selective reporting? | 1 | Nothing suggests selective reporting. |
| Other bias? | 1 | Very small differences at baseline on the tests we use, some but not large differences on other characteristics. |
| Followed a priori protocol? | Unclear | Not reported. |
| Followed a priori analysis plan? | Unclear | Not reported. |
| Confounding? | Not relevant | RCT. |

*Stevens (2003)*

| **Item** | **Assessment** | **Support for assessment** |
| --- | --- | --- |
| Adequate sequence generation? | High | QES, non-random assignment. |
| Allocation concealment? | High | Non-random assignment. |
| Blinding? | 3 | Control group likely to be unaware of treatment status. No other participants are likely to be blind to treatment status. |
| Incomplete outcome data addressed? | Unclear | Nothing reported. |
| Selective reporting? | 1 | Nothing suggests selective reporting. |
| Other bias? | 1 | No indication of other bias. |
| Followed a priori protocol? | Unclear | Not reported. |
| Followed a priori analysis plan? | Unclear | Not reported. |
| Confounding? | 3 | Match on school level. Two experimental schools are matched with three comparison schools in same district. Schools were matched on their initial achievement in reading and language arts, and on ethnicity and SES. There are significant differences on the pre-test at the pre-test at the individual level. Use a MANCOVA nesting classes within schools. |

*Swanson et al. (2015)*

| **Item** | **Assessment** | **Support for assessment** |
| --- | --- | --- |
| Adequate sequence generation? | Unclear | Random assignment of teachers’ classes to intervention and control groups, but no information about how the random sequence was generated. |
| Allocation concealment? | Unclear | Unclear how the random sequence was generated. |
| Blinding? | 3 | Teachers teach both intervention and control classes. Parents give consent. Students are likely to aware as well, due to within school contacts. Tester explicitly unaware of intervention status. |
| Incomplete outcome data addressed? | 1 | Attrition is said to be very low but no exact number is given (less than 13% though). |
| Selective reporting? | 1 | Nothing suggests selective reporting. |
| Other bias? | 2 | Teachers are teaching both control and intervention classes. “An ongoing process of providing checks and feedback to teachers assured that no intervention components were used in the comparison classes. [...] Data indicate (see Tables 2 and 3) that PACT intervention components were rarely observed in comparison classes, and when they were, the component was recognizable at the very lowest levels of implementation.” (p. 433). Missing data is multiply imputed, unclear if imputed for both covariates and dependent variables. |
| Followed a priori protocol? | Unclear | Not reported. |
| Followed a priori analysis plan? | Unclear | Not reported. |
| Confounding? | Not relevant | RCT. |

*Swanson et al. (2016)*

| **Item** | **Assessment** | **Support for assessment** |
| --- | --- | --- |
| Adequate sequence generation? | Unclear | Students are randomly assigned to intervention or typical practice control group, but there is no information about how the random sequence was generated. |
| Allocation concealment? | Unclear | Unclear how the random sequence was generated. |
| Blinding? | 3 | Trained research personnel who were uninformed of treatment status administered the assessments. No indication that any other participant is blind to treatment status. |
| Incomplete outcome data addressed? | 2 | There is some attrition, but no indication of substantial differential attrition between intervention and control groups. |
| Selective reporting? | 2 | Report imputed pre-test and post-test scores but not the raw scores. |
| Other bias? | 1 | No indication of other bias. |
| Followed a priori protocol? | Unclear | Not reported. |
| Followed a priori analysis plan? | Unclear | Not reported. |
| Confounding? | Not relevant | RCT. |

*Tidd et al. (2018)*

| **Item** | **Assessment** | **Support for assessment** |
| --- | --- | --- |
| Adequate sequence generation? | High | Non-random sequence generation. |
| Allocation concealment? | High | High risk sequence generation. |
| Blinding? | 3 | 1.735 students in other schools in the same school district identified as comparison group after the intervention, they and their teachers ought to be blind to treatment status. |
| Incomplete outcome data addressed? | 1 | Sample is selected, both treatment and control group, based on the availability of post-test data. |
| Selective reporting? | 1 | They do not report post-test scores and SD, only test whether a student passes or not. |
| Other bias? | 1 | They start out with 6 schools and then reduce the sample, unclear if this means that there are still students in all six schools in the final analysis sample. |
| Followed a priori protocol? | Unclear | Not reported. |
| Followed a priori analysis plan? | Unclear | Not reported. |
| Confounding? | 3 | Gender, SES, ethniticity, limited English proficiency and pre-test score used as covariates and for matching. Unclear why the six schools implement the intervention and why control schools do not. Reasonably well-balanced after propensity score weighting. |

*Tijms et al. (2017)*

| **Item** | **Assessment** | **Support for assessment** |
| --- | --- | --- |
| Adequate sequence generation? | Unclear | No information about sequence generation. |
| Allocation concealment? | Unclear | Unclear sequence generation. |
| Blinding? | 4 | Parents are informed. Students in the same classes are randomized. No information that any other participant group is blind to treatment status. |
| Incomplete outcome data addressed? | 2 | Start with 100 students. Pre-test or post-test data were missing for four intervention-group students and for six control group students. |
| Selective reporting? | 1 | Nothing suggests selective reporting. |
| Other bias? | 2 | Students randomized within classes increase risk of spillovers. A few moderate imbalances in Table 1, but none large. |
| Followed a priori protocol? | Unclear | Not reported. |
| Followed a priori analysis plan? | Unclear | Not reported. |
| Confounding? | Not relevant | RCT. |

*Travillian (2011)*

| **Item** | **Assessment** | **Support for assessment** |
| --- | --- | --- |
| Adequate sequence generation? | High | QES, non-random assignment. |
| Allocation concealment? | High | Non-random assignment. |
| Blinding? | 4 | Participants are unlikely to be blind to treatment status. |
| Incomplete outcome data addressed? | 1 | There is no indication of any attrition. |
| Selective reporting? | 1 | Nothing suggests selective reporting. |
| Other bias? | 1 | No indication of other bias. |
| Followed a priori protocol? | Unclear | Not reported. |
| Followed a priori analysis plan? | Unclear | Not reported. |
| Confounding? | 3 | Match pairs on gender, grade, pre-test scores, ethnicity and a risk ratio measuring the risk of not graduating. Use an ANCOVA in the analysis. |

*Vaughn et al. (2010)*

| **Item** | **Assessment** | **Support for assessment** |
| --- | --- | --- |
| Adequate sequence generation? | Unclear | No information about how the random sequence was generated. |
| Allocation concealment? | Unclear | Unclear how the random sequence was generated. |
| Blinding? | 4 | Randomisation appears to have been done within schools. There is no indication that any participant is blind to treatment status. |
| Incomplete outcome data addressed? | 4 | Attrition from randomisation to the start of middle school is between 20-25% for the three groups, but not much different between them. There is further attrition during the intervention, resulting in nearly 50% total attrition from randomisation to post-test in the two intervention groups, and around 30% in the control group. “For students who left the study, performance on pre-test measures did not differ significantly from that of students who remained in the study (p > 0.05)” (p. 5). Not clear though whether the same type of students leave the intervention and control groups. |
| Selective reporting? | 1 | Nothing suggests selective reporting. |
| Other bias? | 1 | “The proportions of students from the treatments did not differ in terms of site, sex, free or reduced lunch status, or ethnicity. Students in the two struggling reader groups also did not differ in terms of their struggling reader category (e.g., failure, bubble, or special education) or in age (p > 0.05)” (p. 5-6)” |
| Followed a priori protocol? | Unclear | Not reported. |
| Followed a priori analysis plan? | Unclear | Not reported. |
| Confounding? | Not relevant | RCT. |

*Vaughn et al. (2011)*

| **Item** | **Assessment** | **Support for assessment** |
| --- | --- | --- |
| Adequate sequence generation? | Unclear | Within-teacher randomisation of classes to intervention and control groups. No information about how the random sequence was generated. |
| Allocation concealment? | Unclear | Unclear how the random sequence was generated. |
| Blinding? | 3 | “All student measures were administered to students by trained research personnel who were blind to students’ condition” (p. 951). Unclear whether other participants were blind to treatment status, but the study uses a randomised block design where classes are randomised within schools to intervention and control, so it seems most likely that teachers and students were aware. |
| Incomplete outcome data addressed? | 3 | “A total of 866 students in the initial analysis sample (student cases with missing cluster or grouping data or with missing data on all model-related observed scores were excluded for analysis). Patterns of missing data varied by outcome, so actual sample sizes differed by analysis. For the primary comparison [...], the sample size was 782, with 382 students in the comparison condition and 400 in treatment.” (p. 944) The final group is smaller, 369 in treatment and 354 in the control group. |
| Selective reporting? | 2 | There are no model results for the Metacomprehension test, only raw means. However, it is not a reading test per se, but tests knowledge of reading strategies. Do not report the numbers of students that moved between intervention and control classes. |
| Other bias? | 4 | "Group comparability (i.e., the effectiveness of randomization) was confirmed by comparing pretest scores by group on each of the three outcome measures. [...] For Gates and AIMS, no significant differences were found at pretest (t = –0.562 and 0.997, respectively; Table 4). For TOSREC, the difference between groups at pretest was significant (t = –2.212)” (p. 944-945). The study uses a within-teacher randomization that could result in treatment spilling over to the control group, however measures are taken to prevent this. There was movement across treatment conditions during the school year. No numbers are given though, it is only stated that: it was fairly limited, occurred in both directions (i.e., from treatment to control and from control to treatment), and apparently non-systematic. |
| Followed a priori protocol? | Unclear | Not reported. |
| Followed a priori analysis plan? | Unclear | Not reported. |
| Confounding? | Not relevant | RCT. |

*Vaughn et al. (2013)*

| **Item** | **Assessment** | **Support for assessment** |
| --- | --- | --- |
| Adequate sequence generation? | Unclear | Within-teacher randomisation of classes to intervention and control groups. No information about how the random sequence was generated. |
| Allocation concealment? | Unclear | Unclear how the random sequence was generated. |
| Blinding? | 3 | Testers are unaware of treatment status. No other participants are likely to be unaware of treatment status. |
| Incomplete outcome data addressed? | 3 | “A preliminary screening of the student data indicated a systematically irregular pattern of scores on the Gates–MacGinitie. In the comparison condition for one teacher, students made an average gain of approximately 12.5 standard score points, with the gains evenly distributed across the 22 cases. In the same teacher’s treatment condition (n = 24), the average gain was about –0.8. The gain in the comparison group appeared to be due to non-completion of the Gates–MacGinitie assessment at pretest (no student in this class completed more than half of the items at pretest), resulting in artificially deflated pretest scores. This pattern was not evident in any other teacher’s CSR or TP classes, and it indicates a threat to the validity of this teacher’s data. Accordingly, we elected to remove this teacher from the analysis of the Gates–MacGinitie data, reducing the available Gates-specific sample to 482 students across 45 classes and 11 teachers.” (p. 142-143). “In sum, overall attrition was relatively minimal (11%) and there was no evidence of differential attrition” (p. 144). The latter test is by variable, and there is no joint test. |
| Selective reporting? | 2 | There is no multilevel model/adjusted means presented for the struggling readers sample. |
| Other bias? | 3 | Teachers are teaching both control and treatment classes. “Overall, teachers in typical classrooms received a procedural fidelity average rating of 2.34, indicating that instruction was different from CSR classroom instruction (3.20)” and “In the 2nd year of the study, issues of spillover were infrequently identified or observed. Teachers reported and we observed that they understood clearly the instructional components related to CSR and the need to contain CSR strategies to treatment classrooms. Spillover of CSR strategy instruction was not observed. However, we suspect that there may have been an unanticipated overall teacher effect of participating in the 2 years of CSR professional development that influenced the quality of instruction in the typical classes.” (p. 148). See also results of factor model that indicates relatively large differences in intervention strategy implementation (p. 155). Furthermore: some of the “instructional practices are not “unique” to CSR but are represented in a range of reading intervention practices” (p. 159), so may well be part of treatment as usual. |
| Followed a priori protocol? | Unclear | Not reported. |
| Followed a priori analysis plan? | Unclear | Not reported. |
| Confounding? | Not relevant | RCT. |

*Vaughn et al. (2015)*

| **Item** | **Assessment** | **Support for assessment** |
| --- | --- | --- |
| Adequate sequence generation? | Unclear | Within-school randomisation, but no information on how the random sequence was generated. |
| Allocation concealment? | Unclear | Unclear how the random sequence was generated. |
| Blinding? | 4 | Participants are likely to be aware of treatment status. |
| Incomplete outcome data addressed? | 3 | 375 (82.1%) students remained in the study at the beginning of the intervention, with additional attrition across the subsequent three assessment waves. At the end of ninth grade, 344 (75.3%) students remained; at the beginning of 10th grade, 325 (71.1%) remained; and at the end of tenth grade, 306 (67%) remained with complete data on all measures. No significant differential attrition between intervention and control groups. |
| Selective reporting? | 1 | Nothing suggests selective reporting. |
| Other bias? | 2 | Relatively small imbalances on most pre-intervention characteristics, some but not excessively large pre-test differences. |
| Followed a priori protocol? | Unclear | Not reported. |
| Followed a priori analysis plan? | Unclear | Not reported. |
| Confounding? | Not relevant | RCT. |

*Vaughn et al. (2017)*

| **Item** | **Assessment** | **Support for assessment** |
| --- | --- | --- |
| Adequate sequence generation? | Unclear | No information about sequence generation. |
| Allocation concealment? | Unclear | Unclear sequence generation. |
| Blinding? | 4 | Nothing to indicate blinding of treatment status. |
| Incomplete outcome data addressed? | 2 | Attrition rate of 20,1% for Gates MacGinitie Reading test. Analyses yields no significant results concerning systematic attrition of students (p. 25) |
| Selective reporting? | 1 | Nothing suggests selective reporting. |
| Other bias? | 3 | There is an overall gender imbalance but not reported separately by English language learner (EL) status (and we only include EL students). There is an imbalance on EL students with more ELs in the treatment classes. Pre-tests shown separated by EL status, some imbalance on two of three pre-tests (only one standardized test, the other two researcher developed and there is an imbalance of approximately 0.3 SD on the standardized pre-test). There may be a risk of teacher contamination as the study applies within teacher randomisation. |
| Followed a priori protocol? | Unclear | Not reported. |
| Followed a priori analysis plan? | Unclear | Not reported. |
| Confounding? | Not relevant | RCT. |

*Wanzek et al. (2011)*

| **Item** | **Assessment** | **Support for assessment** |
| --- | --- | --- |
| Adequate sequence generation? | Unclear | Randomly assigns students to a supplemental reading intervention, but no information about how the random sequence was generated. |
| Allocation concealment? | Unclear | Unclear how the random sequence was generated. |
| Blinding? | 4 | Participants are likely to be aware of treatment status. |
| Incomplete outcome data addressed? | 3 | Total number is 76 in intervention and 59 in control group. 11 and 4 students from intervention and control groups leave the study from pre- to post-test. 36 and 44 leave between pre-test and follow-up (we cannot use the follow-up as the results are reported as latent growth curve). There is no formal analysis of differential attrition. |
| Selective reporting? | 1 | Nothing suggests selective reporting. |
| Other bias? | 3 | The intervention group has consistently higher pre-test scores, but the differences are relatively small. Control group students may have gotten more remedial instruction, although there is no systematic evidence to back this up (see p. 77 for discussion). |
| Followed a priori protocol? | Unclear | Not reported. |
| Followed a priori analysis plan? | Unclear | There is a section mentioning an analysis plan, but unclear when this plan was made. |
| Confounding? | Not relevant | RCT. |

*Weichenthal (1985)*

| **Item** | **Assessment** | **Support for assessment** |
| --- | --- | --- |
| Adequate sequence generation? | Unclear | The subjects were randomly assigned to one of the three treatment groups, but there is no information about how the random sequence was generated. |
| Allocation concealment? | Unclear | Unclear how the random sequence was generated. |
| Blinding? | 3 | Parental permission for participation in the study was not obtained because subjects were not treated in a manner that was different from their usual instruction in the resource-specialist programme. Not clear if control and intervention groups came from same school. Also unclear who performs the tests, but no indication in the study that these groups were blinded. |
| Incomplete outcome data addressed? | 1 | Almost no attrition, 88 out of 90 originally assigned to the programme complete it. |
| Selective reporting? | 1 | Nothing suggests selective reporting. |
| Other bias? | 1 | Sample seems to be balanced on baseline age and IQ, although no statistical test to support this. |
| Followed a priori protocol? | Unclear | Not reported. |
| Followed a priori analysis plan? | Unclear | Not reported. |
| Confounding? | Not relevant | RCT. |

*Wexler et al. (2010)*

| **Item** | **Assessment** | **Support for assessment** |
| --- | --- | --- |
| Adequate sequence generation? | Unclear | Students were paired within classes and pairs were randomly assigned to one of three groups. No further information about how the random sequence was generated. |
| Allocation concealment? | Unclear | Unclear how the random sequence was generated. |
| Blinding? | 4 | Participants are likely aware of treatment status. |
| Incomplete outcome data addressed? | 2 | Attrition rate is 9%. |
| Selective reporting? | 1 | Nothing suggests selective reporting. |
| Other bias? | 2 | Student pairs were disrupted during the intervention: “The primary limitation in this study resulted from chronic absenteeism of participating students. When one student was absent, repairing that student with another partner was often difficult because of social issues or because the teacher did not have another partner whose reading level was appropriate for the student. This also influenced the overall total time in intervention. Because of chronic absenteeism, many students in this study were forced to work in trios, rotate partners, or work alone, and some students had to work independently several times when their partners were repeatedly absent” (p. 9-10). |
| Followed a priori protocol? | Unclear | Not reported. |
| Followed a priori analysis plan? | Unclear | Not reported. |
| Confounding? | Not relevant | RCT. |

*White (2000)*

| **Item** | **Assessment** | **Support for assessment** |
| --- | --- | --- |
| Adequate sequence generation? | Unclear | At the eighth-grade level, students were matched on Stanford 9 mathematics score, gender, ESL status, and ethnicity. Then, one student from each matched pair was randomly assigned to either the experimental or control group, and his/her partner was then assigned to the opposite group. No information about how the random sequence was generated. |
| Allocation concealment? | Unclear | Unclear how the random sequence was generated. |
| Blinding? | 4 | Participants are likely aware of treatment status. |
| Incomplete outcome data addressed? | 3 | Initially, 43 at-risk eighth-grade students were identified as potential participants in the study. Two tutors chose not to participate in the study. Thus, these two students and their matched partners from the control group were dropped. In addition, two tutors and their matched partners were dropped from the study when the tutors’ assigned tutees missed more than two tutoring sessions. Lastly, two other tutors were dropped from the study, along with their matched partners, due to scheduling conflicts. One-eighth-grade student was dropped during the data analysis for not completing the posttest instruments. The final sample is comprised of 30 at-risk eighth-grade students. |
| Selective reporting? | 1 | Nothing suggests selective reporting. |
| Other bias? | 3 | Only minor imbalances between intervention and control group. It is unclear how the test items used are selected. We interpret the text as meaning that all previously released items from state tests are used, not just the ones that are close to the intervention content, but this is not explicitly stated. |
| Followed a priori protocol? | Unclear | Not reported. |
| Followed a priori analysis plan? | Unclear | Not reported. |
| Confounding? | Not relevant | RCT. |

*Wyllie (2008)*

| **Item** | **Assessment** | **Support for assessment** |
| --- | --- | --- |
| Adequate sequence generation? | High | QES, non-random assignment. |
| Allocation concealment? | High | Non-random assignment. |
| Blinding? | 4 | Participants are likely aware of treatment status. |
| Incomplete outcome data addressed? | Unclear | Not reported. |
| Selective reporting? | 4 | Not reported how many classes the programme consisted of nor from how many classes (or schools) the students were chosen from. |
| Other bias? | 4 | Not reported how the comparison group is selected. They are almost identical (on average) to the intervention group and equal in number. |
| Followed a priori protocol? | Unclear | Not reported. |
| Followed a priori analysis plan? | Unclear | Not reported. |
| Confounding? | 4 | Initially, 144 slots existed for eleventh-grade participants in the PSSA Prep Programme. Treated number is 96. Not reported how many from the comparison group opted out of the programme and how many were not recommended for the programme. Gender, economically disadvantaged and pre-test scores are considered. No substantial imbalances. Nothing is controlled for in the analysis. |

**Studies with a Too High Risk of Bias Rating**

In Table A5, we have listed the studies that were given a too high risk of bias rating, i.e., 5, on at least one item. There may have been more than one rating of 5, but the table below contains the primary reason plus an explanatory comment.

*Table A5. Studies not included in meta-analysis because of too high risk of bias.*

| **Study** | **Country** | **Test subject** | **Rated 5 on item?** | **Comment** |
| --- | --- | --- | --- | --- |
| Airhart (2005) | US | Reading | Other bias | Experimental group is located at one school, and comparison groups located at two different schools. |
| Alarilla (2016) | US | Math, reading | Other bias | Intervention group from one school and control group from one other school. |
| Alfassi (1998) | Israel | Reading | Other bias | Intervention group from one school and control group from one other school. |
| Azcoitia (1989) | US | Reading | Confounding | Use pre-test as covariate but do not show imbalances. No other confounder is considered. Two schools are involved but it is not reported whether there are treated/control in both schools. |
| Barrett (2011) | US | Math | Other bias | One intervention and one control school |
| Barrus (2014) | US | Math | Other bias | Randomize three class periods of a remedial algebra class to two alternative interventions and one control condition. All class periods share the same teacher. |
| Bayley-Hamlet (2018) | US | Reading | Confounding | No relevant confounders except pre-test described or identified, and some imbalance on one pre-test. Analyse gain scores for one of the outcomes. |
| Bellert (2009) | Australia | Math | Confounding | No confounders are considered by the researcher. |
| Berman (2000) | US | Math, reading | Other bias | Intervention in one school and another school was selected as the comparison group. |
| Beyer (1991) | US | Math, reading | Other bias | There are extensive implementation problems at both sites, and in one site there is no control group for the period where at least some students seem to get an intervention. In the other it is unclear if any student gets the intervention in more than small bits, and a further uncertainty around how students were assigned to intervention and control groups. |
| Biesinger & Crippen (2008) | US | Math | Confounding | Gender, ethnicity and pre-test scores are described but not controlled for. There is a 'built in' gain in the selection process: Select those students who obtained a satisfactory score on a test measure. Those who created accounts and visited the site at least once, but did not complete the programme (in the programme they are not allowed to move on to other topics until they show adequate progress) were discarded. The control group consists of the remaining students at the school who did not participate in the intervention. The second part of the analysis checks for imbalances, but does not control for these. Furthermore, in parts of the analysis, pre-test scores are not controlled for. |
| Bixler (2010) | US | Reading | Other bias | Control- and intervention groups belong to two different schools, intervention effect is confounded by the school effects. |
| Blinkhorn (2009) | US | Reading | Other bias | Separate tests and separate analysis in the content areas of U.S. History and biology respectively. Cannot separate teacher effect from intervention. |
| Brady (1990) | US | Reading | Other bias | "The training was conducted by me, the regular classroom teacher in the combined 7th-8th grade classroom during October and November. Each of the instructional groups was trained in the strategies for a half hour per day for five weeks." (p. 31). Interpret this as the researcher being the teacher for all intervention students. This makes it difficult to interpret the effects as being the result of the intervention. Furthermore, an imbalance on gender distribution, "The gender distribution was not equal, with four girls in the SMART group, two girls in the RT group, and no girls in the control group" (p. 28). |
| Bribiescas (2012) | US | Math, reading | Other bias | Intervention in one school and another school was selected as the control school. |
| Broome (2011) | US | Math, reading | Confounding | Only consider a risk ratio as a confounder in the in analysis, every student with a risk ratio greater than zero is included in the analysis. The risk ratio does not take into account gender and age. Not all of the at-risk students are offered intervention though, 23% were selected, due to capacity constraints. This seems to have resulted in large differences between the intervention and control group, both the levels and the development of the risk ratio is quite different. |
| Burlison & Chave (2014) | US | Reading | Confounding | No confounders are discussed and most are not controlled for, in fact, the control group is not described at all. The author compares the reading growth with a t-test (so implicitly include a pre-test). No information about pre-intervention balance between intervention and control group on any variable. |
| Callahan (2006) | US | Reading | Other bias | Intervention effects confounded by cohort effects, all treated in one cohort, and all control in one other. So if e.g., teacher quality differs over cohorts, this would not be controlled for. |
| Center (2015) | US | Reading | Other bias | Control group and intervention group are from different cohorts, which is not controlled for. |
| Choi & Lemberger (2010) | South Korea | Math, reading | Confounding | All eligible are offered the programme and control group are those who reject participation in intervention but accept being control group. |
| Cobb et al. (2006) | US | Math, reading | Other bias | Students from one intervention school are matched to students from several control schools. As intervention and school effects are completely confounded, the intervention effect cannot be recovered. |
| Cobb (2009) | US | Math | Other bias | One teacher, the researcher, instructs all intervention group students and two other teachers instruct the control group, so intervention effect confounded by teacher effects. |
| Coleman (2009) | US | Math, reading | Other bias | Intervention group is located at one school and control groups are located at two different schools. |
| Collier (2008) | US | Reading | Other bias | One intervention and two control schools. |
| Cornelius (2013) | US | Math | Confounding | Imbalance on ethnicity, which is not controlled for in any of the analyses. Intervention group is selected for intervention based on their performance (tier 2 students receive the intervention, and are chosen based on scoring at the 10th percentile of pre-test scores or below). Unlikely that intervention and control groups are comparable. |
| Curiel et al. (1980) | US | Reading | Confounding | Participants are enrolled in two different schools, not documented how control and intervention groups are distributed across schools. There seems to have been collected data on quite a few demographic variables, however none of these are used in the analysis and it is not mentioned if participants are matched on these. |
| Dalton (1986) | US | Math | Confounding | Nothing reported except students were systematically assigned to the three interventions. No confounders considered. |
| Dorman (2009) | US | Math, reading | Confounding | The CAHSEE test is described, and used in the calculation of gain scores. Nothing else is described. Compare students in a remediation programme to students with similar level of disabilities that does not get an intervention, but there is no information on why some get the intervention and others do not. No formal matching and some moderately to large imbalance for the pre-tests. Nothing is controlled for in the analysis. |
| Dougherty (2010) | US | Reading | Other Bias | Control- and intervention groups belong to two different schools. |
| Derico (2017) | US | Math, reading | Confounding | Compare students taught in an inclusive setting and students taught in resource setting. No adjustment for confounding. |
| Drew (2015) | US | Math, reading | Confounding | Use a "fuzzy RD”, but the assumptions behind this design (similar to instrumental variables, IV) are not discussed and no IV analyses are performed. The 200 (control group) and 150 students (intervention group) closest to the cutoff is used in the analysis. As this is 52% and 60% of the total groups, it reasonably includes many students that are far away from the cutoff. There is a standard deviation difference between top and bottom in this distribution. The evidence of a discontinuity in some of these figures is weak. The effect estimates seem to rely only on a linear regression of an intervention indicator and the composite pre-test score as a covariate. No test of manipulation of the forcing variable is performed and there is no imbalance test. |
| Ehmke et al. (2010) | Germany | Math | Confounding | A lot of matching variables are used, but the means of these variables are only shown for the retained group and the total unmatched comparison group. Not reported how many schools the treated are from (are all retained students included and do all schools have retained students). Unclear whether matching is done within schools or across schools. The matching procedure was done by taking into account 40 variables and interaction terms that are theoretically relevant for the prediction of grade repetition. The predictors cover socio-demographics (age, gender, migration background, SES, parental education, home possessions), school marks in the 9th grade in five subjects (mathematics, German, biology, physics and chemistry) and tests dealing with cognitive ability and achievement results in mathematics and science. All these variables are baseline measures measured before the retention had occurred. However, the promoted students are a doubly selected group, because 1) they are promoted based on some evaluation, and 2) they do move on to 10th grade, which is not compulsory. |
| Evaluation Systems Design, Inc (1981) | US | Math, reading | Confounding | The control group are those qualifying for the programme but are not in grade 9. There is some imbalance on the pre-tests and nothing else is reported concerning the control group. |
| Fowler (2011) | US | Math | Confounding | No confounders controlled for in the analysis, and there is imbalance on gender and grade. |
| Franz et al. (2010) | US | Math | Other bias | 5 teachers participate, however only 1 teacher administers the intervention. Intervention effect is therefore confounded with the teacher effect. |
| Fuchs et al. (1999) | US | Reading | Confounding | No information about assignment and why some teachers get the intervention and others do not. Some pre-intervention imbalances, although not on test scores. The (non-blinded) teachers select the children to be measured after assignment. Comparison of gain scores or raw post-intervention test scores, no other confounders are controlled for. |
| Ganschow (1995) | US | Reading | Other bias | Intervention and control group are located in two different schools, in two different parts of the USA (Baltimore and Cincinnati). Intervention effect is confounded with school/region effect. |
| Gernert (2015) | US | Reading | Confounding | No confounders described or controlled for. Furthermore, intervention and control groups belong to different cohorts. |
| Gickling et al. (1989) | US | Math | Incomplete outcome data | Large attrition from a very small group, 13 out of 30 students do not complete. Attrition in numbers is not that different between intervention and control, 7 and 10 remain from control and treatment groups, but the causes are not fully explained. Control group students seem to drop out more often, and intervention students seem to stay in their classes partially as a result of the intervention. Furthermore, 5 out 7 control group students that stay in study have a very large increase in teacher grade scores from pre-test to the test taken just after randomization, i.e. attrition does not seem random. |
| Girolami (2009) | US | Reading | Confounding | No confounders are described by the researcher, although the data has been collected on gender and special education status. Moderate to severe imbalances between intervention and control groups. The comparison is between a group of barely proficient readers and a group of non-proficient readers, which opens up for regression to the mean problems. Regular OLS regression is used at analysis stage, however none of the confounding variables are included in any of the models. |
| Gonzalez (1996) | US | Math, reading | Confounding | For the effect sizes we could use, the study compare students whose parents refused participation in the language programme with those whose parents accepted. This is a very selected sample. No other controls for confounding. |
| Gottfried (2012) | US | Math, reading | Confounding | Compare retained students to all other students. The implicit assumption is that retained students, conditional on covariates, should have had the same result as the others, had they not been retained. This assumption seems unlikely to hold. |
| Graham & Pegg (2010) | Australia | Math | Confounding | Low achieving students are compared to normal achieving students at the same age from the same schools. Data for the years 2001-2008, but no control students for the year 2002. |
| Graham & Pegg (2013) | Australia | Math | Confounding | Low achieving students compared to normal achieving students at the same age from the same schools. |
| Hall (2004) | US | Math | Other bias | Intervention and control groups are placed in two separate schools. Intervention effect is confounded with the school effect. |
| Halterman (2013) | US | Reading | Confounding | Two classes (same teacher), repeated measure (before/after) design with alternating treatments. |
| Harris (2011) | US | Math | Confounding | Only control for pre-test scores in a linear regression analysis. The pre-test scores are very unbalanced. |
| Hawkins (2011) | US | Reading, math | Confounding | A cohort who did not get the intervention is compared to a cohort that did get an intervention. There are no tests for balanced samples on any covariate. No controls for confounding is used, the authors uses a t-test of whether cohort means are different. |
| Heiney (1998) | US | Math | Confounding | No confounders are controlled for, use regular t-test used to asses differences between control and intervention groups. |
| Holmes & Hwang (2016) | US | Math | Other bias | Control and intervention students at two different schools. Intervention effect is confounded with school effect. |
| Hopkins (1996) | US | Reading | Confounding | Large and systematic pre-intervention differences on most test scores, sometimes up to almost 1 standard deviation, consistently favouring the control group. |
| Hudson (2015) | US | Math, reading | Confounding | No large imbalances on the confounders considered but age, grade level, SES and pre-test are not considered and nothing is controlled for in the analysis. Only one-way ANOVA is applied with no adjustments or control of confounding variables whatsoever. |
| Interactive (2002) | US | Reading | Confounding | Randomisation failed. They control only for pre-test. There is selection in the schools and districts who report results. |
| Jackson (2016) | US | Reading | Confounding | No confounders considered. |
| Johnson (2013) | US | Math | Confounding | No confounders considered. |
| Johnson & Syropoulos (1996) |  | Math | Incomplete outcome data | Missing data level 66%/51% for the intervention group and 52%/43% for the control group (first/second semester). Nothing considered or discussed in relation to missing data. |
| Jones (2011) | US | Math | Confounding | Repeated measures ANOVA is applied to asses difference in gain. However the analysis compares each group separately, and as such it only assess whether any of the groups had a significant growth in test-score, not whether one group had a larger gain or post-score than the other group. |
| Jones-Mason (2012) | US | Math, reading | Confounding | No confounders are considered. Regular independent samples t-test is used to compare post-test scores of control and intervention groups. |
| Karuza (2014) | US | Math | Other bias | Match one intervention school to control schools, thus intervention effect is confounded with school effects. |
| Kerridge (2012) | US | Math | Other bias | In the pilot study: One treated and one control classroom, so confounding of intervention with class- and teacher effects. Similar problems in the two "case studies", at best 2 intervention classes and 1 control class in these. |
| Knuchel (2010) | US | Reading | Confounding | No information on confounders given and nothing is controlled for in the analysis. No explanation for why some students get the Ramp-Up programme and others do not in these years. The likelihood of selection into the programme seems high. Implicit but rough matching on pre-test grades from the procedure of creating intervention and control groups, but no formal test of balance. |
| Kubick (2008) | US | Reading, math | Confounding | Highly selected sample, participants chosen by a disciplinary officer. Some, but not overly large imbalances on pre-tests and characteristics. No confounders controlled for in the analysis. |
| LaChance (2012) | US | Math | Other bias | Treated students are randomly placed in a class and receives instruction from a teacher using “looped” instruction in year two of the study, but was receiving instruction from the teacher in year one. But there is only one looping teacher in year two, so cannot separate intervention effect from teacher effect. |
| Lakins (2016) | US | Math, reading | Confounding | Compare regular students to students receiving interventions a response to intervention system. |
| Lazarev et al. (2010) | US | Reading | Confounding | Large differences between intervention and control at pre-test is said to be eliminated by their standardization procedure (subtract school by year by grade averages from the means). As there are treated and control classes in each school and grade, this procedure should not eliminate any pre-test differences, just re-scale them. |
| Levin (1989) | US | Reading | Other bias | Randomisation seems to have been compromised for several of the teachers. Large imbalances at pre-test, more than 0.5 SDs in most cases. No control for confounding in the analysis. |
| Lewis (2009) | US | Math | Confounding | The 2005–2006 scores for informal geometry were compared with 2006–2007 scores (treated cohort), so intervention effects are confounded with cohort effects. Some of the students repeated the informal geometry in 2006/2007 because they failed in 2005. The researcher taught all classes in both school years. One-tailed t-tests are performed and no confounders are considered. |
| Lowenstein (1982) | US | Math, reading | Confounding | Age and IQ scores are included as confounders. The analysis is too descriptive to draw any conclusions about effect of intervention, and no information about SD/variance of means is provided to calculate any unadjusted effect size. |
| Mackay (2006) | UK | Reading | Confounding | Except for a pre-test, no confounder is described. The pre-test is not used in the analysis. Students are said to be matched, but there is no description of how this matching is done or any formal test of balance (a figure is shown). There is no other information about how pupils where selected. |
| Maldonado (1994) | US | Math, reading | Other bias | The bilingual special education teacher was assigned to the experimental group, and the traditional special education teacher was assigned to the control group. Intervention effects are confounded with teacher effects. |
| Martinez (2012) | US | Reading | Other bias | Unclear how randomisation was performed and likely to have been compromised. There are large differences in the number of intervention and control students without any mention of oversampling one group. Possibly because students without continuous enrolment were excluded. Parents or guardians could request that there students were not placed in the intervention programme, possibly after allocation. There are large and systematic imbalances on variables measured pre-intervention, although for the 10^th^ grade group there are no pre-tests or other characteristics reported. |
| Mason (2013) | US | Math, reading | Other bias | They have 159 students with disabilities to match and are able to form 68 (and 67) matched pairs. On average (incl SD were relevant) the intervention and control groups are exactly identical for both the math and reading groups. This seems like an error, as the participants were matched on IQ within a 7,5 point range (i.e. a 7,5 difference in IQ scores between control- and intervention group participants acted as a “cutoff”, p. 58). |
| Maxwell (2010) | US | Math, reading | Confounding | The pre-test is described, but results are not reported. Not possible to assess imbalances. Compare gain scores of at-risk students, who either participate or decide not to participate in a supplemental education service offer, in a t-test. There is no control for any confounder on this selected sample. |
| McCart (1996) | US | Math | Other bias | Intervention group from one school and control group from one other only. |
| McWhorter (2010) | US | Math, reading | Confounding | No confounders except pre-test considered or controlled for. Participants assigned to intervention or control groups by school staff. No further information on why some students were assigned to intervention and control groups. Pre-test differences are almost one standard deviation in literacy and somewhat smaller but still very large for math. |
| Miller (2011) | US | Math, reading | Confounding | No control for confounders despite differences by design in terms of pre-test scores. |
| Moore (2015) | US | Math | Other bias | One intervention and one control school, so intervention is confounded with school effects. |
| Mothus & Lapadat (2006) | Canada | Reading | Confounding | No control for confounders. Selection into intervention and control groups. |
| Nave (2007) | US | Math, reading | Confounding | No confounders are controlled for. |
| Nazzal (2012) | US | Math | Confounding | There are 116 students in the baseline sample, made up of three different selection criteria (marginal to poor attendance, failure in school and had been referred to the office at least once during the school year for misbehaviour) with respectively 28, 38 and 31 students; which does not add up to 116. The study sample consists of 58 students who volunteered to tutor, of which 47 become tutors. The remaining did not volunteer and served as control group. No information given on how volunteering differs between the three selection criteria. |
| Nesbitt (2007) | US | Reading | Confounding | Compares three cohorts, of which one is control. So the intervention effect is confounded by the cohort effects. There are relatively large imbalances on pre-tests. |
| Neubert (2017) | US | Reading | Confounding | Compare students receiving READ 180 and students not receiving READ 180. Groups are predetermined by the school district based on a cut-off test score on the RI. Control group seems to consist of average performing in addition to low performing students. Hence, groups are not comparable. No pre-tests shown but large imbalances on e.g., gender and ethnicity. |
| Ney (2010) | US | Math, reading | Confounding | No confounders considered. Data was collected for four supplemental control groups consisting of the not low achieving students that did not receive the intervention during the years covered by the study. |
| Nidich et al. (2011) | US | Math, reading | Confounding | Some relevant confounders are briefly mentioned. But no statistics are presented. There are significant imbalances on the baseline test. Nothing else is controlled for. |
| Norris (2009) | US | Reading | Other bias | Not stated anywhere how large the sample is. Large differences between classes at baseline. Teachers teach both control and intervention classes, and are familiar with the researcher. |
| O’Byrne et al. (2006) | US | Math, reading | Confounding | No confounders considered. Where needed, teachers selected which of their classes would be in the intervention group. |
| O’Hare (2012) | US | Reading | Other bias | Treated from one school district, control from three others, so intervention effect is confounded by the school district effect. |
| Oldham (1983) | US | Math, reading | Confounding | As students with similar characteristics where not retained and matching and outcome variables are separated by many years (last outcomes in high school, matching on ability in second grade), there is a high risk of selection on unobserved variables. |
| Olson & Land (2007) | US | Reading | Confounding | Match on one variable. No confounders considered in the analysis. For relevant state and standardised tests, there are only significance levels reported in text or share of students passing/scoring above a certain percentile. |
| Palmer (2016) | US | Reading | Confounding | No baseline information about balance on pre-tests. |
| Pena (2009) | US | Math, reading | Other bias | Compares two cohorts, one intervention and one control. Thus, confounding of intervention effects with cohort effects. |
| Peralta (2012) | US | Math, reading | Other bias | Compares one intervention school to two comparison schools. Thus, complete confounding of intervention effects with school effects |
| Platko (2011) | US | Math | Confounding | There is no baseline imbalance test, and no information on which schools intervention and control groups are in. |
| Plony (2013) | US | Reading | Confounding | No information about selection into intervention, except that the assignment seems purposeful. There are relatively large imbalances over pre-determined characteristics. These imbalances are not shown by grade though, but the analysis is done by grade. Also fairly large imbalances on pre-test scores (shown by grade), consistently favouring the control group. |
| Reese (2010) | US | Reading | Other bias | Of the 55 qualified students at the one school 5 agreed to participate (and 1 was never made available for testing sessions due to her extra-curricular activities) and the numbers for the second school are 2 of 52. Students also leave during the study, and there is shifting of students between the groups after assignment. |
| Reimer (2013) | US | Math | Confounding/Other bias | Trial 1 compares takers to non-takers, without an explanation of why some get Algebra project and others do not (too high risk of bias on the confounding-item). Trial 2 and 3 is a comparison between one intervention school and one control schools, i.e. intervention effect is confounded by the school effect (too high risk of bias on the other bias-item). |
| Robledo et al. (1990) | US | Math, reading | Reporting bias | The results of the year 2 analysis are missing. |
| Rogers (2012) | US | Reading | Confounding | Study participants were selected from the researcher’s six reading classes, which consist of two sections for each grade. Due to the small district population only half or 31 students, actually participated in the BEAR reading programme while the other half served as a comparison group. ANCOVA with pre-test as covariate but unclear description. No other confounders are controlled for. |
| Russell (2012) | US | Reading | Other bias | The intervention group was chosen for the study because they were assigned to the researcher’s reading class, so intervention effects are completely confounded by teacher/class effects. |
| Russell (2014) | US | Math | Other bias | Treated students belong to one cohort in one school, and control to one earlier cohort in the same school. The intervention effect is therefore completely confounded by cohort effects. |
| Sacchetto (2014) | US | Reading | Confounding | No control for any confounders. Participants of the study were selected from two separate high schools in the same district, which contribute eight and four classes/programmes to the intervention group from two cohorts. Two earlier cohorts of similar students from the same schools make up the control group. |
| Samuels (1984) | Canada | Math, reading | Other bias | The effect of the teacher cannot be separated from the effect of the experimental programme. |
| Scott (1999) | US | Reading | Other bias | Two classes from school C formed intervention group and one class each from school A and B was control, i.e. intervention effect is confounded by the school effect. |
| Schneyderman (2001) | US | Math | Confounding | Large imbalance on limited English proficiency students. Furthermore, there is substantial attrition and balance is just checked on the full sample, not the sample actually used in the analysis. |
| Skelton (2017) | US | Math, reading | Other bias | Only one control school, i.e., intervention effects are confounded with school effects. |
| Soriano et al. (2011) | Spain | Reading | Confounding | Mean age difference between intervention and control group is almost 2 years. |
| Starling et al. (2012) | Australia | Reading | Other bias | Randomise two schools to either intervention or control, so not possible to separate intervention effect from school effect |
| Trautman (2005) | US | Math | Other bias | Intervention effects are confounded with school effects, as there is only one intervention school. |
| Urbina (2011) | US | Math, reading | Other bias | One intervention and one control school, i.e. intervention effects are confounded with school effects. |
| Urbina et al. (2017) | US | Math, reading | Other bias | One intervention and one control school, i.e. intervention effects are confounded with school effects. |
| Watson-Barrow (2011) | US | Reading | Confounding | The study does not have access to proper pre-tests, for some students the tests are made after the intervention has begun. The effect of each intervention compared to traditional instruction cannot be separated out from the presentation of the results. |
| Westera & Moore (1995) | New Zealand | Reading | Confounding | No confounders are controlled for and teachers volunteer to be in the study, control students are from classes where teachers did not volunteer. |
| Whitten (2017) | US | Math | Confounding | Large imbalances at pre-test and no confounders are controlled for. |
| Wilks (2012) | US | Reading | Other bias | One high school model employed a single ELL high school English teaching model, while another high school model housed co-taught high school ELL English classes. At the middle school level, one set of classes utilized co-taught ELL reading classes, and the other middle school classes were taught by a single reading teacher. |
| Williams (2013) | US | Math | Other bias | 3 intervention schools and 1 control school, intervention effect is confounded by the school effect. |
| Winkler et al. (2013) | US | Reading | Confounding | Intervention group is self-selecting into treatment, which should reasonably mean that they are different on unobservable variables that are not included in the matching procedure. |
| Woodruff et al. (2002) | US | Reading | Other bias | Intervention students are at one school and control students in another school, treatment effect is confounded by the school effect. |
| Zeuschner (2005) | US | Reading | Confounding | No mentioning of why some students get the programme and others do not. Some tests seem to be measured at "intake" (p. 39), which could potentially be very long before the intervention. The pre-test scores used for matching are not shown. |

1. This risk of bias model was introduced by Prof. Reeves at a workshop on risk of bias in non-randomised studies at SFI Campbell, February 2011. The model is a further development of work carried out in the Cochrane Non-Randomised Studies Method Group (NRSMG). [↑](#footnote-ref-1)
2. See User guide for unobservables. [↑](#footnote-ref-2)
